# Synthesis of Small Libraries of Natural Products: Part II: Identification of a New Natural Product from the Essential Oil of *Pleurospermum austriacum* (L.) Hoffm. (Apiaceae)

**DOI:** 10.3390/molecules28124574

**Published:** 2023-06-06

**Authors:** Niko S. Radulović, Marko Z. Mladenović, Milan S. Dekić, Fabio Boylan

**Affiliations:** 1 Department of Chemistry, Faculty of Sciences and Mathematics, University of Niš, Višegradska 33, 18000 Niš, Serbia; markohem87@gmail.com; 2 Department of Sciences and Mathematics, State University of Novi Pazar, Vuka Karadžića 9, 36300 Novi Pazar, Serbia; mdekic@np.ac.rs; 3 School of Pharmacy and Pharmaceutical Sciences, Panoz Institute, and Trinity Biomedical Sciences Institute, Trinity College Dublin, Dublin 2, D02 PN40 Dublin, Ireland

**Keywords:** synthetic library, esters, NMR, GC-MS, structure elucidation, *Pleurospermum austriacum*

## Abstract

Herein, comprehensive data of NMR, MS, IR, and gas chromatography (RI) obtained by GC-MS on commonly used capillary columns of different polarity (non-polar DB-5MS and polar HP-Innowax) of a series of esters of all constitutional isomers of hexanoic acid with a homologous series of ω-phenylalkan-1-ols (phenylmethanol, 2-phenylethanol, 3-phenylpropan-1-ol, 4-phenylbutan-1-ol, and 5-phenylpentan-1-ol) and phenol, in total 48 chemical entities, were collected. The created synthetic library allowed the identification of a new constituent of the *P. austriacum* essential oil (3-phenylpropyl 2-methylpentanoate). The accumulated spectral and chromatographical data, as well as the established correlation between RI values and structures of regioisomeric hexanoates, provide (phyto)chemists with a tool that will make future identification of related natural compounds a straightforward task.

## 1. Introduction

Minor contributors of essential oils (EOs) and other plant extracts are often neglected due to their difficult identification and isolation from natural sources. On the other side, the importance of minor phytoconstituents has been proven on many occasions, for example, with the discovery of 1-*p*-menthene-8-thiol as a key odorant in grapefruit juice several decades ago. In the same manner, some of the constituents labeled as ‘minors’ quite often appeared to be responsible for the observed biological, pharmacological, or olfactory properties, reminding us of the potential significance of these compounds [1,2,3].

Identification of minor compounds in complex mixtures such as EOs has always represented a demanding task for phytochemists, especially when one considers the challenging isolation of such compounds for structural characterization. A combination of retention indices (RIs) and mass spectral (MS) data remains the most useful approach for the identification of such elusive mixtures of natural products. A thorough analysis of the RI values and fragmentation data in the corresponding mass spectra by experienced interpreters gives us possible candidates of the “minors” and an opportunity for their identification by use of a “synthetic approach”, which includes the synthesis of small libraries of selected compounds and their use in the identification procedure. Despite all its advantages, the identification based on the comparison of MS and RI data also has its drawbacks. For example, a thorough analysis of the RI values and fragmentation pattern in the corresponding mass spectra seem to be satisfactory primarily in distinguishing volatile esters of the homologous acids with the same alcohol and *vice versa*. However, any distinction between the isomeric compounds (e.g., between the isomeric acids, alcohols, and/or their derivatives) is sometimes difficult to ascertain. An additional problem is that RI and MS data for the esters derived from some of the most common alcohols and acids are lacking in the literature. Our previous analysis of the EO of *Pleurospermum austriacum* (L.) Hoffm. (Apiaceae) represents a good example of the difficulties in the identification of natural products with quite simple chemical structures [4]. Using data obtained from the RI and MS analyses, we were only able to make a tentative identification of the detected EO constituent as the ester of 3-phenylpropan-1-ol with one of the hexanoic acid isomers [4]. According to a detailed SciFinder search, only two reports had RI data referring to 3-phenylpropyl hexanoate isomers; however, these data introduced even greater confusion [5,6]. In particular, Barros-Castillo and coworkers identified 3-phenylpropyl hexanoate as one of the major EO constituents of *Artocarpus heterophyllus* with a RI value of 1705 [5]. The tentatively identified 3-phenylpropyl hexanoate (originally named hydrocinnamyl hexanoate) in the slightly older manuscript had a RI value 46 units higher on the GC column of the same polarity as that used in the report mentioned above [6]. The reasonable explanation is that they misidentified the compounds, and this was obviously the result of the scarcity of references and literature dealing with the MS and RI data needed for their differentiation. Even with sufficient literature data, it may be difficult to distinguish between isomeric compounds in the identification process solely based on the comparison of MS and RI data, as we described above as one of the shortcomings of the identification methodology. Sometimes, but not always, the co-injection of pure reference compounds could be used to identify isomeric compounds and differentiate them from one another. As we previously discussed, the use of pure reference compounds and their GC-coinjection cannot provide accurate identification of isomeric compounds due to several reasons including significant inter-laboratory differences in RI data of the same compounds, insufficient column selectivity to elute the isomeric compounds as resolved peaks, etc. [7]. As shown previously, and especially for the identification of components that show similar chromatographic behavior, the best RI datasets are those developed in a single laboratory with fixed operating conditions [7]. These facts justify the use of a different, more reliable analytical procedure to address the issue of accurate identification of isomeric compounds. This so-called “synthetic approach” involves the synthesis of a small library of selected compounds and allows their use in multiple co-injection procedures to eliminate any chance of misidentification [7,8].

Considering the above, we decided to create a small combinatorial library of the esters of regioisomeric hexanoic acids (2-ethylbutanoic, 2,2-dimethylbutanoic, 3,3-dimethylbutanic, 2,3-dimethylbutanic, 2-methylpentanoic, 3-methylpentanoic, 4-methylpentanoic, and hexanoic acid) with 3-phenylpropan-1-ol. In the aim to investigate a structure–chromatographic behavior relationship of such hexanoates, we expanded the library using phenol and additional alcohols (phenol, phenylmethanol, 2-phenylethanol, 4-phenylbutan-1-ol, and 5-phenylpentan-1-ol) that are encountered among the EO constituents. All synthesized compounds (48 esters in total, 24 of which were new compounds) were spectrally (IR, MS, 1D- and 2D-NMR) and chromatographically characterized (GC-retention data determined on two columns of different polarities). Comprehensive spectral and chromatographic data from the created library allowed a straightforward identification of the 3-phenylpropyl 2-methylpentanoate as the *P. austriacum* EO constituent that turned out to be a completely new natural product.

## 2. Results and Discussion

### 2.1. Synthetic Library of the Isomeric Hexanoates

**Figure 1:**
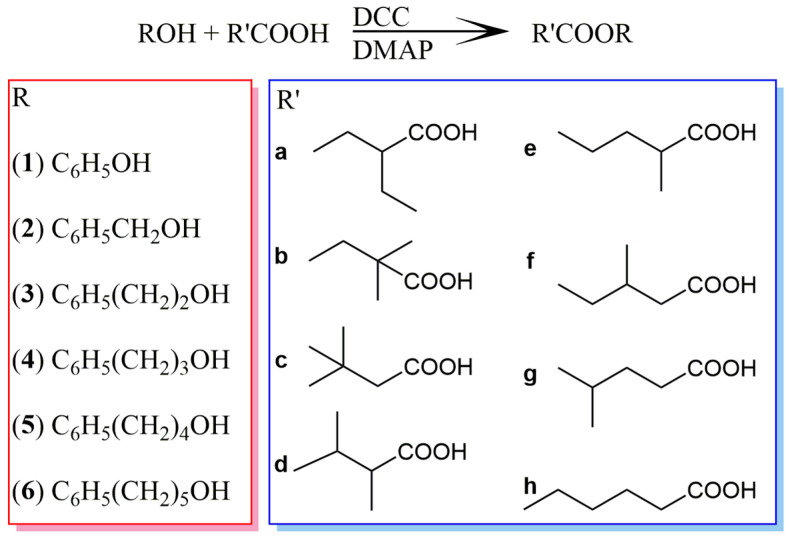
Synthetic scheme and the structures of the synthesized esters.

Esters of 3-phenylpropan-1-ol (**4**) with all constitutional isomers of hexanoic acid (2-ethylbutanoic (**a**), 2,2-dimethylbutanoic (**b**), 3,3-dimethylbutanoic (**c**), 2,3-dimethylbutanoic (**d**), 2-methylpentanoic (**e**), 3-methylpentanoic (**f**), 4-methylpentanoic (**g**), and hexanoic acid (**h**)) were synthesized using the standard Steglich procedure (Figure 1). Motivated by the previous reports from the literature with a systematic library of MS and RI data that made the identification of related natural compounds a straightforward task [7,8,9], alongside 3-phenylpropyl hexanoates, we prepared additional members of the synthetic library using phenol (**1**) and four other homologous alcohols (phenylmethanol (**2**), 2-phenylethanol (**3**), 4-phenylbutan-1-ol (**5**), and 5-phenylpentan-1-ol (**6**)). Mentioned esters were obtained in varying yields from 48 to 95%. Esters of acids with branching in position 2 (2-ethylbutanoic and 2,2-dimethylbutanoic acids) had the lowest final esterification yield, calculated after chromatographic purification of the obtained samples, whereas esters of other used regioisomeric hexanoic acids had a much higher final yield, greater than 70%. The used alcoholic moieties did not have a significant impact on the reaction yield (Supplementary Materials).

A recent SciFinder search of the Chemical Abstracts Service (CAS) database revealed that 24 of the 48 synthesized esters represented new compounds (**3d**, **3e**, **4b**–**4g**, **5a**–**5h**, and **6a**–**6h** in Figure 1), whereas more than 10 other esters from the library were for the first time spectrally (MS, IR, 1D, and 2D NMR) and/or chromatographically (RI) characterized (**1a**–**1c**, **2a**–**2c**, **3a**–**3c**, **3f**, **4a**, and **4h**).

### 2.2. Mass Spectrometry (MS) and Chromatographic (RI) Data

Over time, we are more and more convinced that such compounds, with a quite simple structure but with a lot of possible regioisomers, are very often simply neglected by researchers due to the lack of mass spectra and, especially, chromatographic retention indices values in the literature. This represents a vicious circle due to which the opportunity for identification and isolation/synthesis of many unknown natural products with great biological, pharmacological, and chemical potential was missed. In such a case, straightforward identification, based only on MS and RI data, is practically impossible. The reasons are that differences in the RI values in some cases are less than 10 units and fragmentation patterns visible in mass spectra of the esters of regioisomeric hexanoic acids with the same alcohol were almost identical (Supplementary Materials). The only possible approach to resolve that Gordian Knot was the use of the above-mentioned “synthetic approach” and “multiple co-injection procedure” which includes the creation of a small library with the MS, RI, and NMR data of all possible regioisomeric hexanoates and their co-injection with a sample of the essential oil/plant extract.

**Figure 2:**
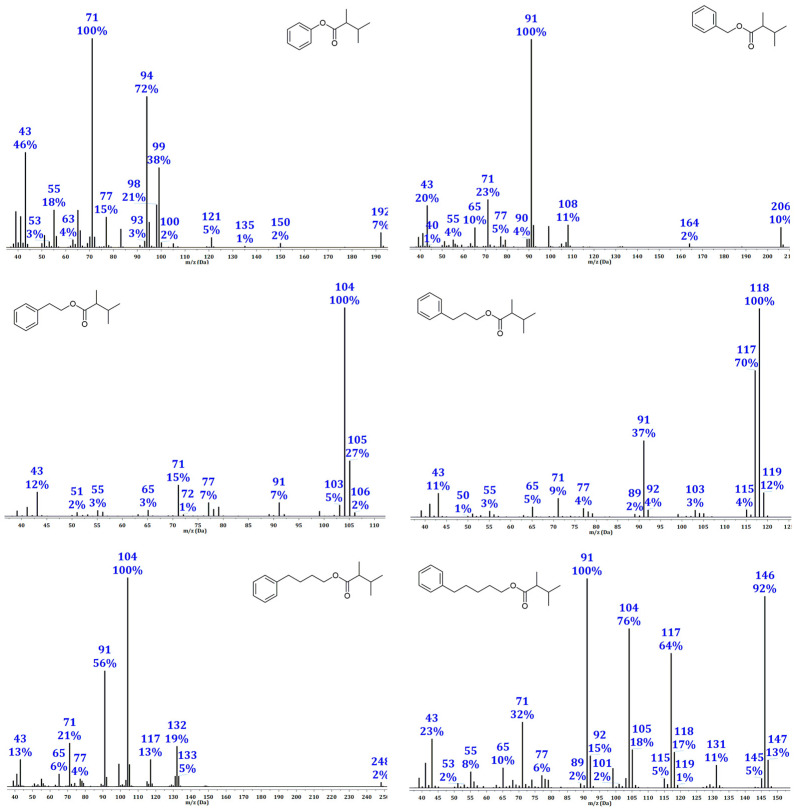
Typical representatives of mass spectra of phenyl, benzyl, phenethyl, 3-phenylpropyl, 4-phenylbutyl, and 5-phenylpentyl esters of regioisomeric hexanoic acids (randomly chosen esters of 2,3-dimethylbutanoic acid).

The electron impact (EI) mass spectra of the synthesized esters revealed fragmentation taking place in both segments of the molecules, i.e., the alcoholic part, with phenyl or phenyl-substituted alkyl chain, and hexanoic acid moiety (Figure 2). As expected, the intensity of the molecular ion peak [M^+^], otherwise of low intensity, decreases with increasing molecular weight, while in the spectra of some esters, it is completely absent (Figure 2 and Supplementary Materials). All synthesized compounds showed typical fragmentation processes under EI conditions. The ions formed by α-cleavage next to the carbonyl group appeared in mass spectra of all synthesized esters as the signals at *m*/*z* 71 and 99 corresponding to [C_5_H_11_]^+^ and [C_6_H_11_O]^+^, respectively. Benzylic cleavage is represented by the signal at *m*/*z* 91 in the spectra of all synthesized esters except those of phenol. On the other hand, the phenyl esters undergo an onium reaction with the ejection of acyl residues, giving the stable phenol cation a base peak at *m*/*z* 94 of **1a**–**h**. This fragmentation reaction also occurs in benzyl esters **2a**–**h** at *m*/*z* 108, although to a lesser extent. A McLafferty rearrangement that occurs under the influence of a C=O bond formed the signals at *m*/*z* 104, 118, 132, and 146, in the spectra of **3a**–**h**, **4a**–**h**, **5a**–**h**, and **6a**–**h**, respectively. The resulting product of McLafferty rearrangement of the esters **6a**–**h** undergoes additional McLafferty rearrangement under the influence of a resulting C=C bond giving the signal found at *m*/*z* 104 (corresponding to the loss of *m*/*z* 42). The signal at *m*/*z* 117 in the esters derived from alcohols having aliphatic chains with at least three C atoms (**4a**–**h**, **5a**–**h**, and **6a**–**h**) can be explained as a product of “McLafferty+1 rearrangement” (McLafferty rearrangement that involves the migration of two H atoms) that gives the C_6_H_13_O_2_ fragment ion.

The mass fragmentation pattern in the spectra of esters of regioisomeric hexanoic acids with the same alcohol, which can be crucial for their identification based on MS spectra, was almost identical. For that reason, MS spectra can give only reliable data about the structure of alcohol whereas the identity of the acid part remains unknown. The lack of [M^+^] in mass spectra of phenethyl, 3-phenylpropyl, and 5-phenylpentyl esters makes the identification of such compounds even harder. In that case, acquiring RI data can significantly contribute to the identification of mentioned esters. For those reasons, the collection of the values of the experimentally determined retention indices for all synthesized esters was carried out using the two commonly used GC columns of different polarities (DB-5MS and HP-Innowax). These data were presented in Table 1.

**Figure 3:**
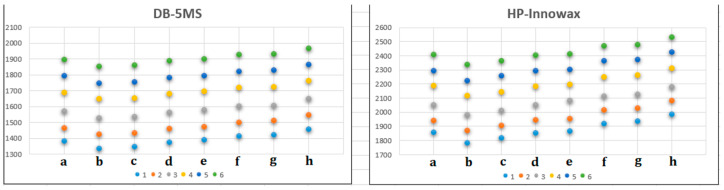
The dependence of RI values (DB-5MS and HP-Innowax columns) on the structure of the synthesized esters of 2-ethylbutanoic (**a**), 2,2-dimethylbutanoic (**b**), 3,3-dimethylbutanoic (**c**), 2,3-dimethylbutanoic (**d**), 2-methylpentanoic (**e**), 3-methylpentanoic (**f**), 4-methylpentanoic (**g**), and hexanoic acid (**h**).

Analysis of the obtained RI data (Table 1) showed that esters from the same alcohol and different isomeric hexanoic acids always elute in the same order from both used GC columns (Table 1): 2,2-dimethylbutanoates, 3,3-dimethylbutanoates, 2,3-dimethylbutanoates, 2-ethylbutanoates, 2-methylpentanoates, 3-methylpentanoates, 4-methylpentanoates, and hexanoates (Figure 3). Although RI values between isomers with the lowest (2,2-dimethylbutanoates) and highest RI data (hexanoates) covered a relatively large portion of the GC chromatograms (ΔRI = 116–133 units), several subgroups of isomers were found to almost co-elute at the DB-5MS column (e.g., 2,2-dimethylbutanoates and 3,3-dimethylbutanoates (ΔRI = 5–10 units), as well as 3-methylpentanoates and 4-methylpentanoates (ΔRI = 4–9 units)). Contrary to this, at the polar HP-Innowax column, close RI values were noted between 2-ethylbutanoates and 2,3-dimethylbutanoates (ΔRI = 0–3 units). Calculated RI increments within the homologous series of esters of the phenol and 2-phenylmethanol (ΔRI = 83–91 units) were quite different when compared with RI increments between the esters of the alcohols **2**–**3**, **4**–**5**, and **5**–**6** (ΔRI = 95–105 units). Surprisingly, the highest RI increment was noted between the esters of the 3-phenylpropan-1-ol and 4-phenylbutan-1-ol (ΔRI = 116–120 units; Figure S145, Supplementary Materials). One might speculate that the additional methylene group, somehow, has a differing impact on the molecular change of 4-phenylbutyl esters compared to the lower and higher members of the series.

The slope of the linear dependences of the esters with the same acid moiety and different alcoholic parts (Figures S146–S153, Supplementary Materials) corresponds to the average increment of RI per methylene group in the series, and the values (103–105 at DB-5MS) are in a general agreement with those assigned to an *n*-alkane series (RI increment of 100). The average increment of RI per methylene group was slightly higher at the used polar GC column (112–113). The values of y-intercepts, however, are significantly different for the homologous series (e.g., at DB-5MS values for the intercepts were 1348, 1223, 1233, 1260, 1276, 1302, 1310, and 1348 for series **a**–**h**, respectively). This means that the present branch(es) determines the base value of the retention indices. The presence of the α-ethyl branch, one or two methyl branches, and the position of the branch drastically alter the y-intercept. Thus, a direct regioisomeric relationship between the branch(es) and the ester carbonyl group is an important structural feature that determines the value of RI. Obtained very close values of y-intercepts for 2,2- and 3,3-dimethylbutanoates (1223 and 1233, respectively), as well as for 3- and 4-methylpentanoates (1302 and 1310, respectively) suggested that mentioned esters had very close RI values in general. Surprisingly, the much greater difference between mentioned y-intercepts at the used polar GC column (36 and 16 units, respectively) suggested an easier distinction between these regioisomeric hexanoates. Thus, GC analysis of (essential oil) samples with such regioisomers should be performed on two GC columns with different polarities.

Closer inspections of the literature data (NIST Chemistry WebBook, SRD 69) suggested that obtained correlations between branch(es) and RI data can be used for the tentative prediction of the structures of other homologous series of regioisomers. For example, in a recent report, the published RI data of methyl 2-methylnonadecanoate, 18-methylnonadecanoate, 17-methylnonadecanoate, and eicosanoate (RI = 2260, 2288, 2296, and 2324, respectively) were in agreement with calculated ΔRI for *iso*-, *anteiso*-, and 2-methyl branched isomers, i.e., 4-methylpentanoates, 3-methylpentanoates, and 2-methylpentanoates, compared to n-hexanoates [10]. Thus, if a mass spectral fragmentation indicates an ester of hexanoic acid, the data from the created library can help researchers, through the use of ΔRI between regioisomeric hexanoates from Table 1, to predict the structure of the ester or, at least, the presence/absence of branches in the acidic moiety if not guess the full identity of the ester.

### 2.3. NMR Data

**Figure 4:**
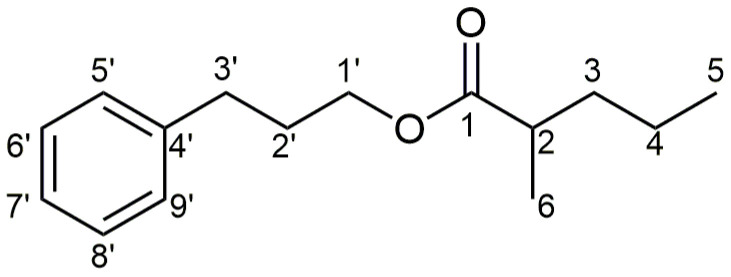
The structure of 3-phenylpropyl 2-methylpentanoate (**4e**) with the carbon atom numbering scheme.

For all compounds from the synthetic library, complete assignations (Figure 4) of ^1^H and ^13^C NMR signals were based on the detailed analysis of 1D (including homonuclear decoupling and ^1^H NMR simulation), and 2D NMR spectra (gradient ^1^H−^1^H correlation spectroscopy (COSY), heteronuclear multiple bond correlation (gHMBC), and heteronuclear single-quantum coherence (gHSQC) and nuclear Overhauser effect spectroscopy (NOESY)). Both ^1^H and ^13^C NMR spectra of the esters from the same subfamily of compounds differed only in the signals of the atoms from the acid moieties. The opposite was true when comparing the spectra of the esters of different alcohols and the same acid (**a–h**).

### 2.4. Identification of New Pleurospermum austriacum Metabolite

One of the key goals of this study was the determination of the structure of the previously detected *P. austriacum* essential oil constituent [4]. Unfortunately, this constituent could not be isolated from the essential oil sample due to its low relative abundance (less than 0.1%) and the complexity of the essential oil, with 156 detected essential oil constituents. At the time of the EO screening and the detection of the compound in question, the literature data that was available at the time allowed us only a tentative identification of the compound as a rare naturally occurring ester of 3-phenylpropan-1-ol with one of the eight possible constitutional isomers of hexanoic acid. Surprisingly, even now, after more than ten years, the MS and RI data on the mentioned esters are still quite scarce, with only one report with Rt data for 3-phenylpropyl 2-ethylbutanoate [11] and two with RI data for 3-phenylpropyl hexanoate [5,6]. However, the mentioned reports did not help us with the unambiguous GC-MS identification due to the incoherence of the presented RI data of the 3-phenylpropyl hexanoate, since the authors reported unusually different RI values on the same GC column (RI = 1705 and 1751) [5,6]. It was clearly a matter of misidentification of different isomeric hexanoic esters of 3-phenylpropan-ol, which, in turn, was expected and a very common issue in the literature in the case of isomeric compounds since they have similar mass spectra and sometimes very close RI values. Thus, the final structural confirmation of the tentatively identified ester could not be relied solely on MS and RI criteria, but require a thorough analysis of the MS, RI, ^1^H, and ^13^C NMR data of all possible isomeric hexanoates. Collected spectral and chromatographical data from the created synthetic library, as well as GC co-injection of the synthesized esters with *P. austriacum* essential oil, provided unambiguous information that led to a straightforward identification of the mentioned *P. austriacum* fruit essential oil constituent as 3-phenylpropyl 2-methylpentanoate that represent a new natural compound.

## 3. Materials and Methods

### 3.1. General Experimental Procedures

All solvents (*n*-hexane, diethyl ether (Et_2_O), tetrahydrofuran (THF), dichloromethane (DCM), ethyl acetate (EtOAc), methanol, and deuterated chloroform (CDCl_3_); HPLC grade), anhydrous MgSO_4_, anhydrous K_2_CO_3_, diethyl malonate, methyl iodide, ethyl iodide, isopropyl iodide, sodium hydride (60% dispersion in mineral oil), phenol, benzyl alcohol, phenethyl alcohol, 3-phenylpropan-1-ol, 4-phenylbutan-1-ol, 5-phenylpentan-1-ol, 4-(dimethylamino)pyridine (DMAP), *N*,*N*′-dicyclohexylcarbodiimide (DCC) and corresponding acids (2,2-dimethylbutanoic, 3,3-dimethylbutanoic, 2-methylpentanoic (mixture of stereoisomers), 3-methylpentanoic (mixture of stereoisomers), 4-methylpentanoic, and hexanoic acid) were purchased from Sigma-Aldrich (St Louis, MO, USA). Two hydrocarbon mixtures (Sigma-Aldrich (St Louis, MO, USA)), ranging from heptane to icosane and from heneicosane to tetracontane, were used for the determination of retention indices.

Silica gel 60, particle size distribution 40–63 mm, was used for “dry-flash” chromatography, whereas precoated Al silica gel plates (Merck (Darmstadt, Germany), Kieselgel 60 F_254_, 0.2 mm) were used for analytical TLC analyses. The spots on TLC were visualized by UV light (254 nm) and by spraying with 50% (*v*/*v*) aq. H_2_SO_4_ or 10% (*w*/*v*) ethanolic solution of phosphomolybdic acid, followed by 10 min heating at 110 °C. IR measurements (ATR-attenuated total reflectance) were carried out using a Thermo Nicolet model 6700 FTIR instrument (Waltham, MA, USA).

### 3.2. Gas Chromatography-Mass Spectrometry (GC-MS) Analyses

The GC-MS analyses (three repetitions) of the obtained samples were carried out using a Hewlett-Packard 6890N gas chromatograph equipped with a fused silica capillary column (DB-5MS (5% diphenyl-(95% dimethyl)polysiloxane, 30 m × 0.25 mm, film thickness 0.25 μm)) and coupled with a 5975B mass selective detector from the same company. The injector and interface were operated at 250 °C and 300 °C, respectively. The oven temperature was raised from 70 °C to 290 °C at a heating rate of 5 °C/min and the program ended with an isothermal period of 10 min. As a carrier gas helium at 1.0 mL/min was used. The samples, 1.0 μL of the Et_2_O solutions of the esters, were injected in a pulsed split mode (the flow was 1.5 mL/min for the first 0.5 min and then set to 1.0 mL/min throughout the remainder of the analysis; split ratio 40:1). MS conditions were as follows: ionization voltage of 70 eV, acquisition mass range 35–650, scan time 0.32 s. The GC-MS analyses were also performed on an Agilent Technologies 7890B gas chromatograph equipped with an HP-Innowax capillary column (polyethylene glycol stationary phase, 30 m × 0.25 mm, film thickness 0.25 μm, Agilent Technologies, Santa Clara, CA, USA) and coupled with a 240-MS ion trap detector (Agilent Technologies, Santa Clara, CA, USA). The injector and interface were operated at 220 and 230 °C, respectively. The oven temperature was raised from 40 to 220 °C at a heating rate of 7 °C/min and, then, isothermally held for 10 min. Helium at 1.0 mL/min was used as a carrier gas. The samples were injected with a split ratio of 40:1. The MS conditions were as follows: trap, ion source, and manifold temperatures were 100, 180, and 50 °C, respectively, ionization energy 70 eV, acquisition mass range 35–500 amu, and scan data rate of 2.08 Hz. The linear retention indices were determined relative to the retention times of C_13_–C_26_ *n*-alkanes on both columns [12].

### 3.3. NMR Measurements

The ^1^H (including ^1^H NMR selective homonuclear decoupling experiments), ^13^C (with and without heteronuclear decoupling) nuclear magnetic resonance (NMR) spectra, distortionless enhancement by polarization transfer (DEPT90 and DEPT135), and 2D (NOESY, and gradient ^1^H–^1^H COSY, HSQC, and HMBC) NMR spectra were recorded on a Bruker Avance III 400 MHz NMR spectrometer (Fällanden, Switzerland; ^1^H at 400 MHz, ^13^C at 101 MHz) equipped with a 5–11 mm dual ^13^C/^1^H probe head. All NMR spectra were measured at 25 °C in CDCl_3_ with tetramethylsilane (TMS) as an internal standard. Chemical shifts are reported in ppm (*δ*) and referenced to TMS (*δ*_H_ = 0 ppm) in ^1^H NMR spectra and/or to solvent protons (deuterated chloroform: *δ*_H_ = 7.26 ppm and *δ*_C_ = 77.16 ppm) in ^13^C and heteronuclear 2D spectra. The samples were dissolved in 1 mL of the solvent, and 0.7 mL of the solutions were transferred into a 5 mm Wilmad, 528-TR-7 NMR tube. The acquired NMR experiments, both 1D and 2D, were recorded using standard Bruker built-in pulse sequences. In some cases, the values of chemical shifts and coupling constants were determined by a simulation of the ^1^H NMR spectrum (manual iterative full spin analysis) of synthesized esters using MestReNova 11.0.3 software (tools/spin simulation).

### 3.4. Synthesis of 2,3-Dimethylbutanoic Acid

Solutions of dimethyl malonate (7.76 g; 48.5 mmol), isopropyl iodide (15 g; 88.2 mmol), and anhydrous K_2_CO_3_ (32.5 g; 240 mmol) in dry acetone (75 mL) were vigorously stirred and refluxed for 8 h and, then left overnight at ambient temperature. The solvent was evaporated and the remaining slurry was diluted with H_2_O (150 mL) and extracted with Et_2_O (4 × 50 mL). The organic layers were dried over anhydrous MgSO_4_ and concentrated under reduced pressure to yield a crude mixture (11.5 g) that was further fractionated by distillation under reduced pressure (in total 6 fractions). GC-MS analyses of the obtained fractions confirmed that fraction 6 (residue in the flask; 2.69 g) represented pure diethyl 2-isopropylmalonate. After that, in a suspension of sodium hydride (1.1 eq), washed from the mineral oil with dry hexane, in freshly distilled dry THF (70 mL) was added diethyl 2-isopropylmalonate (2.5 g; 12.4 mmol) and, after 10 min, methyl iodide (4 eq; 49.5 mmol). After vigorously stirring at ambient temperature (2 h) reaction was quenched by the addition of NaCl water solution (100 mL). THF was evaporated and the water layer was extracted with Et_2_O (5 × 70 mL). Combined ether extracts were concentrated under reduced pressure to yield crude diethyl 2-isopropyl-2-methylmalonate (2.55 g). Afterward, a mixture of crude dialkylated diethyl malonate (11.8 mmol), an aqueous solution of NaOH (60 mmol in 120 mL), and EtOH (40 mL) was stirred and refluxed for 4 h. EtOH was evaporated, and the water layer was washed with Et_2_O (2 × 50 mL), then acidified with 1 M HCl and extracted with Et_2_O (5 × 70 mL). Combined ether extracts were concentrated under reduced pressure to yield crude 2-isopropyl-2-methylmalonic acid (837 mg) that was subsequently decarboxylated by heating at 210 °C for 2 h under nitrogen. The obtained dark oil was purified by dry-flash chromatography on SiO_2_ using mixtures of the increasing polarity of hexane and EtOAc as the eluent to give pure 2,3-dimethylbutanoic acid (522 mg).

### 3.5. Synthesis of 2-Ethylbutanoic Acid

A solution of dimethyl malonate (2.0 g; 12.5 mmol), ethyl iodide (4 eq; 7.8 g; 50.0 mmol), and sodium hydride (4 eq; 1.2 g; 50.0 mmol) in dry THF (50 mL) were vigorously stirred for 3 h and, then the reaction was quenched by the addition of NaCl water solution (40 mL). THF was evaporated and the remaining water layer was extracted with Et_2_O (4 × 50 mL). The organic layers were dried over anhydrous MgSO_4_ and concentrated under reduced pressure to give a crude product (2.6 g) that, according to the GC-MS analysis, represented pure diethyl 2,2-diethylmalonate. Afterward, a mixture of crude dialkylated diethyl 2,2-diethylmalonate (2.5 g; 11.6 mmol), an aqueous solution of NaOH (46 mmol in 25 mL), and EtOH (10 mL) was stirred and refluxed for 3 h. EtOH was evaporated, and the water layer was washed with Et_2_O (2 × 20 mL), then acidified with 1 M HCl and extracted with Et_2_O (5 × 40 mL). Combined ether extracts were concentrated under reduced pressure to yield crude 2,2-diethylmalonic acid (1.89 g) that was subsequently decarboxylated by heating at 210 °C for 2 h under nitrogen. The obtained dark oil was purified by dry-flash chromatography on SiO_2_ using mixtures of the increasing polarity of hexane and EtOAc as the eluent to give pure 2-ethylbutanoic acid (572 mg).

### 3.6. Synthesis of Esters

A solution of the appropriate alcohol (phenol (**1**), phenylmethanol (**2**), 2-phenylethanol (**3**), 3-phenylpropan-1-ol (**4**), 4-phenylbutan-1-ol (**5**), and 5-phenylpentan-1-ol (**6**)), carboxylic acid (1.1 eq; 2-ethylbutanoic (**a**), 2,2-dimethylbutanoic (**b**), 3,3-dimethylbutanoic (**c**), 2,3-dimethylbutanoic (**d**), 2-methylpentanoic (**e**), 3-methylpentanoic (**f**), 4-methylpentanoic (**g**), and hexanoic acid (**h**)), DMAP (0.3 eq) and DCC (1.1 eq) in 30 mL of dry DCM was stirred overnight, at room temperature, in a round bottom flask equipped with a CaCl_2_ guard tube. The precipitated urea was filtered off and the filtrate was concentrated under a vacuum. The resulting residue was purified by “dry-flash” chromatography using mixtures of hexane and Et_2_O of increasing polarity for elution. Esters were washed from the column with 10% (*v*/*v*) Et_2_O in hexane. The purity of the ester fractions was checked by TLC and GC-MS. The yield of the esterification step, spectral data (NMR, MS, and IR), and assignments of ^1^H and ^13^C signals for the synthesized esters are given below and in the Supplementary Materials (Figures S1–S144).

*Phenyl 2-ethylbutanoate* (**1a**), Yield: 48%; IR (cm^−1^) 2964, 2935, 2877, 1753, 1594, 1493, 1458, 1384, 1372, 1266, 1193, 1157, 1110, 1069, 1024, 927, 899, 823, 744, 688; ^1^H NMR (400 MHz, CDCl_3_) 7.40–7.34 (2H, multiplet, H-3′ and H-5′), 7.26–7.17 (1H, multiplet, H-4′), 7.09–7.05 (2H, multiplet, H-2′ and H-6′), 2.4570* (1H, triplet of triplets, *J* = 8.9, 5.5 Hz, H-2), 1.7813* (1H, doublet of doublets of doublets, *J* = −13.5, 8.9, 7.4 Hz, H-3b), 1.7804 * (1H, doublet of doublets of doublets, *J* = −13.5, 8.9, 7.4 Hz, H-5b), 1.6614* (1H, doublet of doublets of doublets, *J* = −13.5, 7.4, 5.5 Hz, H-3a), 1.6608* (1H, doublet of doublets of doublets, *J* = −13.5, 7.4, 5.5 Hz, H-5a), 1.0240 * (6H, triplet, *J* = 7.4 Hz, H-4 and H-6); ^13^C NMR (101 MHz, CDCl_3_) 174.70 (C-1), 150.82 (C-1′), 129.37 (C-3′ and C-5′), 125.66 (C-4′), 121.63 (C-2′ and C-6′), 48.96 (C-2), 25.13 (C-3 and C-5), 11.86 (C-4 and C-6); MS (EI), *m*/*z* (%) 192 (2), 99 (36), 98 (26), 94 (100), 77 (15), 71 (91), 66 (9), 65 (18), 55 (18), 43 (46), 41 (15). * The values of chemical shift and coupling constants were determined by a simulation of the ^1^H NMR spectrum (manual iterative full spin analysis).

*Phenyl 2,2-dimethylbutanoate* (**1b**), Yield: 52%; IR (cm^−1^) 2969, 2935, 2877, 1750, 1593, 1494, 1474, 1458, 1389, 1366, 1314, 1234, 1192, 1161, 1108, 1070, 1056, 1008, 930, 912, 871, 831, 781, 737, 688; ^1^H NMR (400 MHz, CDCl_3_) 7.40–7.33 (2H, multiplet, H-3′ and H-5′), 7.24–7.18 (1H, multiplet, H-4′), 7.07–7.02 (2H, multiplet, H-2′ and H-6′), 1.73 (2H, quartet, *J* = 7.5 Hz, H-3), 1.31 (6H, singlet, H-5 and H-6), 0.98 (3H, triplet, *J* = 7.5 Hz, H-4); ^13^C NMR (101 MHz, CDCl_3_) 176.52 (C-1), 151.06 (C-1′), 129.33 (C-3′ and C-5′), 125.56 (C-4′), 121.55 (C-2′ and C-6′), 42.95 (C-2), 33.38 (C-3), 24.68 (C-5 and C-6), 9.36 (C-4); MS (EI), *m*/*z* (%) 192 (4), 99 (12), 95 (8), 94 (100), 77 (8), 71 (48), 70 (12), 65 (10), 55 (9), 43 (20), 41 (10).

*Phenyl 3,3-dimethylbutanoate* (**1c**), Yield: 70%; IR (cm^−1^) 2970, 2934, 2875, 1751, 1592, 1494, 1470, 1388, 1360, 1235, 1193, 1162, 1072, 1057, 1009, 932, 870, 830, 737, 688; ^1^H NMR (400 MHz, CDCl_3_) 7.40–7.34 (2H, multiplet, H-3′, and H-5′), 7.26–7.17 (1H, multiplet, H-4′), 7.10–7.05 (2H, multiplet, H-2′, and H-6′), 2.44 (2H, singlet, H-2), 1.14 (9H, singlet, H-4–H-6); ^13^C NMR (101 MHz, CDCl_3_) 170.73 (C-1), 150.67 (C-1′), 129.37 (C-3′ and C-5′), 125.68 (C-4′), 121.66 (C-2′ and C-6′), 47.82 (C-2), 31.13 (C-3), 29.68 (C-4–C-6); MS (EI), *m*/*z* (%) 192 (3), 99 (73), 94 (100), 77 (21), 71 (17), 65 (25), 55 (9), 41 (25).

*Phenyl 2,3-dimethylbutanoate* (**1d**), Yield: 66%; IR (cm^−1^) 2965, 2935, 2875, 1754, 1593, 1493, 1457, 1388, 1369, 1352, 1231, 1196, 1178, 1161, 1110, 1070, 1023, 1003, 924, 848, 749, 688; ^1^H NMR (400 MHz, CDCl_3_) 7.40–7.34 (2H, multiplet, H-3′ and H-5′), 7.24–7.19 (1H, multiplet, H-4′), 7.09–7.04 (2H, multiplet, H-2′ and H-6′), 2.49 (1H, pseudo quintet, *J* = 7.0 Hz, H-2), 2.08 (1H, pseudo octet, *J* = 7.0 Hz, H-3), 1.25 (3H, doublet, *J* = 7.0 Hz, H-6), 1.05 (3H, doublet, *J* = 7.0 Hz, H-4), 1.01 (3H, doublet, *J* = 7.0 Hz, H-5); ^13^C NMR (101 MHz, CDCl_3_) 174.82 (C-1), 150.83 (C-1′), 129.36 (C-3′ and C-5′), 125.64 (C-4′), 121.58 (C-2′ and C-6′), 46.20 (C-2), 31.16 (C-3), 20.70 (C-4), 19.23 (C-5), 13.72 (C-6); MS (EI), *m*/*z* (%) 192 (4), 99 (37), 98 (25), 95 (15), 94 (84), 77 (16), 71 (100), 65 (19), 55 (19), 43 (53), 41 (17).

*Phenyl 2-methylpentanoate* (**1e**), Yield: 74%; IR (cm^−1^) 2960, 2934, 2874, 1754, 1593, 1493, 1456, 1379, 1273, 1194, 1160, 1113, 1069, 1050, 1025, 997, 912, 887, 852, 811, 743, 688; ^1^H NMR (400 MHz, CDCl_3_) 7.40–7.34 (2H, multiplet, H-3′ and H-5′), 7.26–7.16 (1H, multiplet, H-4′), 7.09–7.05 (2H, multiplet, H-2′ and H-6′), 2.70 (1H, pseudo sextet, *J* = 6.9 Hz, H-2), 1.85–1.75 (1H, multiplet, H_a_-3), 1.59–1.50 (1H, multiplet, H_b_-3), 1.49–1.40 (2H, multiplet, H-4), 1.29 (3H, doublet, *J* = 6.9 Hz, H-6), 0.97 (3H, triplet, *J* = 7.2, Hz H-5); ^13^C NMR (101 MHz, CDCl_3_) 175.33 (C-1), 150.87 (C-1′), 129.36 (C-3′ and C-5′), 125.63 (C-4′), 121.55 (C-2′ and C-6′), 39.44 (C-2), 35.93 (C-3), 20.45 (C-4), 17.00 (C-6), 13.99 (C-5); MS (EI), *m*/*z* (%) 192 (4), 99 (30), 98 (33), 94 (100), 77 (15), 71 (96), 69 (12), 65 (21), 55 (12), 43 (51), 41 (18).

*Phenyl 3-methylpentanoate* (**1f**), Yield: 80%; IR (cm^−1^) 2961, 2929, 2876, 1753, 1593, 1493, 1457, 1379, 1314, 1285, 1262, 1234, 1195, 1162, 1145, 1104, 1024, 937, 887, 812, 760, 687; ^1^H NMR (400 MHz, CDCl_3_) 7.40–7.34 (2H, multiplet, H-3′ and H-5′), 7.26–7.17 (1H, multiplet, H-4′), 7.10–7.04 (2H, multiplet, H-2′ and H-6′), 2.56 (1H, doublet of doublets, *J* = 14.8, 6.1 Hz, H_a_-2), 2.35 (1H, doublet of doublets, *J* = 14.8, 8.2 Hz, H_b_-2), 2.03 (1H, doublet of doublets of quartets of doublets of doublets, *J* = 8.2, 7.4, 6.7, 6.1, 5.7 Hz, H-3), 1.47 (1H, doublet of quartets of doublets, *J* = 13.3, 7.4, 5.7 Hz, H_a_-4), 1.33 (1H, pseudo doublet of quintets, *J* = 13.3, 7.4 Hz, H_b_-4), 1.04 (3H, doublet, *J* = 6.7 Hz, H-6), 0.95 (3H, triplet, *J* = 7.4 Hz, H-5); ^13^C NMR (101 MHz, CDCl_3_) 171.78 (C-1), 150.76 (C-1′), 129.38 (C-3′ and C-5′), 125.69 (C-4′), 121.60 (C-2′ and C-6′), 41.45 (C-2), 32.10 (C-3), 29.37 (C-4), 19.31 (C-6), 11.32 (C-5); MS (EI), *m*/*z* (%) 192 (10), 99 (80), 95 (14), 94 (100), 77 (20), 71 (63), 69 (21), 65 (23), 55 (10), 43 (51), 41 (22).

*Phenyl 4-methylpentanoate* (**1g**), Yield: 88%; IR (cm^−1^) 2956, 2929, 2871, 1755, 1593, 1493, 1469, 1387, 1368, 1329, 1269, 1194, 1161, 1141, 1096, 1070, 1024, 1007, 928, 897, 813, 747, 688; ^1^H NMR (400 MHz, CDCl_3_) 7.40–7.34 (2H, multiplet, H-3′ and H-5′), 7.24–7.19 (1H, multiplet, H-4′), 7.10–7.04 (2H, multiplet, H-2′ and H-6′), 2.60–2.52 (2H, multiplet, H-2), 1.73–1.61 (3H, overlapping peaks, H-3 and H-4), 0.96 (6H, doublet, *J* = 6.4 Hz, H-5 and H-6); ^13^C NMR (101 MHz, CDCl_3_) 172.50 (C-1), 150.75 (C-1′), 129.38 (C-3′ and C-5′), 125.69 (C-4′), 121.56 (C-2′ and C-6′), 33.72 (C-3), 32.50 (C-2), 27.73 (C-4), 22.25 (C-5 and C-6); MS (EI), *m*/*z* (%) 192 (10), 99 (38), 95 (18), 94 (100), 81 (49), 77 (14), 71 (13), 65 (17), 55 (21), 43 (46), 41 (16).

*Phenyl hexanoate* (**1h**), Yield: 86%; IR (cm^−1^) 2957, 2930, 2871, 1759, 1593, 1493, 1456, 1363, 1194, 1161, 1140, 1101, 1070, 1024, 1007, 932, 877, 813, 750, 689; ^1^H NMR (400 MHz, CDCl_3_) 7.40–7.34 (2H, multiplet, H-3′ and H-5′), 7.24–7.19 (1H, multiplet, H-4′), 7.10–7.05 (2H, multiplet, H-2′ and H-6′), 2.55 (2H, triplet, *J* = 7.5 Hz, H-2), 1.76 (2H, quintet, *J* = 7.5 Hz, H-3), 1.45–1.32 (4H, overlapping peaks, H-4 and H-5), 0.92 (3H, triplet, *J* = 7.2 Hz, H-6); ^13^C NMR (101 MHz, CDCl_3_) 172.23 (C-1), 150.75 (C-1′), 129.38 (C-3′ and C-5′), 125.69 (C-4′), 121.58 (C-2′ and C-6′), 34.37 (C-2), 31.27 (C-4), 24.64 (C-3), 22.33 (C-5), 13.94 (C-6); MS (EI), *m*/*z* (%) 192 (9), 99 (46), 95 (10), 94 (100), 77 (12), 71 (28), 65 (16), 55 (13), 43 (46), 41 (14).

*Benzyl 2-ethylbutanoate* (**2a**), Yield: 49%; IR (cm^−1^) 2958, 2930, 2870, 1753, 1593, 1493, 1455, 1362, 1190, 1161, 1140, 1075, 1007, 932, 876, 750, 689; ^1^H NMR (400 MHz, CDCl_3_) 7.40–7.29 (5H, multiplet, H-3′–H-7′), 5.13 (2H, singlet, H-1′), 2.2602* (1H, triplet of triplets, *J* = 8.9, 5.5 Hz, H-2), 1.6412* (1H, doublet of doublets of doublets, *J* = −13.5, 8.9, 7.4 Hz, H-3b), 1.6403* (1H, doublet of doublets of doublets, *J* = −13.5, 8.9, 7.4 Hz, H-5b), 1.5412* (1H, doublet of doublets of doublets, *J* = −13.5, 7.4, 5.5 Hz, H-3a), 1.5408* (1H, doublet of doublets of doublets, *J* = −13.5, 7.4, 5.5 Hz, H-5a), 0.8839* (6H, triplet, *J* = 7.4 Hz, H-4 and H-6); ^13^C NMR (101 MHz, CDCl_3_) 176.13 (C-1), 136.30 (C-2′), 128.50 (C-4′ and C-6′), 128.12 (C-3′ and C-7′), 128.08 (C-5′), 65.87 (C-1′), 48.92 (C-2), 25.05 (C-3 and C-5), 11.82 (C-4 and C-6); MS (EI), *m*/*z* (%) 206 (6), 92 (11), 91 (100), 89 (8), 77 (11), 71 (75), 65 (17), 55 (11), 43 (44), 41 (13). * The values of chemical shift and coupling constants were determined by a simulation of the ^1^H NMR spectrum (manual iterative full spin analysis).

*Benzyl 2,2-dimethylbutanoate* (**2b**), Yield: 51%; IR (cm^−1^) 2969, 2932, 1727, 1498, 1474, 1455, 1389, 1314, 1238, 1137, 1062, 1029, 1008, 968, 911, 793, 734, 696; ^1^H NMR (400 MHz, CDCl_3_) 7.39–7.28 (5H, multiplet, H-3′–H-7′), 5.11 (2H, singlet, H-1′), 1.59 (2H, quartet, *J* = 7.5 Hz, H-3), 1.18 (6H, singlet, H-5 and H-6), 0.82 (3H, triplet, *J* = 7.5 Hz, H-4); ^13^C NMR (101 MHz, CDCl_3_) 177.80 (C-1), 136.47 (C-2′), 128.46 (C-4′ and C-6′), 127.95 (C-3′ and C-7′), 127.79 (C-5′), 65.96 (C-1′), 42.71 (C-2), 33.36 (C-3), 24.66 (C-5 and C-6), 9.23 (C-4); MS (EI), *m*/*z* (%) 206 (7), 92 (9), 91 (100), 89 (6), 77 (8), 71 (80), 65 (16), 55 (6), 43 (46), 41 (11).

*Benzyl 3,3-dimethylbutanoate* (**2c**), Yield: 67%; IR (cm^−1^) 2955, 2932, 2868, 1731, 1498, 1455, 1367, 1321, 1224, 1125, 1045, 996, 891, 737, 696; ^1^H NMR (400 MHz, CDCl_3_) 7.38–7.29 (5H, multiplet, H-3′–H-7′), 5.10 (2H, singlet, H-1′), 2.25 (2H, singlet, H-2), 1.02 (9H, singlet, H-4–H-6); ^13^C NMR (101 MHz, CDCl_3_) 172.17 (C-1), 136.14 (C-2′), 128.50 (C-4′ and C-6′), 128.26 (C-3′ and C-7′), 128.11 (C-5′), 65.89 (C-1′), 47.93 (C-2), 30.83 (C-3), 29.64 (C-4–C-6); MS (EI), *m*/*z* (%) 206 (8), 131 (5), 108 (36), 99 (9), 92 (8), 91 (100), 77 (6), 65 (10), 57 (19), 41 (8).

*Benzyl 2,3-dimethylbutanoate* (**2d**), Yield: 65%; IR (cm^−1^) 2955, 2867, 1733, 1497, 1454, 1365, 1321, 1124, 1045, 999, 737, 695; ^1^H NMR (400 MHz, CDCl_3_) 7.39–7.28 (5H, multiplet, H-3′–H-7′), 5.11 (2H, singlet, H-1′), 2.29 (1H, pseudo quintet, *J* = 7.0 Hz, H-2), 1.94 (1H, pseudo octet, *J* = 7.0 Hz, H-3), 1.12 (3H, doublet, *J* = 7.0 Hz, H-6), 0.91 (3H, doublet, *J* = 7.0 Hz, H-4), 0.89 (3H, doublet, *J* = 7.0 Hz, H-5); ^13^C NMR (101 MHz, CDCl_3_) 176.25 (C-1), 136.24 (C-2′), 128.50 (C-4′ and C-6′), 128.12 (C-3′ and C-7′), 128.08 (C-5′), 65.90 (C-1′), 46.16 (C-2), 31.03 (C-3), 20.71 (C-4), 19.14 (C-5), 13.68 (C-6); MS (EI), *m*/*z* (%) 206 (2), 131 (4), 108 (44), 99 (12), 92 (6), 91 (100), 77 (8), 65 (13), 57 (11), 41 (5).

*Benzyl 2-methylpentanoate* (**2e**), Yield: 70%; IR (cm^−1^) 2959, 2933, 2873, 1732, 1498, 1455, 1381, 1350, 1230, 1213, 1171, 1142, 1081, 1028, 1004, 966, 910, 735, 696; ^1^H NMR (400 MHz, CDCl_3_) 7.39–7.28 (5H, multiplet, H-3′–H-7′), 5.11 (2H, singlet, H-1′), 2.50 (1H, sextet, *J* = 7.0 Hz, H-2), 1.72–1.62 (1H, multiplet, H_a_-3), 1.46–1.36 (1H, multiplet, H_b_-3), 1.35–1.24 (2H, multiplet, H-4), 1.16 (3H, doublet, *J* = 7.0 Hz, H-6), 0.89 (3H, triplet, *J* = 7.2 Hz, H-5); ^13^C NMR (101 MHz, CDCl_3_) 176.73 (C-1), 136.28 (C-2′), 128.51 (C-4′ and C-6′), 128.07 (C-3′ and C-7′), 128.01 (C-5′), 65.95 (C-1′), 39.32 (C-2), 35.92 (C-3), 20.37 (C-4), 17.01 (C-6), 13.94 (C-5); MS (EI), *m*/*z* (%) 206 (2), 164 (5), 108 (13), 99 (5), 92 (13), 91 (100), 77 (6), 71 (18), 65 (11), 43 (18), 41 (7).

*Benzyl 3-methylpentanoate* (**2f**), Yield: 75%; IR (cm^−1^) 2960, 2930, 2876, 1732, 1498, 1456, 1380, 1357, 1285, 1238, 1174, 1153, 1122, 1095, 978, 910, 736, 696; ^1^H NMR (400 MHz, CDCl_3_) 7.42–7.28 (5H, multiplet, H-3′–H-7′), 5.12 (2H, singlet, H-1′), 2.36 (1H, doublet of doublets, *J* = 14.7, 6.1 Hz, H_a_-2), 2.16 (1H, doublet of doublets, *J* = 14.7, 8.2 Hz, H_b_-2), 1.91 (1H, doublet of doublets of quartets of doublets of doublets, *J* = 8.2, 7.4, 6.7, 6.1, 5.7 Hz, H-3), 1.36 (1H, doublet of quartets of doublets, *J* = 13.2, 7.4, 5.7 Hz, H_a_-4), 1.23 (1H, pseudo doublet of quintets, *J* = 13.2, 7.4 Hz, H_b_-4), 0.93 (3H, doublet, *J* = 6.7 Hz, H-6), 0.88 (3H, triplet, *J* = 7.4 Hz, H-5); ^13^C NMR (101 MHz, CDCl_3_) 173.22 (C-1), 136.14 (C-2′), 128.52 (C-4′ and C-6′), 128.17 (C-3′ and C-7′), 128.14 (C-5′), 66.02 (C-1′), 41.47 (C-2), 31.95 (C-3), 29.33 (C-4), 19.27 (C-6), 11.27 (C-5); MS (EI), *m*/*z* (%) 206 (1), 108 (44), 107 (8), 97 (5), 92 (9), 91 (100), 90 (14), 79 (7), 73 (11), 71 (8), 43 (5).

*Benzyl 4-methylpentanoate* (**2g**), Yield: 80%; IR (cm^−1^) 2956, 2870, 1733, 1497, 1455, 1386, 1367, 1328, 1263, 1161, 1102, 1028, 970, 735, 696; ^1^H NMR (400 MHz, CDCl_3_) 7.40–7.29 (5H, multiplet, H-3′–H-7′), 5.11 (2H, singlet, H-1′), 2.39–2.34 (2H, multiplet, H-2), 1.64–1.51 (3H, overlapping peaks, H-3 and H-4), 0.89 (6H, doublet, *J* = 6.3 Hz, H-5 and H-6); ^13^C NMR (101 MHz, CDCl_3_) 173.88 (C-1), 136.10 (C-2′), 136.28 (C-2′), 128.53 (C-4′ and C-6′), 128.16 (C-3′, C-5′, and C-7′), 66.10 (C-1′), 33.72 (C-3), 32.40 (C-2), 27.67 (C-4), 22.22 (C-5 and C-6); MS (EI), *m*/*z* (%) 206 (1), 115 (23), 108 (44), 97 (15), 92 (17), 91 (100), 90 (11), 81 (11), 65 (12), 43 (13), 41 (9).

*Benzyl hexanoate* (**2h**),Yield: 78%; IR (cm^−1^) 2956, 2931, 2871, 1734, 1498, 1456, 1379, 1352, 1213, 1160, 1097, 1029, 994, 905, 734, 696; ^1^H NMR (400 MHz, CDCl_3_) 7.39–7.30 (5H, multiplet, H-3′–H-7′), 5.11 (2H, singlet, H-1′), 2.35 (2H, triplet, *J* = 7.6 Hz, H-2), 1.65 (2H, quintet, *J* = 7.6 Hz, H-3), 1.37–1.24 (4H, overlapping peaks, H-4 and H-5), 0.89 (3H, triplet, *J* = 6.9 Hz, H-6); ^13^C NMR (101 MHz, CDCl_3_) 173.73 (C-1), 136.14 (C-2′), 128.55 (C-4′ and C-6′), 128.17 (C-3′, C-5′, and C-7′), 66.08 (C-1′), 34.32 (C-2), 31.31 (C-4), 24.66 (C-3), 22.33 (C-5), 13.93 (C-6); MS (EI), *m*/*z* (%) 206 (6), 108 (40), 99 (16), 97 (7), 92 (23), 91 (100), 65 (19), 43 (10), 41 (11).

*Phenethyl 2-ethylbutanoate* (**3a**), Yield: 45%; IR (cm^−1^) 2963, 2934, 2876, 1730, 1674, 1605, 1497, 1456, 1385, 1363, 1267, 1228, 1171, 1145, 1085, 1045, 988, 812, 746, 698; ^1^H NMR (400 MHz, CDCl_3_) 7.32–7.27 (2H, multiplet, H-4′ and H-8′), 7.25–7.19 (3H, multiplet, H-5′–H-7′), 4.31 (2H, triplet, *J* = 7.0 Hz, H-1′), 2.94 (2H, triplet, *J* = 7.0 Hz, H-2′), 2.1801* (1H, triplet of triplets, *J* = 8.9, 5.5 Hz, H-2), 1.5911* (1H, doublet of doublets of doublets, *J* = −13.5, 8.9, 7.4 Hz, H-3b), 1.5906* (1H, doublet of doublets of doublets, *J* = −13.5, 8.9, 7.4 Hz, H-5b), 1.4912* (1H, doublet of doublets of doublets, *J* = −13.5, 7.4, 5.5 Hz, H-3a), 1.4908* (1H, doublet of doublets of doublets, *J* = −13.5, 7.4, 5.5 Hz, H-5a), 0.8301* (6H, triplet, *J* = 7.4 Hz, H-4 and H-6); ^13^C NMR (101 MHz, CDCl_3_) 176.17 (C-1), 137.90 (C-3′), 128.89 (C-5′ and C-7′), 128.43 (C-4′ and C-8′), 126.48 (C-6′), 64.48 (C-1′), 48.93 (C-2), 35.23 (C-2′), 25.00 (C-3 and C-5), 11.77 (C-4 and C-6); MS (EI), *m*/*z* (%) 105 (18), 104 (100), 91 (6), 79 (4), 78 (3), 77 (6), 71 (11), 55 (3), 43 (11), 41 (4). * The values of chemical shift and coupling constants were determined by a simulation of the ^1^H NMR spectrum (manual iterative full spin analysis).

*Phenethyl 2,2-dimethylbutanoate* (**3b**), Yield: 42%; IR (cm^−1^) 2969, 2931, 2856, 1726, 1604, 1497, 1474, 1454, 1380, 1362, 1239, 1047, 1019, 990, 890, 747, 698; ^1^H NMR (400 MHz, CDCl_3_) 7.32–7.27 (2H, multiplet, H-4′ and H-8′), 7.25–7.19 (3H, multiplet, H-5′–H-7′), 4.31 (2H, triplet, *J* = 7.0 Hz, H-1′), 2.94 (2H, triplet, *J* = 7.0 Hz, H-2′), 1.59 (2H, quartet, *J* = 7.5 Hz, H-3), 1.18 (6H, singlet, H-5 and H-6), 0.82 (3H, triplet, *J* = 7.5 Hz, H-4); ^13^C NMR (101 MHz, CDCl_3_) 177.80 (C-1), 137.90 (C-3′), 128.89 (C-5′ and C-7′), 128.43 (C-4′ and C-8′), 126.48 (C-6′), 64.48 (C-1′), 42.71 (C-2), 35.23 (C-2′), 33.36 (C-3), 24.66 (C-5 and C-6), 9.23 (C-4); MS (EI), *m*/*z* (%) 105 (20), 104 (100), 91 (5), 79 (4), 78 (3), 77 (6), 71 (17), 65 (3), 43 (11), 41 (4).

*Phenethyl 3,3-dimethylbutanoate* (**3c**), Yield: 60%; IR (cm^−1^) 2969, 2931, 2856, 1726, 1604, 1497, 1474, 1454, 1380, 1362, 1239, 1047, 1019, 990, 890, 747, 698; ^1^H NMR (400 MHz, CDCl_3_) 7.32–7.27 (2H, multiplet, H-4′ and H-8′), 7.24–7.19 (3H, multiplet, H-5′–H-7′), 4.28 (2H, triplet, *J* = 7.1 Hz, H-1′), 2.94 (2H, triplet, *J* = 7.1 Hz, H-2′), 2.17 (2H, singlet, H-2), 0.98 (9H, singlet, H-4–H-6); ^13^C NMR (101 MHz, CDCl_3_) 172.30 (C-1), 137.87 (C-3′), 128.87 (C-5′ and C-7′), 128.44 (C-4′ and C-8′), 126.49 (C-6′), 64.48 (C-1′), 47.97 (C-2), 35.16 (C-2′), 30.67 (C-3), 29.60 (C-4–C-6); MS (EI), *m*/*z* (%) 105 (45), 104 (100), 99 (5), 91 (8), 79 (5), 78 (4), 77 (7), 65 (4), 57 (17), 41 (6).

*Phenethyl 2,3-dimethylbutanoate* (**3d**), Yield: 68%; IR (cm^−1^) 2971, 2931, 2855, 1731, 1605, 1497, 1470, 1454, 1389, 1344, 1255, 1187, 1151, 1075, 1031, 988, 747, 698; ^1^H NMR (400 MHz, CDCl_3_) 7.33–7.27 (2H, multiplet, H-4′ and H-8′), 7.25–7.19 (3H, multiplet, H-5′–H-7′), 4.35–4.24 (2H, multiplet, H-1′), 2.94 (2H, triplet, *J* = 7.0 Hz, H-2′), 2.20 (1H, pseudo quintet, *J* = 7.0 Hz, H-2), 1.87 (1H, pseudo octet, *J* = 7.0 Hz, H-3), 1.07 (3H, doublet, *J* = 7.0 Hz, H-6), 0.87 (3H, doublet, *J* = 7.0 Hz, H-4), 0.85 (3H, doublet, *J* = 7.0 Hz, H-5); ^13^C NMR (101 MHz, CDCl_3_) 176.34 (C-1), 137.91 (C-2′), 128.89 (C-4′ and C-6′), 128.43 (C-3′ and C-7′), 126.48 (C-5′), 64.59 (C-1′), 46.19 (C-2), 35.18 (C-2′), 30.93 (C-3), 20.67 (C-4), 19.13 (C-5), 13.69 (C-6); MS (EI), *m*/*z* (%) 105 (26), 104 (100), 99 (8), 91 (7), 79 (6), 78 (6), 77 (8), 65 (5), 57 (12), 41 (5).

*Phenethyl 2-methylpentanoate* (**3e**), Yield: 62%; IR (cm^−1^) 2958, 2934, 2873, 1731, 1604, 1497, 1455, 1382, 1349, 1273, 1245, 1173, 1146, 1084, 1055, 1031, 746, 698; ^1^H NMR (400 MHz, CDCl_3_) 7.32–7.27 (2H, multiplet, H-4′ and H-8′), 7.25–7.19 (3H, multiplet, H-5′–H-7′), 4.29 (2H, multiplet, H-1′), 2.94 (2H, triplet, *J* = 7.0 Hz, H-2′), 2.50 (1H, pseudo sextet, *J* = 7.0 Hz, H-2), 1.65–1.54 (1H, multiplet, H_a_-3), 1.41–1.30 (1H, multiplet, H_b_-3), 1.29–1.19 (2H, multiplet, H-4), 1.10 (3H, doublet, *J* = 7.0 Hz, H-6), 0.87 (3H, triplet, *J* = 7.2 Hz, H-5); ^13^C NMR (101 MHz, CDCl_3_) 176.83 (C-1), 137.91 (C-3′), 128.91 (C-5′ and C-7′), 128.43 (C-4′ and C-8′), 126.48 (C-6′), 64.59 (C-1′), 39.32 (C-2), 35.92 (C-3), 35.17 (C-2′), 20.35 (C-4), 17.02 (C-6), 13.95 (C-5); MS (EI), *m*/*z* (%) 105 (18), 104 (100), 99 (4), 91 (4), 79 (6), 78 (4), 77 (5), 43 (9), 41 (12).

*Phenethyl 3-methylpentanoate* (**3f**), Yield: 75%; IR (cm^−1^) 2960, 2931, 2875, 1732, 1605, 1497, 1455, 1381, 1359, 1286, 1242, 1176, 1154, 1124, 1096, 1053, 1031, 1000, 748, 698; ^1^H NMR (400 MHz, CDCl_3_) 7.32–7.27 (2H, multiplet, H-4′ and H-8′), 7.24–7.19 (3H, multiplet, H-5′–H-7′), 4.29 (2H, triplet, *J* = 7.1 Hz, H-1′), 2.93 (2H, triplet, *J* = 7.1 Hz, H-2′), 2.28 (1H, doublet of doublets, *J* = 14.7, 6.1 Hz, H_a_-2), 2.08 (1H, doublet of doublets, *J* = 14.7, 8.1 Hz, H_b_-2), 1.85 (1H, doublet of doublets of quartets of doublets of doublets, *J* = 8.1, 7.4, 6.7, 6.1, 5.7 Hz, H-3), 1.32 (1H, doublet of quartets of doublets, *J* = 13.2, 7.4, 5.7 Hz, H_a_-4), 1.19 (1H, pseudo doublet of quintets, *J* = 13.2, 7.4 Hz, H_b_-4), 0.89 (3H, doublet, *J* = 6.7 Hz, H-6), 0.86 (3H, triplet, *J* = 7.4 Hz, H-5); ^13^C NMR (101 MHz, CDCl_3_) 173.32 (C-1), 137.90 (C-3′), 128.90 (C-5′ and C-7′), 128.47 (C-4′ and C-8′), 126.52 (C-6′), 64.63 (C-1′), 41.51 (C-2), 35.18 (C-2′), 31.91 (C-3), 29.30 (C-4), 19.25 (C-6), 11.28 (C-5); MS (EI), *m*/*z* (%) 105 (26), 104 (100), 99 (5), 91 (7), 79 (4), 78 (3), 77 (6), 43 (7), 41 (5).

*Phenethyl 4-methylpentanoate* (**3g**), Yield: 80%; IR (cm^−1^) 2956, 2870, 1732, 1605, 1497, 1468, 1454, 1386, 1367, 1329, 1245, 1164, 1103, 1055, 1031, 999, 748, 698; ^1^H NMR (400 MHz, CDCl_3_) 7.33–7.20 (5H, multiplet, H-4′–H-8′), 4.29 (2H, triplet, *J* = 7.1, Hz H-1′), 2.94 (2H, triplet, *J* = 7.1 Hz, H-2′), 2.32–2.26 (2H, multiplet, H-2), 1.58–1.44 (3H, overlapping peaks, H-3 and H-4), 0.88 (6H, doublet, *J* = 6.3 Hz, H-5 and H-6); ^13^C NMR (101 MHz, CDCl_3_) 174.01 (C-1), 137.89 (C-3′), 128.91 (C-5′ and C-7′), 128.47 (C-4′ and C-8′), 126.53 (C-6′), 64.73 (C-1′), 35.15 (C-2′), 33.75 (C-3), 32.39 (C-2), 27.63 (C-4), 22.22 (C-5 and C-6); MS (EI), *m*/*z* (%) 105 (27), 104 (100), 99 (3), 91 (7), 79 (5), 78 (4), 77 (6), 43 (10), 41 (5).

*Phenethyl hexanoate* (**3h**), Yield: 82%; IR (cm^−1^) 2955, 2930, 2860, 1732, 1604, 1497, 1454, 1381, 1353, 1242, 1165, 1098, 1031, 1001, 748, 698; ^1^H NMR (400 MHz, CDCl_3_) 7.33–7.28 (2H, multiplet, H-4′ and H-8′), 7.25–7.20 (3H, multiplet, H-5′–H-7′), 4.29 (2H, triplet, *J* = 7.1 Hz, H-1′), 2.94 (2H, triplet, *J* = 7.1 Hz, H-2′), 2.28 (2H, triplet, *J* = 7.5 Hz, H-2), 1.60 (2H, quintet, *J* = 7.5 Hz, H-3), 1.36–1.18 (4H, overlapping peaks, H-4 and H-5), 0.88 (3H, triplet, *J* = 7.0 Hz, H-6); ^13^C NMR (101 MHz, CDCl_3_) 173.82 (C-1), 137.88 (C-3′), 128.89 (C-5′ and C-7′), 128.46 (C-4′ and C-8′), 126.51 (C-6′), 64.69 (C-1′), 35.14 (C-2′), 34.30 (C-2), 31.27 (C-4), 24.63 (C-3), 22.32 (C-5), 13.91 (C-6); MS (EI), *m*/*z* (%) 105 (21), 104 (100), 99 (5), 91 (7), 79 (4), 78 (4), 77 (6), 43 (10), 41 (4).

*3-Phenylpropyl 2-ethylbutanoate* (**4a**), Yield: 52%; IR (cm^−1^) 2963, 2933, 2876, 1730, 1604, 1497, 1455, 1383, 1367, 1324, 1267, 1229, 1173, 1146, 1086, 1015, 945, 910, 743, 698; ^1^H NMR (400 MHz, CDCl_3_) 7.31–7.26 (2H, multiplet, H-6′ and H-8′), 7.22–7.16 (3H, multiplet, H-5′, H-7′, and H-9′), 4.11 (2H, triplet, *J* = 6.5 Hz, H-1′), 2.69 (2H, pseudo triplet, *J* = 7.5 Hz, H-3′), 2.2186* (1H, triplet of triplets, *J* = 8.9, 5.5 Hz, H-2), 2.00–1.92 (2H, multiplet, H-2′), 1.5912* (1H, doublet of doublets of doublets, *J* = −13.5, 8.9, 7.4 Hz, H-3b), 1.5906* (1H, doublet of doublets of doublets, *J* = −13.5, 8.9, 7.4 Hz, H-5b), 1.4914* (1H, doublet of doublets of doublets, *J* = −13.5, 7.4, 5.5 Hz, H-3a), 1.4907* (1H, doublet of doublets of doublets, *J* = −13.5, 7.4, 5.5 Hz, H-5a), 0.9101* (6H, triplet, *J* = 7.4 Hz, H-4 and H-6); ^13^C NMR (101 MHz, CDCl_3_) 176.29 (C-1), 141.24 (C-4′), 128.43 (C-6′ and C-8′), 128.40 (C-5′ and C-9′), 125.99 (C-7′), 63.32 (C-1′), 49.03 (C-2), 32.21 (C-3′), 30.42 (C-2′), 25.10 (C-3 and C-5), 11.89 (C-4 and C-6); MS (EI), *m*/*z* (%) 119 (10), 118 (100), 117 (78), 115 (3), 91 (33), 77 (4), 65 (6), 43 (12), 41 (6). * The values of chemical shift and coupling constants were determined by a simulation of the ^1^H NMR spectrum (manual iterative full spin analysis).

*3-Phenylpropyl 2,2-dimethylbutanoate* (**4b**), Yield: 48%; IR (cm^−1^) 2969, 2932, 2879, 1726, 1604, 1497, 1473, 1455, 1389, 1365, 1314, 1240, 1148, 1053, 1019, 989, 745, 698; ^1^H NMR (400 MHz, CDCl_3_) 7.31–7.26 (2H, multiplet, H-6′ and H-8′), 7.22–7.16 (3H, multiplet, H-5′, H-7′, and H-9′), 4.08 (2H, triplet, *J* = 6.4 Hz, H-1′), 2.69 (2H, doublet of doublets, *J* = 8.6, 6.8 Hz, H-3′), 2.00–1.91 (2H, multiplet, H-2′), 1.58 (2H, quartet, *J* = 7.5 Hz, H-3), 1.17 (6H, singlet, H-5 and H-6), 0.85 (3H, triplet, *J* = 7.5 Hz, H-4); ^13^C NMR (101 MHz, CDCl_3_) 177.99 (C-1), 141.26 (C-4′), 128.43 (C-6′ and C-8′), 128.40 (C-5′ and C-9′), 125.99 (C-7′), 63.45 (C-1′), 42.69 (C-2), 33.38 (C-3), 32.20 (C-3′), 30.37 (C-2′), 24.70 (C-5 and C-6), 9.32 (C-4); MS (EI), *m*/*z* (%) 119 (10), 118 (100), 117 (68), 92 (3), 91 (32), 77 (4), 65 (6), 43 (14), 41 (6).

*3-Phenylpropyl 3,3-dimethylbutanoate* (**4c**), Yield: 65%; IR (cm^−1^) 2955, 2868, 1730, 1604, 1497, 1473, 1465, 1454, 1366, 1321, 1226, 1197, 1128, 1046, 1019, 966, 912, 746, 698; ^1^H NMR (400 MHz, CDCl_3_) 7.31–7.26 (2H, multiplet, H-6′ and H-8′), 7.22–7.16 (3H, multiplet, H-5′, H-7′, and H-9′), 4.08 (2H, triplet, *J* = 6.6 Hz, H-1′), 2.69 (2H, pseudo triplet, *J* = 7.5 Hz, H-3′), 2.21 (2H, singlet, H-2), 2.00–1.91 (2H, multiplet, H-2′), 1.04 (9H, singlet, H-4–H-6); ^13^C NMR (101 MHz, CDCl_3_) 172.42 (C-1), 141.24 (C-4′), 128.42 (C-6′ and C-8′), 128.38 (C-5′ and C-9′), 125.98 (C-7′), 63.34 (C-1′), 48.04 (C-2), 32.28 (C-3′), 30.72 (C-3), 30.37 (C-2′), 29.47 (C-4–C-6); MS (EI), *m*/*z* (%) 119 (16), 118 (100), 117 (70), 115 (4), 92 (3), 91 (46), 77 (4), 65 (6), 41 (9).

*3-Phenylpropyl 2,3-dimethylbutanoate* (**4d**), Yield: 53%; IR (cm^−1^) 2930, 2855, 1731, 1604, 1497, 1453, 1389, 1345, 1298, 1257, 1188, 1152, 1122, 1076, 1045, 951, 890, 744, 698; ^1^H NMR (400 MHz, CDCl_3_) 7.31–7.26 (2H, multiplet, H-6′ and H-8′), 7.22–7.16 (3H, multiplet, H-5′, H-7′, and H-9′), 4.09 (2H, multiplet, H-1′), 2.69 (2H, doublet of doublets, *J* = 8.6, 6.8 Hz, H-3′), 2.24 (1H, quintet, *J* = 7.0 Hz, H-2), 2.00–1.86 (3H, overlapping peaks, H-3 and H-2′), 1.11 (3H, doublet, *J* = 7.0 Hz, H-6), 0.93 (6H, pseudo triplet, *J* = 6.7 Hz, H-4 and H-5); ^13^C NMR (101 MHz, CDCl_3_) 176.45 (C-1), 141.24 (C-4′), 128.42 (C-6′ and C-8′), 128.39 (C-5′ and C-9′), 125.98 (C-7′), 63.35 (C-1′), 46.26 (C-2), 32.22 (C-3′), 31.01 (C-3), 30.37 (C-2′), 20.75 (C-5), 19.21 (C-4), 13.79 (C-6); MS (EI), *m*/*z* (%) 119 (11), 118 (100), 117 (76), 92 (4), 91 (37), 77 (4), 71 (7), 65 (5), 43 (11), 41 (7).

*3-Phenylpropyl 2-methylpentanoate* (**4e**), Yield: 65%; IR (cm^−1^) 2957, 2934, 2873, 1731, 1604, 1497, 1454, 1379, 1273, 1240, 1174, 1147, 1085, 1054, 1029, 912, 743, 698; ^1^H NMR (400 MHz, CDCl_3_) 7.31–7.26 (2H, multiplet, H-6′ and H-8′), 7.24–7.14 (3H, multiplet, H-5′, H-7′, and H-9′), 4.12 (2H, multiplet, H-1′), 2.69 (2H, doublet of doublets, *J* = 8.7, 6.8 Hz, H-3′), 2.46 (1H, pseudo sextet, *J* = 7.0 Hz, H-2), 2.00–1.92 (2H, multiplet, H-2′), 1.71–1.59 (1H, multiplet, H_a_-3), 1.45–1.27 (3H, overlapping peaks, H_b_-3 and H-4), 1.15 (3H, doublet, *J* = 7.0 Hz, H-6), 0.92 (3H, triplet, *J* = 7.2 Hz, H-5); ^13^C NMR (101 MHz, CDCl_3_) 176.94 (C-1), 141.25 (C-4′), 128.43 (C-6′ and C-8′), 128.40 (C-5′ and C-9′), 125.99 (C-7′), 63.43 (C-1′), 39.38 (C-2), 35.99 (C-3), 32.18 (C-3′), 30.34 (C-2′), 20.44 (C-4), 17.11 (C-6), 13.98 (C-5); MS (EI), *m*/*z* (%) 119 (10), 118 (100), 117 (78), 92 (4), 91 (35), 77 (4), 71 (7), 65 (6), 43 (11), 41 (7).

*3-Phenylpropyl 3-methylpentanoate* (**4f**), Yield: 82%; IR (cm^−1^) 2959, 2875, 1732, 1496, 1454, 1380, 1363, 1243, 1177, 1125, 1095, 1019, 912, 745, 698; ^1^H NMR (400 MHz, CDCl_3_) 7.31–7.26 (2H, multiplet, H-6′ and H-8′), 7.22–7.16 (3H, multiplet, H-5′, H-7′, and H-9′), 4.09 (2H, triplet, *J* = 6.5 Hz, H-1′), 2.69 (2H, doublet of doublets, *J* = 8.7, 6.8 Hz, H-3′), 2.31 (1H, doublet of doublets, *J* = 14.6, 6.1 Hz, H_a_-2), 2.11 (1H, doublet of doublets, *J* = 14.6, 8.1 Hz, H_b_-2), 2.00 –1.83 (3H, overlapping peaks, H-3 and H-2′), 1.38 (1H, doublet of quartets of doublets, *J* = 13.2, 7.4, 5.7 Hz, H_a_-4), 1.19 (1H, pseudo doublet of quintets, *J* = 13.2, 7.4 Hz, H_b_-4), 0.94 (3H, doublet, *J* = 6.7 Hz, H-6), 0.90 (3H, triplet, *J* = 7.4 Hz, H-5); ^13^C NMR (101 MHz, CDCl_3_) 173.43 (C-1), 141.23 (C-4′), 128.42 (C-6′ and C-8′), 128.39 (C-5′ and C-9′), 125.99 (C-7′), 63.49 (C-1′), 41.53 (C-2), 32.22 (C-3′), 31.97 (C-3), 30.31 (C-2′), 29.34 (C-4), 19.30 (C-6), 11.30 (C-5); MS (EI), *m*/*z* (%) 119 (11), 118 (100), 117 (77), 92 (4), 91 (40), 77 (4), 71 (4), 65 (6), 43 (7), 41 (8).

*3-Phenylpropyl 4-methylpentanoate* (**4g**), Yield: 90%; IR (cm^−1^) 2955, 2869, 1732, 1603, 1497, 1454, 1386, 1367, 1329, 1247, 1166, 1104, 1028, 745, 698; ^1^H NMR (400 MHz, CDCl_3_) 7.32–7.26 (2H, multiplet, H-6′ and H-8′), 7.22–7.16 (3H, multiplet, H-5′, H-7′, and H-9′), 4.09 (2H, triplet, *J* = 7.1 Hz, H-1′), 2.69 (2H, doublet of doublets, *J* = 8.6, 6.8 Hz, H-3′), 2.34–2.27 (2H, multiplet, H-2), 2.00–1.92 (2H, multiplet, H-2′), 1.62–1.49 (3H, overlapping peaks, H-3 and H-4), 0.91 (6H, doublet, *J* = 6.3 Hz, H-5 and H-6); ^13^C NMR (101 MHz, CDCl_3_) 174.10 (C-1), 141.24 (C-4′), 128.42 (C-6′ and C-8′), 128.38 (C-5′ and C-9′), 125.98 (C-7′), 63.60 (C-1′), 33.82 (C-3), 32.42 (C-2), 32.41 (C-3′), 30.26 (C-2′), 27.69 (C-4), 22.24 (C-5 and C-6); MS (EI), *m*/*z* (%) 119 (12), 118 (100), 117 (78), 92 (4), 91 (40), 81 (4), 77 (4), 65 (5), 43 (10), 41 (7).

*3-Phenylpropyl hexanoate* (**4h**), Yield: 85%; IR (cm^−1^) 2955, 2930, 2860, 1733, 1604, 1496, 1454, 1389, 1360, 1243, 1167, 1098, 1020, 910, 744, 698; ^1^H NMR (400 MHz, CDCl_3_) 7.31–7.26 (2H, multiplet, H-6′ and H-8′), 7.22–7.16 (3H, multiplet, H-5′, H-7′, and H-9′), 4.09 (2H, triplet, *J* = 6.5 Hz, H-1′), 2.69 (2H, doublet of doublets, *J* = 8.6, 6.8 Hz, H-3′), 2.30 (2H, triplet, *J* = 7.5 Hz, H-2), 2.00–1.91 (2H, multiplet, H-2′), 1.63 (2H, quintet, *J* = 7.5 Hz, H-3), 1.39–1.23 (4H, overlapping peaks, H-4 and H-5), 0.90 (3H, triplet, *J* = 7.0 Hz, H-6); ^13^C NMR (101 MHz, CDCl_3_) 173.92 (C-1), 141.24 (C-4′), 128.42 (C-6′ and C-8′), 128.39 (C-5′ and C-9′), 125.98 (C-7′), 63.56 (C-1′), 34.33 (C-2), 32.20 (C-3′), 31.34 (C-4), 30.27 (C-2′), 24.71 (C-3), 22.34 (C-5), 13.93 (C-6); MS (EI), *m*/*z* (%) 119 (10), 118 (100), 117 (80), 115 (4), 92 (4), 91 (37), 77 (4), 65 (5), 43 (8), 41 (7).

*4-Phenylbutyl 2-ethylbutanoate* (**5a**), Yield: 55%; IR (cm^−1^) 2955, 2867, 1733, 1604, 1496, 1454, 1381, 1360, 1167, 1021, 991, 746, 698; ^1^H NMR (400 MHz, CDCl_3_) 7.31–7.26 (2H, multiplet, H-7′ and H-9′), 7.21–7.15 (3H, multiplet, H-6′, H-8′, and H-10′), 4.11 (2H, multiplet, H-1′), 2.65 (2H, multiplet, H-4′), 2.1901* (1H, triplet of triplets, *J* = 8.9, 5.5 Hz, H-2), 1.75–1.66 (4H, overlapping peaks, H-2′ and H-3′), 1.5913* (1H, doublet of doublets of doublets, *J* = −13.5, 8.9, 7.4 Hz, H-3b), 1.5905* (1H, doublet of doublets of doublets, *J* = −13.5, 8.9, 7.4 Hz, H-5b), 1.4919* (1H, doublet of doublets of doublets, *J* = −13.5, 7.4, 5.5 Hz, H-3a), 1.4912* (1H, doublet of doublets of doublets, *J* = −13.5, 7.4, 5.5 Hz, H-5a), 0.8802* (6H, triplet, *J* = 7.4 Hz, H-4 and H-6); ^13^C NMR (101 MHz, CDCl_3_) 176.36 (C-1), 142.07 (C-5′), 128.37 (C-7′ and C-9′), 128.33 (C-6′ and C-10′), 125.81 (C-8′), 63.86 (C-1′), 49.02 (C-2), 35.45 (C-4′), 28.33 and 27.78 (C-2′ and C-3′), 25.08 (C-3 and C-5), 11.86 (C-4 and C-6); MS (EI), *m*/*z* (%) 248 (1), 132 (12), 131 (5), 117 (10), 105 (11), 104 (100), 91 (39), 71 (30), 65 (5), 43 (11), 41 (5). * The values of chemical shift and coupling constants were determined by a simulation of the ^1^H NMR spectrum (manual iterative full spin analysis).

*4-Phenylbutyl 2,2-dimethylbutanoate* (**5b**), Yield: 51%; IR (cm^−1^) 2969, 2935, 2862, 1726, 1604, 1496, 1473, 1460, 1454, 1388, 1365, 1314, 1240, 1149, 1063, 1019, 990, 746, 698; ^1^H NMR (400 MHz, CDCl_3_) 7.31–7.26 (2H, multiplet, H-7′ and H-9′), 7.21–7.15 (3H, multiplet, H-6′, H-8′, and H-10′), 4.07 (2H, multiplet, H-1′), 2.64 (2H, multiplet, H-4′), 1.74–1.63 (4H, overlapping peaks, H-2′ and H-3′), 1.56 (2H, quartet, *J* = 7.5 Hz, H-3), 1.15 (6H, singlet, H-5 and H-6), 0.83 (3H, triplet, *J* = 7.5 Hz, H-4); ^13^C NMR (101 MHz, CDCl_3_) 178.10 (C-1), 142.09 (C-5′), 128.36 (C-7′ and C-9′), 128.33 (C-6′ and C-10′), 125.80 (C-8′), 64.06 (C-1′), 42.65 (C-2), 35.44 (C-4′), 33.37 (C-3), 28.27 and 27.77 (C-2′ and C-3′), 24.68 (C-5 and C-6), 9.29 (C-4); MS (EI), *m*/*z* (%) 248 (1), 132 (18), 131 (4), 117 (12), 105 (10), 104 (100), 91 (49), 71 (31), 65 (6), 43 (16), 41 (6).

*4-Phenylbutyl 3,3-dimethylbutanoate* (**5c**), Yield: 81%; IR (cm^−1^) 2953, 2866, 1730, 1604, 1496, 1473, 1465, 1454, 1322, 1226, 1128, 1046, 746, 698; ^1^H NMR (400 MHz, CDCl_3_) 7.31–7.25 (2H, multiplet, H-7′ and H-9′), 7.21–7.15 (3H, multiplet, H-6′, H-8′, and H-10′), 4.08 (2H, multiplet, H-1′), 2.64 (2H, pseudo triplet, *J* = 7.2 Hz, H-4′), 2.19 (2H, singlet, H-2), 1.74–1.62 (4H, overlapping peaks, H-2′ and H-3′), 1.02 (9H, singlet, H-4–H-6); ^13^C NMR (101 MHz, CDCl_3_) 172.46 (C-1), 142.03 (C-5′), 128.36 (C-7′ and C-9′), 128.32 (C-6′ and C-10′), 125.80 (C-8′), 63.83 (C-1′), 48.03 (C-2), 35.43 (C-4′), 30.70 (C-3), 29.65 (C-4–C-6), 28.27 and 27.83 (C-2′ and C-3′); MS (EI), *m*/*z* (%) 248 (1), 133 (10), 132 (21), 117 (12), 105 (10), 104 (100), 99 (14), 91 (68), 65 (7), 41 (10).

*4-Phenylbutyl 2,3-dimethylbutanoate* (**5d**), Yield: 64%; IR (cm^−1^) 2930, 2854, 1732, 1604, 1496, 1450, 1389, 1359, 1346, 1298, 1188, 1151, 1075, 1045, 891, 746, 698; ^1^H NMR (400 MHz, CDCl_3_) 7.31–7.26 (2H, multiplet, H-7′ and H-9′), 7.21–7.15 (3H, multiplet, H-6′, H-8′, and H-10′), 4.14–4.03 (2H, multiplet, H-1′), 2.64 (2H, multiplet, H-4′), 2.22 (1H, quintet, *J* = 7.0 Hz, H-2), 1.90 (1H, pseudo octet, *J* = 7.0 Hz, H-3), 1.75–1.60 (4H, overlapping peaks, H-2′, and H-3′), 1.09 (3H, doublet, *J* = 7.0 Hz, H-6), 0.91 (3H, doublet, *J* = 7.0 Hz, H-4), 0.90 (3H, doublet, *J* = 7.0 Hz, H-5); ^13^C NMR (101 MHz, CDCl_3_) 176.50 (C-1), 142.07 (C-5′), 128.37 (C-7′ and C-9′), 128.33 (C-6′ and C-10′), 125.81 (C-8′), 63.89 (C-1′), 46.26 (C-2), 35.46 (C-4′), 31.01 (C-3), 28.30 and 27.78 (C-2′ and C-3′), 20.73 (C-5), 19.20 (C-4), 13.77 (C-6); MS (EI), *m*/*z* (%) 248 (1), 132 (19), 117 (13), 105 (10), 104 (100), 99 (8), 91 (54), 71 (18), 65 (6), 43 (13), 41 (6).

*4-Phenylbutyl 2-methylpentanoate* (**5e**), Yield: 63%; IR (cm^−1^) 2956, 2934, 2872, 1731, 1604, 1496, 1454, 1378, 1352, 1240, 1175, 1147, 1085, 1031, 745, 698; ^1^H NMR (400 MHz, CDCl_3_) 7.31–7.26 (2H, multiplet, H-7′ and H-9′), 7.21–7.15 (3H, multiplet, H-6′, H-8′, and H-10′), 4.08 (2H, multiplet, H-1′), 2.65 (2H, multiplet, H-4′), 2.43 (1H, pseudo sextet, *J* = 7.0 Hz, H-2), 1.75–1.60 (5H, overlapping peaks, H_a_-3, H-2′, and H-3′), 1.44–1.25 (3H, overlapping peaks, H_b_-3 and H-4), 1.13 (3H, doublet, *J* = 7.0 Hz, H-6), 0.90 (3H, triplet, *J* = 7.2 Hz, H-5); ^13^C NMR (101 MHz, CDCl_3_) 177.00 (C-1), 142.08 (C-5′), 128.37 (C-7′ and C-9′), 128.33 (C-6′ and C-10′), 125.81 (C-8′), 63.98 (C-1′), 39.37 (C-2), 35.98 (C-3), 35.45 (C-4′), 28.28 and 27.75 (C-2′ and C-3′), 20.42 (C-4), 17.09 (C-6), 13.97 (C-5); MS (EI), *m*/*z* (%) 248 (1), 132 (17), 117 (12), 105 (10), 104 (100), 99 (6), 91 (49), 71 (16), 65 (6), 43 (12), 41 (6).

*4-Phenylbutyl 3-methylpentanoate* (**5f**), Yield: 92%; IR (cm^−1^) 2959, 2874, 1731, 1604, 1496, 1454, 1380, 1286, 1243, 1178, 1125, 1095, 1030, 746, 698; ^1^H NMR (400 MHz, CDCl_3_) 7.31–7.25 (2H, multiplet, H-7′ and H-9′), 7.21–7.15 (3H, multiplet, H-6′, H-8′, and H-10′), 4.08 (2H, multiplet, H-1′), 2.64 (2H, multiplet, H-4′), 2.30 (1H, doublet of doublets, *J* = 14.6, 6.1 Hz, H_a_-2), 2.11 (1H, doublet of doublets, *J* = 14.6, 8.1 Hz, H_b_-2), 1.87 (1H, doublet of doublets of quartets of doublets of doublets, *J* = 8.1, 7.4, 6.7, 6.1, 5.7 Hz, H-3), 1.74–1.62 (4H, overlapping peaks, H-2′, and H-3′), 1.36 (1H, doublet of quartets of doublets, *J* = 13.2, 7.4, 5.7 Hz, H_a_-4), 1.22 (1H, pseudo doublet of quintets, *J* = 13.2, 7.4 Hz, H_b_-4), 0.92 (3H, doublet, *J* = 6.7 Hz, H-6), 0.90 (3H, triplet, *J* = 7.4 Hz, H-5); ^13^C NMR (101 MHz, CDCl_3_) 173.47 (C-1), 142.05 (C-5′), 128.37 (C-7′ and C-9′), 128.33 (C-6′ and C-10′), 125.81 (C-8′), 64.01 (C-1′), 41.54 (C-2), 35.45 (C-4′), 31.95 (C-3), 29.34 (C-4), 28.27 and 27.77 (C-2′ and C-3′), 19.28 (C-6), 11.28 (C-5); MS (EI), *m*/*z* (%) 248 (1), 132 (19), 117 (12), 105 (10), 104 (100), 99 (12), 91 (54), 71 (8), 43 (7), 41 (6).

*4-Phenylbutyl 4-methylpentanoate* (**5g**), Yield: 95%; IR (cm^−1^) 2954, 2869, 1732, 1604, 1496, 1453, 1386, 1367, 1329, 1265, 1166, 1103, 1065, 1030, 746, 698; ^1^H NMR (400 MHz, CDCl_3_) 7.31–7.25 (2H, multiplet, H-7′ and H-9′), 7.21–7.16 (3H, multiplet, H-6′, H-8′, and H-10′), 4.08 (2H, multiplet, H-1′), 2.64 (2H, multiplet, H-4′), 2.32–2.27 (2H, multiplet, H-2), 1.74–1.61 (4H, overlapping peaks, H-2′, and H-3′), 1.60–1.49 (3H, overlapping peaks, H-3 and H-4), 0.89 (6H, doublet, *J* = 6.3 Hz, H-5 and H-6); ^13^C NMR (101 MHz, CDCl_3_) 174.14 (C-1), 142.04 (C-5′), 128.37 (C-7′ and C-9′), 128.32 (C-6′ and C-10′), 125.81 (C-8′), 64.11 (C-1′), 35.44 (C-4′), 33.80 (C-3), 32.43 (C-2), 28.23 and 27.74 (C-2′ and C-3′), 27.68 (C-4), 22.23 (C-5 and C-6); MS (EI), *m*/*z* (%) 248 (1), 132 (18), 131 (8), 117 (12), 105 (10), 104 (100), 99 (7), 91 (53), 81 (8), 43 (10), 41 (8).

*4-Phenylbutyl hexanoate* (**5h**), Yield: 90%; IR (cm^−1^) 2931, 2860, 1732, 1604, 1496, 1454, 1244, 1167, 1097, 1065, 1030, 746, 698; ^1^H NMR (400 MHz, CDCl_3_) 7.31–7.25 (2H, multiplet, H-7′ and H-9′), 7.23–7.14 (3H, multiplet, H-6′, H-8′, and H-10′), 4.09 (2H, pseudo triplet, *J* = 6.5 Hz, H-1′), 2.64 (2H, pseudo triplet, *J* = 7.2 Hz, H-4′), 2.28 (2H, triplet, *J* = 7.5 Hz, H-2), 1.75–1.55 (6H, overlapping peaks, H-2′, H-3′, and H-3), 1.39–1.24 (4H, overlapping peaks, H-4 and H-5), 0.89 (3H, triplet, *J* = 7.0 Hz, H-6); ^13^C NMR (101 MHz, CDCl_3_) 173.97 (C-1), 142.05 (C-5′), 128.37 (C-7′ and C-9′), 128.33 (C-6′ and C-10′), 125.81 (C-8′), 64.08 (C-1′), 35.45 (C-4′), 34.34 (C-2), 31.33 (C-4), 28.25 and 27.76 (C-2′ and C-3′), 24.70 (C-3), 22.33 (C-5), 13.92 (C-6); MS (EI), *m*/*z* (%) 248 (1), 132 (17), 131 (9), 117 (12), 105 (10), 104 (100), 99 (9), 91 (48), 65 (6), 43 (10), 41 (6).

*5-Phenylpentyl 2-ethylbutanoate* (**6a**), Yield: 58%; IR (cm^−1^) 2931, 2855, 1730, 1604, 1495, 1454, 1240, 1155, 1020, 747, 698; ^1^H NMR (400 MHz, CDCl_3_) 7.30–7.24 (2H, multiplet, H-8′ and H-10′), 7.22–7.13 (3H, multiplet, H-7′, H-9′, and H-11′), 4.04 (2H, triplet, *J* = 6.7 Hz, H-1′), 2.62 (2H, pseudo triplet, *J* = 7.6 Hz, H-5′), 2.4571* (1H, triplet of triplets, *J* = 8.9, 5.5 Hz, H-2), 1.7812* (1H, doublet of doublets of doublets, *J* = −13.5, 8.9, 7.4 Hz, H-3b), 1.7803* (1H, doublet of doublets of doublets, *J* = −13.5, 8.9, 7.4 Hz, H-5b), 1.70–1.59 (6H, overlapping peaks, H-3a, H-5a, H-2′ and H-4′), 1.44–1.35 (2H, multiplet, H-3′), 1.0242* (6H, triplet, *J* = 7.4 Hz, H-4 and H-6); ^13^C NMR (101 MHz, CDCl_3_) 176.36 (C-1), 142.40 (C-6′), 128.37 (C-8′ and C-10′), 128.27 (C-7′ and C-11′), 125.68 (C-9′), 64.17 (C-1′), 49.02 (C-2), 35.78 (C-5′), 31.08 (C-4′), 28.54 (C-2′), 25.63 (C-3′), 25.08 (C-3 and C-5), 11.86 (C-4 and C-6); MS (EI), *m*/*z* (%) 146 (92), 131 (13), 118 (19), 117 (79), 105 (20), 104 (94), 92 (17), 91 (100), 71 (29), 43 (26). *The values of chemical shift and coupling constants were determined by a simulation of the ^1^H NMR spectrum (manual iterative full spin analysis).

*5-Phenylpentyl 2,2-dimethylbutanoate* (**6b**), Yield: 55%; IR (cm^−1^) 2970, 2929, 2856, 1728, 1451, 1388, 1360, 1346, 1311, 1240, 1150, 1018, 989, 748, 698; ^1^H NMR (400 MHz, CDCl_3_) 7.31–7.26 (2H, multiplet, H-8′ and H-10′), 7.22–7.15 (3H, multiplet, H-7′, H-9′, and H-11′), 4.05 (2H, triplet, *J* = 6.6 Hz, H-1′), 2.62 (2H, pseudo triplet, *J* =7.7 Hz, H-5′), 1.74–1.58 (4H, overlapping peaks, H-2′ and H-4′), 1.53 (2H, quartet, *J* = 7.5 Hz, H-3), 1.44–1.31 (2H, multiplet, H-3′), 1.13 (6H, singlet, H-5 and H-6), 0.83 (3H, triplet, *J* = 7.5 Hz, H-4); ^13^C NMR (101 MHz, CDCl_3_) 176.66 (C-1), 142.40 (C-6′), 128.37 (C-8′ and C-10′), 128.26 (C-7′ and C-11′), 125.68 (C-9′), 64.17 (C-1′), 42.18 (C-2), 35.78 (C-5′), 33.96 (C-3), 31.01 (C-4′), 28.52 (C-2′), 25.61 (C-3′), 24.98 (C-5 and C-6), 9.15 (C-4); MS (EI), *m*/*z* (%) 146 (93), 118 (18), 117 (76), 105 (19), 104 (94), 92 (16), 91 (100), 71 (47), 43 (34), 41 (13).

*5-Phenylpentyl 3,3-dimethylbutanoate* (**6c**), Yield: 84%; IR (cm^−1^) 2933, 2859, 1730, 1604, 1496, 1465, 1453, 1366, 1322, 1227, 1129, 1046, 1003, 746, 698; ^1^H NMR (400 MHz, CDCl_3_) 7.30–7.24 (2H, multiplet, H-8′ and H-10′), 7.22–7.13 (3H, multiplet, H-7′, H-9′, and H-11′), 4.04 (2H, triplet, *J* = 6.7 Hz, H-1′), 2.62 (2H, pseudo triplet, *J* =7.6 Hz, H-5′), 2.18 (2H, singlet, H-2), 1.70–1.59 (4H, overlapping peaks, H-2′ and H-4′), 1.44–1.35 (2H, multiplet, H-3′), 1.02 (9H, singlet, H-4–H-6); ^13^C NMR (101 MHz, CDCl_3_) 172.44 (C-1), 142.37 (C-6′), 128.36 (C-8′ and C-10′), 128.26 (C-7′ and C-11′), 125.68 (C-9′), 63.96 (C-1′), 48.05 (C-2), 35.78 (C-5′), 31.08 (C-4′), 30.68 (C-3), 29.64 (C-4–C-6), 28.54 (C-2′), 25.63 (C-3′); MS (EI), *m*/*z* (%) 146 (71), 118 (15), 117 (55), 105 (16), 104 (66), 99 (14), 92 (14), 91 (100), 57 (31), 41 (14).

*5-Phenylpentyl 2,3-dimethylbutanoate* (**6d**), Yield: 68%; IR (cm^−1^) 2929, 2855, 1731, 1604, 1496, 1451, 1389, 1359, 1345, 1298, 1258, 1189, 1152, 1075, 1045, 953, 746, 698; ^1^H NMR (400 MHz, CDCl_3_) 7.30–7.24 (2H, multiplet, H-8′ and H-10′), 7.20–7.14 (3H, multiplet, H-7′, H-9′, and H-11′), 4.12–3.99 (2H, multiplet, H-1′), 2.62 (2H, pseudo triplet, *J* =7.7 Hz, H-5′), 2.21 (1H, pseudo quintet, *J* = 7.0 Hz, H-2), 1.90 (1H, pseudo octet, *J* = 7.0 Hz, H-3), 1.70–1.60 (4H, overlapping peaks, H-2′ and H-4′), 1.44–1.35 (2H, multiplet, H-3′), 1.09 (3H, doublet, *J* = 7.0 Hz, H-6), 0.91 (3H, doublet, *J* = 7.0 Hz, H-5), 0.89 (3H, doublet, *J* = 7.0 Hz, H-4); ^13^C NMR (101 MHz, CDCl_3_) 176.50 (C-1), 142.39 (C-6′), 128.37 (C-8′ and C-10′), 128.27 (C-7′ and C-11′), 125.68 (C-9′), 64.02 (C-1′), 46.25 (C-2), 35.79 (C-5′), 31.03 (C-4′), 31.00 (C-3), 28.55 (C-2′), 25.59 (C-3′), 20.71 (C-5), 19.19 (C-4), 13.75 (C-6); MS (EI), *m*/*z* (%) 146 (86), 118 (17), 117 (69), 105 (19), 104 (80), 92 (16), 91 (100), 71 (28), 43 (23), 41 (12).

*5-Phenylpentyl 2-methylpentanoate* (**6e**), Yield: 62%; IR (cm^−1^) 2930, 2857, 1731, 1604, 1496, 1453, 1378, 1347, 1240, 1177, 1147, 1085, 1046, 955, 745, 698; ^1^H NMR (400 MHz, CDCl_3_) 7.31–7.24 (2H, multiplet, H-8′ and H-10′), 7.22–7.13 (3H, multiplet, H-7′, H-9′, and H-11′), 4.06 (2H, multiplet, H-1′), 2.62 (2H, pseudo triplet, *J* = 7.6 Hz, H-5′), 2.43 (1H, pseudo sextet, *J* = 7.0 Hz, H-2), 1.71–1.56 (5H, overlapping peaks, H_a_-3, H-2′, and H-4′), 1.44–1.26 (5H, overlapping peaks, H_b_-3, H-3′, and H-4), 1.12 (3H, doublet, *J* = 7.0 Hz, H-6), 0.90 (3H, triplet, *J* = 7.2 Hz, H-5); ^13^C NMR (101 MHz, CDCl_3_) 177.00 (C-1), 142.39 (C-6′), 128.37 (C-8′ and C-10′), 128.26 (C-7′ and C-11′), 125.68 (C-9′), 64.11 (C-1′), 39.36 (C-2), 35.97 (C-3), 35.79 (C-5′), 31.04 (C-4′), 28.53 (C-2′), 25.56 (C-3′), 20.41 (C-4), 17.08 (C-6), 13.97 (C-5); MS (EI), *m*/*z* (%) 146 (81), 118 (18), 117 (73), 105 (19), 104 (89), 92 (17), 91 (100), 71 (27), 43 (24), 41 (13).

*5-Phenylpentyl 3-methylpentanoate* (**6f**), Yield: 75%; IR (cm^−1^) 2960, 2930, 2857, 1732, 1604, 1496, 1454, 1380, 1360, 1242, 1179, 1124, 1097, 1040, 746, 698; ^1^H NMR (400 MHz, CDCl_3_) 7.30–7.24 (2H, multiplet, H-8′ and H-10′), 7.20–7.14 (3H, multiplet, H-7′, H-9′, and H-11′), 4.07 (2H, triplet, *J* = 6.7 Hz, H-1′), 2.64 (2H, pseudo triplet, *J* = 7.6 Hz, H-5′), 2.29 (1H, doublet of doublets, *J* = 14.6, 6.1 Hz, H_a_-2), 2.09 (1H, doublet of doublets, *J* = 14.6, 8.1 Hz, H_b_-2), 1.87 (1H, doublet of doublets of quartets of doublets of doublets, *J* = 8.1, 7.4, 6.7, 6.1, 5.7 Hz, H-3), 1.70–1.60 (4H, overlapping peaks, H-2′ and H-4′), 1.44–1.30 (3H, overlapping peaks, H_a_-4 and H-3′), 1.22 (1H, pseudo doublet of quintets, *J* = 13.3, 7.4 Hz, H_b_-4), 0.92 (3H, doublet, *J* = 6.7 Hz, H-6), 0.89 (3H, triplet, *J* = 7.4 Hz, H-5); ^13^C NMR (101 MHz, CDCl_3_) 173.47 (C-1), 142.40 (C-6′), 128.37 (C-8′ and C-10′), 128.27 (C-7′ and C-11′), 125.69 (C-9′), 64.14 (C-1′), 41.56 (C-2), 35.79 (C-5′), 31.95 (C-3), 31.05 (C-4′), 29.34 (C-4), 28.53 (C-2′), 25.59 (C-3′), 19.27 (C-6), 11.28 (C-5); MS (EI), *m*/*z* (%) 146 (75), 118 (17), 117 (65), 105 (18), 104 (78), 99 (15), 92 (16), 91 (100), 71 (15), 43 (14).

*5-Phenylpentyl 4-methylpentanoate* (**6g**), Yield: 80%; IR (cm^−1^) 2954, 2931, 2860, 1733, 1604, 1496, 1454, 1386, 1329, 1265, 1166, 1104, 1030, 745, 698; ^1^H NMR (400 MHz, CDCl_3_) 7.30–7.24 (2H, multiplet, H-8′ and H-10′), 7.20–7.15 (3H, multiplet, H-7′, H-9′, and H-11′), 4.05 (2H, triplet, *J* = 6.7 Hz, H-1′), 2.62 (2H, pseudo triplet, *J* =7.6 Hz, H-5′), 2.32–2.26 (2H, multiplet, H-2), 1.69–1.47 (7H, overlapping peaks, H-2′, H-4′, H-3 and H-4), 1.44–1.34 (2H, multiplet, H-3′), 0.89 (6H, doublet, *J* = 6.3 Hz, H-5 and H-6); ^13^C NMR (101 MHz, CDCl_3_) 174.14 (C-1), 142.39 (C-6′), 128.36 (C-8′ and C-10′), 128.27 (C-7′ and C-11′), 125.69 (C-9′), 64.25 (C-1′), 35.78 (C-5′), 33.80 (C-3), 32.44 (C-2), 31.06 (C-4′), 28.50 (C-2′), 27.68 (C-4), 25.57 (C-3′), 22.23 (C-5 and C-6); MS (EI), *m*/*z* (%) 146 (74), 118 (16), 117 (67), 105 (18), 104 (79), 92 (15), 91 (100), 81 (15), 43 (20), 41 (13).

*5-Phenylpentyl hexanoate* (**6h**), Yield: 82%; IR (cm^−1^) 2930, 2858, 1733, 1604, 1496, 1454, 1353, 1244, 1167, 1098, 1031, 745, 698; ^1^H NMR (400 MHz, CDCl_3_) 7.30–7.24 (2H, multiplet, H-8′ and H-10′), 7.20–7.14 (3H, multiplet, H-7′, H-9′, and H-11′), 4.05 (2H, triplet, *J* = 6.7 Hz, H-1′), 2.61 (2H, pseudo triplet, *J* = 7.6 Hz, H-5′), 2.28 (2H, triplet, *J* = 7.5 Hz, H-2), 1.70–1.57 (6H, overlapping peaks, H-2′, H-4′, and H-3), 1.44–1.26 (2H, multiplet, H-3′), 1.36–1.24 (4H, overlapping peaks, H-4 and H-5), 0.89 (3H, triplet, *J* = 6.9 Hz, H-6); ^13^C NMR (101 MHz, CDCl_3_) 173.95 (C-1), 142.40 (C-6′), 128.37 (C-8′ and C-10′), 128.27 (C-7′ and C-11′), 125.69 (C-8′), 64.21 (C-1′), 35.80 (C-5′), 34.35 (C-2), 31.33 (C-4), 31.06 (C-4′), 28.53 (C-2′), 25.59 (C-3′), 24.70 (C-3), 22.33 (C-5), 13.93 (C-6); MS (EI), *m*/*z* (%) 146 (79), 131 (12), 118 (18), 117 (73), 105 (19), 104 (87), 99 (14), 92 (17), 91 (100), 43 (18).

## 4. Conclusions

In conclusion, the creation of a synthetic library consisting of a series of esters of all constitutional isomers of hexanoic acid with phenol and a homologous series of ω-phenylalkanols (phenylmethanol, 2-phenylethan-1-ol, 3-phenylpropan-1-ol, 4-phenylbutan-1-ol, and 5-phenylpentan-1-ol), in total 48 chemical entities, enabled unambiguous identification of *Pleurospermum austriacum* essential oil constituent that was previously tentatively identified as the ester of 3-phenylpropanol and one of the isomeric hexanoic acids. The constituent of *P. austriacum* essential oil (3-phenylpropyl 2-methylpentanoate) represents a new natural product.

All synthesized esters were spectrally (NMR, MS, and IR) and gas chromatographically characterized, with RI values on commonly used capillary columns of different polarity (non-polar DB-5MS and polar HP-Innowax). A literature search showed that 24 out of the 48 synthesized esters represented new compounds (**3d**, **3e**, **4b**–**4g**, **5a**–**5h**, and **6a**–**6h**) whereas more than 10 previously known esters from the library were spectrally (MS, IR, 1D, and 2D NMR) and/or chromatographically (RI) (**1a**–**1c**, **2a**–**2c**, **3a**–**3c**, **3f**, **4a**, and **4h**) characterized for the first time. The accumulated spectral and chromatographic data, as well as the regular dependence of RI values on the presence/absence of ethyl, dimethyl, and methyl branches, provide a method for the straightforward identification of related regioisomeric compounds.

## Figures and Tables

**Table 1:** A list of synthesized esters and their retention indices on DB-5MS and HP-Innowax GC columns.

	1		2		3		4		5		6	
	i	j	i	j	i	j	i	j	i	j	i	j
**a**	1383	1856	1466	1941	1572	2052	1690	2187	1793	2295	1895	2407
**b**	1337	1784	1425	1870	1529	1981	1649	2118	1748	2224	1853	2336
**c**	1346	1818	1435	1906	1538	2013	1654	2145	1757	2258	1861	2366
**d**	1374	1855	1461	1944	1564	2051	1682	2184	1784	2295	1889	2404
**e**	1390	1866	1474	1954	1579	2081	1695	2195	1795	2305	1899	2415
**f**	1416	1921	1502	2015	1603	2115	1722	2250	1823	2364	1928	2470
**g**	1423	1936	1511	2031	1608	2126	1726	2261	1829	2375	1932	2481
**h**	1459	1985	1549	2081	1648	2179	1764	2312	1865	2427	1968	2533

i Experimental retention indices determined relative to a homologous series of *n*-alkanes (C_13_–C_20_) on a non-polar DB-5MS column; j Experimental retention indices determined relative to a homologous series of *n*-alkanes (C_17_–C_26_) on a polar HP-Innowax GC column; The precision of the RI values, on both GC columns, was confirmed by triplicate analysis with differences of <1 RI unit for all samples noted.

## Data Availability

Not applicable.

## Supplementary Materials

The following supporting information can be downloaded at: https://www.mdpi.com/article/10.3390/molecules28124574/s1, Figures S1–S153.
