# Supplementary material for: Synthesis of Small Libraries of Natural Products: Part II: Identification of a New Natural Product from the Essential Oil of Pleurospermum austriacum (L.) Hoffm. (Apiaceae)
Source: Molecules. 2023 Jun 6;28(12):4574. doi: 10.3390/molecules28124574 (PMC10305184; doi:10.3390/molecules28124574)
Supplement: Supplementary file 1 [file molecules-28-04574-s001.zip › molecules-2405467-supplementary.pdf]

Supplementary material

for

Synthesis of small libraries of natural products. Part II: identification of a new natural product from the essential oil of *Pleurospermum austriacum* (L.) Hoffm. (Apiaceae)

Niko S. Radulović [1,*], Marko Z. Mladenović [1], Milan S. Dekić [2], Fabio Boylan [3,*]

[1] Department of Chemistry, Faculty of Sciences and Mathematics, University of Niš, Višegradska 33, 18000 Niš, Serbia
[2] Department of Chemical and Technological Sciences, State University of Novi Pazar, Vuka Karadžića bb, 36300 Novi Pazar, Serbia
[3] School of Pharmacy and Pharmaceutical Sciences and Trinity Biomedical Sciences Institute, Panoz Institute, Trinity College Dublin, Dublin 2, Ireland
* Correspondence: fabio.boylan@tcd.ie; Tel.: +353-1-896-4154 and nikoradulovic@yahoo.com; Tel.: +381-18-533-015; Fax: +381-18-533-014

Content:

Figure S1. Mass spectrum of phenyl 2-ethylbutanoate (**1a**)
Figure S2a. ^{1}H NMR spectrum of phenyl 2-ethylbutanoate (**1a**) recorded in $CDCl_3$
Figure S2b. Spin simulation of the methyl groups at position 4 and 6 (lower) and comparison with the experimental ones (upper) for phenyl 2-ethylbutanoate (**1a**)
Figure S2c. Spin simulation of the proton signals for the methylene groups at position 3 and 5 (lower) and comparison with the experimental ones (upper) for phenyl 2-ethylbutanoate (**1a**)
Figure S2d. Spin simulation of the proton signal at position 2 (lower) and comparison with the experimental ones (upper) for phenyl 2-ethylbutanoate (**1a**)
Figure S3. ^{13}C NMR spectrum of phenyl 2-ethylbutanoate (**1a**) recorded in $CDCl_3$
Figure S4. Mass spectrum of phenyl 2,2-dimethylbutanoate (**1b**)
Figure S5. ^{1}H NMR spectrum of phenyl 2,2-dimethylbutanoate (**1b**) recorded in $CDCl_3$
Figure S6. ^{13}C NMR spectrum of phenyl 2,2-dimethylbutanoate (**1b**) recorded in $CDCl_3$
Figure S7. Mass spectrum of phenyl 3,3-dimethylbutanoate (**1c**)
Figure S8. ^{1}H NMR spectrum of phenyl 3,3-dimethylbutanoate (**1c**) recorded in $CDCl_3$
Figure S9. ^{13}C NMR spectrum of phenyl 3,3-dimethylbutanoate (**1c**) recorded in $CDCl_3$
Figure S10. Mass spectrum of phenyl 2,3-dimethylbutanoate (**1d**)
Figure S11. ^{1}H NMR spectrum of phenyl 2,3-dimethylbutanoate (**1d**) recorded in $CDCl_3$

Figure S12. ^{13}C NMR spectrum of phenyl 2,3-dimethylbutanoate (**1d**) recorded in $CDCl_3$
Figure S13. Mass spectrum of phenyl 2-methylpentanoate (**1e**)
Figure S14. ^{1}H NMR spectrum of phenyl 2-methylpentanoate (**1e**) recorded in $CDCl_3$
Figure S15. ^{13}C NMR spectrum of phenyl 2-methylpentanoate (**1e**) recorded in $CDCl_3$
Figure S16. Mass spectrum of phenyl 3-methylpentanoate (**1f**)
Figure S17. ^{1}H NMR spectrum of phenyl 3-methylpentanoate (**1f**) recorded in $CDCl_3$
Figure S18. ^{13}C NMR spectrum of phenyl 3-methylpentanoate (**1f**) recorded in $CDCl_3$
Figure S19. Mass spectrum of phenyl 4-methylpentanoate (**1g**)
Figure S20. ^{1}H NMR spectrum of phenyl 4-methylpentanoate (**1g**) recorded in $CDCl_3$
Figure S21. ^{13}C NMR spectrum of phenyl 4-methylpentanoate (**1g**) recorded in $CDCl_3$
Figure S22. Mass spectrum of phenyl hexanoate (**1h**)
Figure S23. ^{1}H NMR spectrum of phenyl hexanoate (**1h**) recorded in $CDCl_3$
Figure S24. ^{13}C NMR spectrum of phenyl hexanoate (**1h**) recorded in $CDCl_3$
Figure S25. Mass spectrum of benzyl 2-ethylbutanoate (**2a**)
Figure S26. ^{1}H NMR spectrum of benzyl 2-ethylbutanoate (**2a**) recorded in $CDCl_3$
Figure S27. ^{13}C NMR spectrum of benzyl 2-ethylbutanoate (**2a**) recorded in $CDCl_3$
Figure S28. Mass spectrum of benzyl 2,2-dimethylbutanoate (**2b**)
Figure S29. ^{1}H NMR spectrum of benzyl 2,2-dimethylbutanoate (**2b**) recorded in $CDCl_3$
Figure S30. ^{13}C NMR spectrum of benzyl 2,2-dimethylbutanoate (**2b**) recorded in $CDCl_3$
Figure S31. Mass spectrum of benzyl 3,3-dimethylbutanoate (**2c**)
Figure S32. ^{1}H NMR spectrum of benzyl 3,3-dimethylbutanoate (**2c**) recorded in $CDCl_3$
Figure S33. ^{13}C NMR spectrum of benzyl 3,3-dimethylbutanoate (**2c**) recorded in $CDCl_3$
Figure S34. Mass spectrum of benzyl 2,3-dimethylbutanoate (**2d**)
Figure S35. ^{1}H NMR spectrum of benzyl 2,3-dimethylbutanoate (**2d**) recorded in $CDCl_3$
Figure S36. ^{13}C NMR spectrum of benzyl 2,3-dimethylbutanoate (**2d**) recorded in $CDCl_3$
Figure S37. Mass spectrum of benzyl 2-methylpentanoate (**2e**)
Figure S38. ^{1}H NMR spectrum of benzyl 2-methylpentanoate (**2e**) recorded in $CDCl_3$
Figure S39. ^{13}C NMR spectrum of benzyl 2-methylpentanoate (**2e**) recorded in $CDCl_3$
Figure S40. Mass spectrum of benzyl 3-methylpentanoate (**2f**)
Figure S41. ^{1}H NMR spectrum of benzyl 3-methylpentanoate (**2f**) recorded in $CDCl_3$
Figure S42. ^{13}C NMR spectrum of benzyl 3-methylpentanoate (**2f**) recorded in $CDCl_3$
Figure S43. Mass spectrum of benzyl 4-methylpentanoate (**2g**)
Figure S44. ^{1}H NMR spectrum of benzyl 4-methylpentanoate (**2g**) recorded in $CDCl_3$
Figure S45. ^{13}C NMR spectrum of benzyl 4-methylpentanoate (**2g**) recorded in $CDCl_3$
Figure S46. Mass spectrum of benzyl hexanoate (**2h**)
Figure S47. ^{1}H NMR spectrum of benzyl hexanoate (**2h**) recorded in $CDCl_3$
Figure S48. ^{13}C NMR spectrum of benzyl hexanoate (**2h**) recorded in $CDCl_3$
Figure S49. Mass spectrum of phenethyl 2-ethylbutanoate (**3a**)
Figure S50. ^{1}H NMR spectrum of phenethyl 2-ethylbutanoate (**3a**) recorded in $CDCl_3$
Figure S51. ^{13}C NMR spectrum of phenethyl 2-ethylbutanoate (**3a**) recorded in $CDCl_3$
Figure S52. Mass spectrum of phenethyl 2,2-dimethylbutanoate (**3b**)
Figure S53. ^{1}H NMR spectrum of phenethyl 2,2-dimethylbutanoate (**3b**) recorded in $CDCl_3$
Figure S54. ^{13}C NMR spectrum of phenethyl 2,2-dimethylbutanoate (**3b**) recorded in $CDCl_3$

Figure S55. Mass spectrum of phenethyl 3,3-dimethylbutanoate (**3c**)
Figure S56. ^{1}H NMR spectrum of phenethyl 3,3-dimethylbutanoate (**3c**) recorded in $CDCl_3$
Figure S57. ^{13}C NMR spectrum of phenethyl 3,3-dimethylbutanoate (**3c**) recorded in $CDCl_3$
Figure S58. Mass spectrum of phenethyl 2,3-dimethylbutanoate (**3d**)
Figure S59. ^{1}H NMR spectrum of phenethyl 2,3-dimethylbutanoate (**3d**) recorded in $CDCl_3$
Figure S60. ^{13}C NMR spectrum of phenethyl 2,3-dimethylbutanoate (**3d**) recorded in $CDCl_3$
Figure S61. Mass spectrum of phenethyl 2-methylpentanoate (**3e**)
Figure S62. ^{1}H NMR spectrum of phenethyl 2-methylpentanoate (**3e**) recorded in $CDCl_3$
Figure S63. ^{13}C NMR spectrum of phenethyl 2-methylpentanoate (**3e**) recorded in $CDCl_3$
Figure S64. Mass spectrum of phenethyl 3-methylpentanoate (**3f**)
Figure S65. ^{1}H NMR spectrum of phenethyl 3-methylpentanoate (**3f**) recorded in $CDCl_3$
Figure S66. ^{13}C NMR spectrum of phenethyl 3-methylpentanoate (**3f**) recorded in $CDCl_3$
Figure S67. Mass spectrum of phenethyl 4-methylpentanoate (**3g**)
Figure S68. ^{1}H NMR spectrum of phenethyl 4-methylpentanoate (**3g**) recorded in $CDCl_3$
Figure S69. ^{13}C NMR spectrum of phenethyl 4-methylpentanoate (**3g**) recorded in $CDCl_3$
Figure S70. Mass spectrum of phenethyl hexanoate (**3h**)
Figure S71. ^{1}H NMR spectrum of phenethyl hexanoate (**3h**) recorded in $CDCl_3$
Figure S72. ^{13}C NMR spectrum of phenethyl hexanoate (**3h**) recorded in $CDCl_3$
Figure S73. Mass spectrum of 3-phenylpropyl 2-ethylbutanoate (**4a**)
Figure S74. ^{1}H NMR spectrum of 3-phenylpropyl 2-ethylbutanoate (**4a**) recorded in $CDCl_3$
Figure S75. ^{13}C NMR spectrum of 3-phenylpropyl 2-ethylbutanoate (**4a**) recorded in $CDCl_3$
Figure S76. Mass spectrum of 3-phenylpropyl 2,2-dimethylbutanoate (**4b**)
Figure S77. ^{1}H NMR spectrum of 3-phenylpropyl 2,2-dimethylbutanoate (**4b**) recorded in $CDCl_3$
Figure S78. ^{13}C NMR spectrum of 3-phenylpropyl 2,2-dimethylbutanoate (**4b**) recorded in $CDCl_3$
Figure S79. Mass spectrum of 3-phenylpropyl 3,3-dimethylbutanoate (**4c**)
Figure S80. ^{1}H NMR spectrum of 3-phenylpropyl 3,3-dimethylbutanoate (**4c**) recorded in $CDCl_3$
Figure S81. ^{13}C NMR spectrum of 3-phenylpropyl 3,3-dimethylbutanoate (**4c**) recorded in $CDCl_3$
Figure S82. Mass spectrum of 3-phenylpropyl 2,3-dimethylbutanoate (**4d**)
Figure S83. ^{1}H NMR spectrum of 3-phenylpropyl 2,3-dimethylbutanoate (**4d**) recorded in $CDCl_3$
Figure S84. ^{13}C NMR spectrum of 3-phenylpropyl 2,3-dimethylbutanoate (**4d**) recorded in $CDCl_3$
Figure S85. Mass spectrum of 3-phenylpropyl 2-methylpentanoate (**4e**)
Figure S86a. ^{1}H NMR spectrum of 3-phenylpropyl 2-methylpentanoate (**4e**) recorded in $CDCl_3$
Figure S86b. ^{1}H – ^{1}H COSY spectrum of 3-phenylpropyl 2-methylpentanoate (**4e**)
Figure S86c. NOESY spectrum of 3-phenylpropyl 2-methylpentanoate (**4e**)
Figure S87a. ^{13}C NMR spectrum of 3-phenylpropyl 2-methylpentanoate (**4e**) recorded in $CDCl_3$
Figure S87b. DEPT 135 spectrum of 3-phenylpropyl 2-methylpentanoate (**4e**)
Figure S87c. Proton-coupled ^{13}C NMR spectrum of 3-phenylpropyl 2-methylpentanoate (**4e**)
Figure S87d. grHSQC spectrum of 3-phenylpropyl 2-methylpentanoate (**4e**)
Figure S87e. grHMBC spectrum of 3-phenylpropyl 2-methylpentanoate (**4e**)
Figure S88. Mass spectrum of 3-phenylpropyl 3-methylpentanoate (**4f**)
Figure S89. ^{1}H NMR spectrum of 3-phenylpropyl 3-methylpentanoate (**4f**) recorded in $CDCl_3$

Figure S90. ^{13}C NMR spectrum of 3-phenylpropyl 3-methylpentanoate (**4f**) recorded in $CDCl_3$
Figure S91. Mass spectrum of 3-phenylpropyl 4-methylpentanoate (**4g**)
Figure S92. ^{1}H NMR spectrum of 3-phenylpropyl 4-methylpentanoate (**4g**) recorded in $CDCl_3$
Figure S93. ^{13}C NMR spectrum of 3-phenylpropyl 4-methylpentanoate (**4g**) recorded in $CDCl_3$
Figure S94. Mass spectrum of 3-phenylpropyl hexanoate (**4h**)
Figure S95. ^{1}H NMR spectrum of 3-phenylpropyl hexanoate (**4h**) recorded in $CDCl_3$
Figure S96. ^{13}C NMR spectrum of 3-phenylpropyl hexanoate (**4h**) recorded in $CDCl_3$
Figure S97. Mass spectrum of 4-phenylbutyl 2-ethylbutanoate (**5a**)
Figure S98. ^{1}H NMR spectrum of 4-phenylbutyl 2-ethylbutanoate (**5a**) recorded in $CDCl_3$
Figure S99. ^{13}C NMR spectrum of 4-phenylbutyl 2-ethylbutanoate (**5a**) recorded in $CDCl_3$
Figure S100. Mass spectrum of 4-phenylbutyl 2,2-dimethylbutanoate (**5b**)
Figure S101. ^{1}H NMR spectrum of 4-phenylbutyl 2,2-dimethylbutanoate (**5b**) recorded in $CDCl_3$
Figure S102. ^{13}C NMR spectrum of 4-phenylbutyl 2,2-dimethylbutanoate (**5b**) recorded in $CDCl_3$
Figure S103. Mass spectrum of 4-phenylbutyl 3,3-dimethylbutanoate (**5c**)
Figure S104. ^{1}H NMR spectrum of 4-phenylbutyl 3,3-dimethylbutanoate (**5c**) recorded in $CDCl_3$
Figure S105. ^{13}C NMR spectrum of 4-phenylbutyl 3,3-dimethylbutanoate (**5c**) recorded in $CDCl_3$
Figure S106. Mass spectrum of 4-phenylbutyl 2,3-dimethylbutanoate (**5d**)
Figure S107. ^{1}H NMR spectrum of 4-phenylbutyl 2,3-dimethylbutanoate (**5d**) recorded in $CDCl_3$
Figure S108. ^{13}C NMR spectrum of 4-phenylbutyl 2,3-dimethylbutanoate (**5d**) recorded in $CDCl_3$
Figure S109. Mass spectrum of 4-phenylbutyl 2-methylpentanoate (**5e**)
Figure S110. ^{1}H NMR spectrum of 4-phenylbutyl 2-methylpentanoate (**5e**) recorded in $CDCl_3$
Figure S111. ^{13}C NMR spectrum of 4-phenylbutyl 2-methylpentanoate (**5e**) recorded in $CDCl_3$
Figure S112. Mass spectrum of 4-phenylbutyl 3-methylpentanoate (**5f**)
Figure S113. ^{1}H NMR spectrum of 4-phenylbutyl 3-methylpentanoate (**5f**) recorded in $CDCl_3$
Figure S114. ^{13}C NMR spectrum of 4-phenylbutyl 3-methylpentanoate (**5f**) recorded in $CDCl_3$
Figure S115. Mass spectrum of 4-phenylbutyl 4-methylpentanoate (**5g**)
Figure S116. ^{1}H NMR spectrum of 4-phenylbutyl 4-methylpentanoate (**5g**) recorded in $CDCl_3$
Figure S117. ^{13}C NMR spectrum of 4-phenylbutyl 4-methylpentanoate (**5g**) recorded in $CDCl_3$
Figure S118. Mass spectrum of 4-phenylbutyl hexanoate (**5h**)
Figure S119. ^{1}H NMR spectrum of 4-phenylbutyl hexanoate (**5h**) recorded in $CDCl_3$
Figure S120. ^{13}C NMR spectrum of 4-phenylbutyl hexanoate (**5h**) recorded in $CDCl_3$
Figure S121. Mass spectrum of 5-phenylpentyl 2-ethylbutanoate (**6a**)
Figure S122. ^{1}H NMR spectrum of 5-phenylpentyl 2-ethylbutanoate (**6a**) recorded in $CDCl_3$
Figure S123. ^{13}C NMR spectrum of 5-phenylpentyl 2-ethylbutanoate (**6a**) recorded in $CDCl_3$
Figure S124. Mass spectrum of 5-phenylpentyl 2,2-dimethylbutanoate (**6b**)
Figure S125. ^{1}H NMR spectrum of 5-phenylpentyl 2,2-dimethylbutanoate (**6b**) recorded in $CDCl_3$
Figure S126. ^{13}C NMR spectrum of 5-phenylpentyl 2,2-dimethylbutanoate (**6b**) recorded in $CDCl_3$
Figure S127. Mass spectrum of 5-phenylpentyl 3,3-dimethylbutanoate (**6c**)
Figure S128. ^{1}H NMR spectrum of 5-phenylpentyl 3,3-dimethylbutanoate (**6c**) recorded in $CDCl_3$

Figure S129. ^{13}C NMR spectrum of 5-phenylpentyl 3,3-dimethylbutanoate (**6c**) recorded in $CDCl_3$
Figure S130. Mass spectrum of 5-phenylpentyl 2,3-dimethylbutanoate (**6d**)
Figure S131. ^{1}H NMR spectrum of 5-phenylpentyl 2,3-dimethylbutanoate (**6d**) recorded in $CDCl_3$
Figure S132. ^{13}C NMR spectrum of 5-phenylpentyl 2,3-dimethylbutanoate (**6d**) recorded in $CDCl_3$
Figure S133. Mass spectrum of 5-phenylpentyl 2-methylpentanoate (**6e**)
Figure S134. ^{1}H NMR spectrum of 5-phenylpentyl 2-methylpentanoate (**6e**) recorded in $CDCl_3$
Figure S135. ^{13}C NMR spectrum of 5-phenylpentyl 2-methylpentanoate (**6e**) recorded in $CDCl_3$
Figure S136. Mass spectrum of 5-phenylpentyl 3-methylpentanoate (**6f**)
Figure S137. ^{1}H NMR spectrum of 5-phenylpentyl 3-methylpentanoate (**6f**) recorded in $CDCl_3$
Figure S138. ^{13}C NMR spectrum of 5-phenylpentyl 3-methylpentanoate (**6f**) recorded in $CDCl_3$
Figure S139. Mass spectrum of 5-phenylpentyl 4-methylpentanoate (**6g**)
Figure S140. ^{1}H NMR spectrum of 5-phenylpentyl 4-methylpentanoate (**6g**) recorded in $CDCl_3$
Figure S141. ^{13}C NMR spectrum of 5-phenylpentyl 4-methylpentanoate (**6g**) recorded in $CDCl_3$
Figure S142. Mass spectrum of 5-phenylpentyl hexanoate (**6h**)
Figure S143. ^{1}H NMR spectrum of 5-phenylpentyl hexanoate (**6h**) recorded in $CDCl_3$
Figure S144. ^{13}C NMR spectrum of 5-phenylpentyl hexanoate (**6h**) recorded in $CDCl_3$
Figure S145. Typical total ion current (TIC) chromatogram of a mixture of synthesized (**A**) 3-methylpentanoates and (**B**) 4-methylpentanoates
Figure S146. The dependence of RI values of the esters of the same acid (2-ethylbutanoic acid) and phenol (**1**), benzyl alcohol (**2**), phenethyl alcohol (**3**), 3-phenylpropanol (**4**), 4-phenylbutanol (**5**), and 5-phenylpentanol (**6**)
Figure S147. The dependence of RI values of the esters of the same acid (2,2-dimethylbutanoic acid) and phenol (**1**), benzyl alcohol (**2**), phenethyl alcohol (**3**), 3-phenylpropanol (**4**), 4-phenylbutanol (**5**), and 5-phenylpentanol (**6**)
Figure S148. The dependence of RI values of the esters of the same acid (3,3-dimethylbutanoic acid) and phenol (**1**), benzyl alcohol (**2**), phenethyl alcohol (**3**), 3-phenylpropanol (**4**), 4-phenylbutanol (**5**), and 5-phenylpentanol (**6**)
Figure S149. The dependence of RI values of the esters of the same acid (2,3-dimethylbutanoic acid) and phenol (**1**), benzyl alcohol (**2**), phenethyl alcohol (**3**), 3-phenylpropanol (**4**), 4-phenylbutanol (**5**), and 5-phenylpentanol (**6**)
Figure S150. The dependence of RI values of the esters of the same acid (2-methylpentanoic acid) and phenol (**1**), benzyl alcohol (**2**), phenethyl alcohol (**3**), 3-phenylpropanol (**4**), 4-phenylbutanol (**5**), and 5-phenylpentanol (**6**)
Figure S151. The dependence of RI values of the esters of the same acid (3-methylpentanoic acid) and phenol (**1**), benzyl alcohol (**2**), phenethyl alcohol (**3**), 3-phenylpropanol (**4**), 4-phenylbutanol (**5**), and 5-phenylpentanol (**6**)
Figure S152. The dependence of RI values of the esters of the same acid (4-methylpentanoic acid) and phenol (**1**), benzyl alcohol (**2**), phenethyl alcohol (**3**), 3-phenylpropanol (**4**), 4-phenylbutanol (**5**), and 5-phenylpentanol (**6**)

Figure S153. The dependence of RI values of the esters of the same acid (hexanoic acid) and phenol (**1**), benzyl alcohol (**2**), phenethyl alcohol (**3**), 3-phenylpropanol (**4**), 4-phenylbutanol (**5**), and 5-phenylpentanol (**6**)

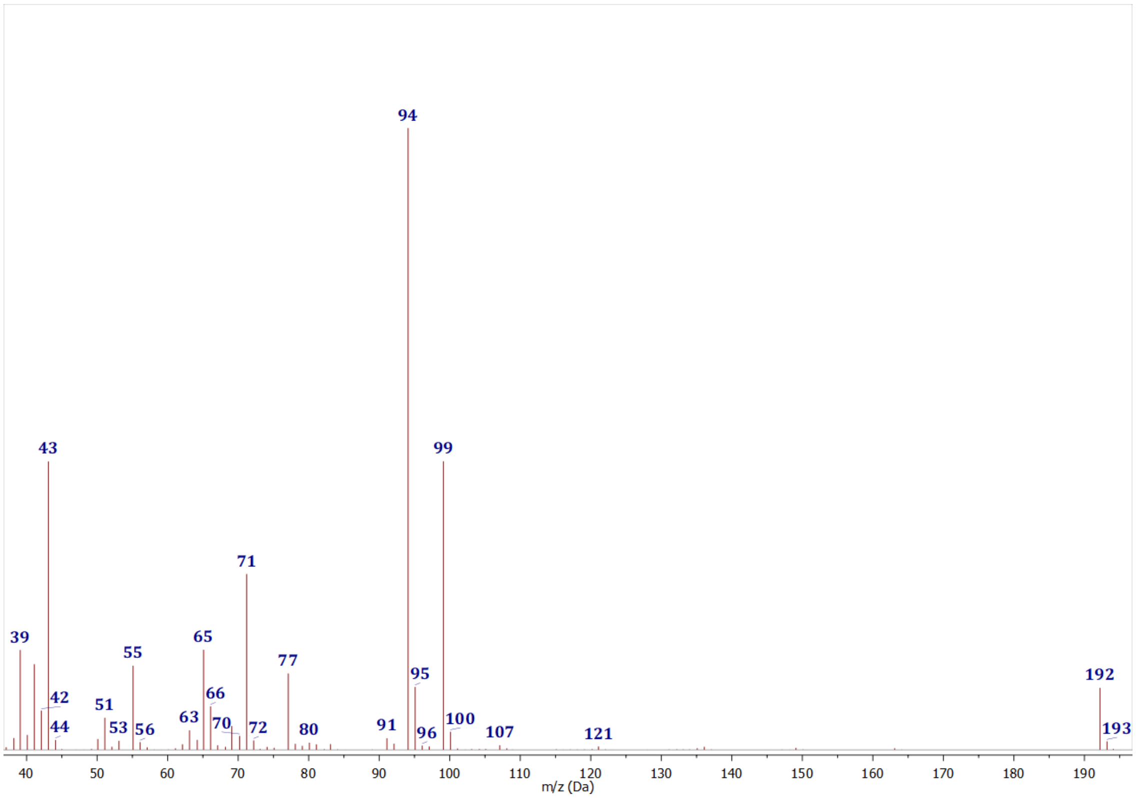

Figure S1. Mass spectrum of phenyl 2-ethylbutanoate (**1a**)

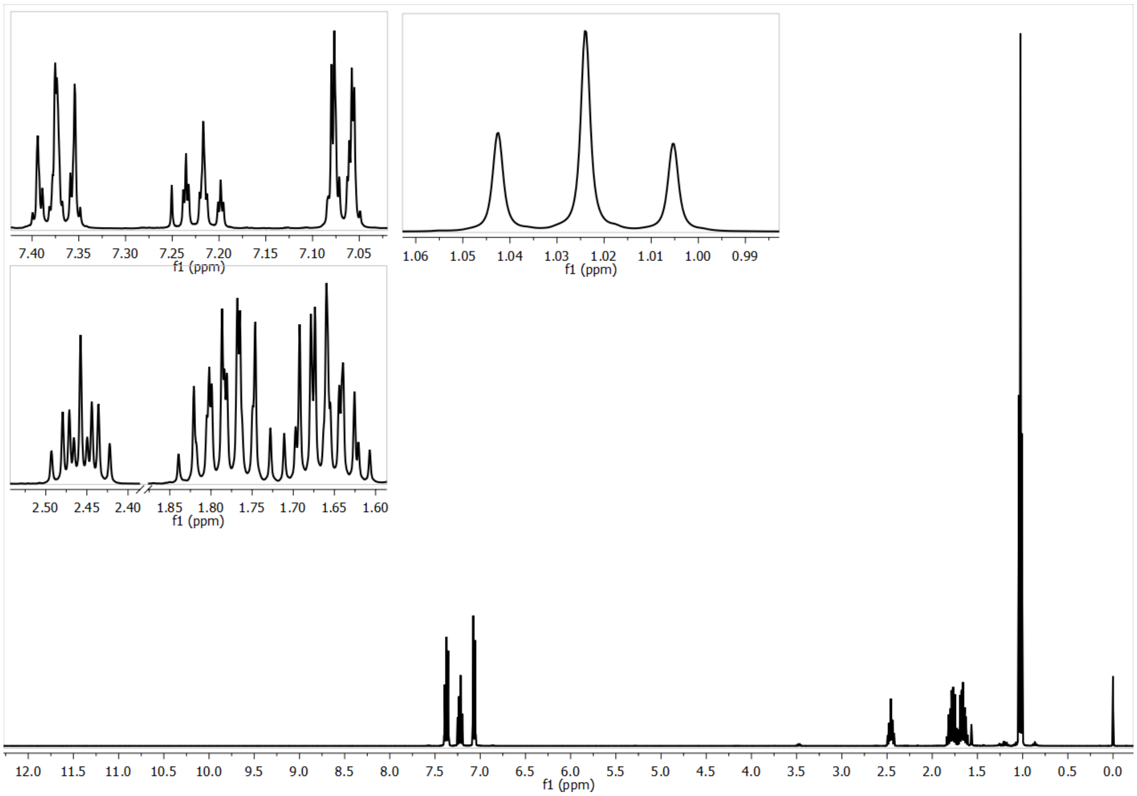

Figure S2a. ^{1}H NMR spectrum of phenyl 2-ethylbutanoate (**1a**) recorded in $CDCl_3$

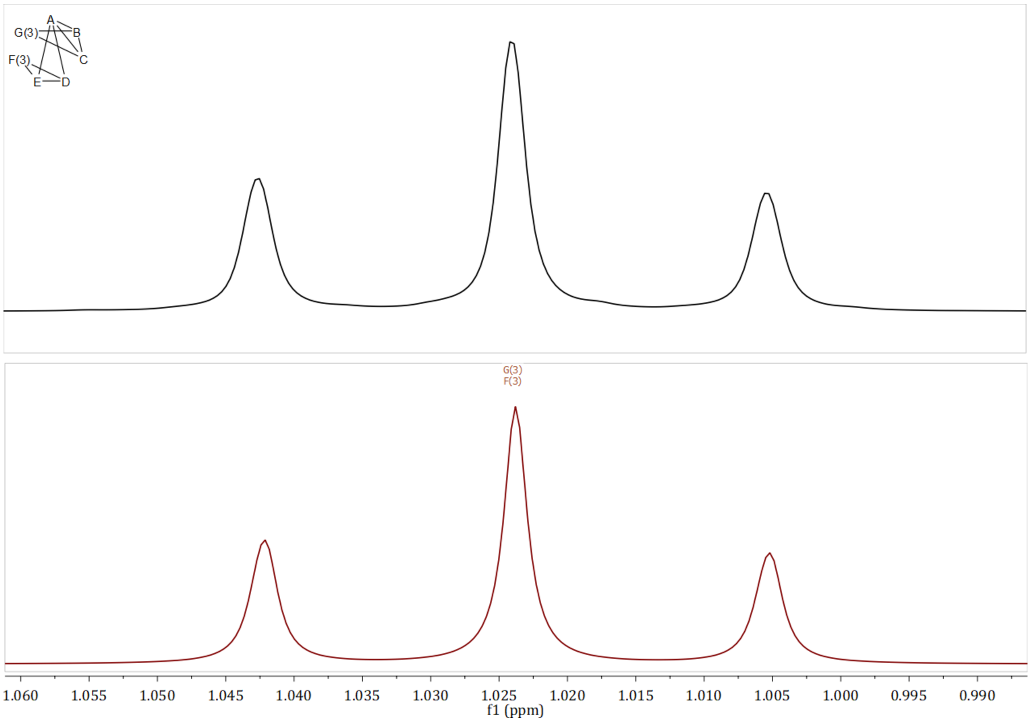

Figure S2b. Spin simulation of the methyl groups at position 4 and 6 (lower) and comparison with the experimental ones (upper) for phenyl 2-ethylbutanoate (**1a**)

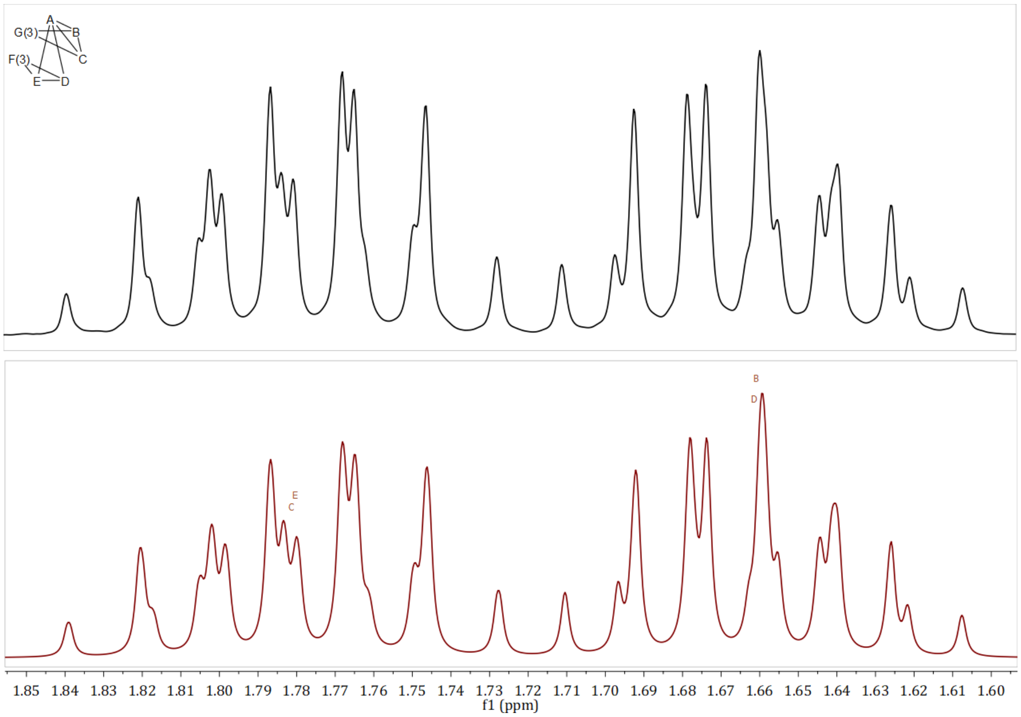

Figure S2c. Spin simulation of the proton signals for the methylene groups at position 3 and 5 (lower) and comparison with the experimental ones (upper) for phenyl 2-ethylbutanoate (**1a**)

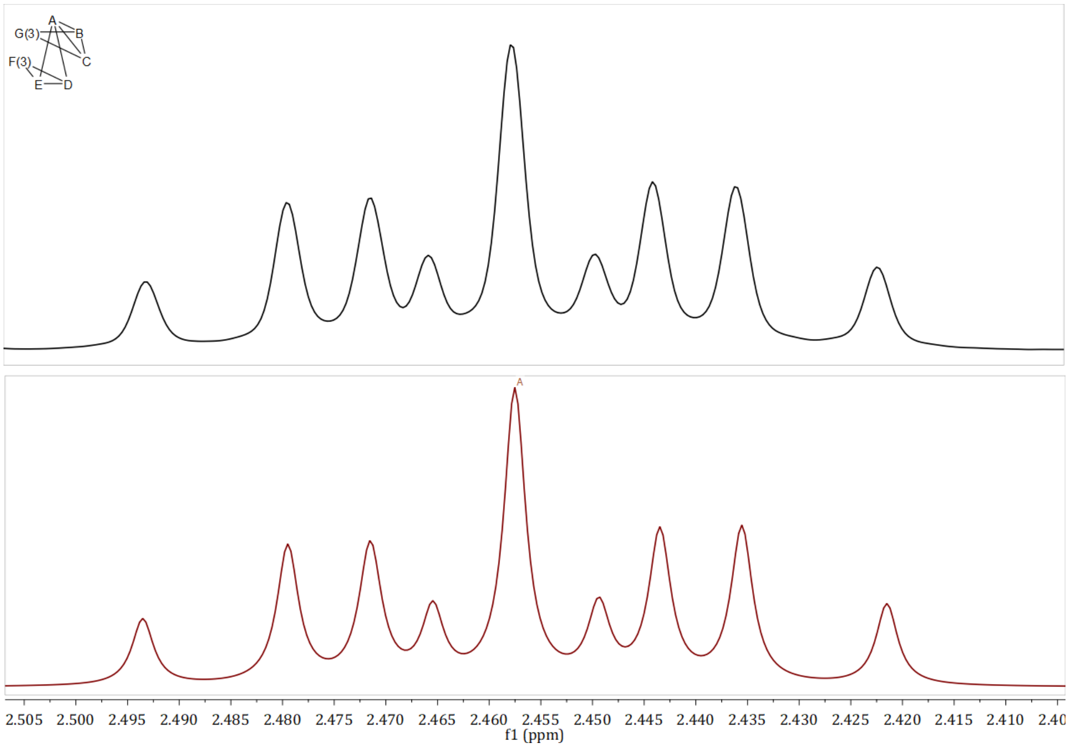

Figure S2d. Spin simulation of the proton signal at position 2 (lower) and comparison with the experimental ones (upper) for phenyl 2-ethylbutanoate (**1a**)

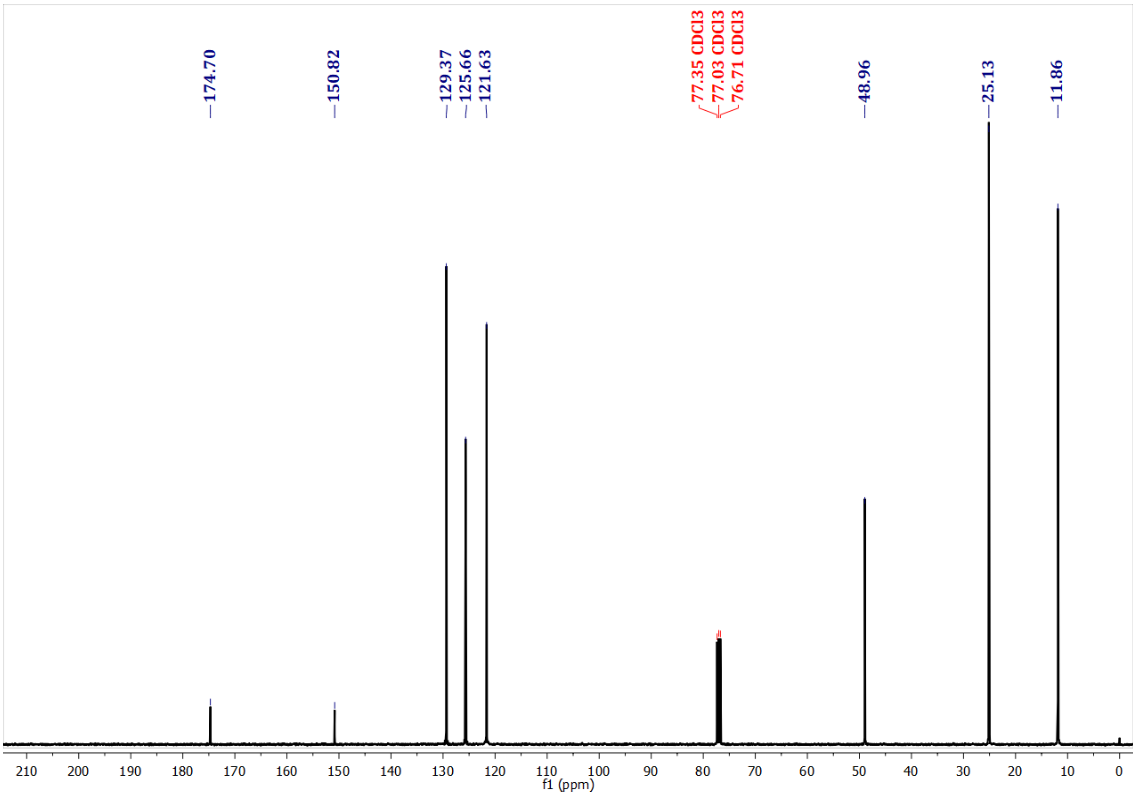

Figure S3. ^{13}C NMR spectrum of phenyl 2-ethylbutanoate (**1a**) recorded in $CDCl_3$

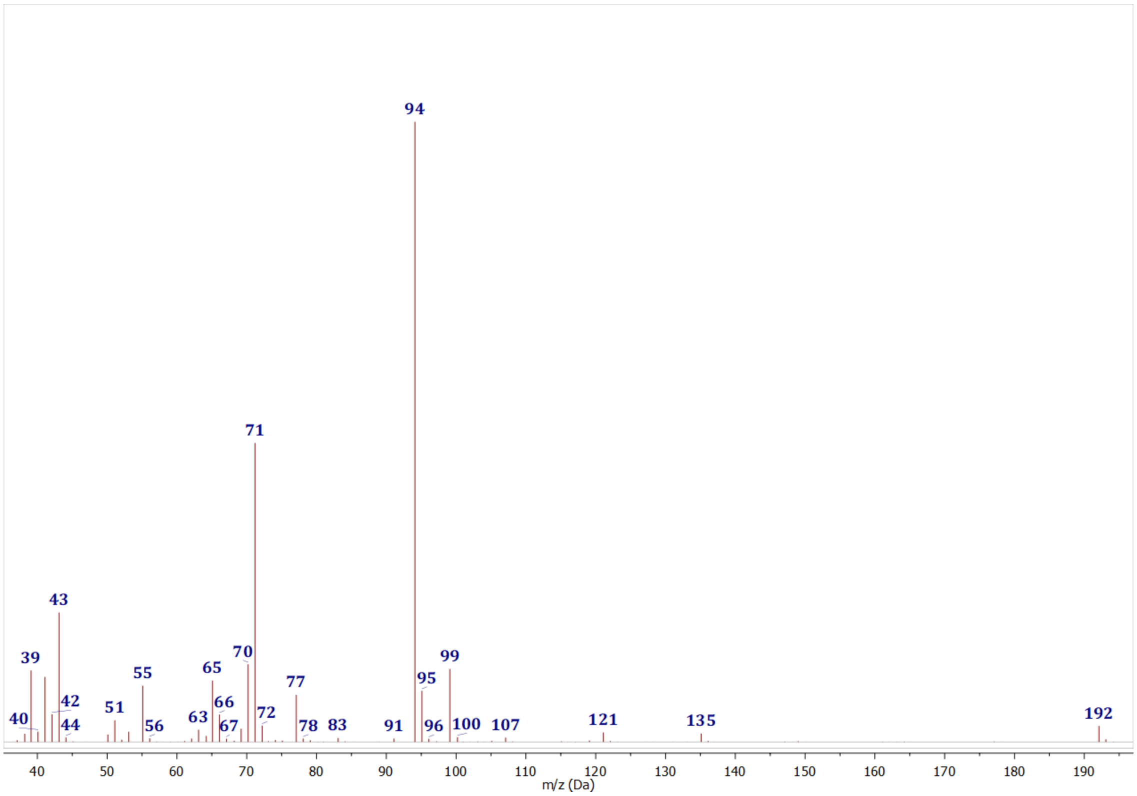

Figure S4. Mass spectrum of phenyl 2,2-dimethylbutanoate (**1b**)

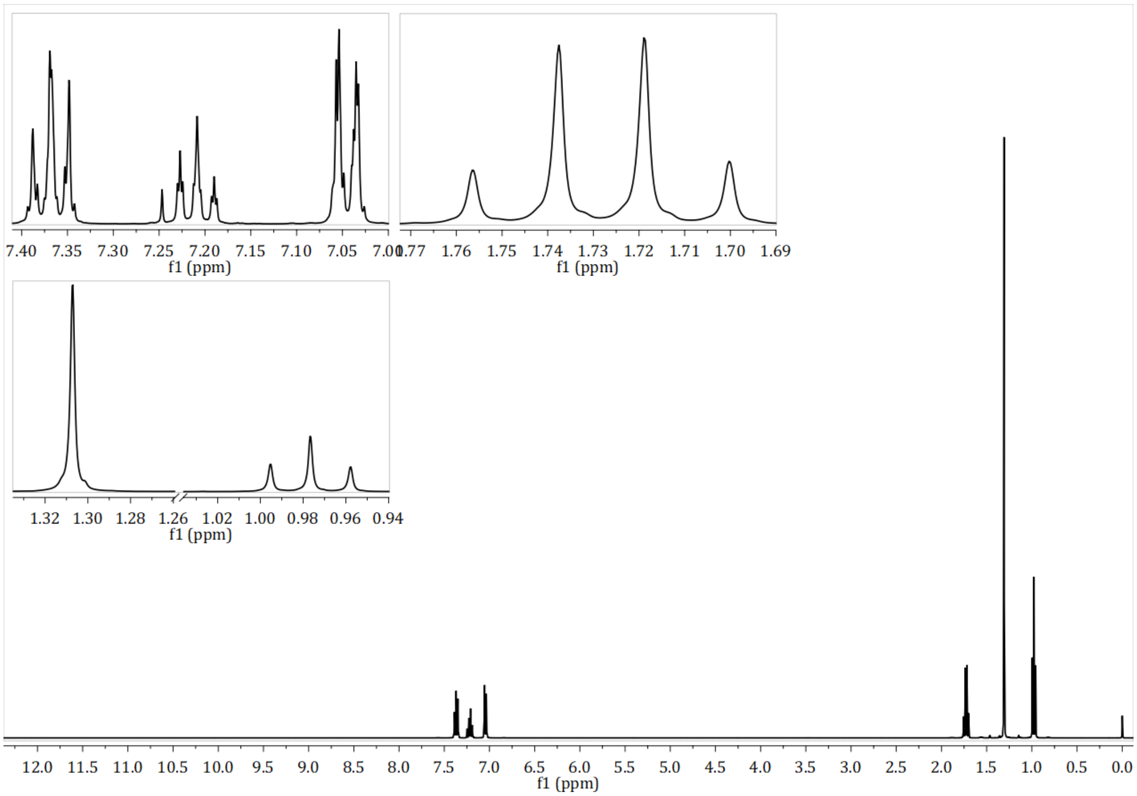

Figure S5. ^{1}H NMR spectrum of phenyl 2,2-dimethylbutanoate (**1b**) recorded in $CDCl_3$

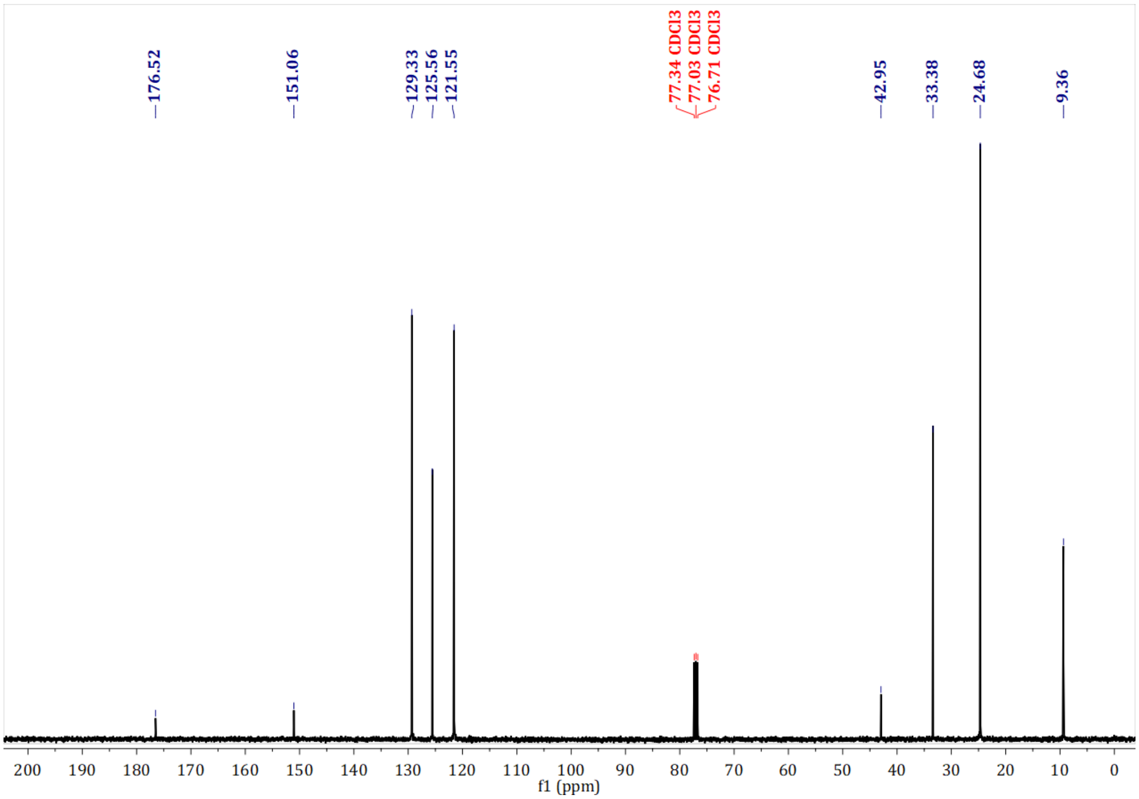

Figure S6. ^{13}C NMR spectrum of phenyl 2,2-dimethylbutanoate (**1b**) recorded in $CDCl_3$

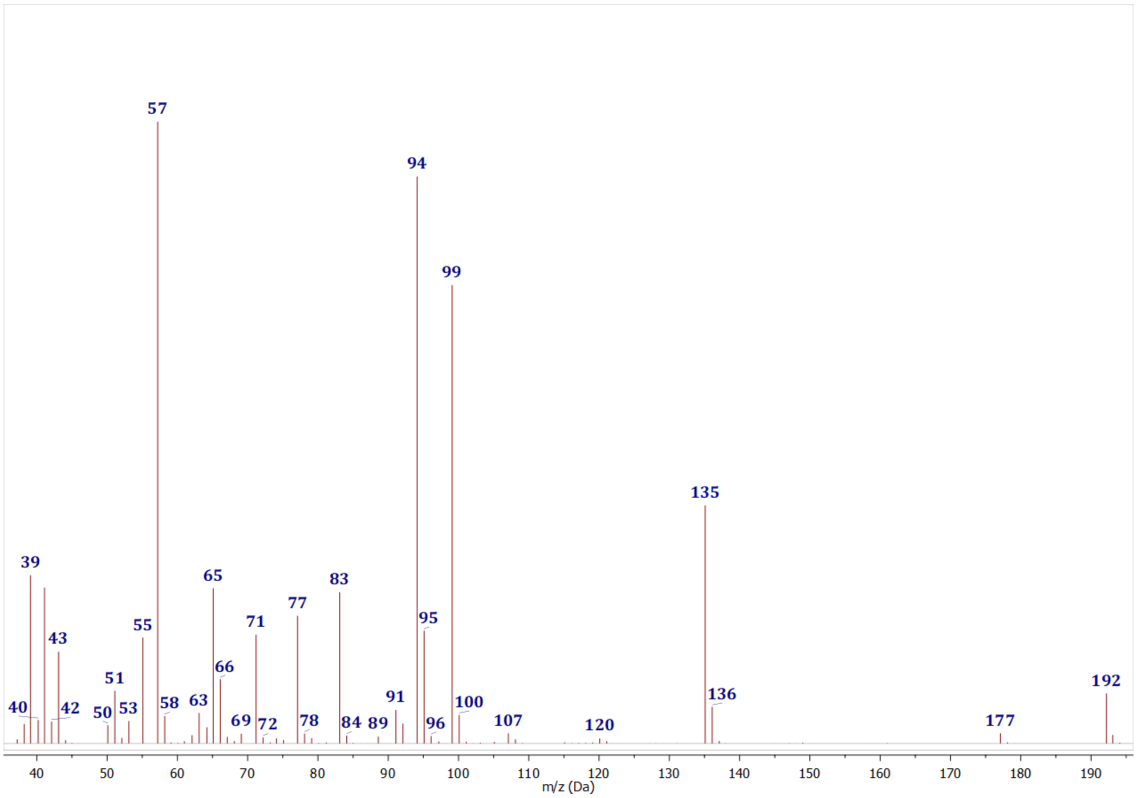

Figure S7. Mass spectrum of phenyl 3,3-dimethylbutanoate (**1c**)

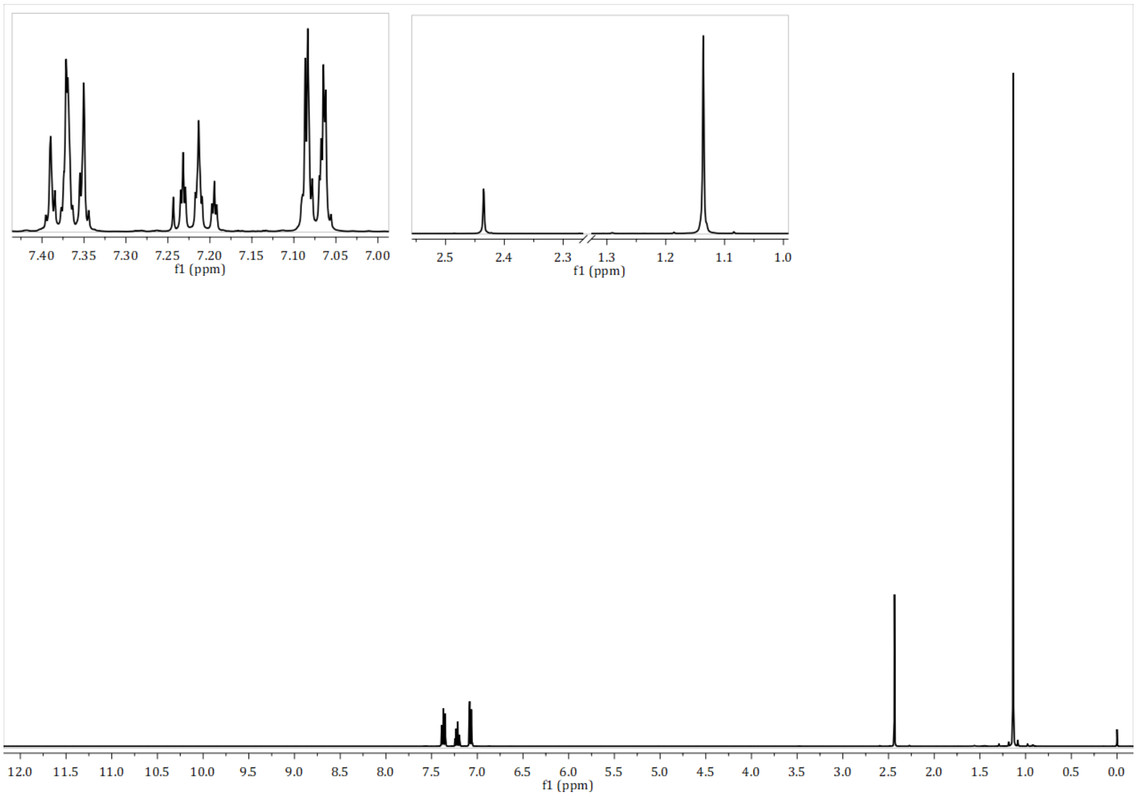

Figure S8. ^{1}H NMR spectrum of phenyl 3,3-dimethylbutanoate (**1c**) recorded in $CDCl_3$

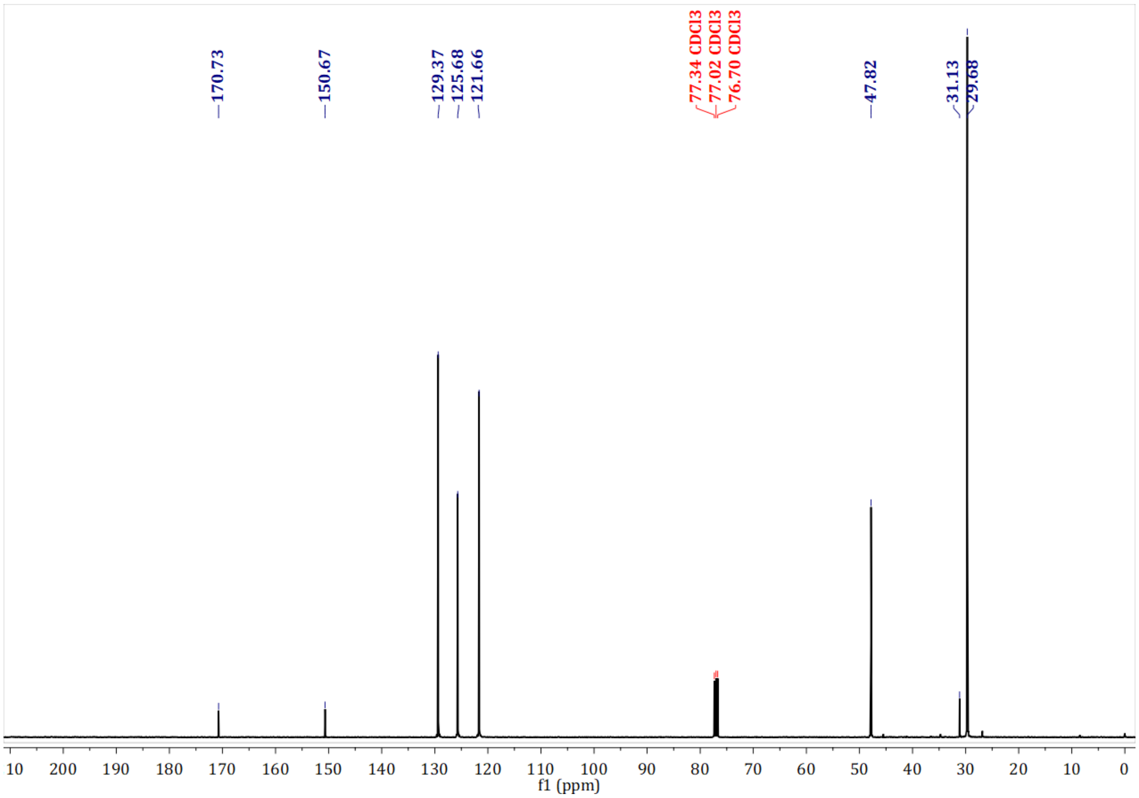

Figure S9. ^{13}C NMR spectrum of phenyl 3,3-dimethylbutanoate (**1c**) recorded in $CDCl_3$

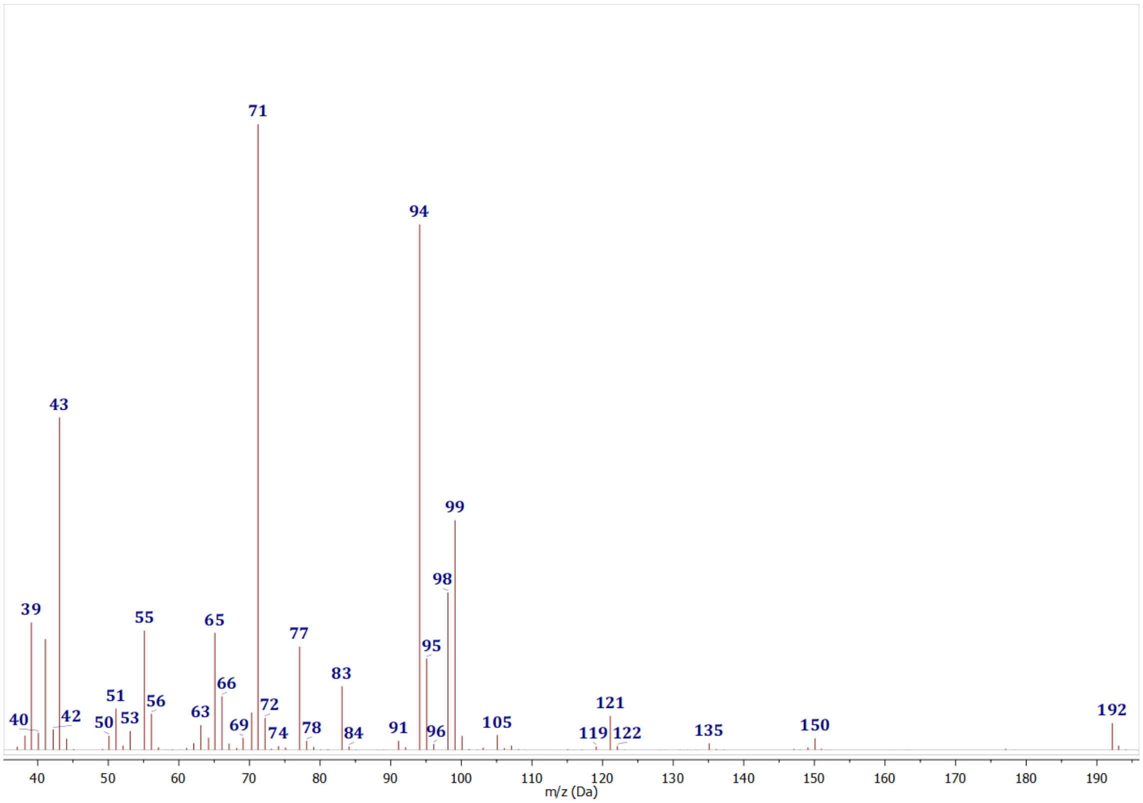

Figure S10. Mass spectrum of phenyl 2,3-dimethylbutanoate (**1d**)

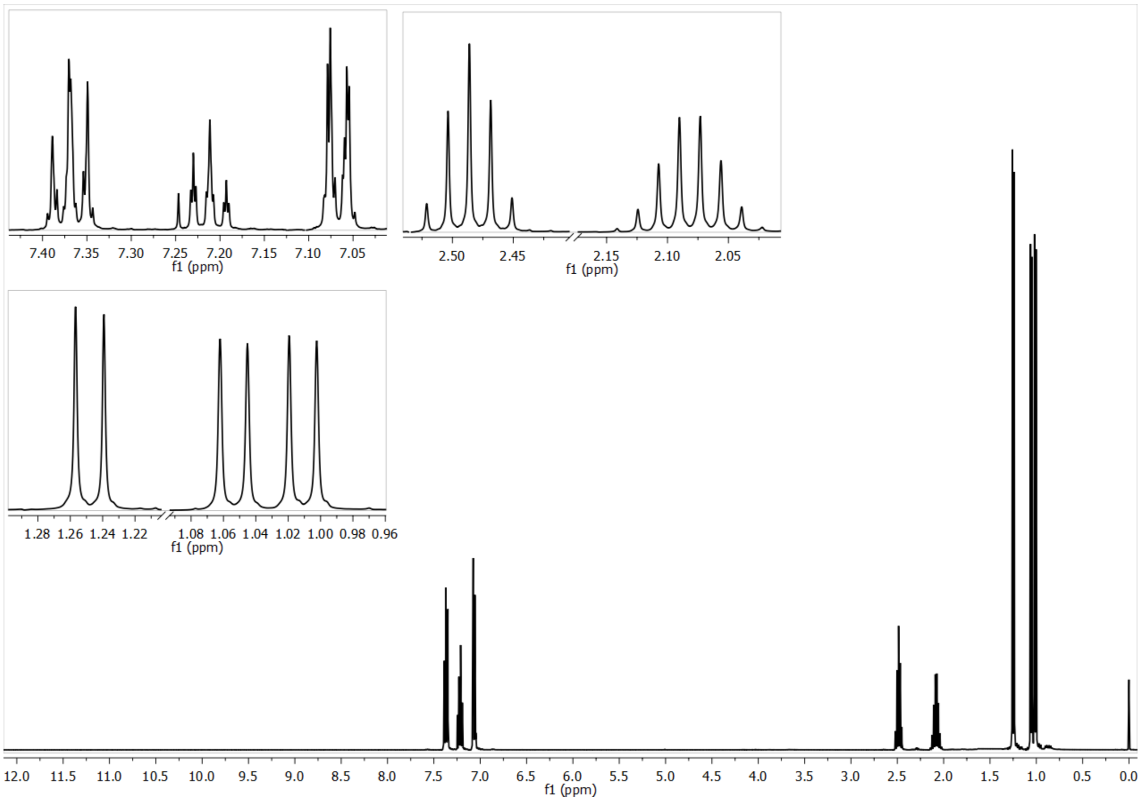

Figure S11. ^{1}H NMR spectrum of phenyl 2,3-dimethylbutanoate (**1d**) recorded in $CDCl_3$

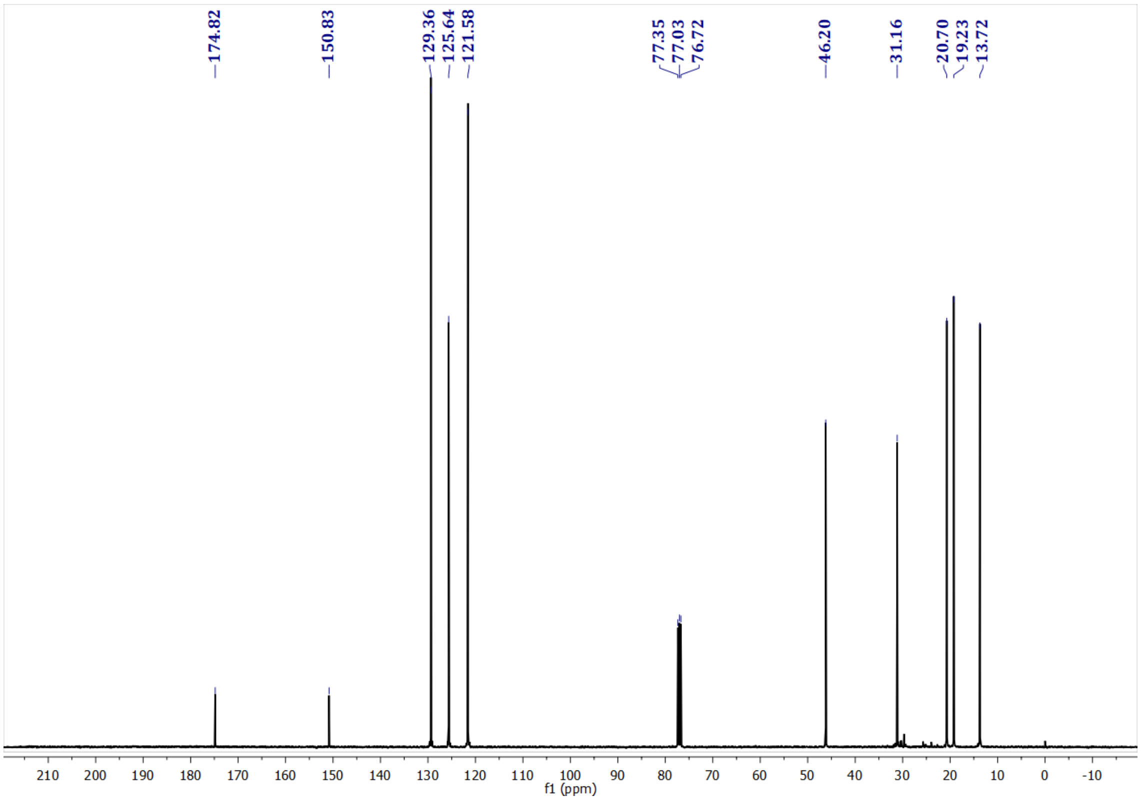

Figure S12. ^{13}C NMR spectrum of phenyl 2,3-dimethylbutanoate (**1d**) recorded in $CDCl_3$

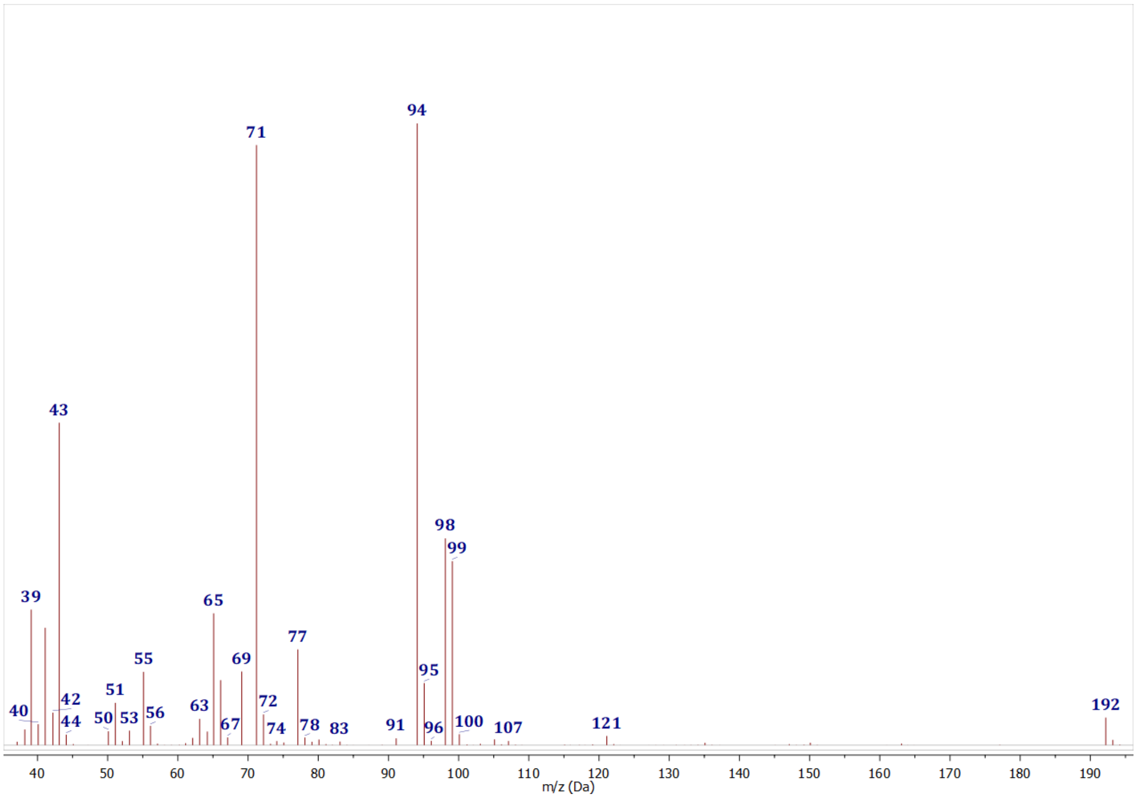

Figure S13. Mass spectrum of phenyl 2-methylpentanoate (**1e**)

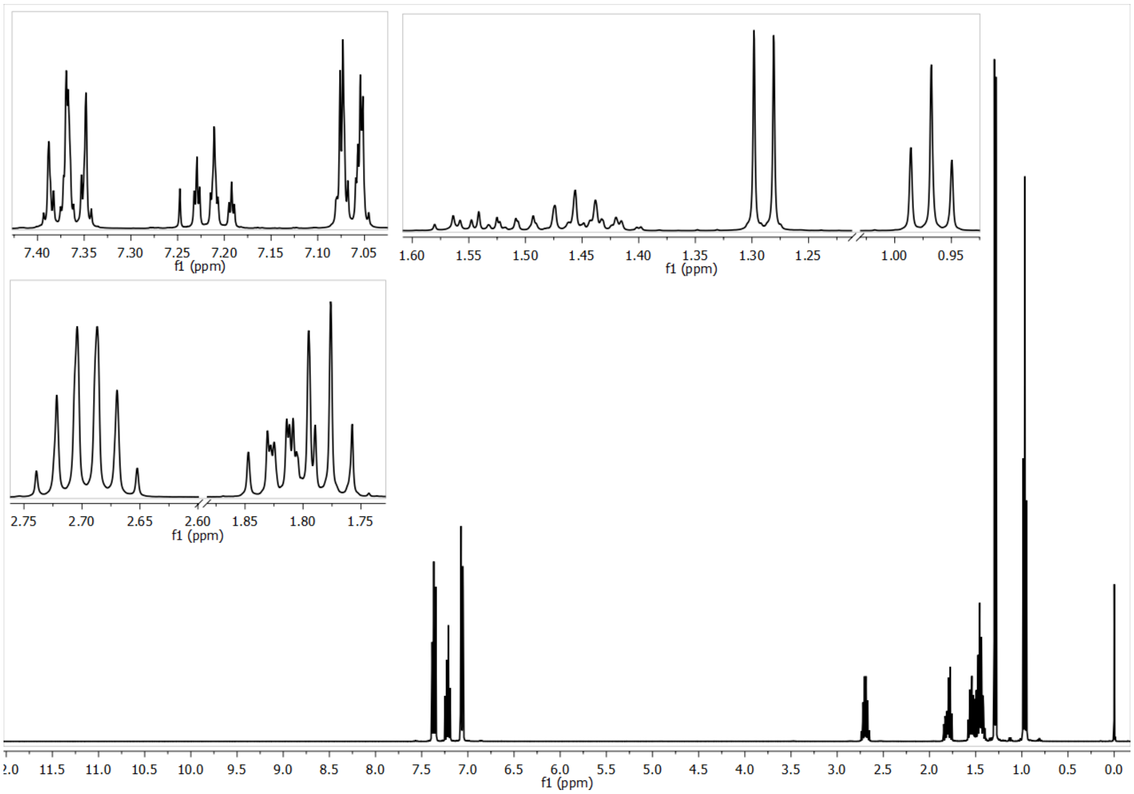

Figure S14. ^{1}H NMR spectrum of phenyl 2-methylpentanoate (**1e**) recorded in $CDCl_3$

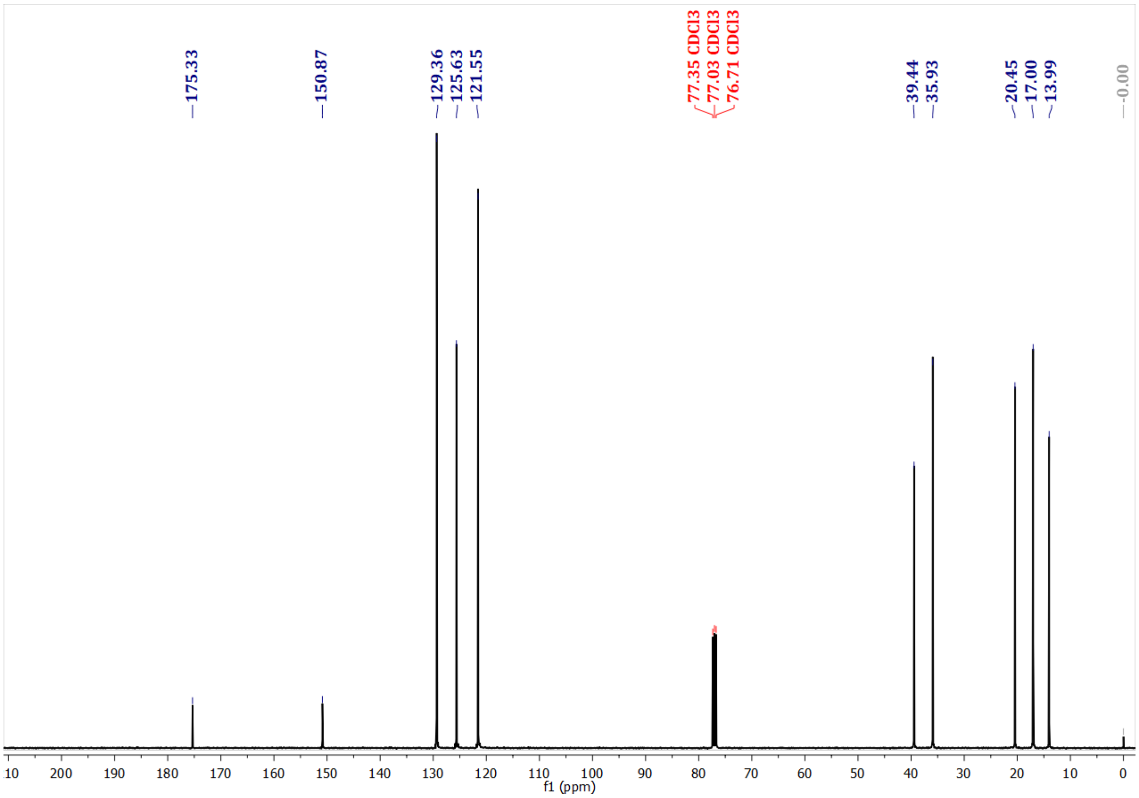

Figure S15. ^{13}C NMR spectrum of phenyl 2-methylpentanoate (**1e**) recorded in $CDCl_3$

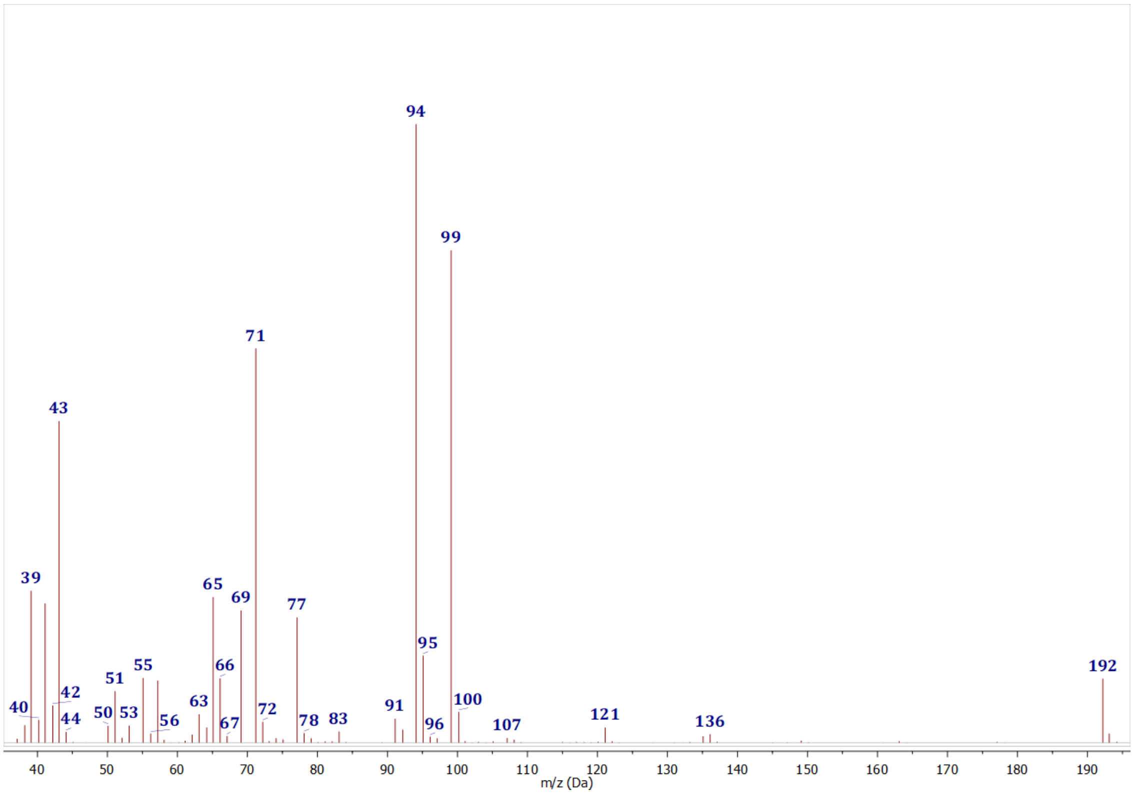

Figure S16. Mass spectrum of phenyl 3-methylpentanoate (**1f**)

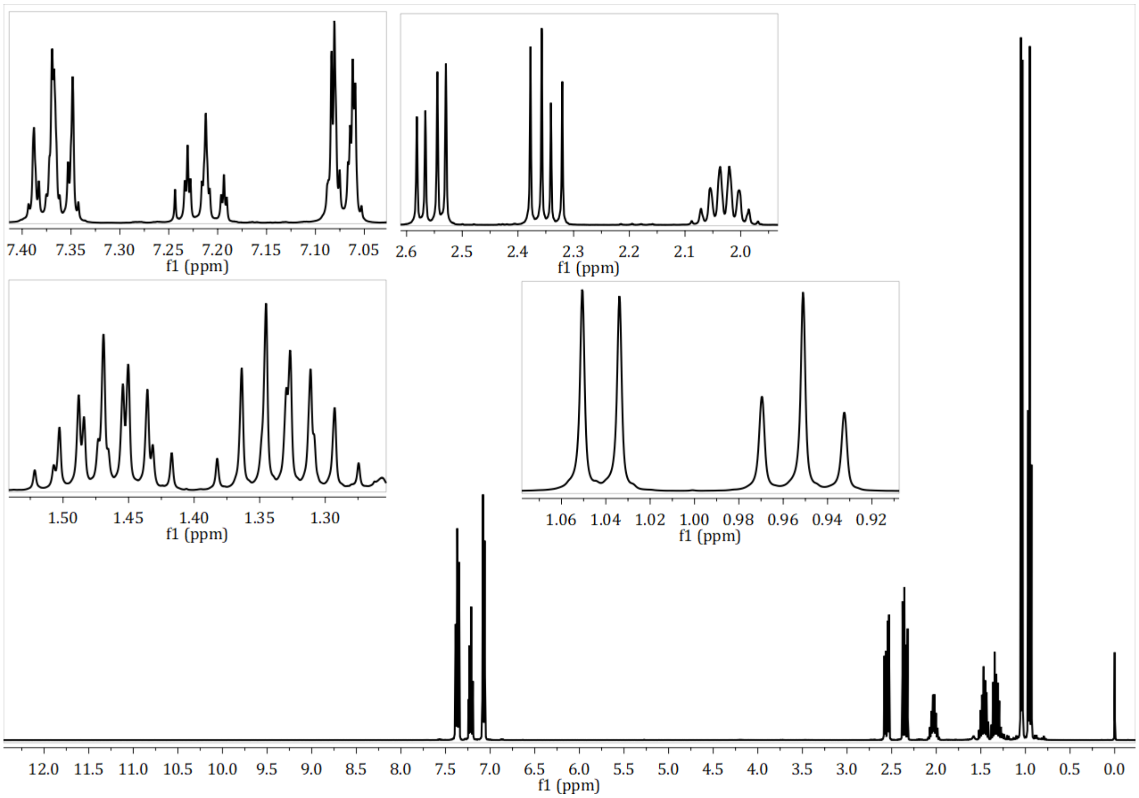

Figure S17. ^{1}H NMR spectrum of phenyl 3-methylpentanoate (**1f**) recorded in $CDCl_3$

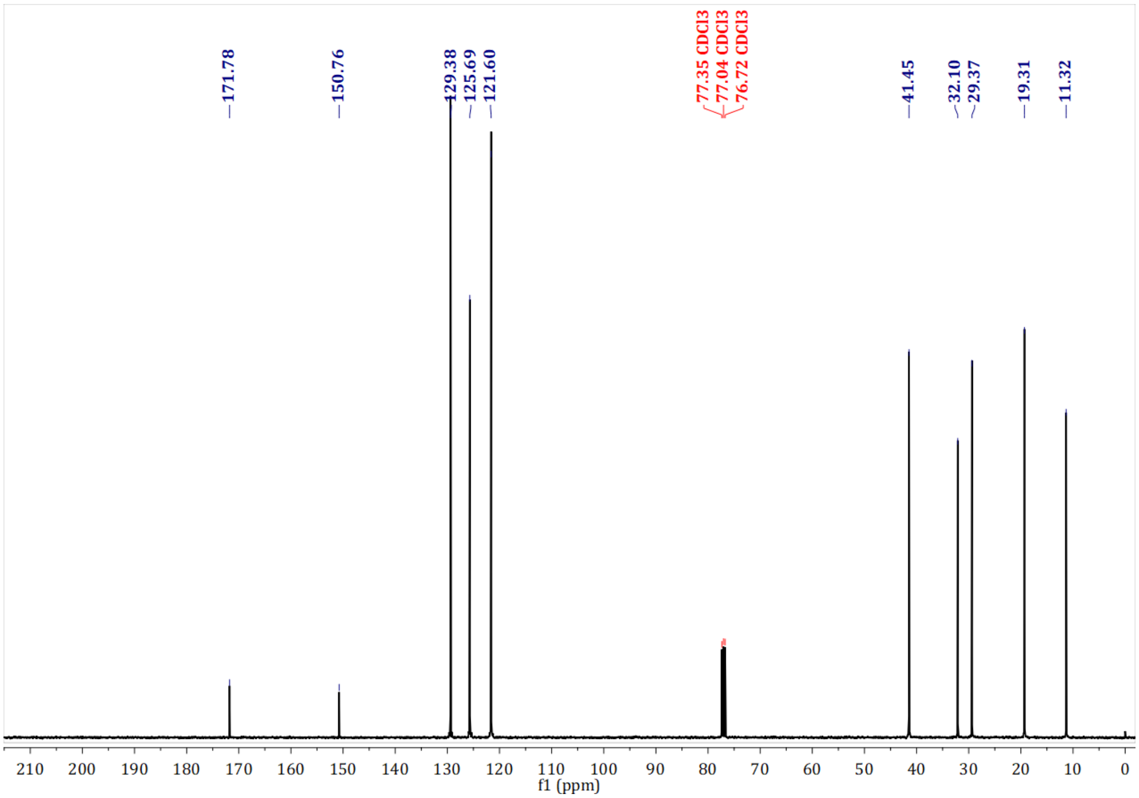

Figure S18. ^{13}C NMR spectrum of phenyl 3-methylpentanoate (**1f**) recorded in $CDCl_3$

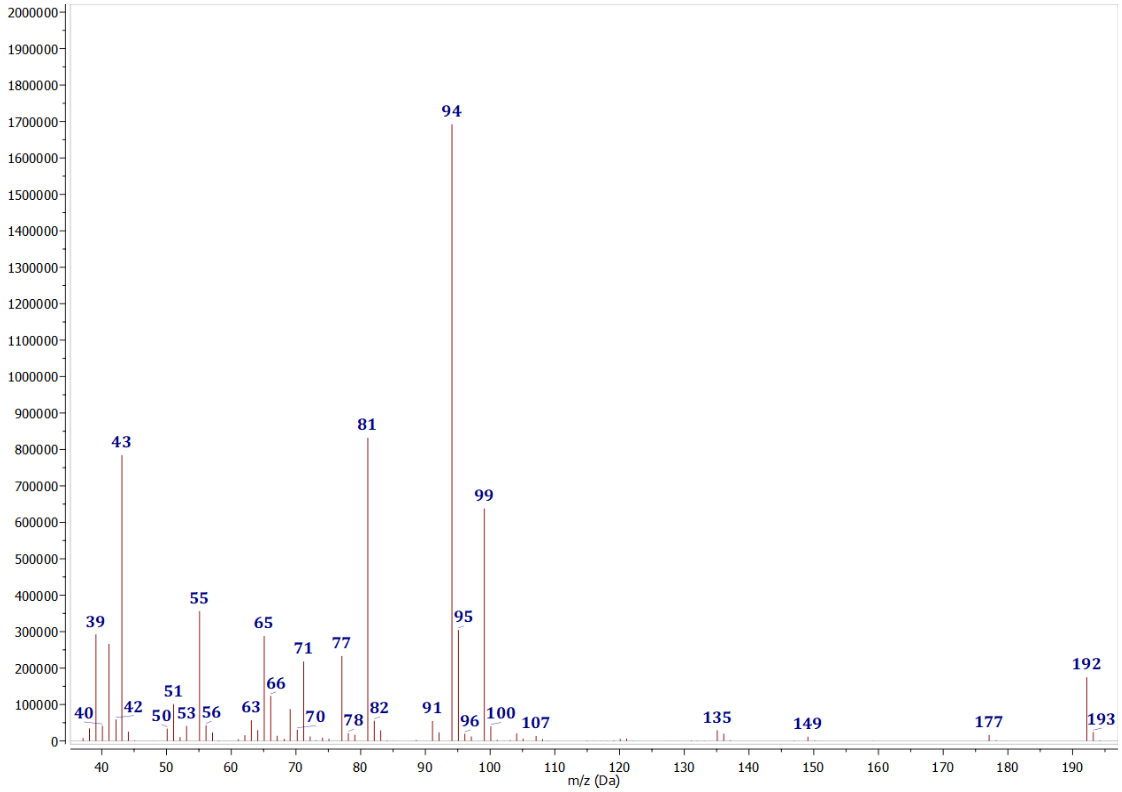

Figure S19. Mass spectrum of phenyl 4-methylpentanoate (**1g**)

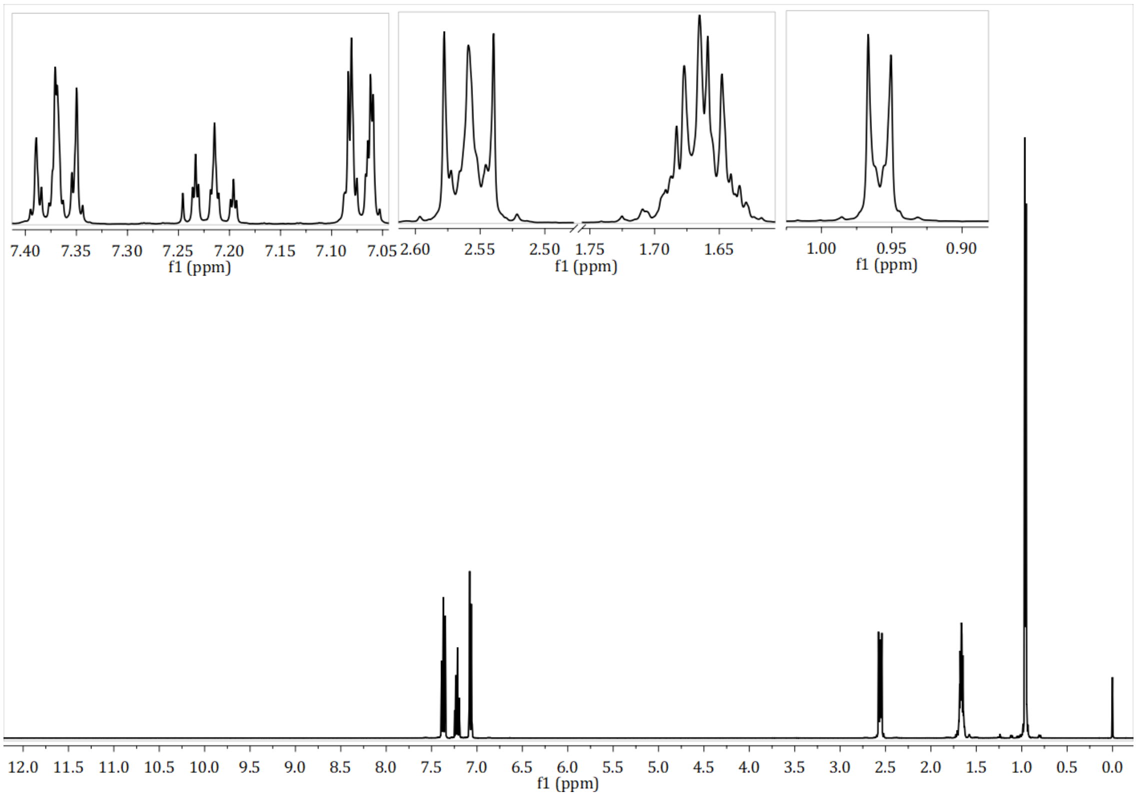

Figure S20. ^{1}H NMR spectrum of phenyl 4-methylpentanoate (**1g**) recorded in $CDCl_3$

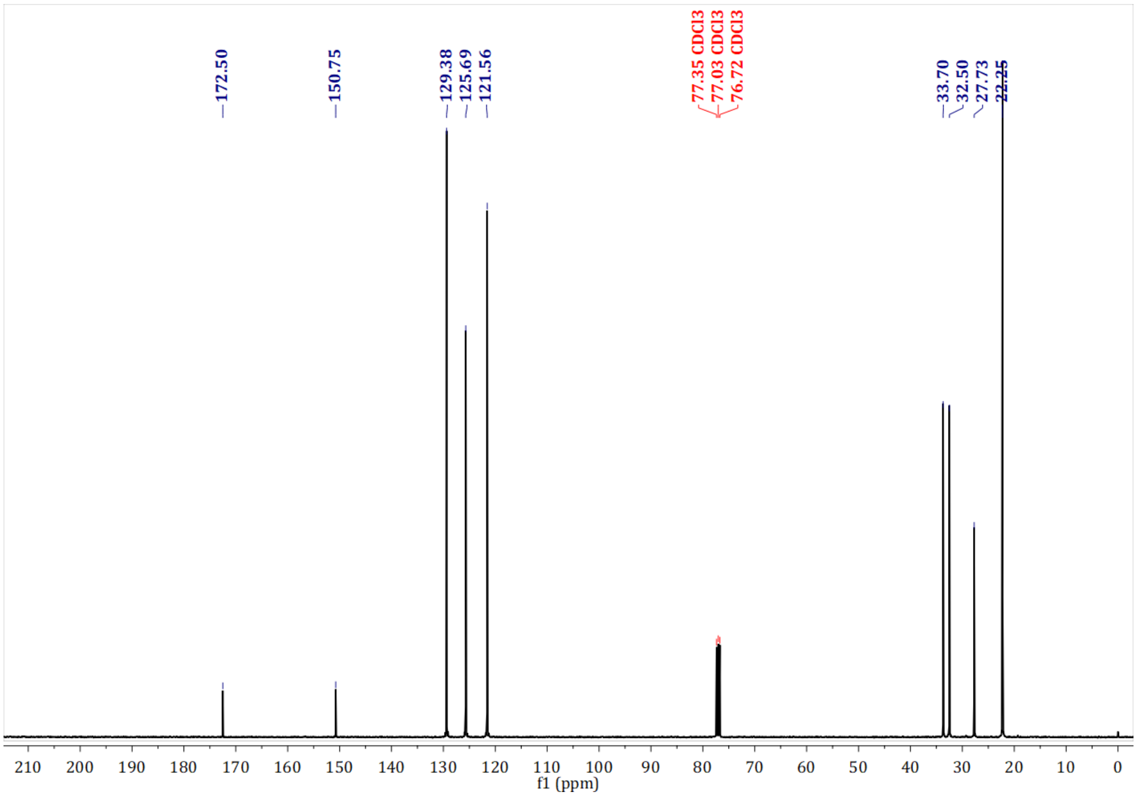

Figure S21. ^{13}C NMR spectrum of phenyl 4-methylpentanoate (**1g**) recorded in $CDCl_3$

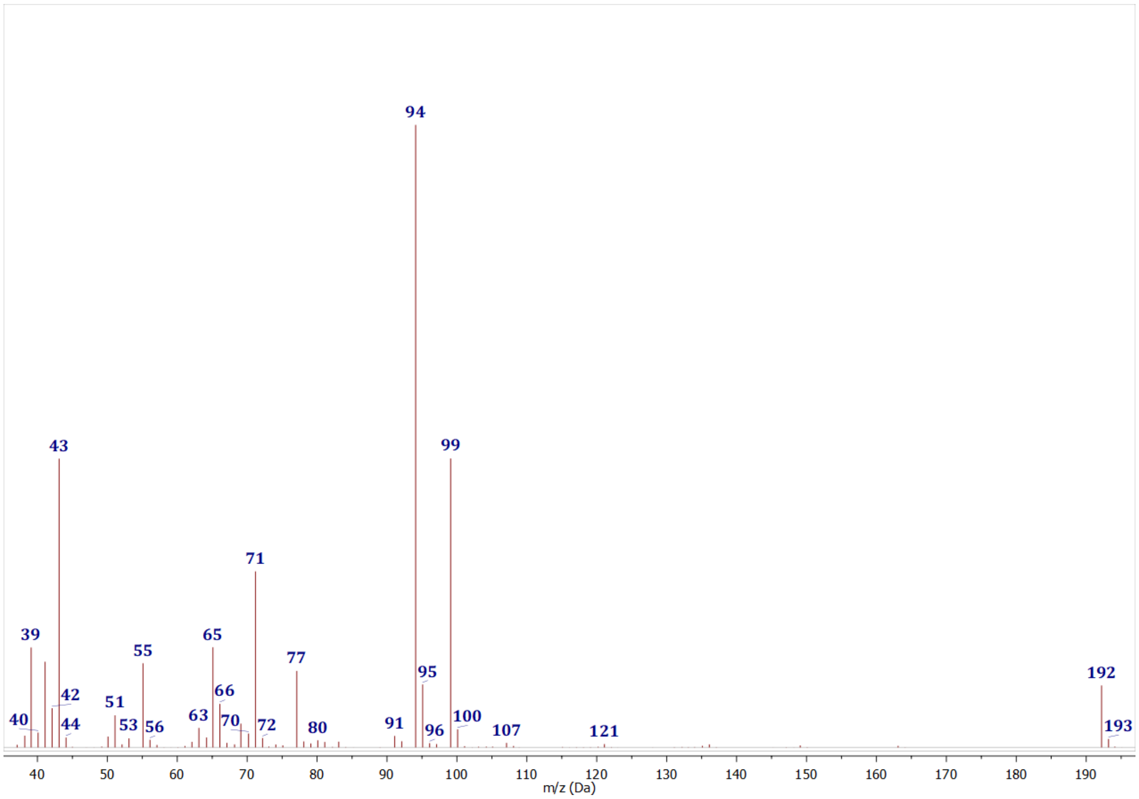

Figure S22. Mass spectrum of phenyl hexanoate (**1h**)

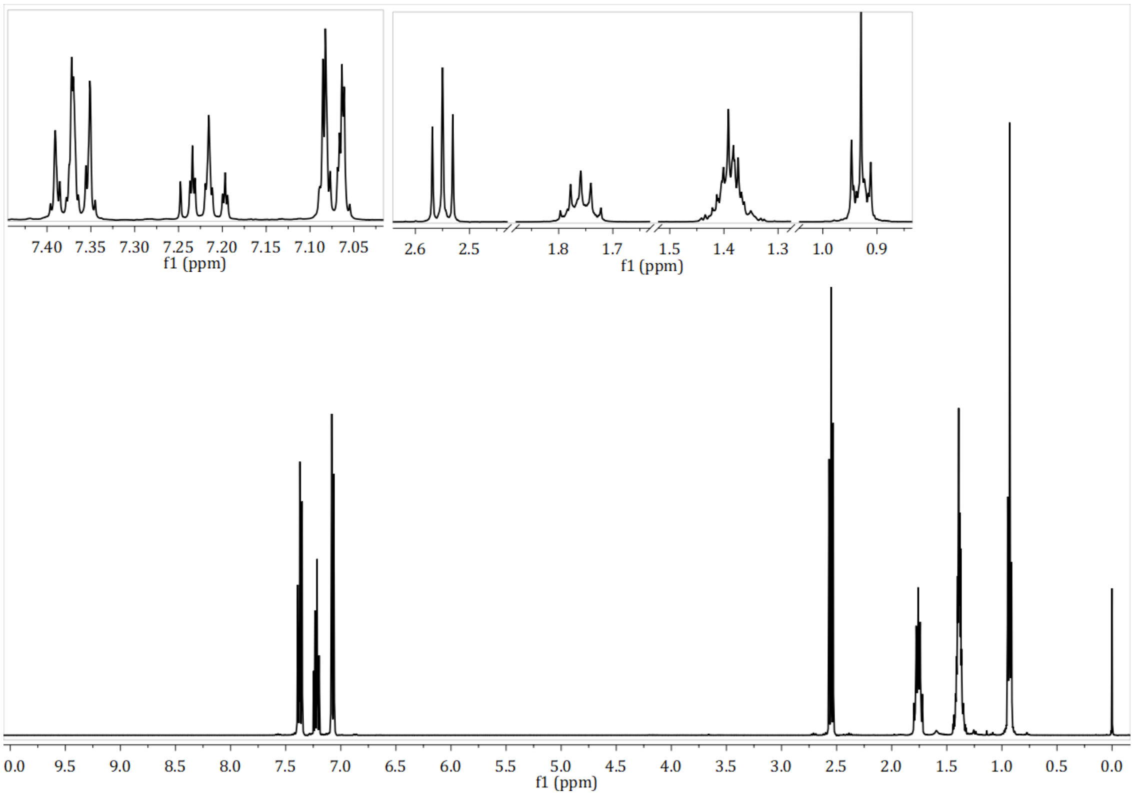

Figure S23. ^{1}H NMR spectrum of phenyl hexanoate (**1h**) recorded in $CDCl_3$

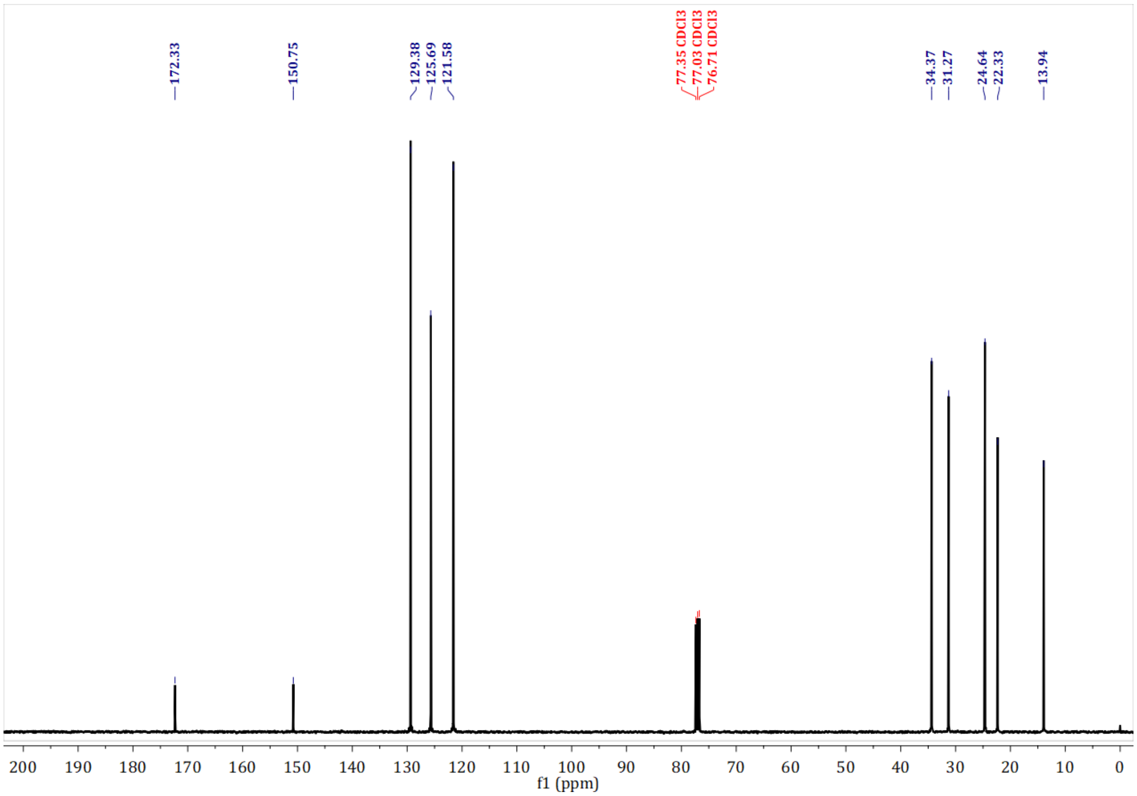

Figure S24. ^{13}C NMR spectrum of phenyl hexanoate (**1h**) recorded in $CDCl_3$

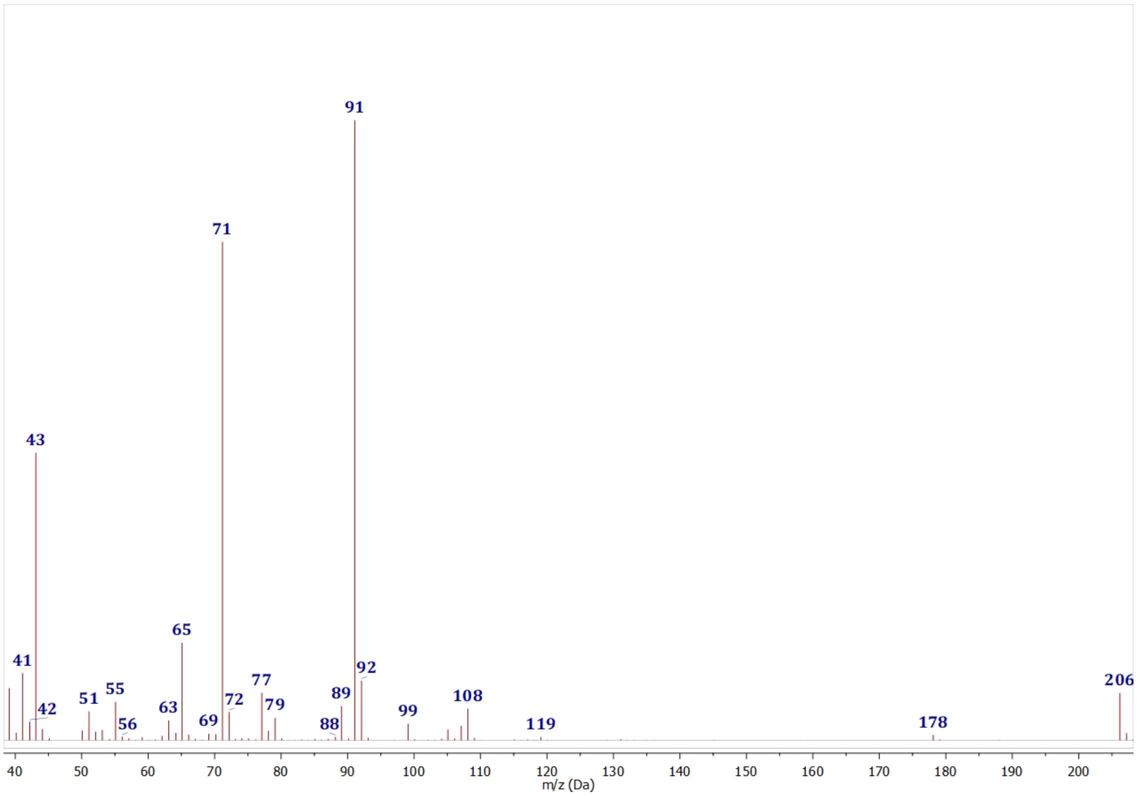

Figure S25. Mass spectrum of benzyl 2-ethylbutanoate (**2a**)

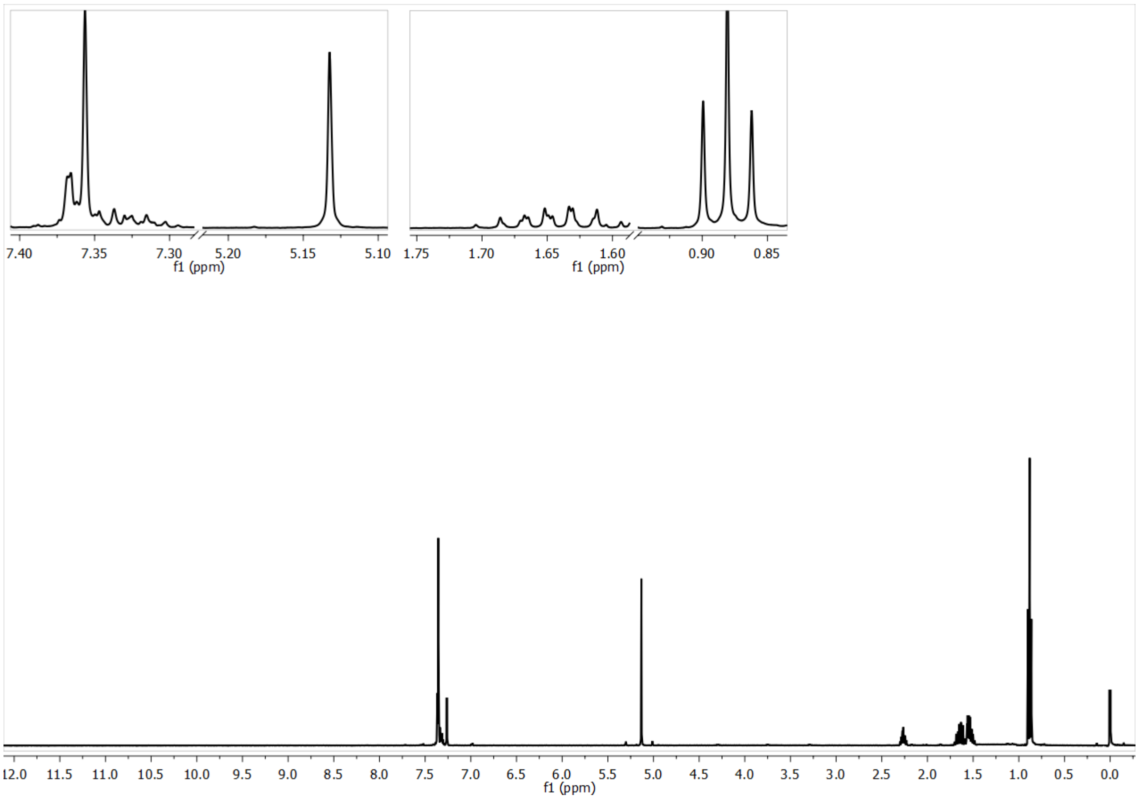

Figure S26. ^{1}H NMR spectrum of benzyl 2-ethylbutanoate (**2a**) recorded in $CDCl_3$

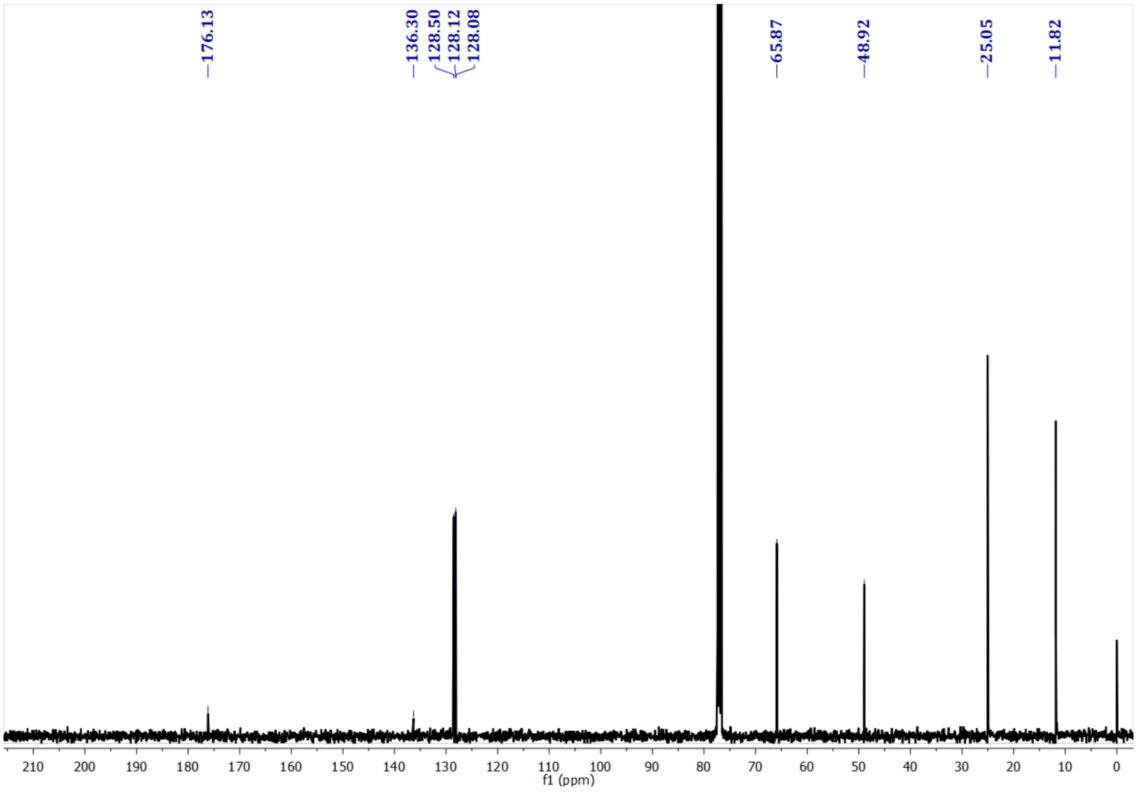

Figure S27. ^{13}C NMR spectrum of benzyl 2-ethylbutanoate (**2a**) recorded in $CDCl_3$

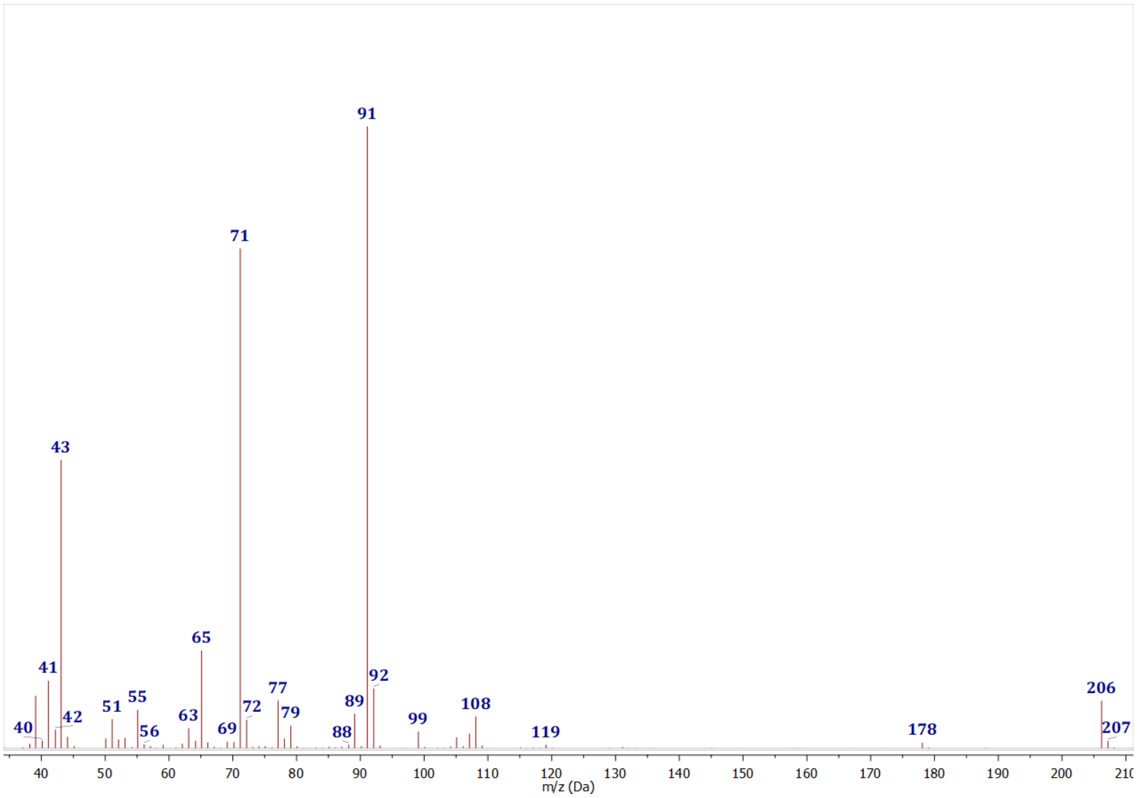

Figure S28. Mass spectrum of benzyl 2,2-dimethylbutanoate (**2b**)

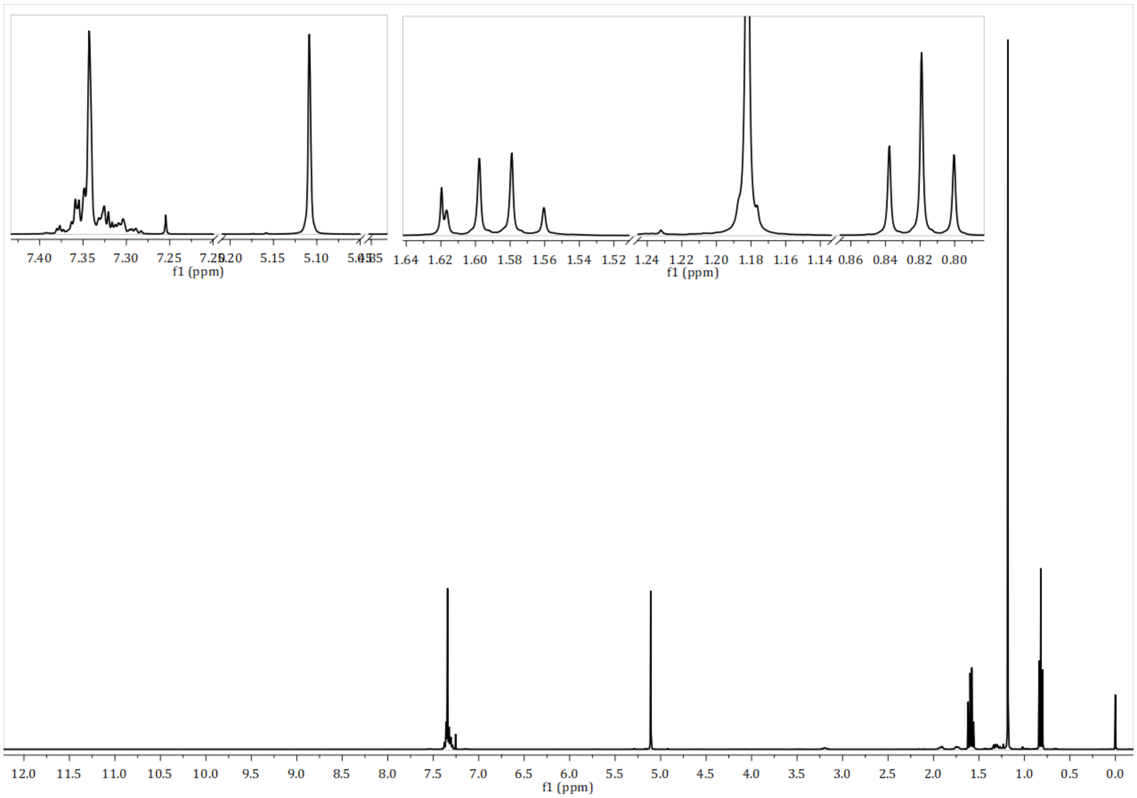

Figure S29. ^{1}H NMR spectrum of benzyl 2,2-dimethylbutanoate (**2b**) recorded in $CDCl_3$

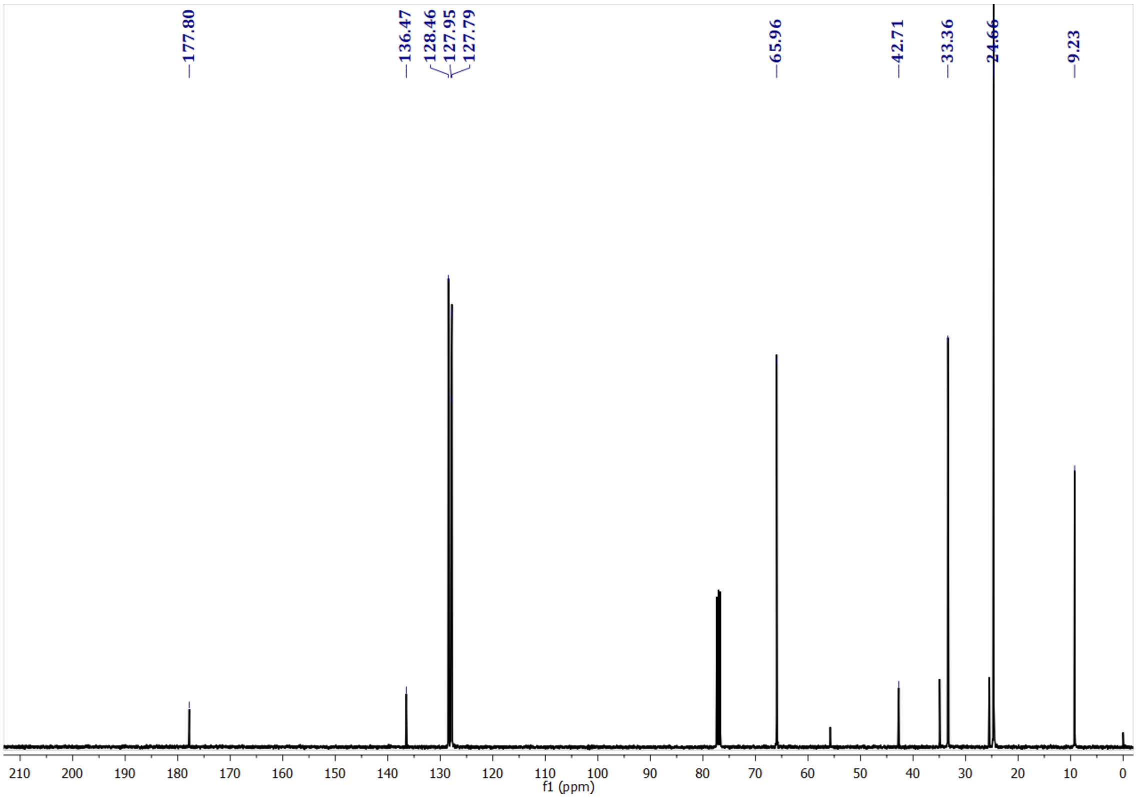

Figure S30. ^{13}C NMR spectrum of benzyl 2,2-dimethylbutanoate (**2b**) recorded in $CDCl_3$

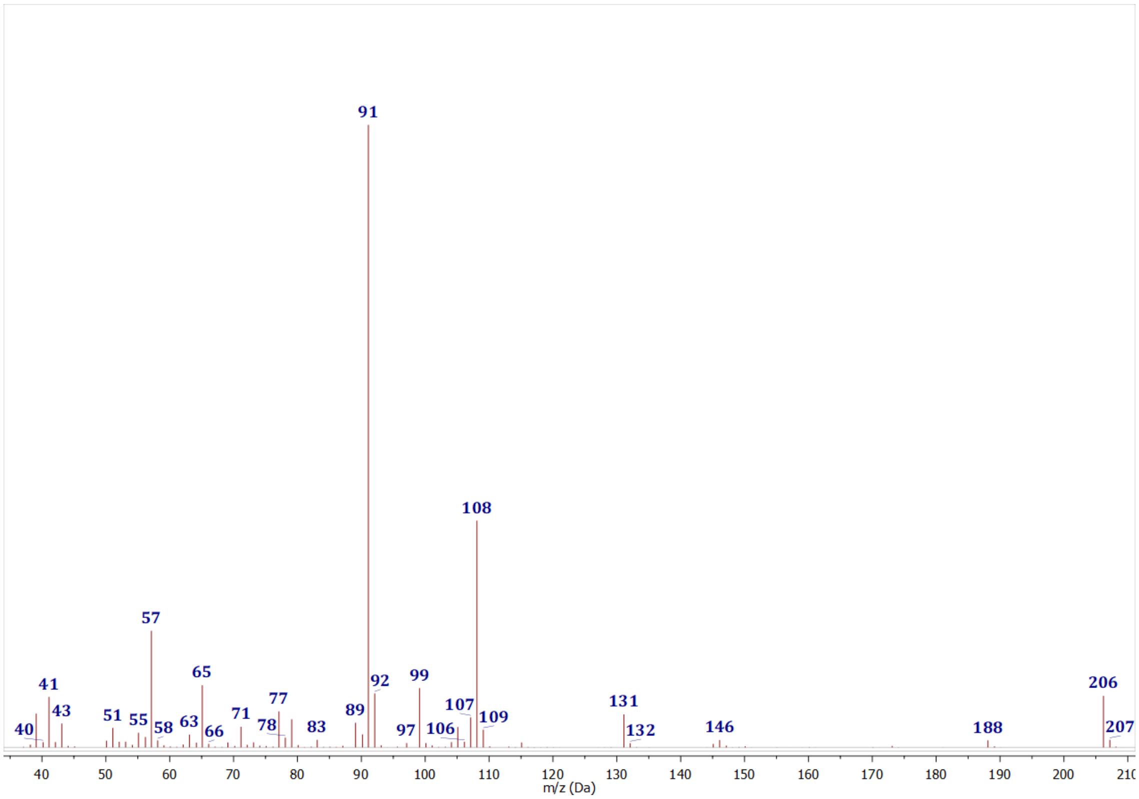

Figure S31. Mass spectrum of benzyl 3,3-dimethylbutanoate (**2c**)

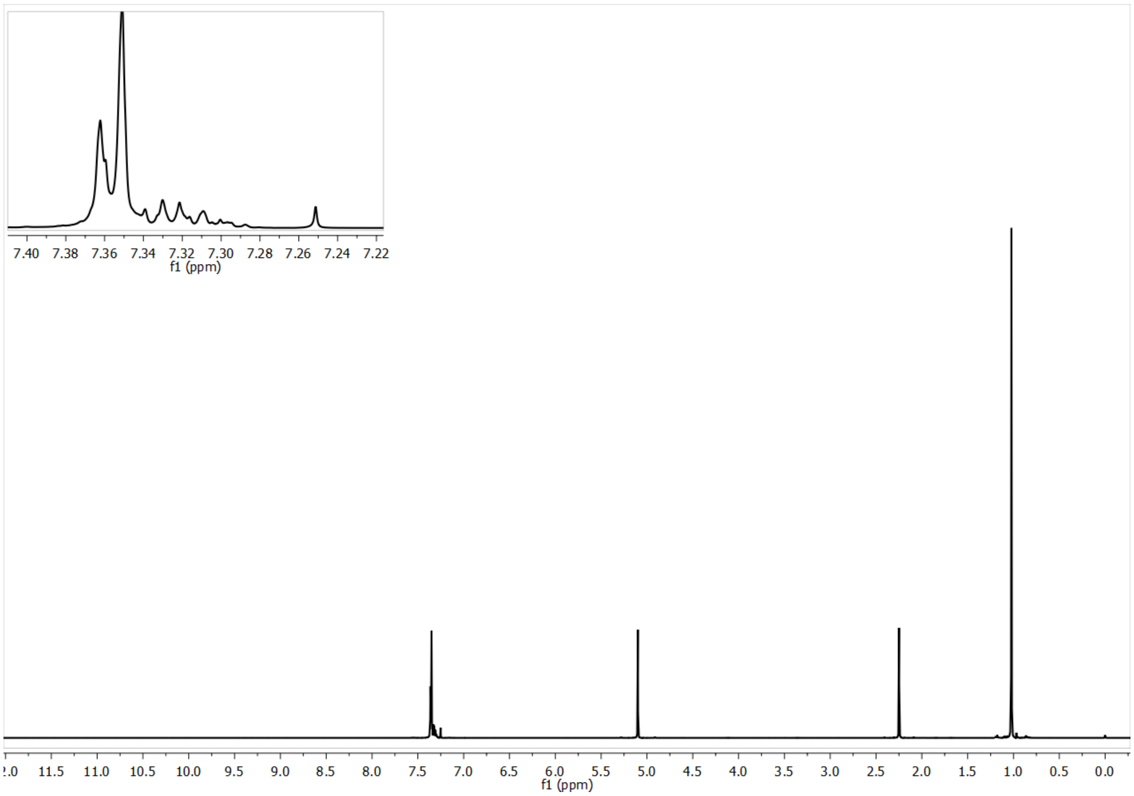

Figure S32. ^{1}H NMR spectrum of benzyl 3,3-dimethylbutanoate (**2c**) recorded in $CDCl_3$

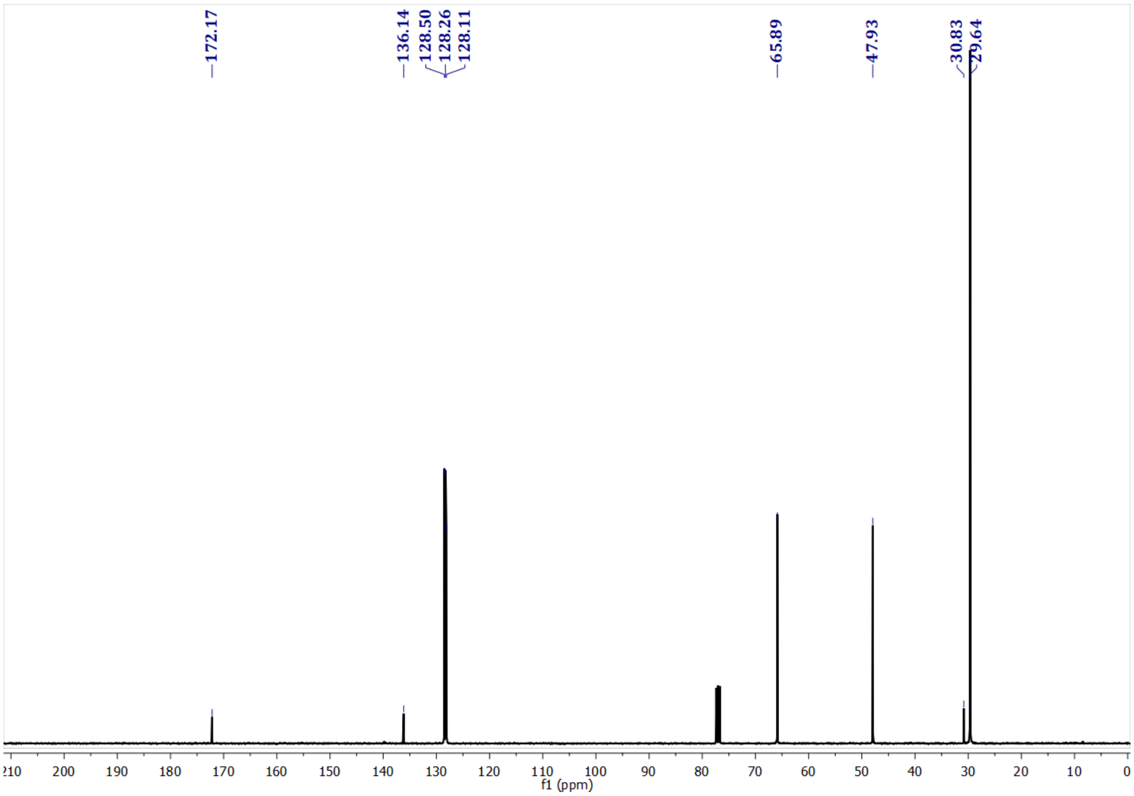

Figure S33. ^{13}C NMR spectrum of benzyl 3,3-dimethylbutanoate (**2c**) recorded in $CDCl_3$

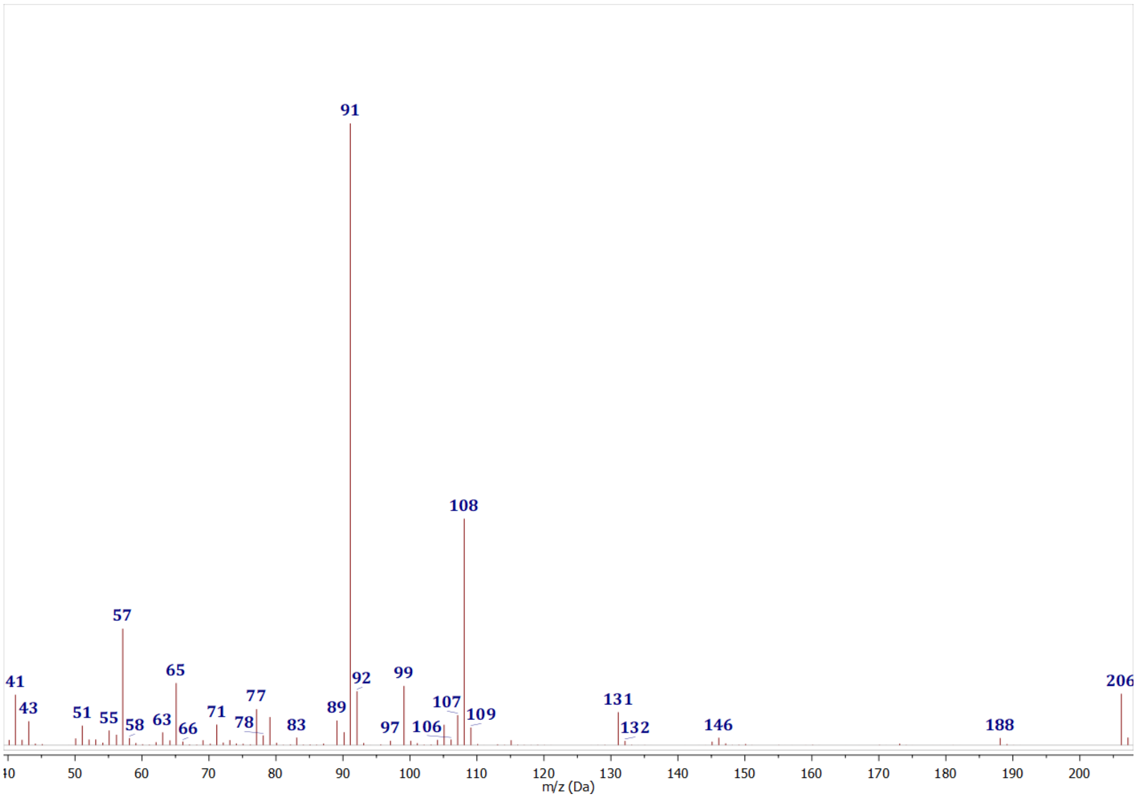

Figure S34. Mass spectrum of benzyl 2,3-dimethylbutanoate (**2d**)

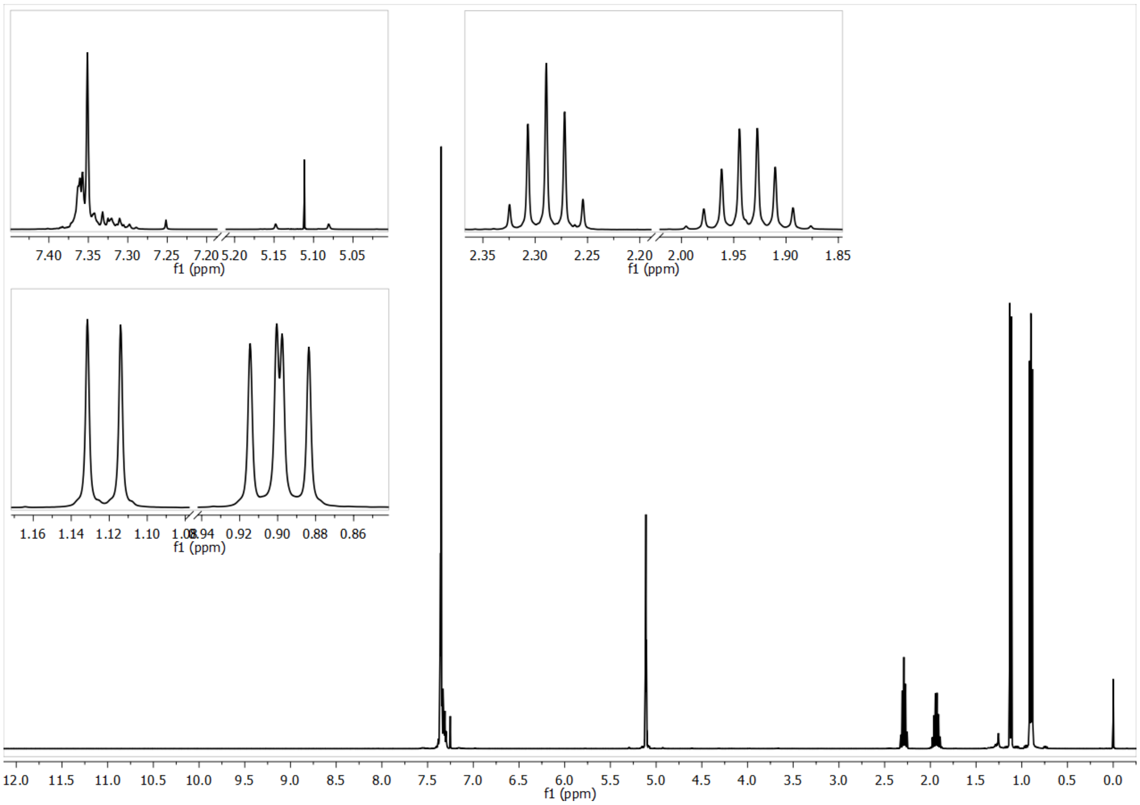

Figure S35. ^{1}H NMR spectrum of benzyl 2,3-dimethylbutanoate (**2d**) recorded in $CDCl_3$

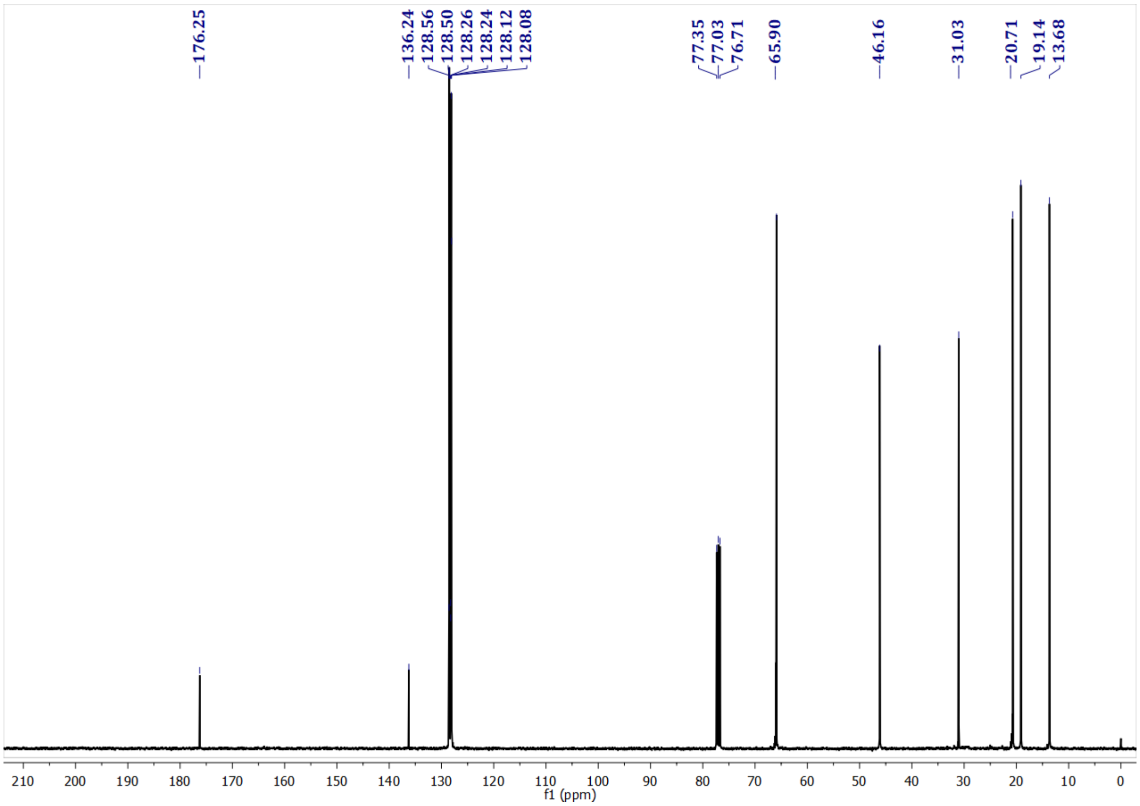

Figure S36. ^{13}C NMR spectrum of benzyl 2,3-dimethylbutanoate (**2d**) recorded in $CDCl_3$

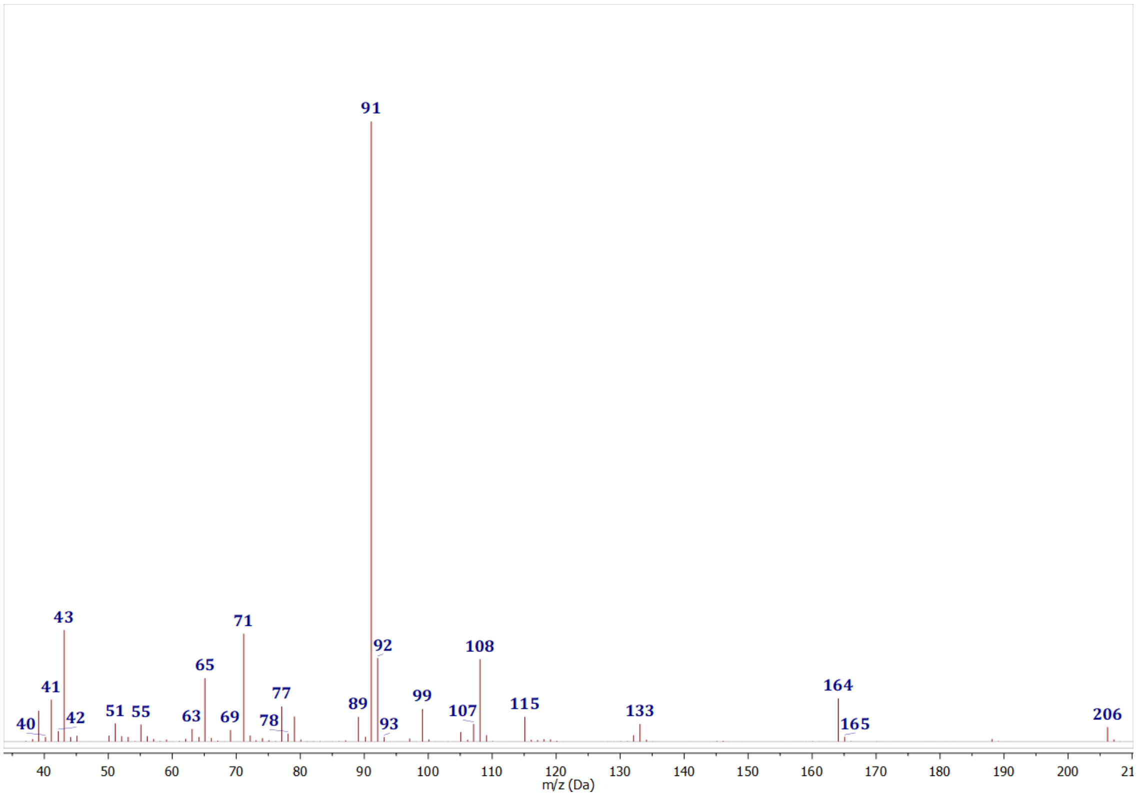

Figure S37. Mass spectrum of benzyl 2-methylpentanoate (**2e**)

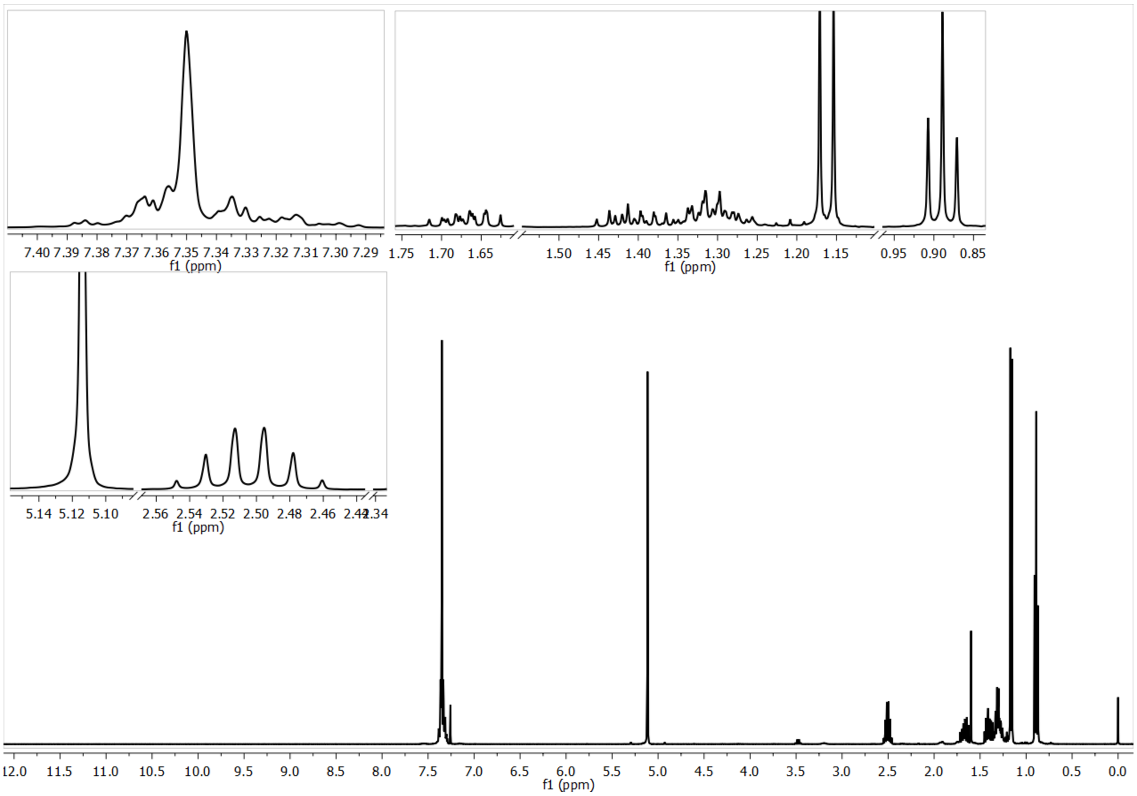

Figure S38. ^{1}H NMR spectrum of benzyl 2-methylpentanoate (**2e**) recorded in $CDCl_3$

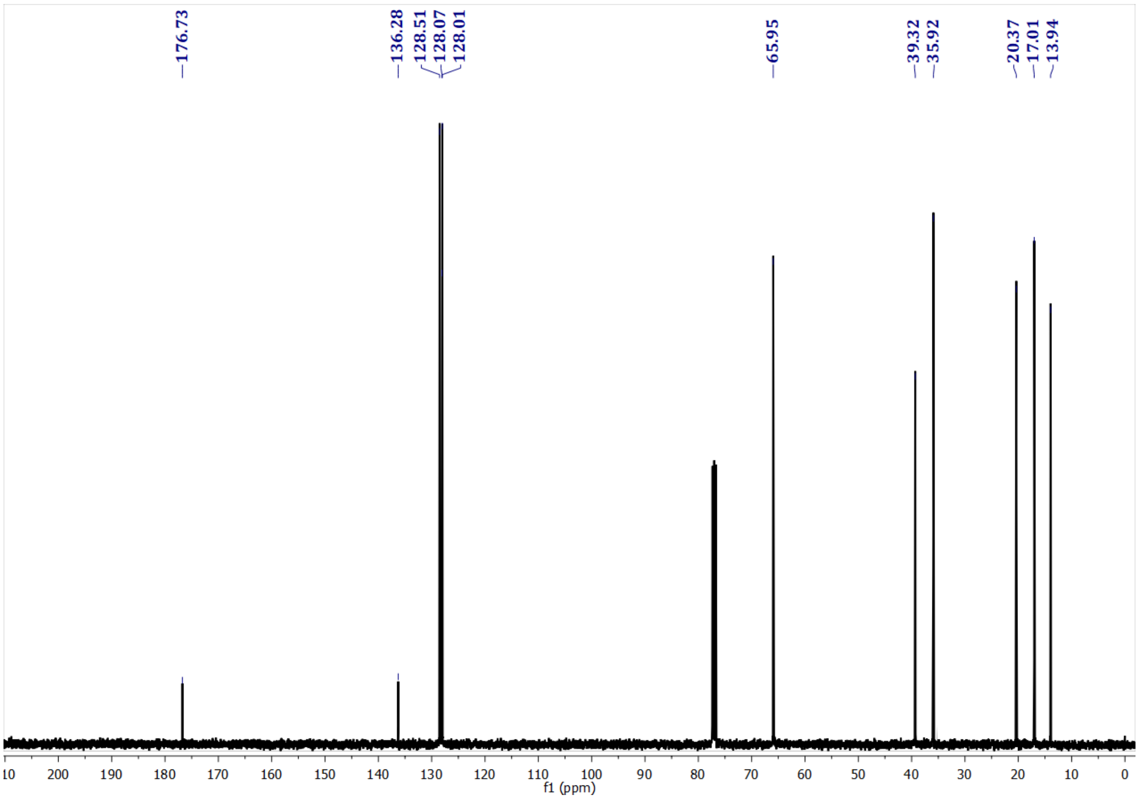

Figure S39. ^{13}C NMR spectrum of benzyl 2-methylpentanoate (**2e**) recorded in $CDCl_3$

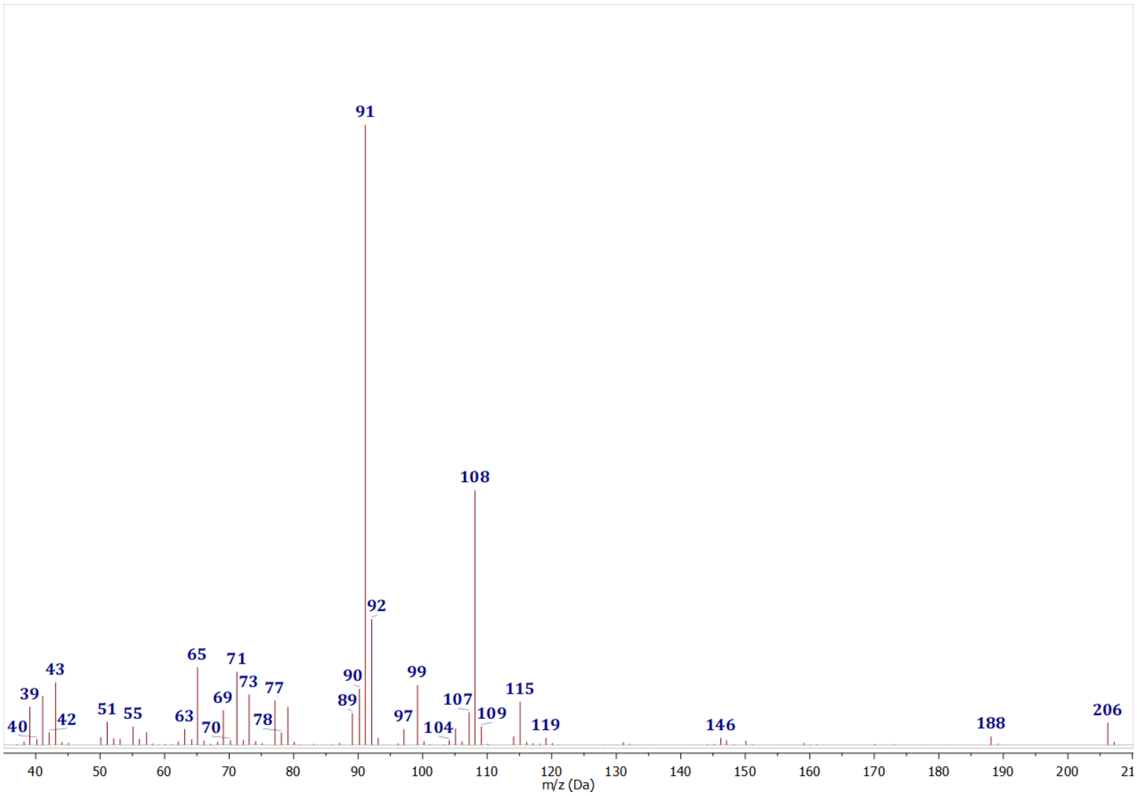

Figure S40. Mass spectrum of benzyl 3-methylpentanoate (**2f**)

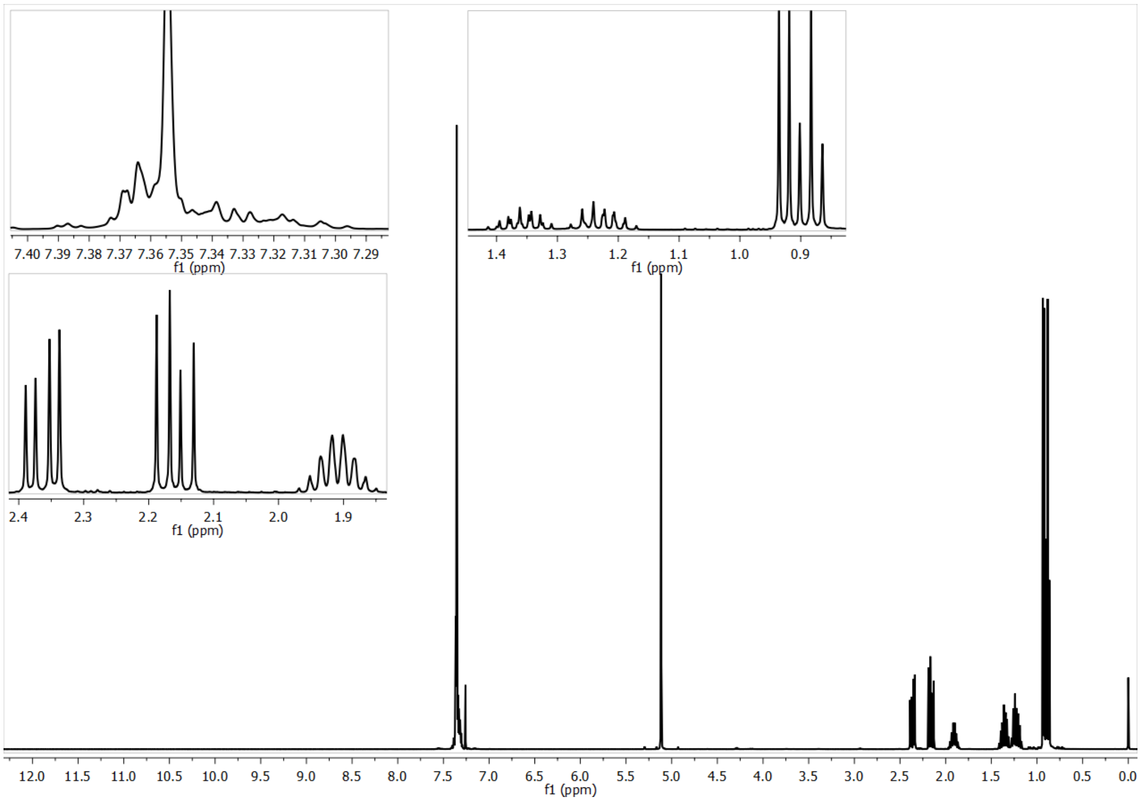

Figure S41. ^{1}H NMR spectrum of benzyl 3-methylpentanoate (**2f**) recorded in $CDCl_3$

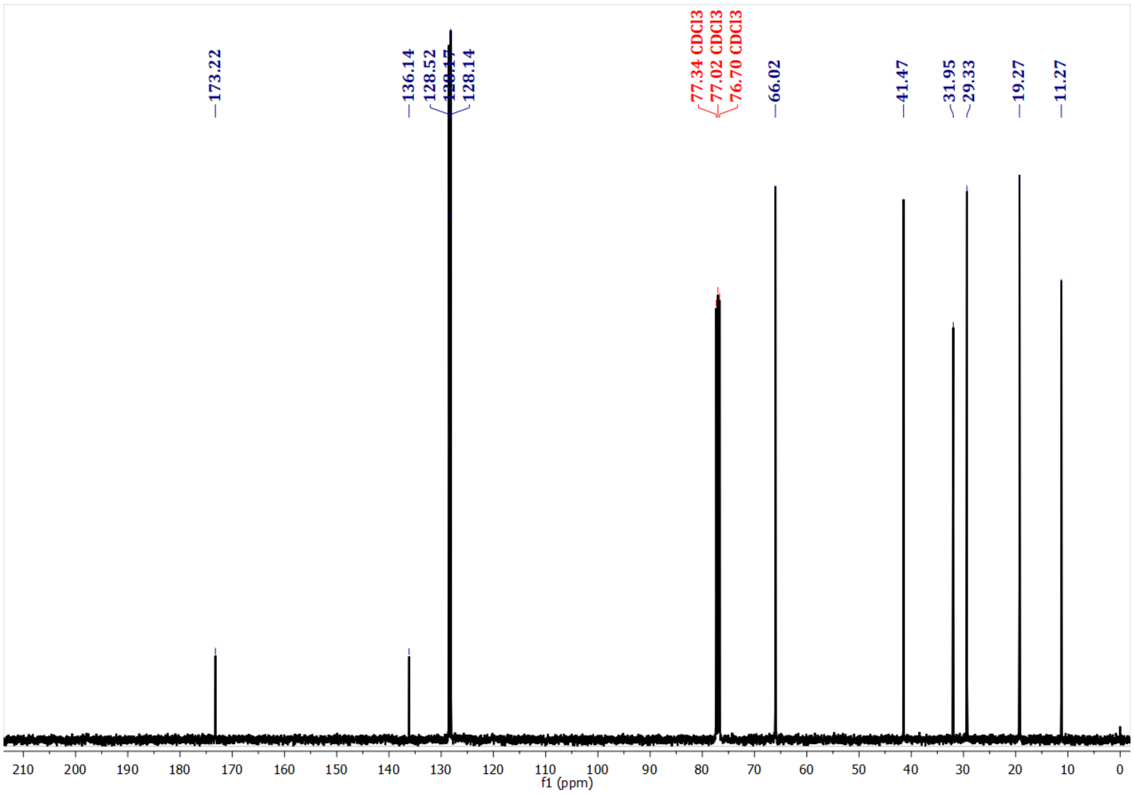

Figure S42. ^{13}C NMR spectrum of benzyl 3-methylpentanoate (**2f**) recorded in $CDCl_3$

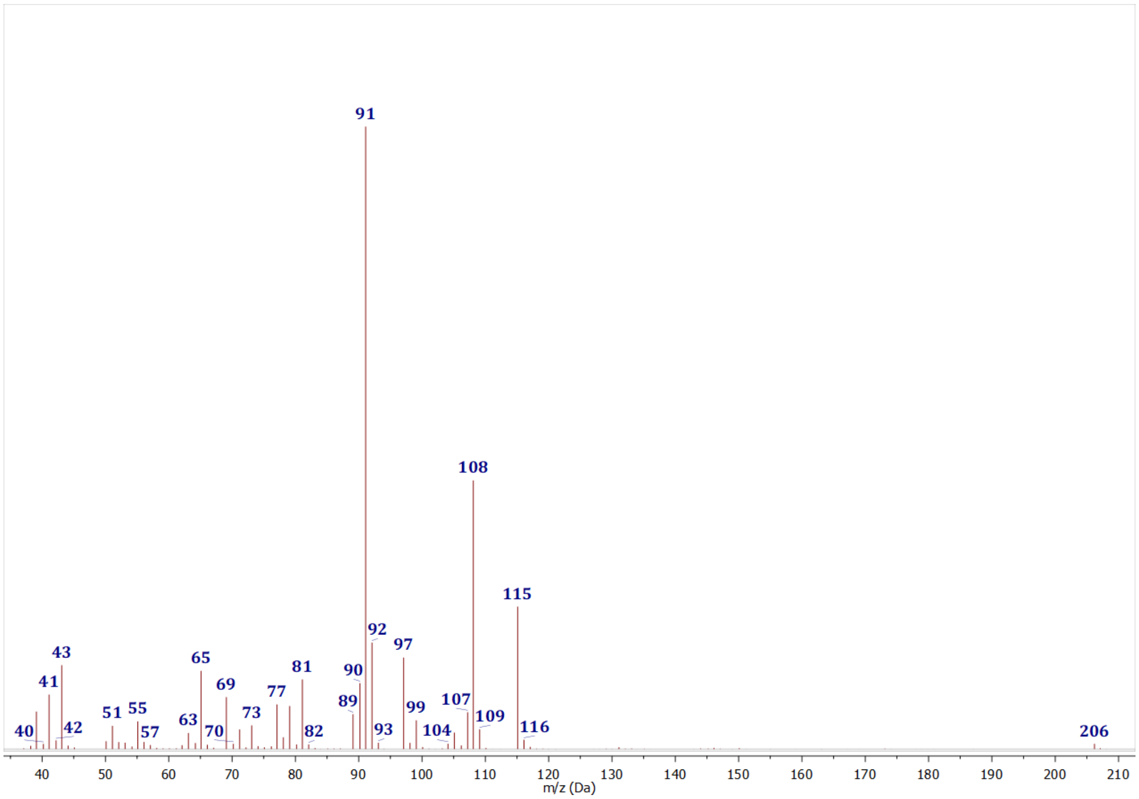

Figure S43. Mass spectrum of benzyl 4-methylpentanoate (**2g**)

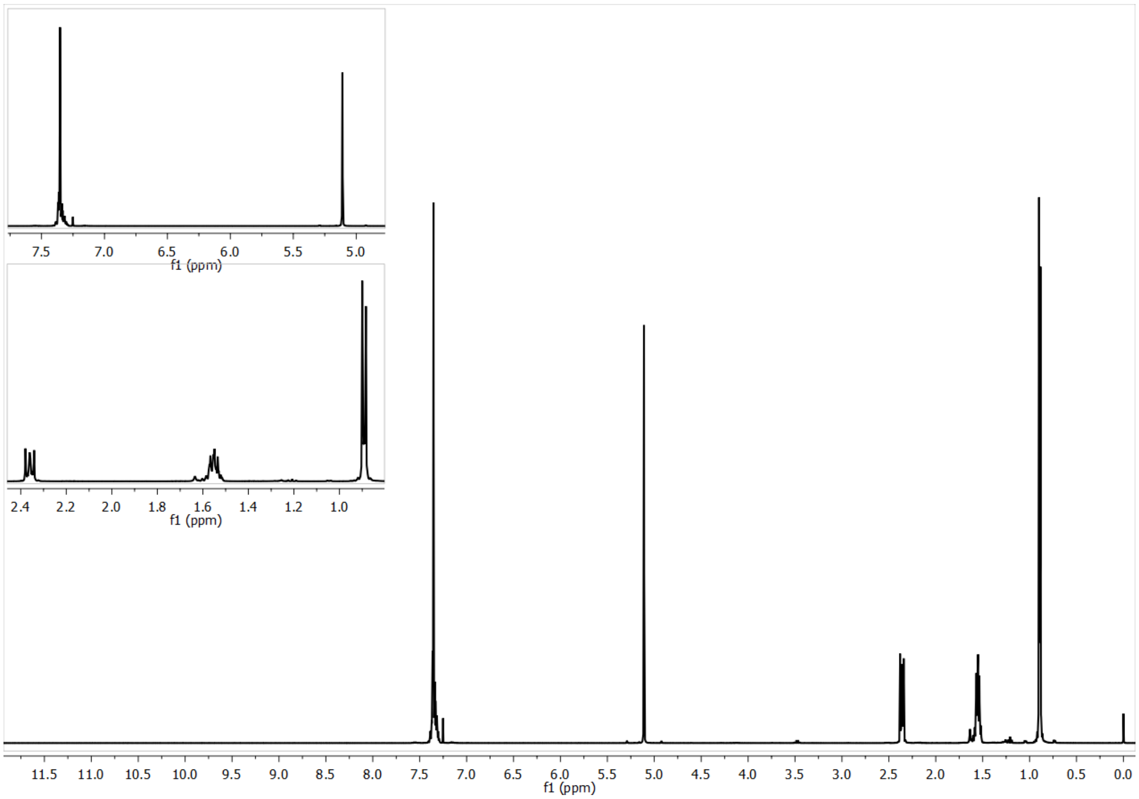

Figure S44. ^{1}H NMR spectrum of benzyl 4-methylpentanoate (**2g**) recorded in $CDCl_3$

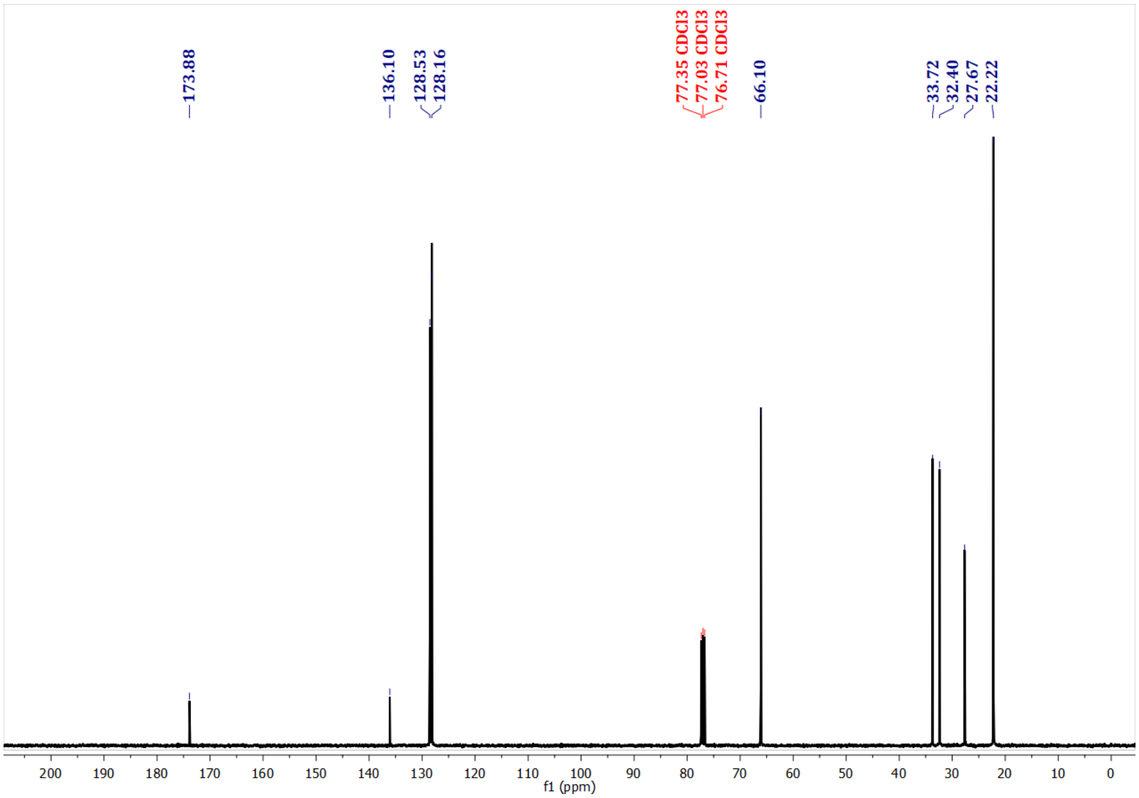

Figure S45. ^{13}C NMR spectrum of benzyl 4-methylpentanoate (**2g**) recorded in $CDCl_3$

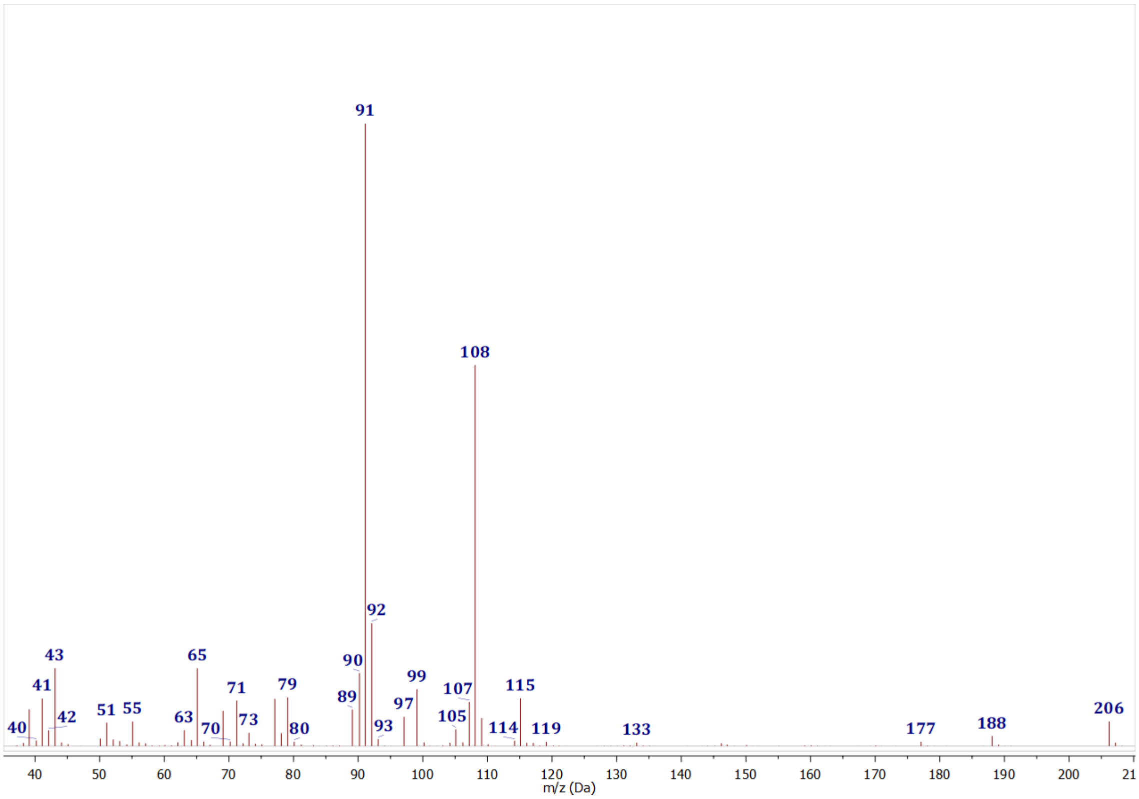

Figure S46. Mass spectrum of benzyl hexanoate (**2h**)

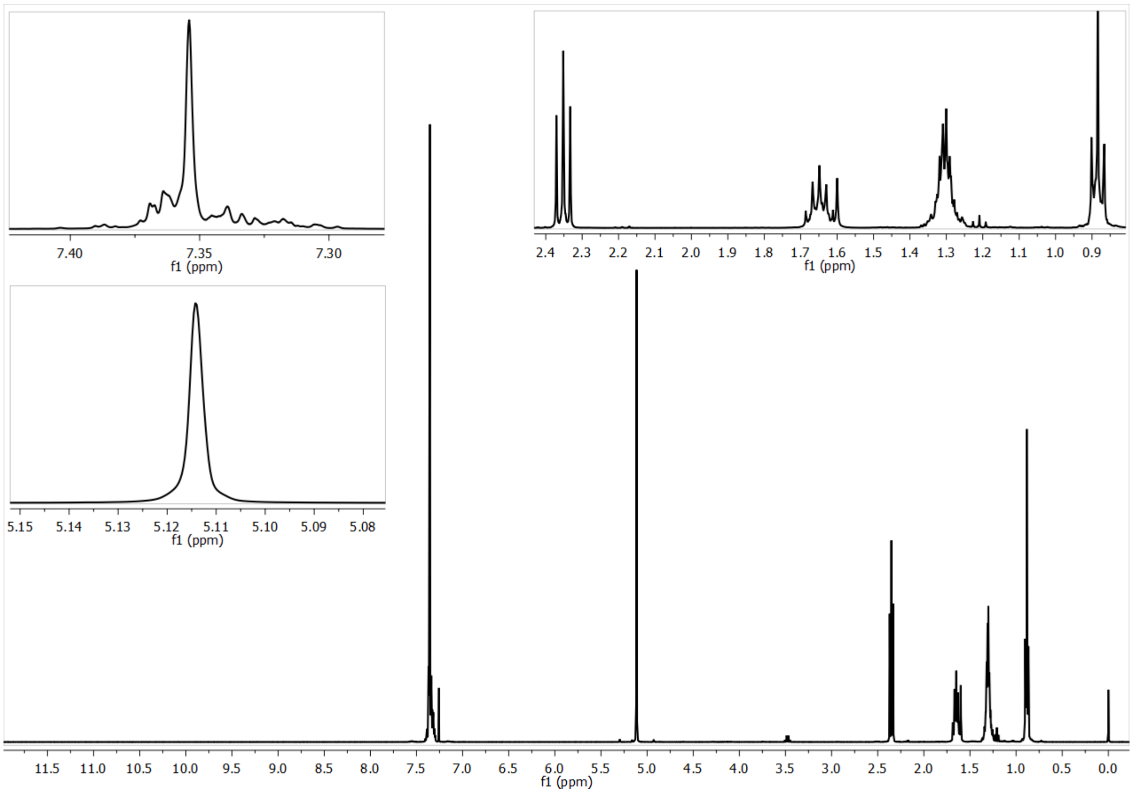

Figure S47. ^{1}H NMR spectrum of benzyl hexanoate (**2h**) recorded in $CDCl_3$

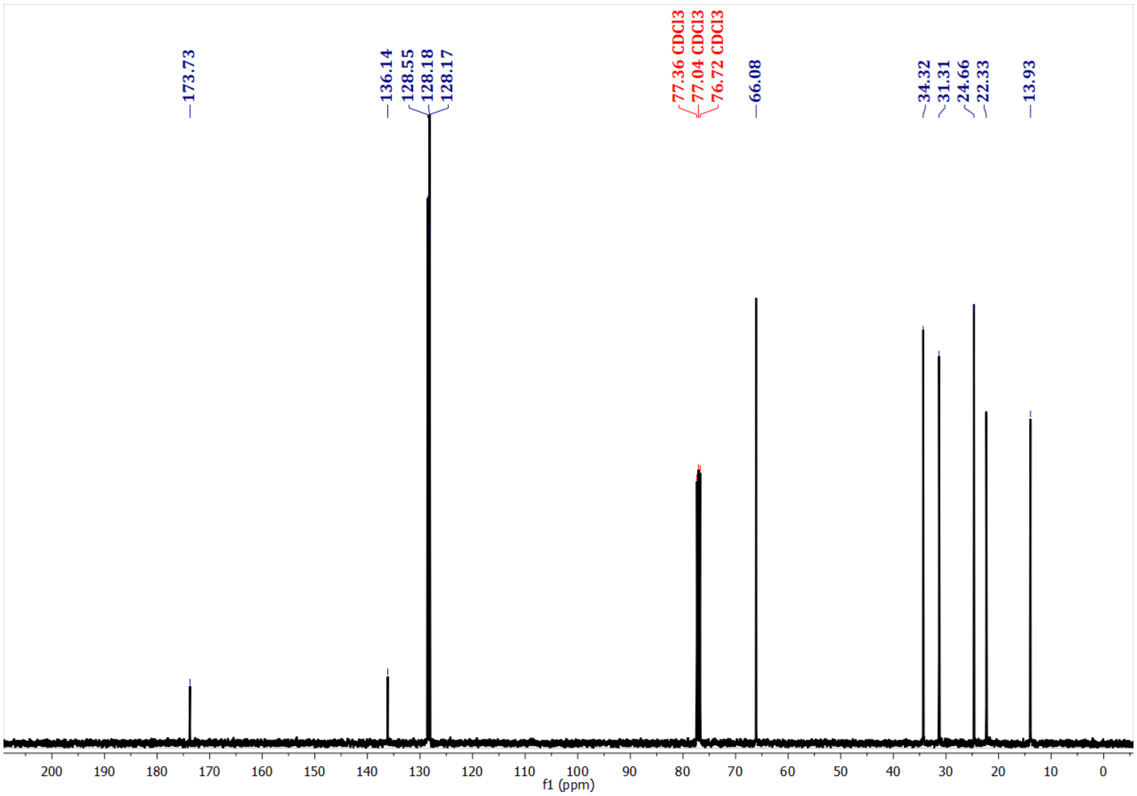

Figure S48. ^{13}C NMR spectrum of benzyl hexanoate (**2h**) recorded in $CDCl_3$

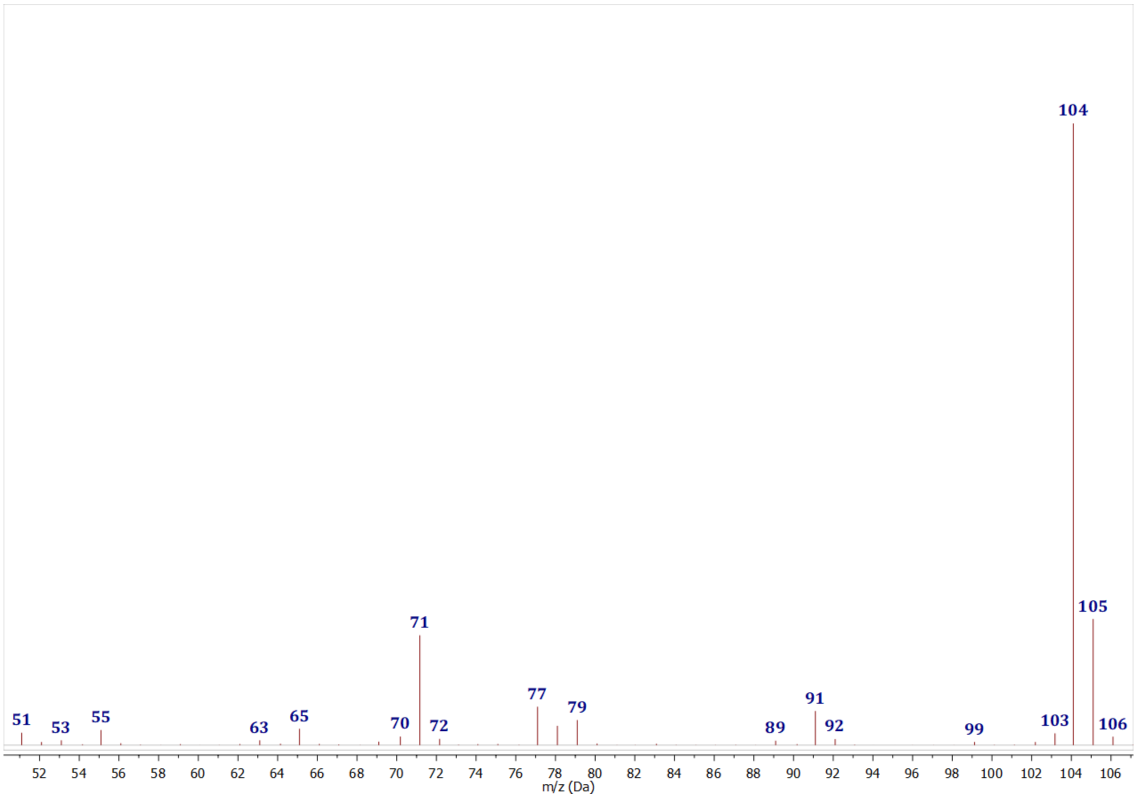

Figure S49. Mass spectrum of phenethyl 2-ethylbutanoate (**3a**)

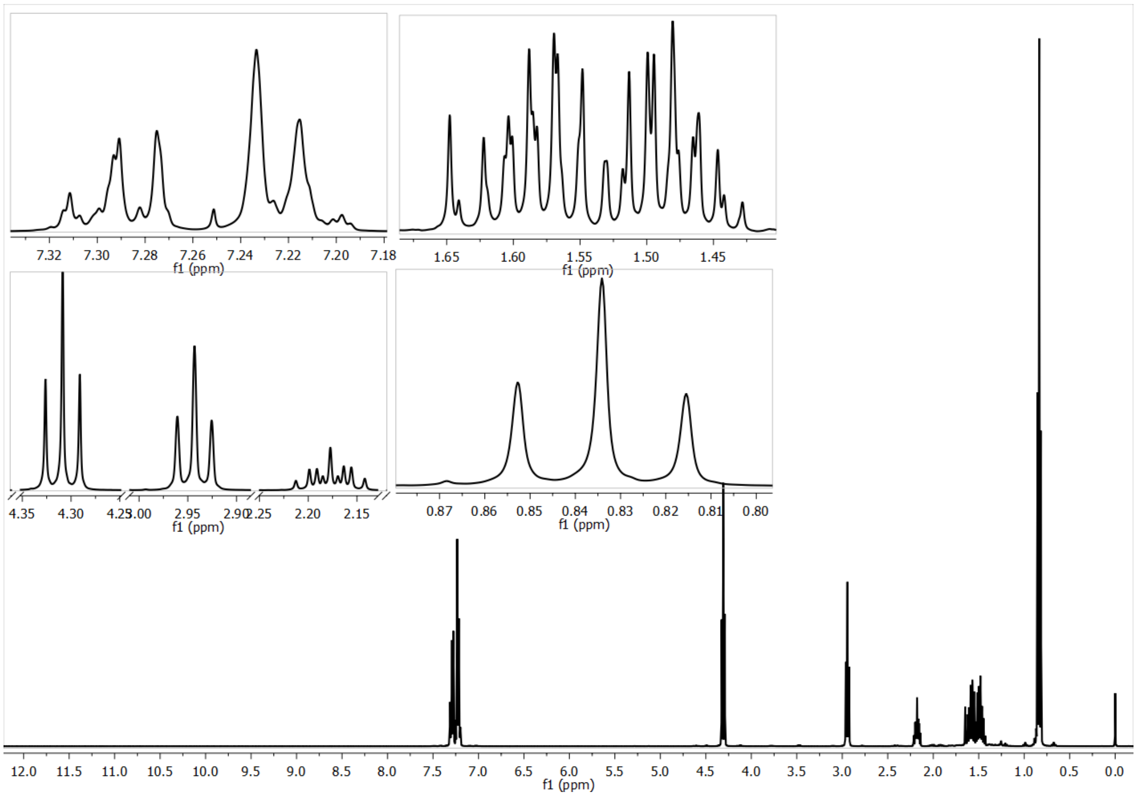

Figure S50. ^{1}H NMR spectrum of phenethyl 2-ethylbutanoate (**3a**) recorded in $CDCl_3$

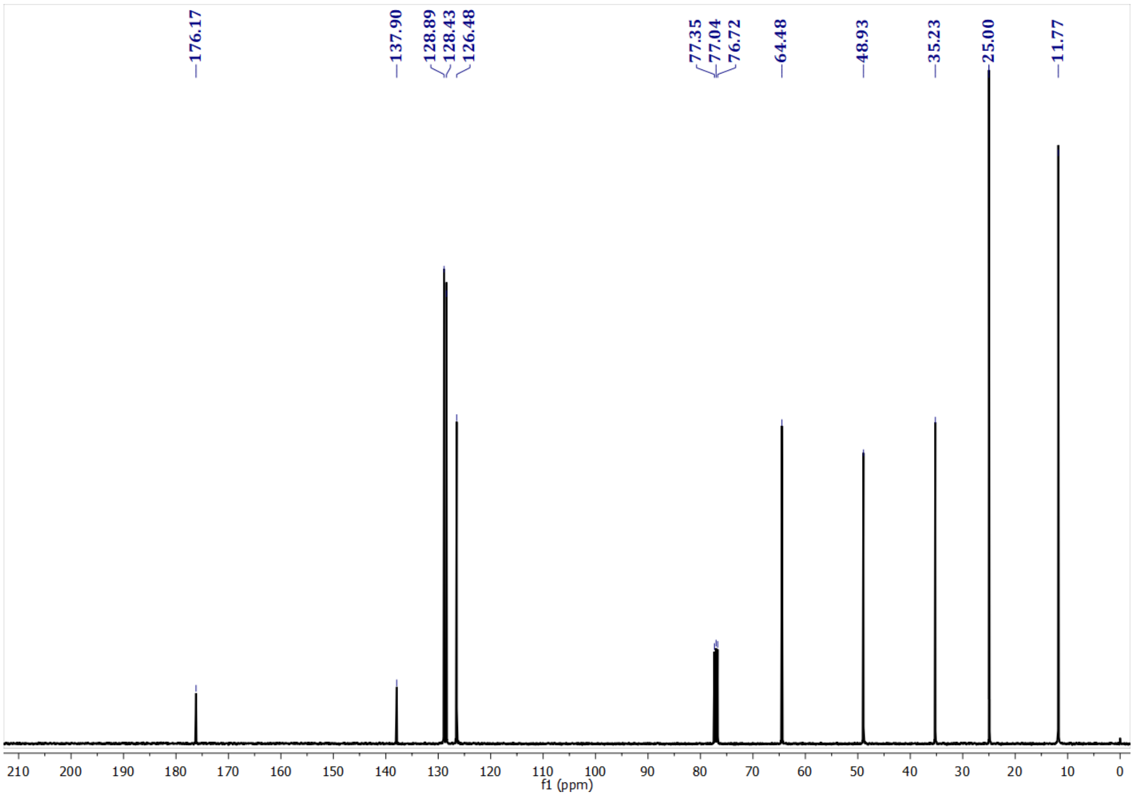

Figure S51. ^{13}C NMR spectrum of phenethyl 2-ethylbutanoate (**3a**) recorded in $CDCl_3$

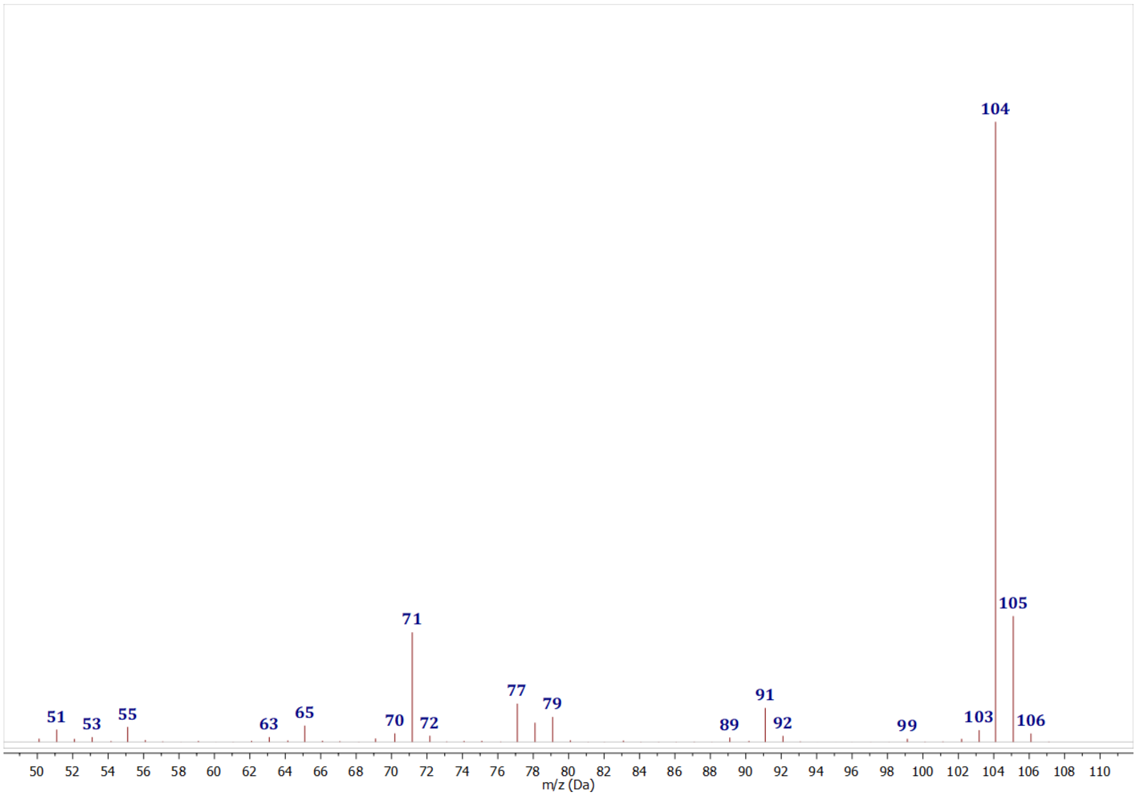

Figure S52. Mass spectrum of phenethyl 2,2-dimethylbutanoate (**3b**)

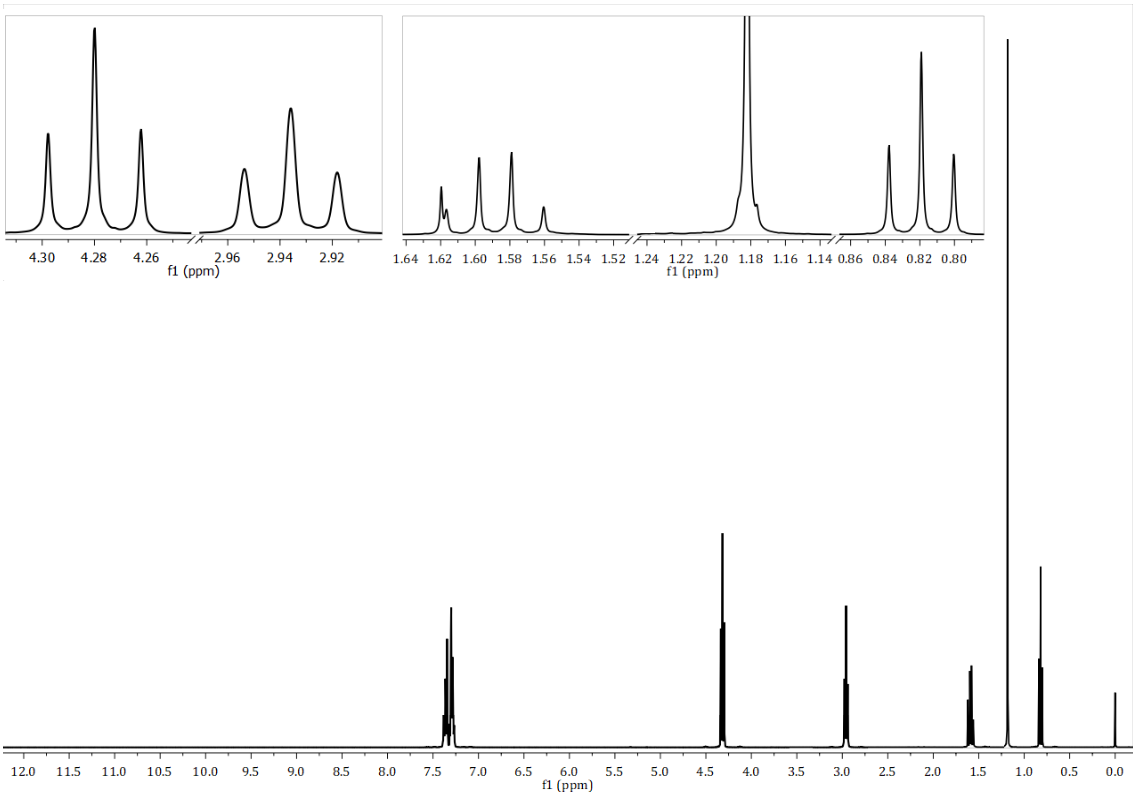

Figure S53. ^{1}H NMR spectrum of phenethyl 2,2-dimethylbutanoate (**3b**) recorded in $CDCl_3$

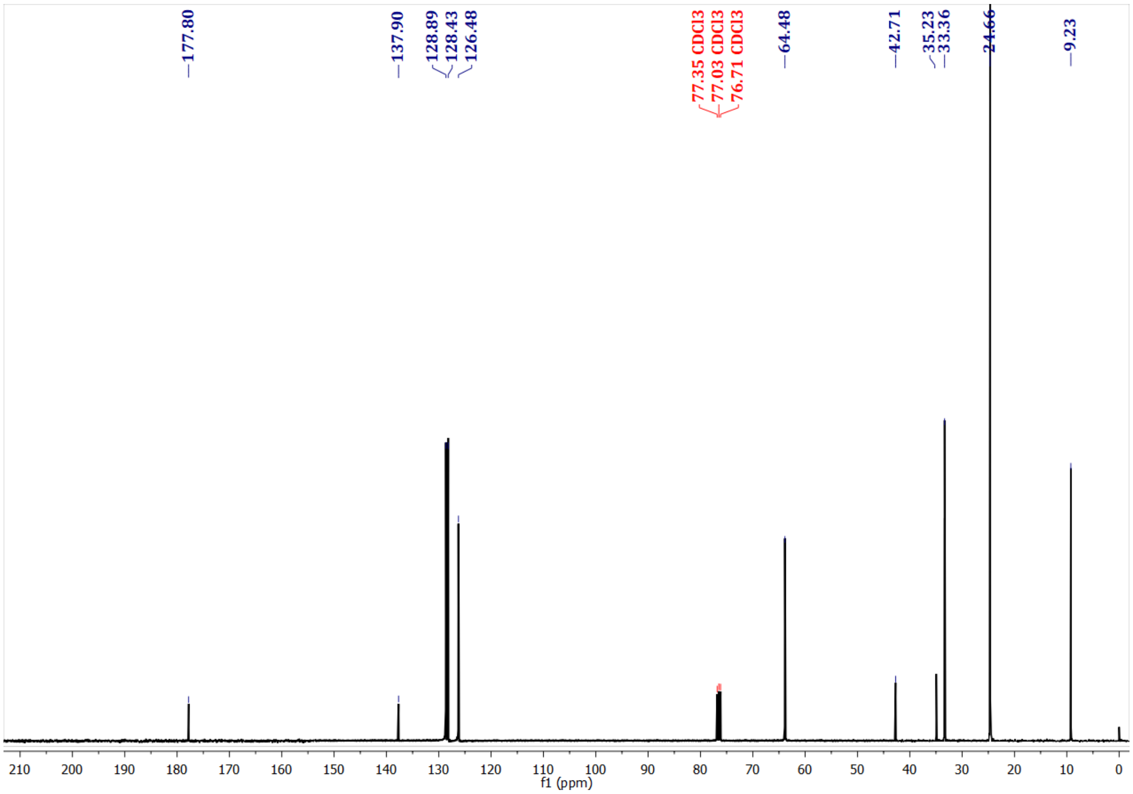

Figure S54. ^{13}C NMR spectrum of phenethyl 2,2-dimethylbutanoate (**3b**) recorded in $CDCl_3$

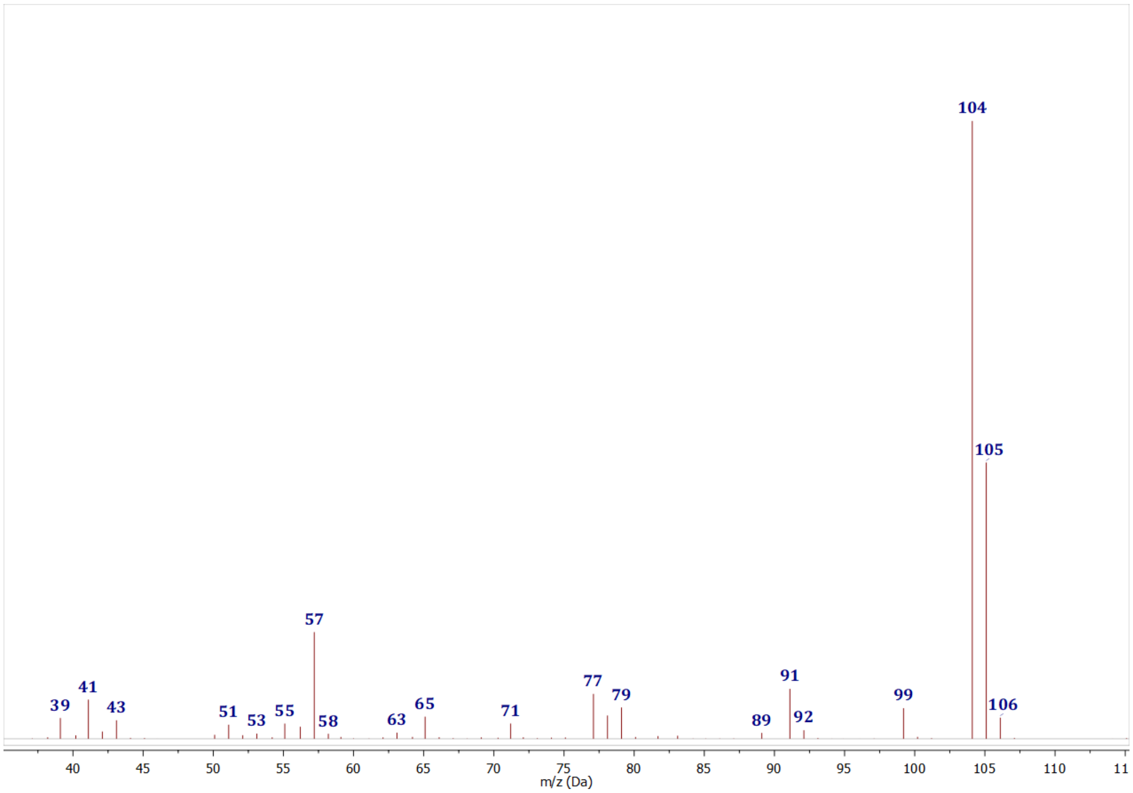

Figure S55. Mass spectrum of phenethyl 3,3-dimethylbutanoate (**3c**)

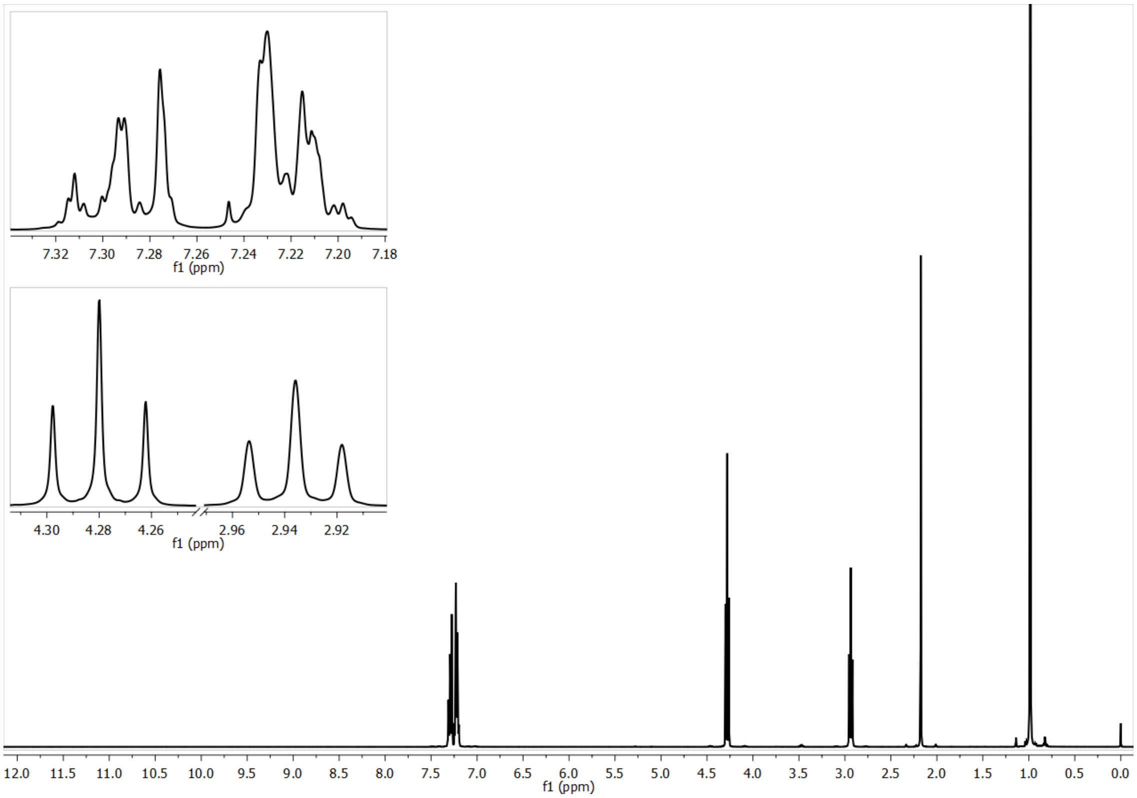

Figure S56. ^{1}H NMR spectrum of phenethyl 3,3-dimethylbutanoate (**3c**) recorded in $CDCl_3$

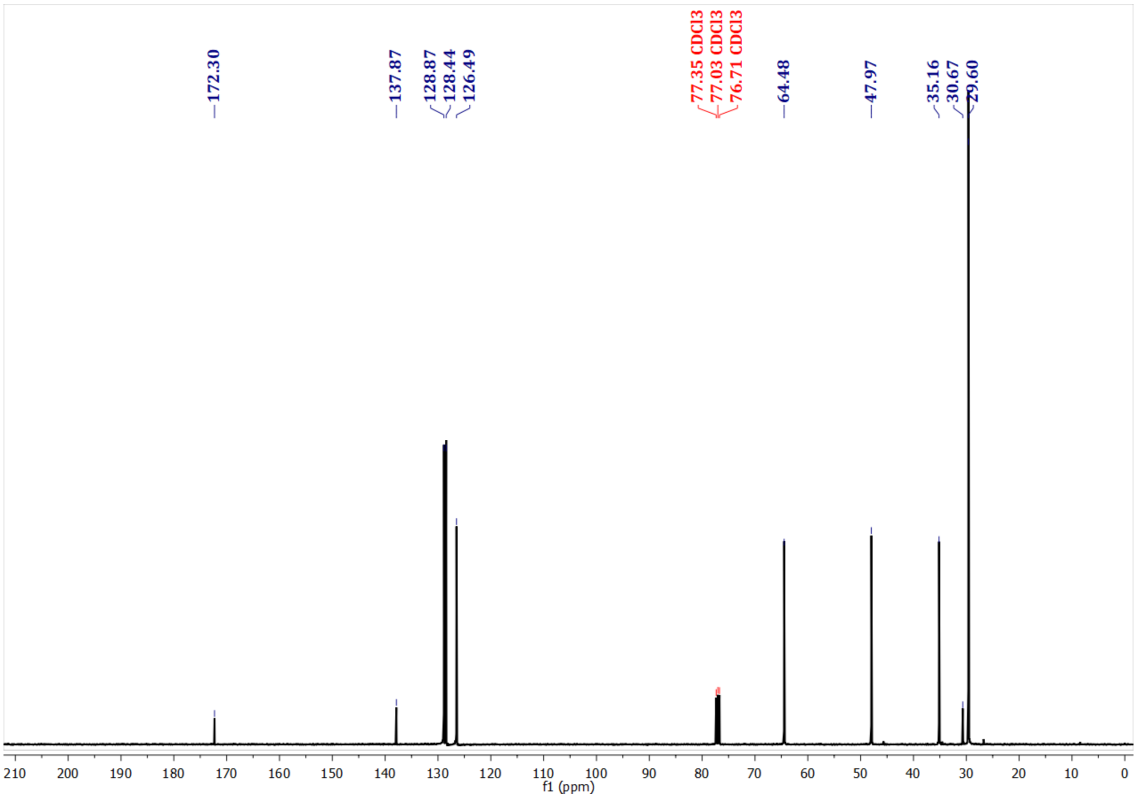

Figure S57. ^{13}C NMR spectrum of phenethyl 3,3-dimethylbutanoate (**3c**) recorded in $CDCl_3$

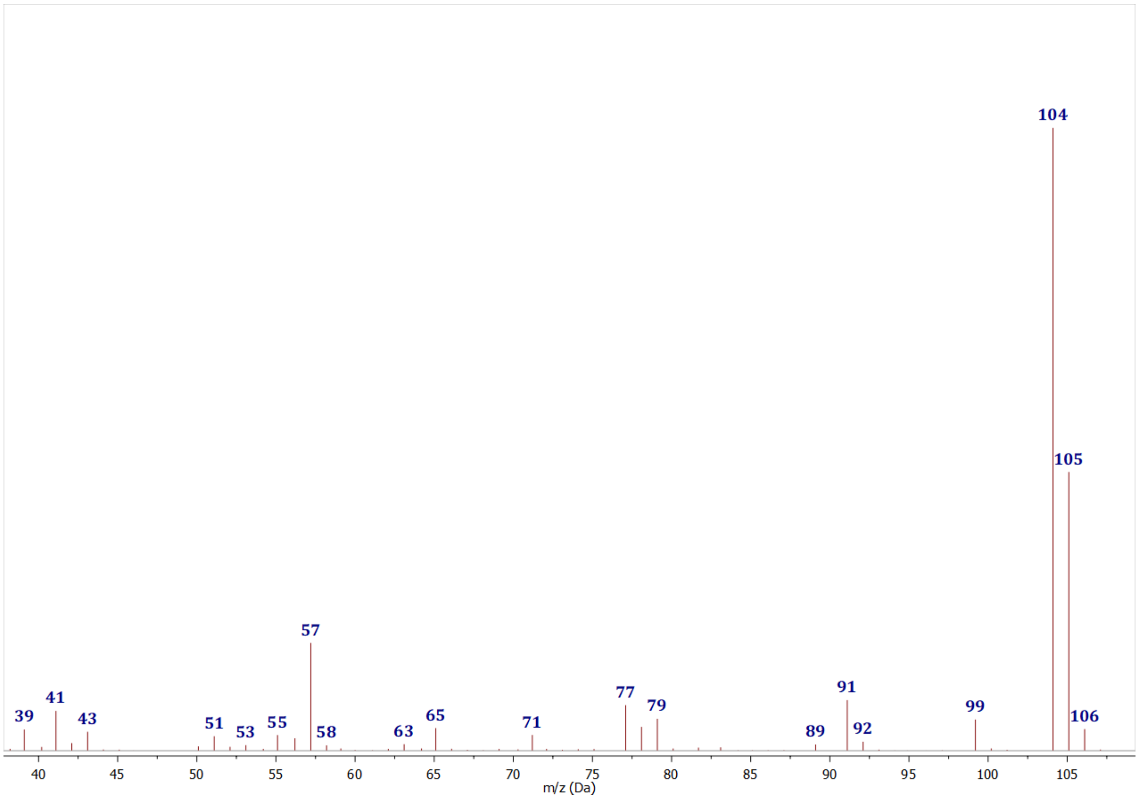

Figure S58. Mass spectrum of phenethyl 2,3-dimethylbutanoate (**3d**)

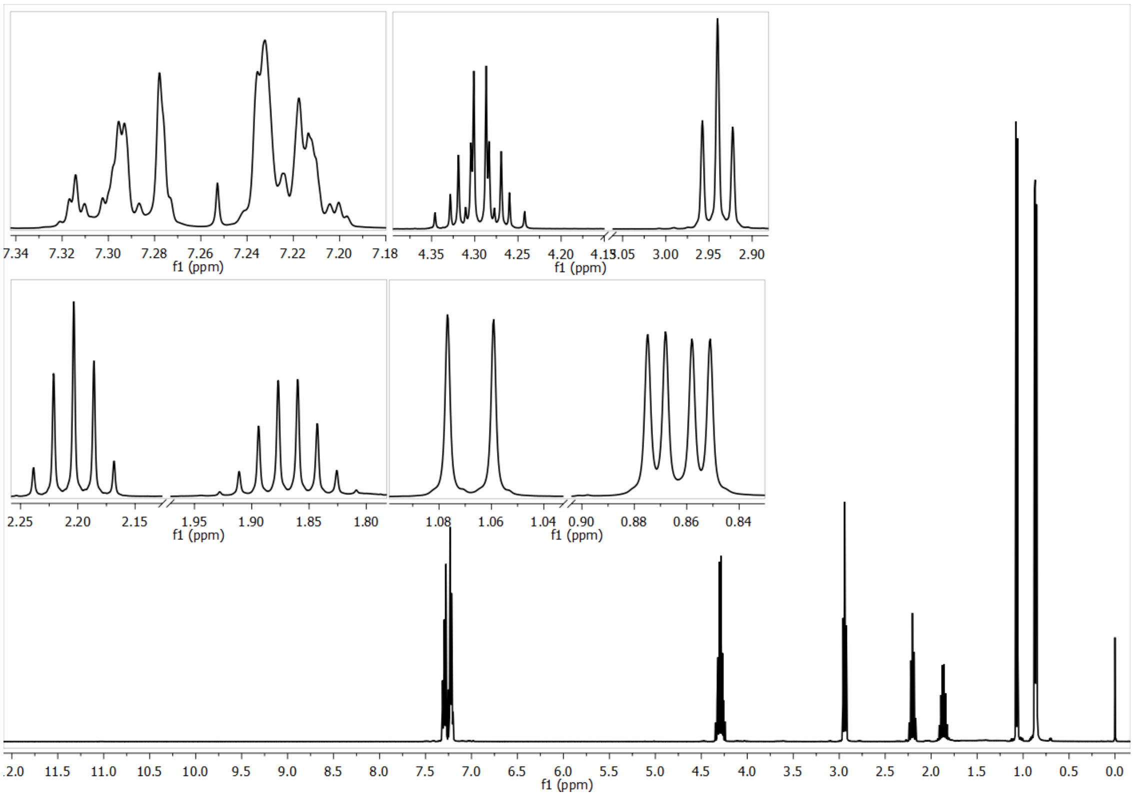

Figure S59. ^{1}H NMR spectrum of phenethyl 2,3-dimethylbutanoate (**3d**) recorded in $CDCl_3$

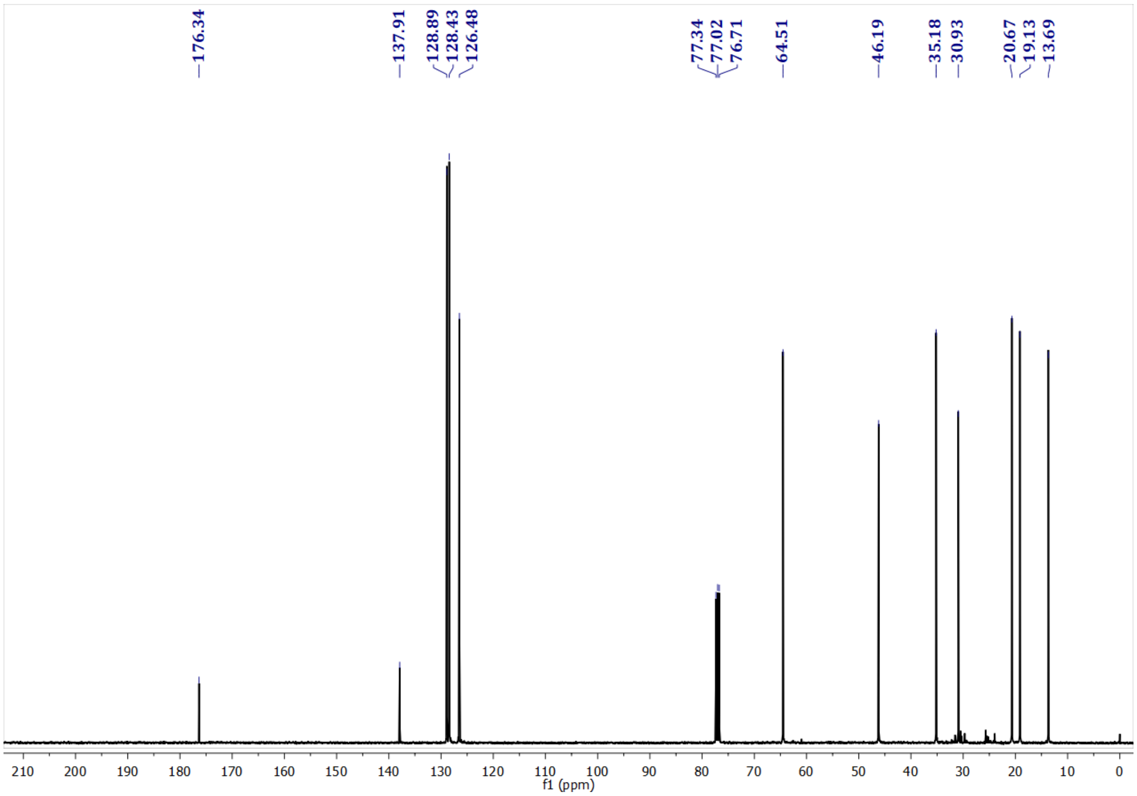

Figure S60. ^{13}C NMR spectrum of phenethyl 2,3-dimethylbutanoate (**3d**) recorded in $CDCl_3$

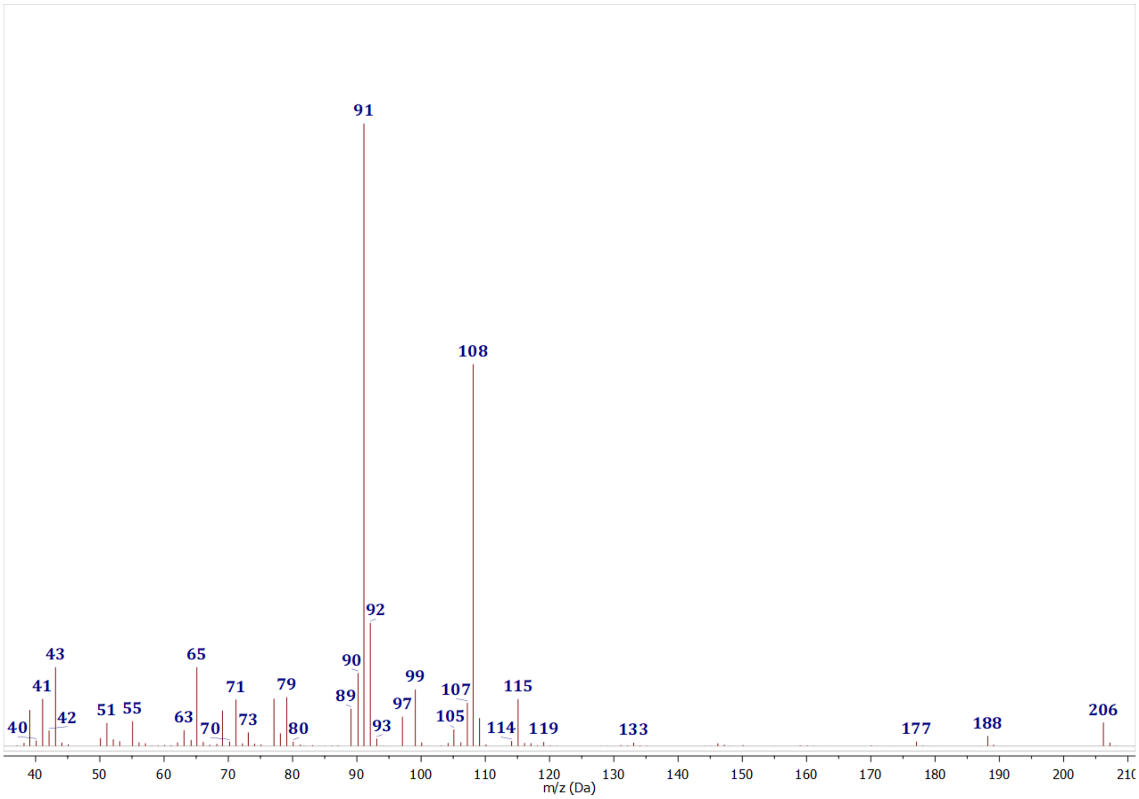

Figure S61. Mass spectrum of phenethyl 2-methylpentanoate (**3e**)

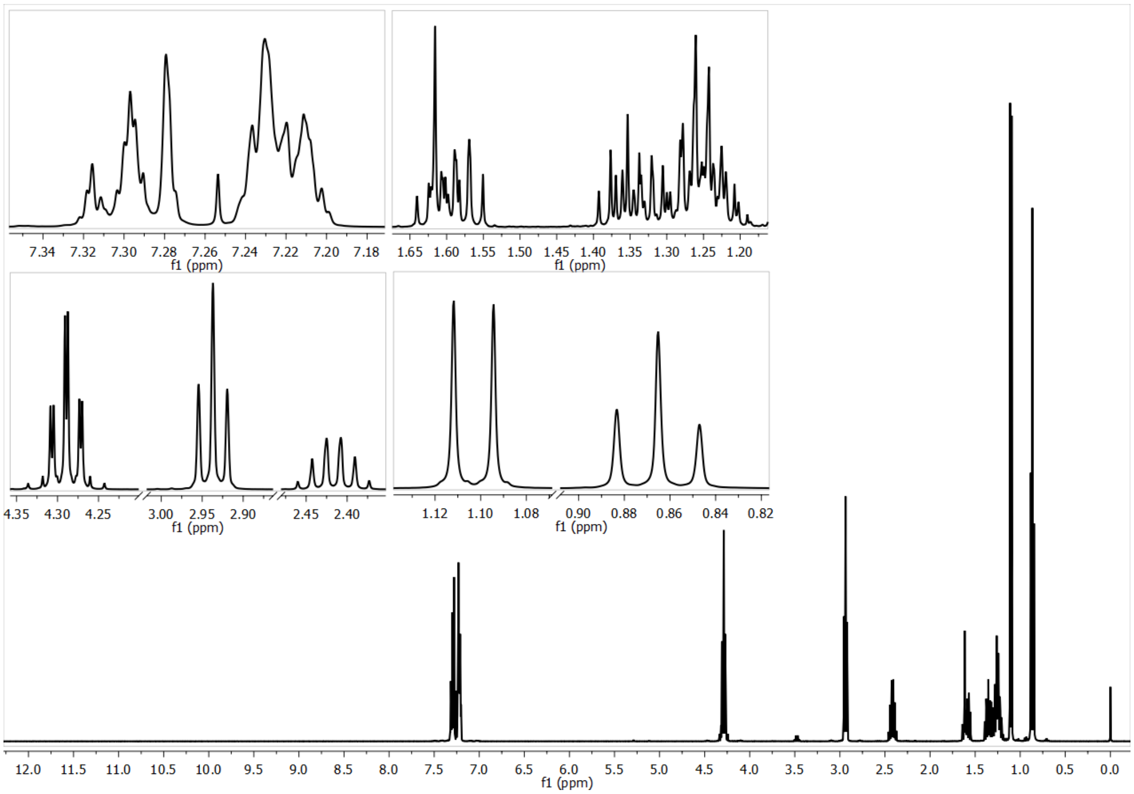

Figure S62. ^{1}H NMR spectrum of phenethyl 2-methylpentanoate (**3e**) recorded in $CDCl_3$

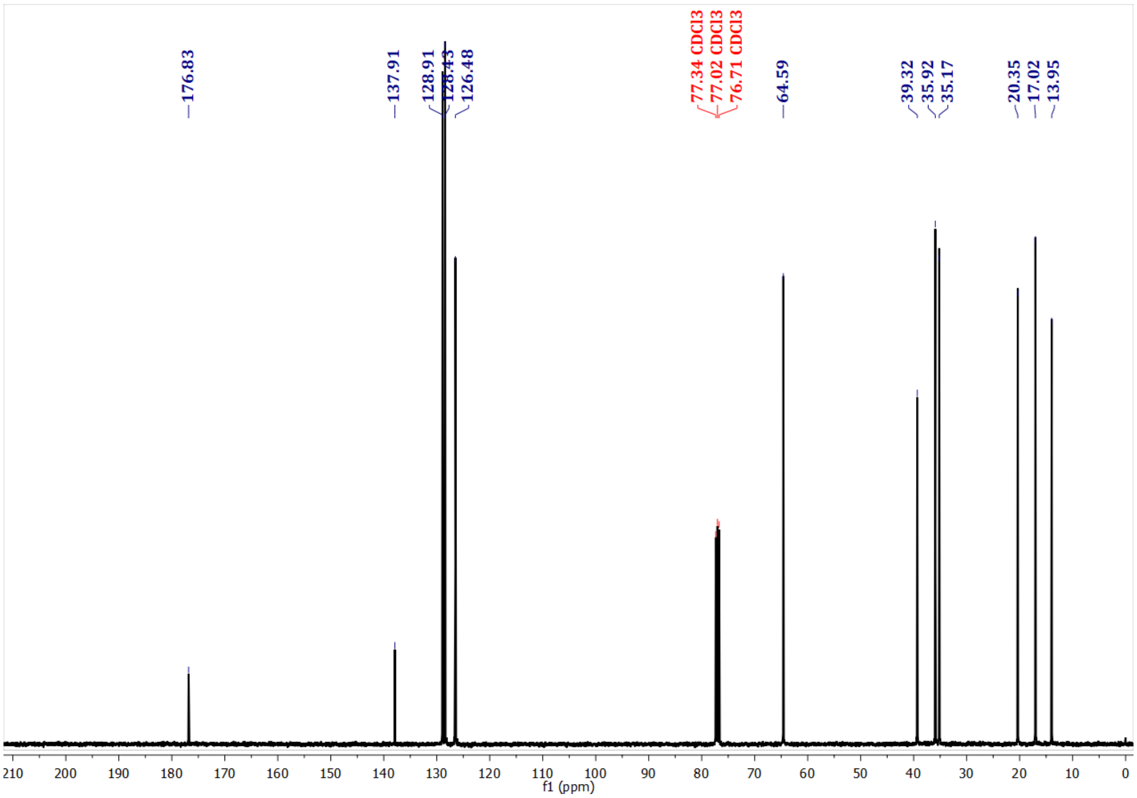

Figure S63. ^{13}C NMR spectrum of phenethyl 2-methylpentanoate (**3e**) recorded in $CDCl_3$

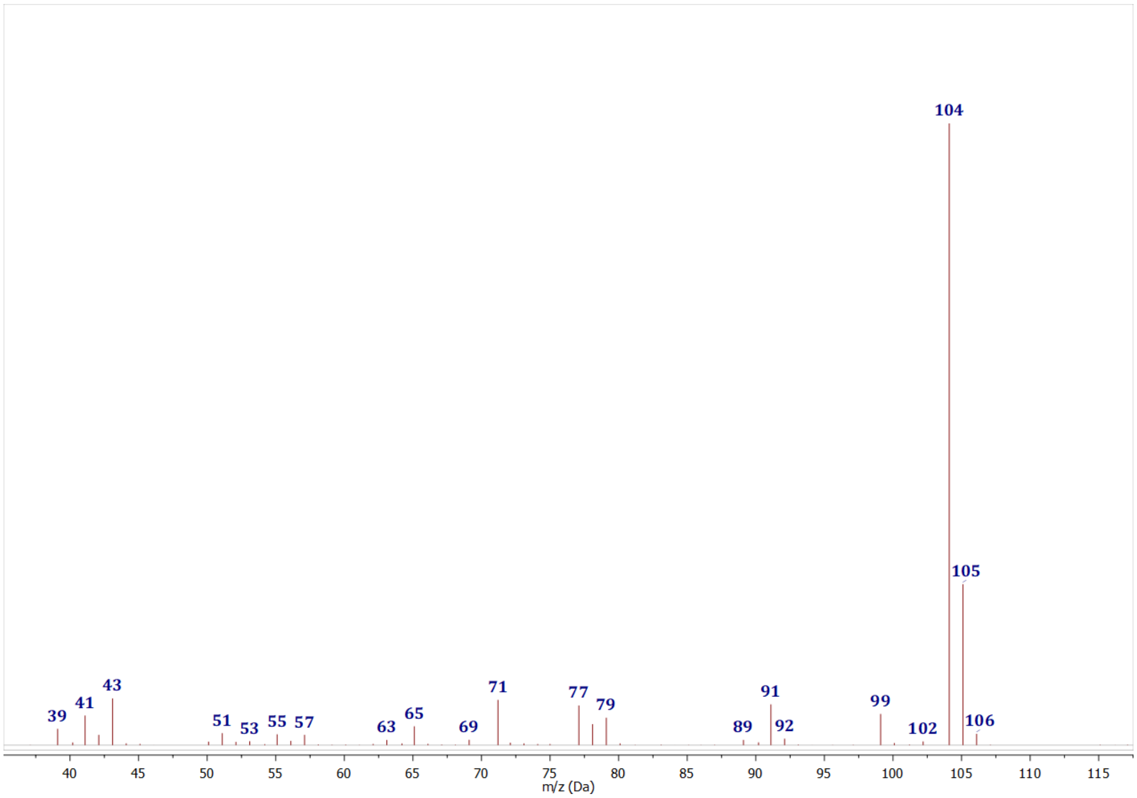

Figure S64. Mass spectrum of phenethyl 3-methylpentanoate (**3f**)

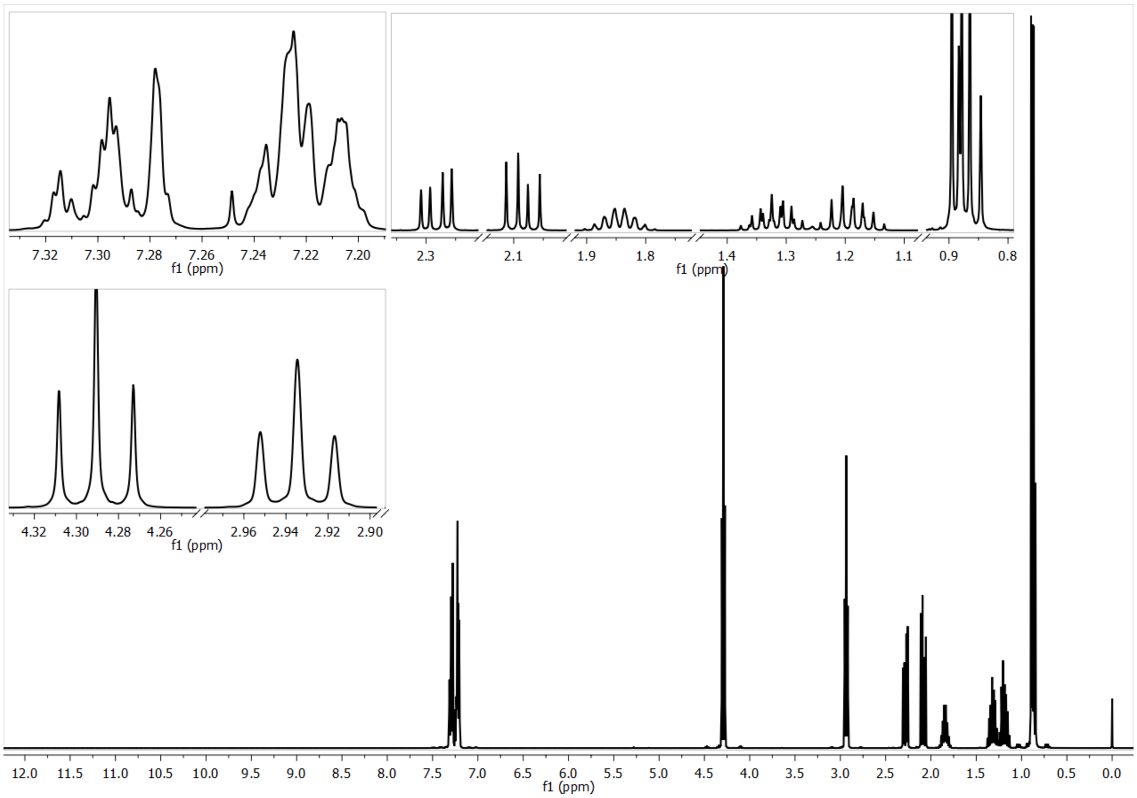

Figure S65. ^{1}H NMR spectrum of phenethyl 3-methylpentanoate (**3f**) recorded in $CDCl_3$

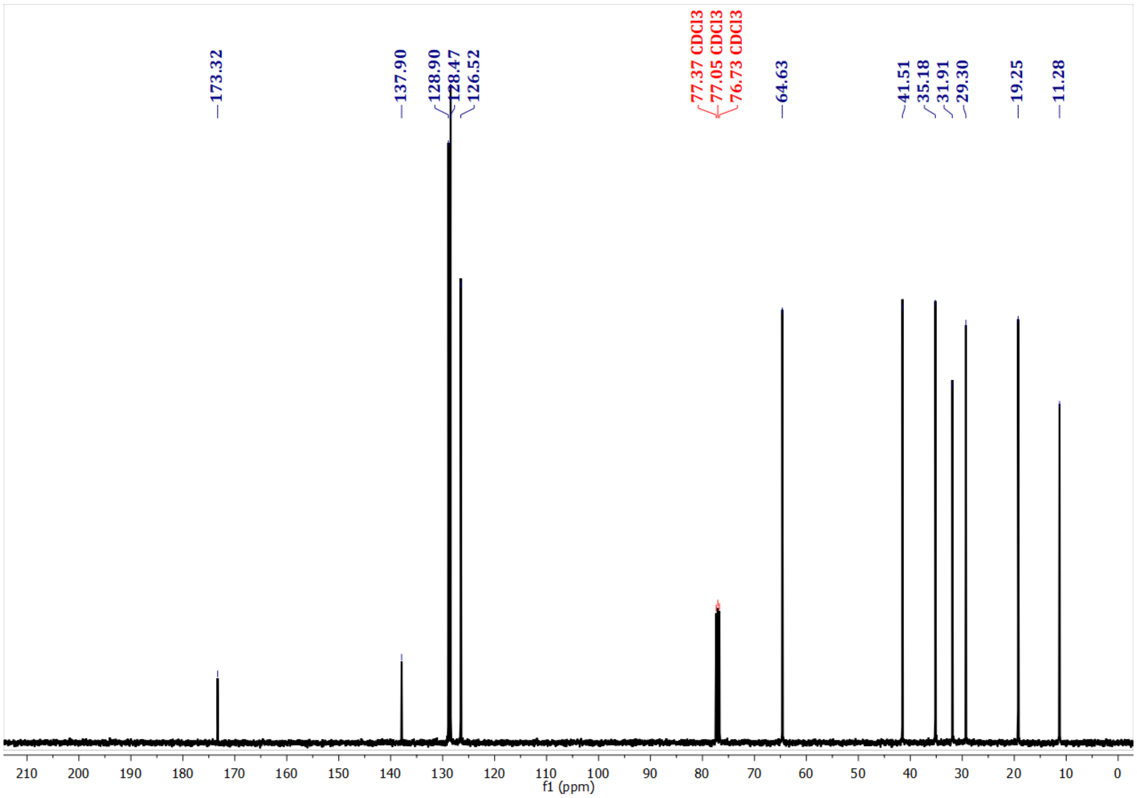

Figure S66. ^{13}C NMR spectrum of phenethyl 3-methylpentanoate (**3f**) recorded in $CDCl_3$

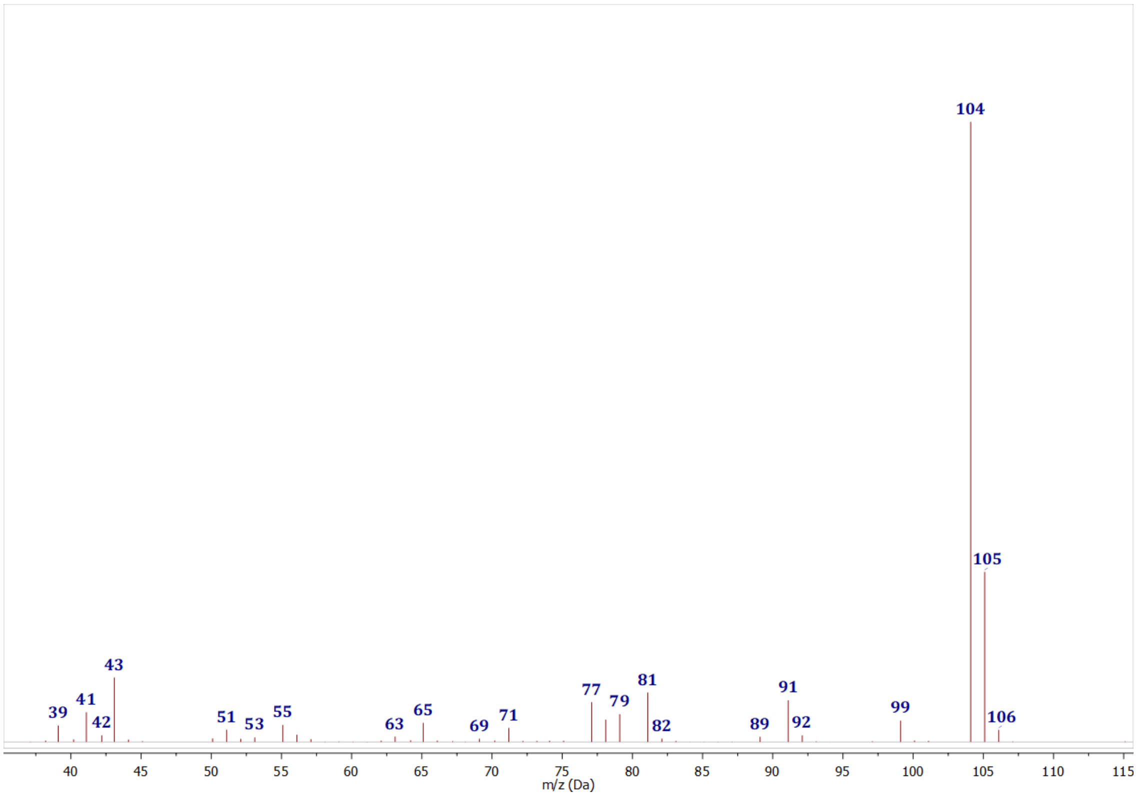

Figure S67. Mass spectrum of phenethyl 4-methylpentanoate (**3g**)

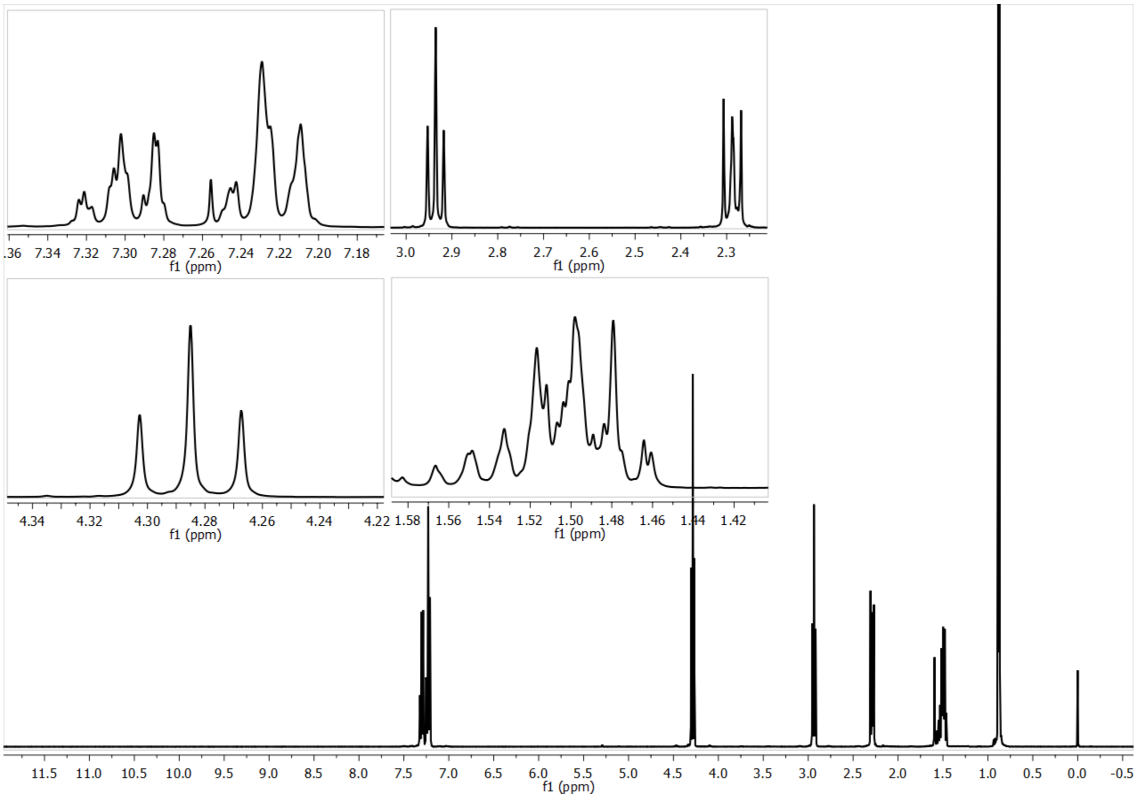

Figure S68. ^{1}H NMR spectrum of phenethyl 4-methylpentanoate (**3g**) recorded in $CDCl_3$

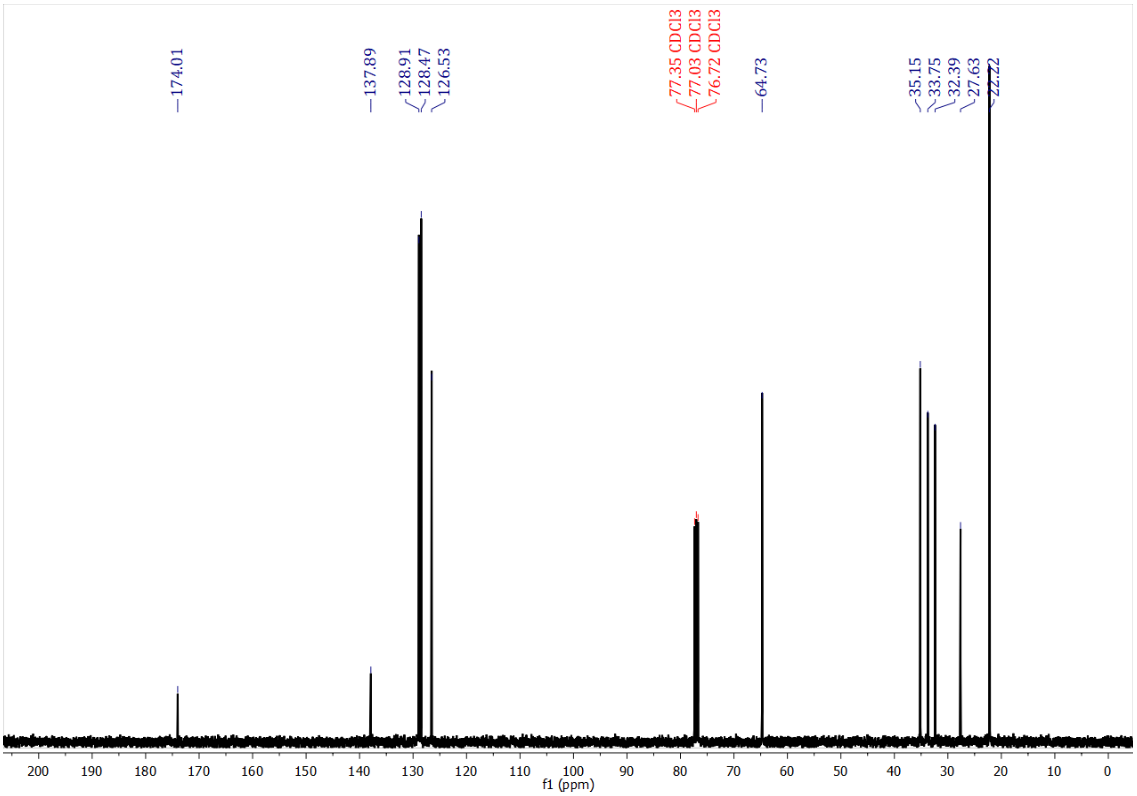

Figure S69. ^{13}C NMR spectrum of phenethyl 4-methylpentanoate (**3g**) recorded in $CDCl_3$

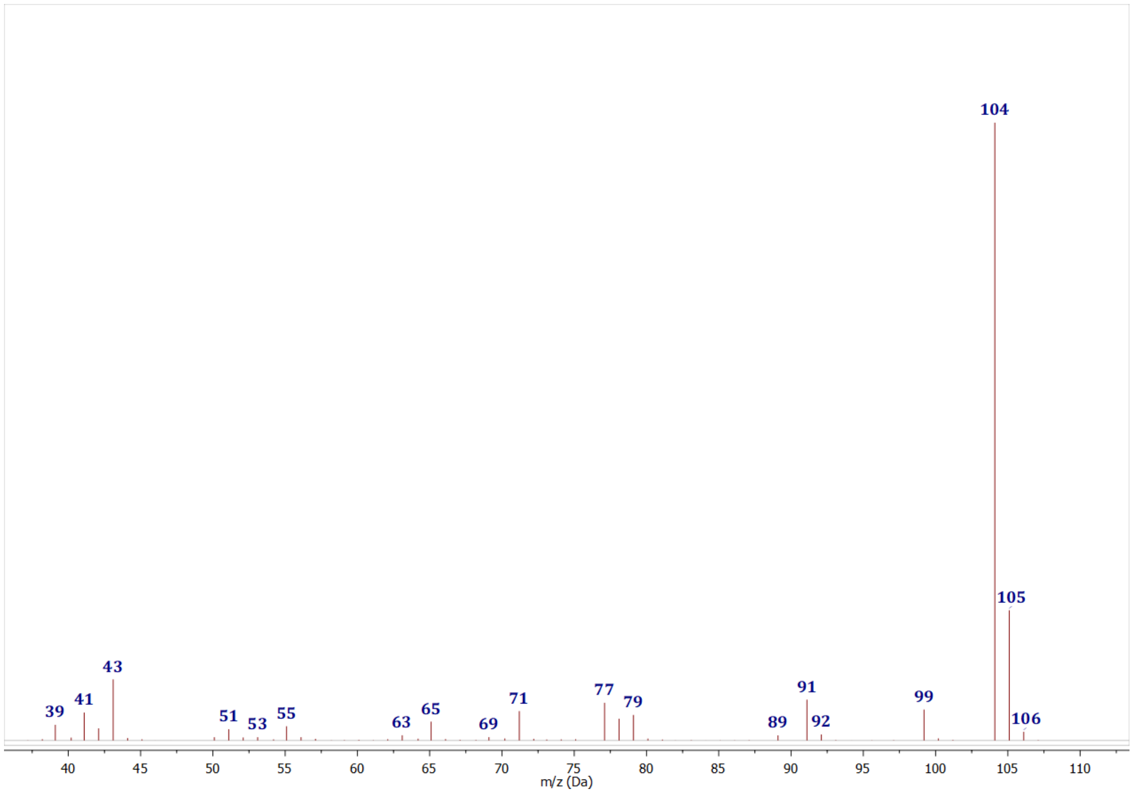

Figure S70. Mass spectrum of phenethyl hexanoate (**3h**)

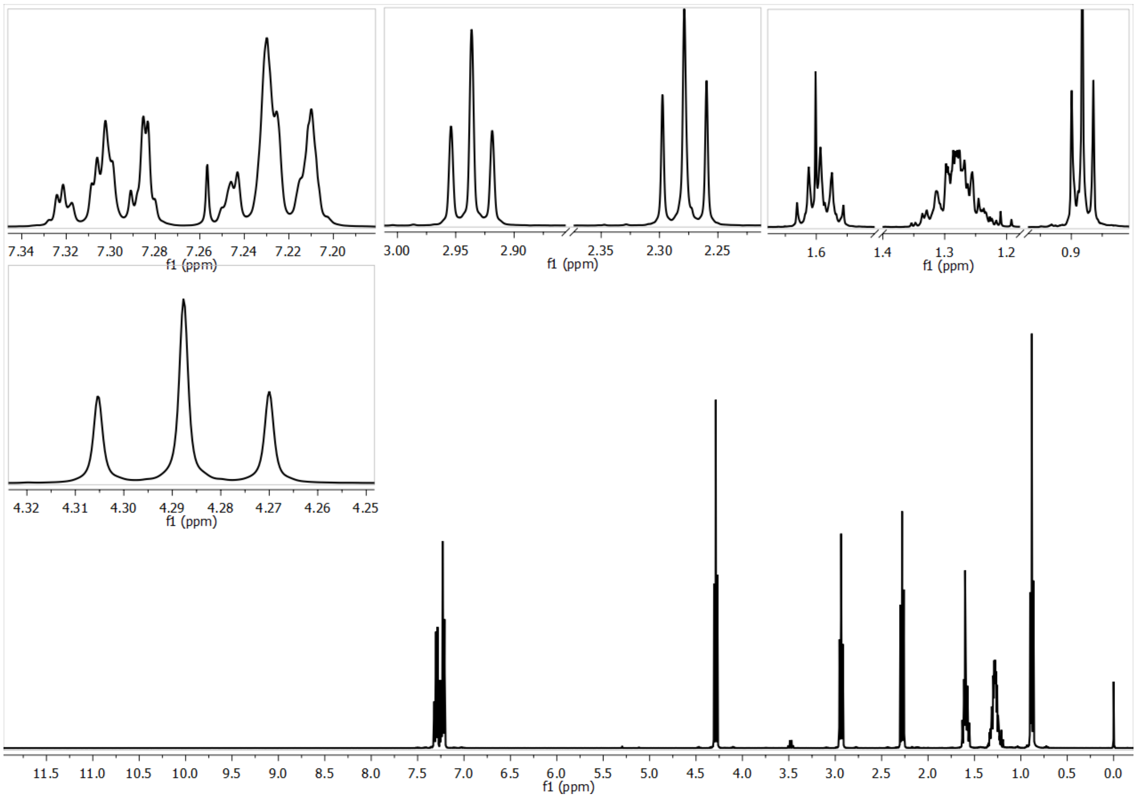

Figure S71. ^{1}H NMR spectrum of phenethyl hexanoate (**3h**) recorded in $CDCl_3$

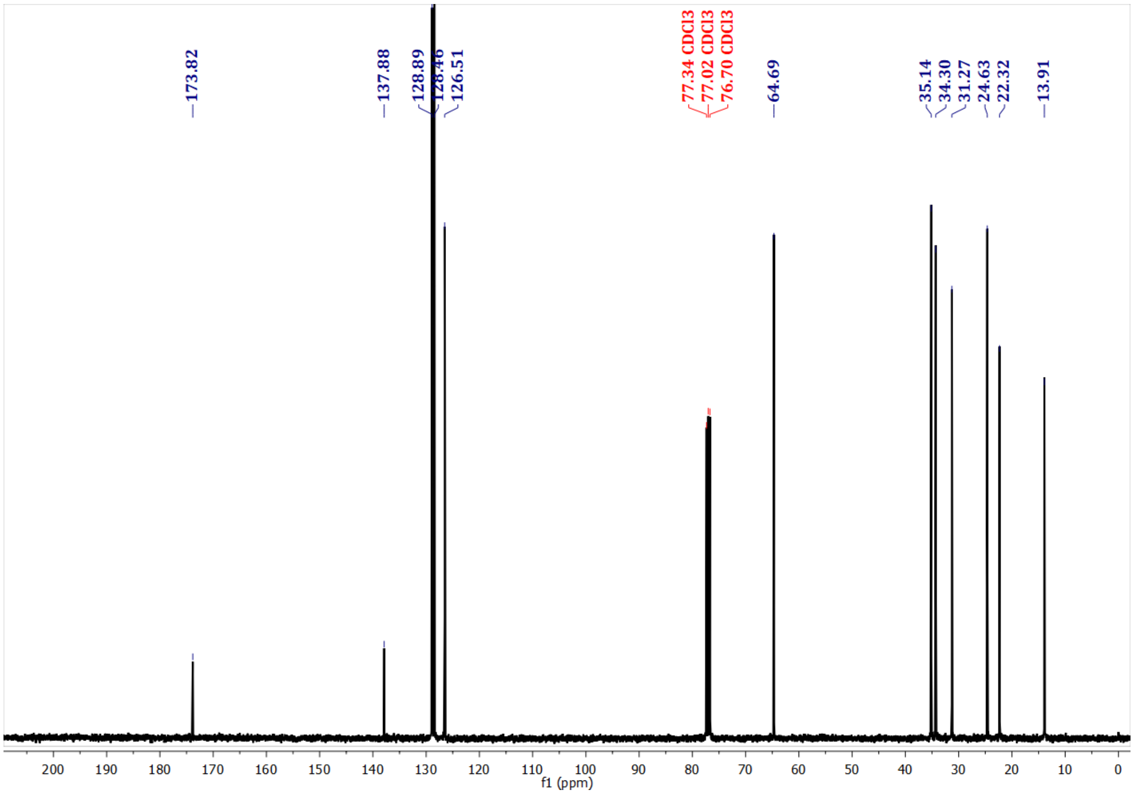

Figure S72. ^{13}C NMR spectrum of phenethyl hexanoate (**3h**) recorded in $CDCl_3$

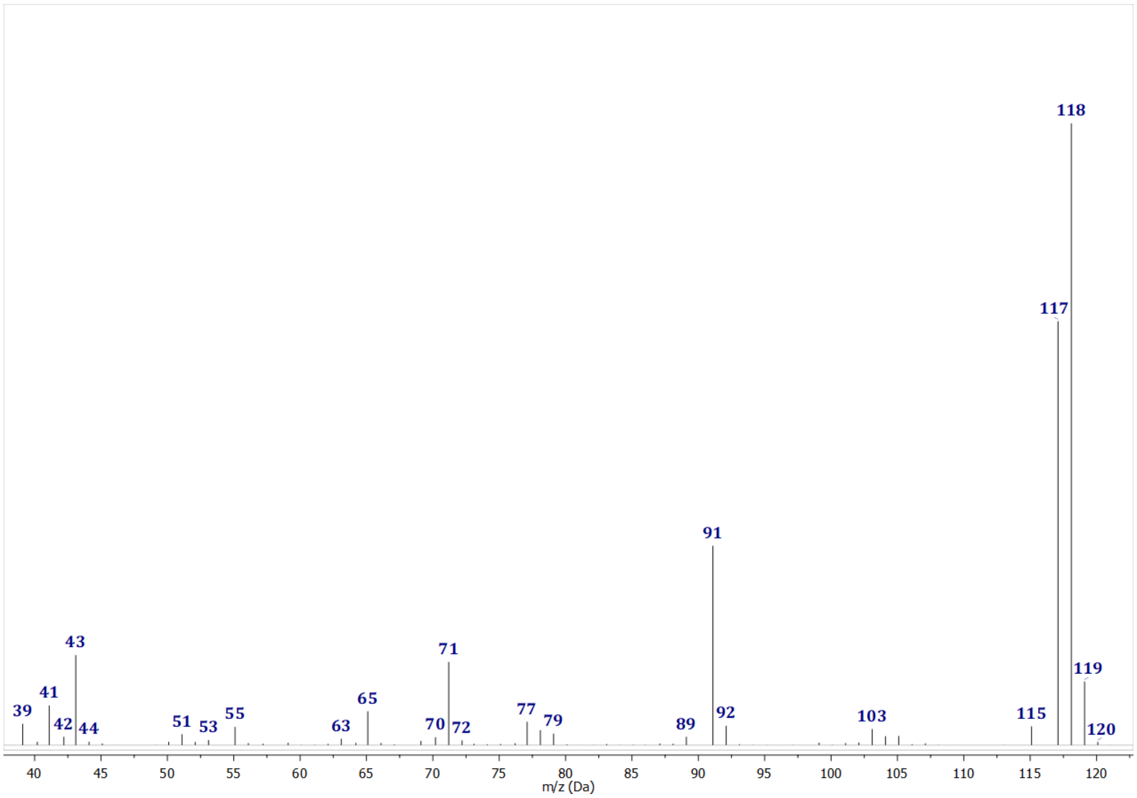

Figure S73. Mass spectrum of 3-phenylpropyl 2-ethylbutanoate (**4a**)

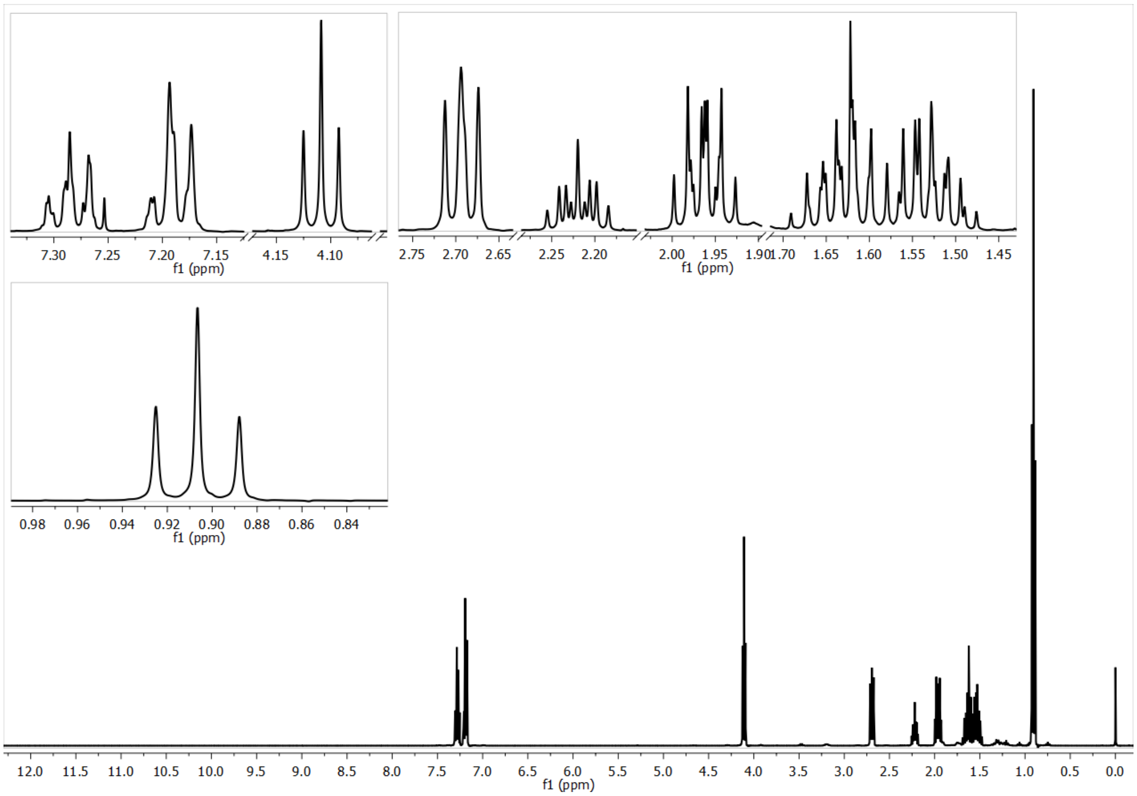

Figure S74. ^{1}H NMR spectrum of 3-phenylpropyl 2-ethylbutanoate (**4a**) recorded in $CDCl_3$

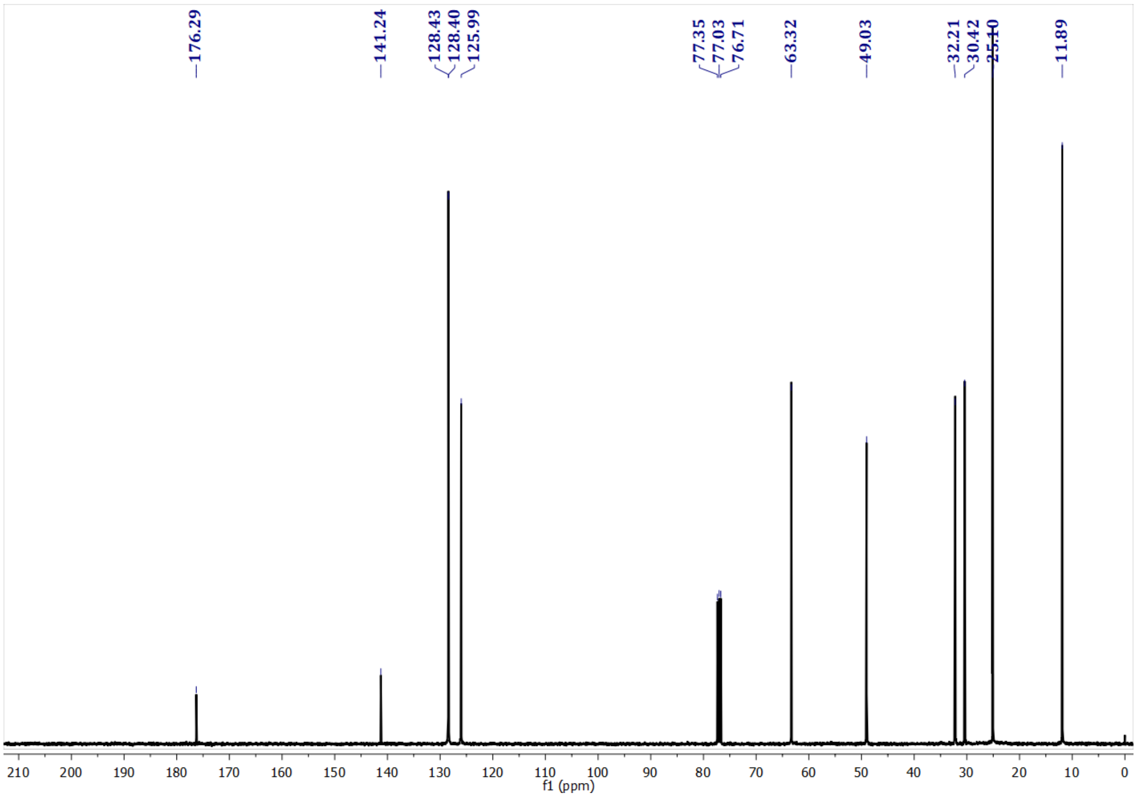

Figure S75. ^{13}C NMR spectrum of 3-phenylpropyl 2-ethylbutanoate (**4a**) recorded in $CDCl_3$

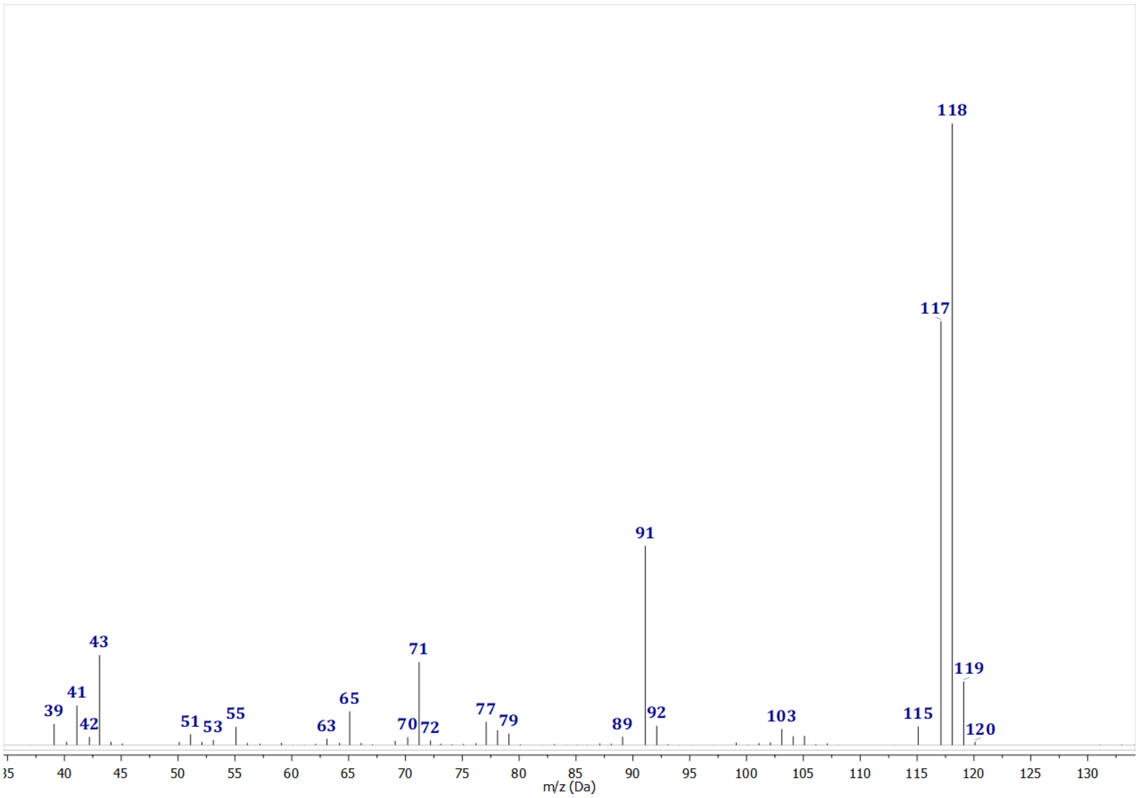

Figure S76. Mass spectrum of 3-phenylpropyl 2,2-dimethylbutanoate (**4b**)

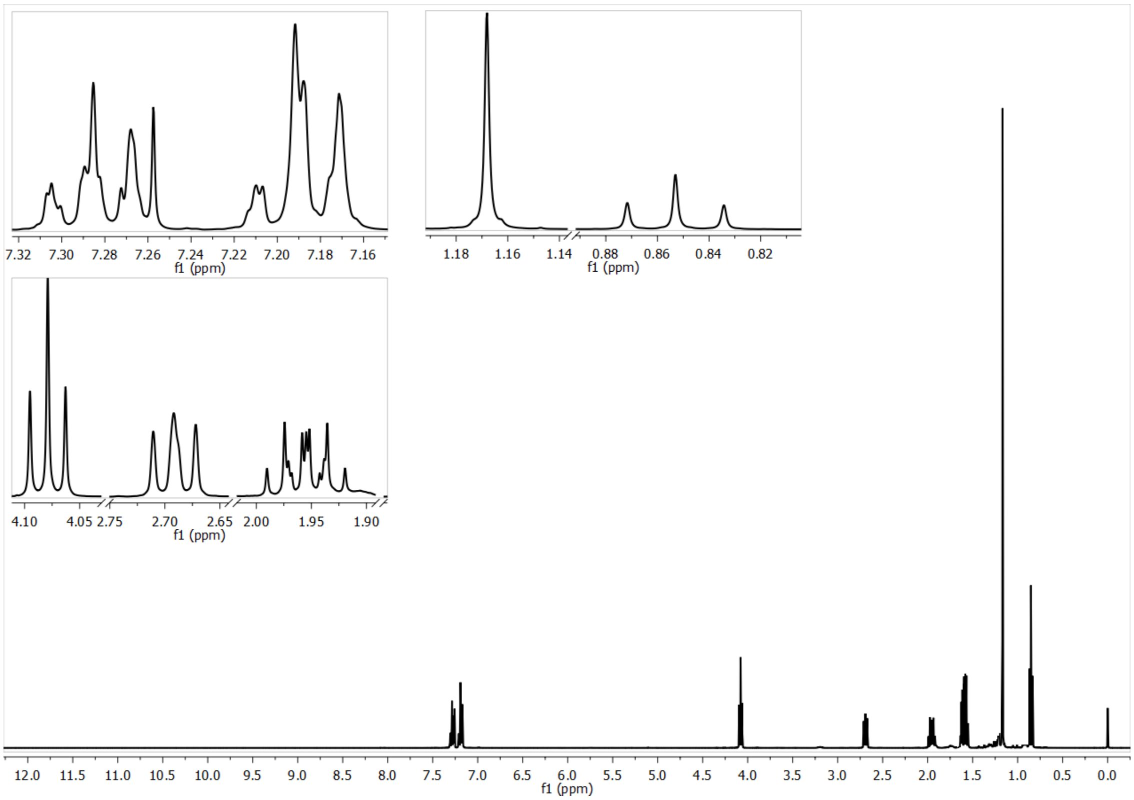

Figure S77. ^{1}H NMR spectrum of 3-phenylpropyl 2,2-dimethylbutanoate (**4b**) recorded in $CDCl_3$

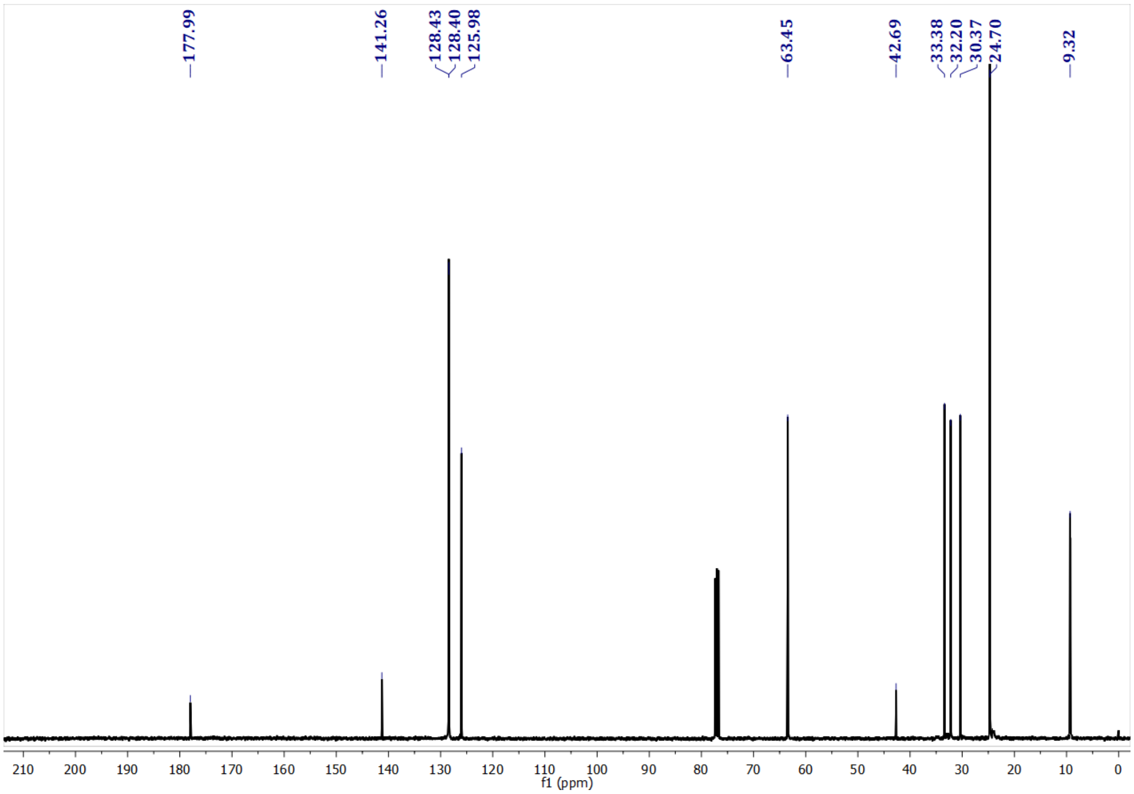

Figure S78. ^{13}C NMR spectrum of 3-phenylpropyl 2,2-dimethylbutanoate (**4b**) recorded in $CDCl_3$

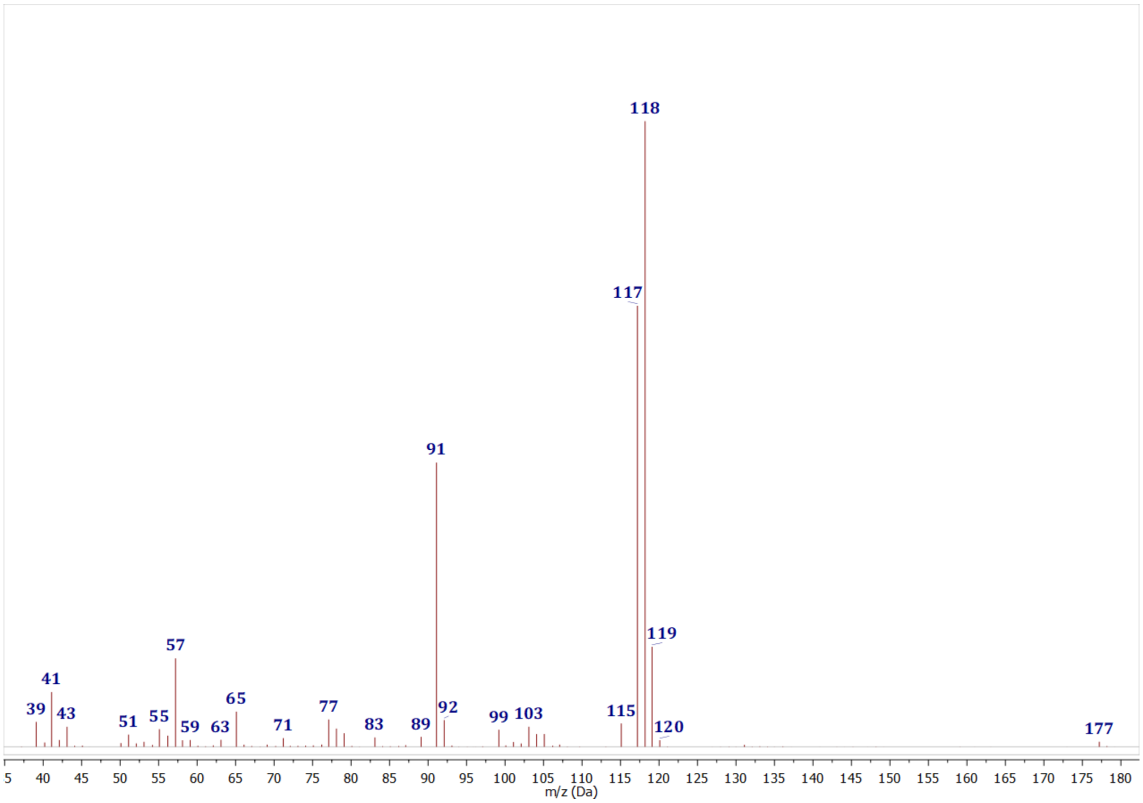

Figure S79. Mass spectrum of 3-phenylpropyl 3,3-dimethylbutanoate (**4c**)

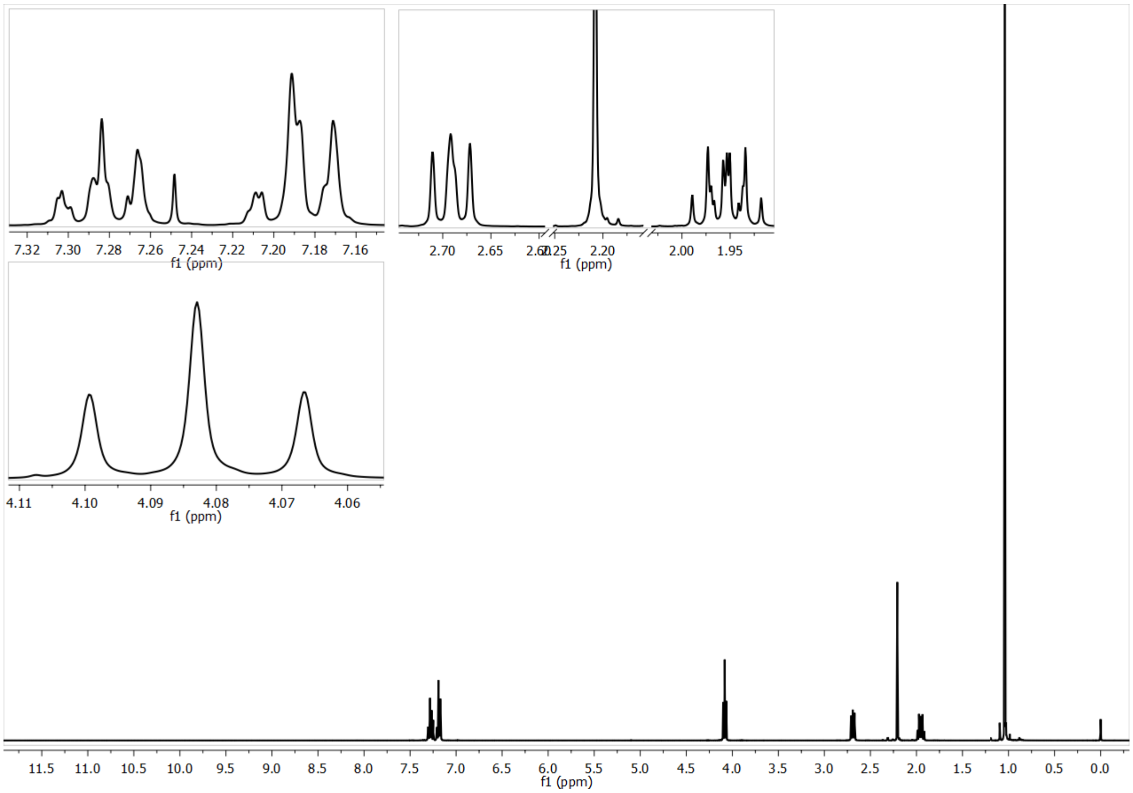

Figure S80. ^{1}H NMR spectrum of 3-phenylpropyl 3,3-dimethylbutanoate (**4c**) recorded in $CDCl_3$

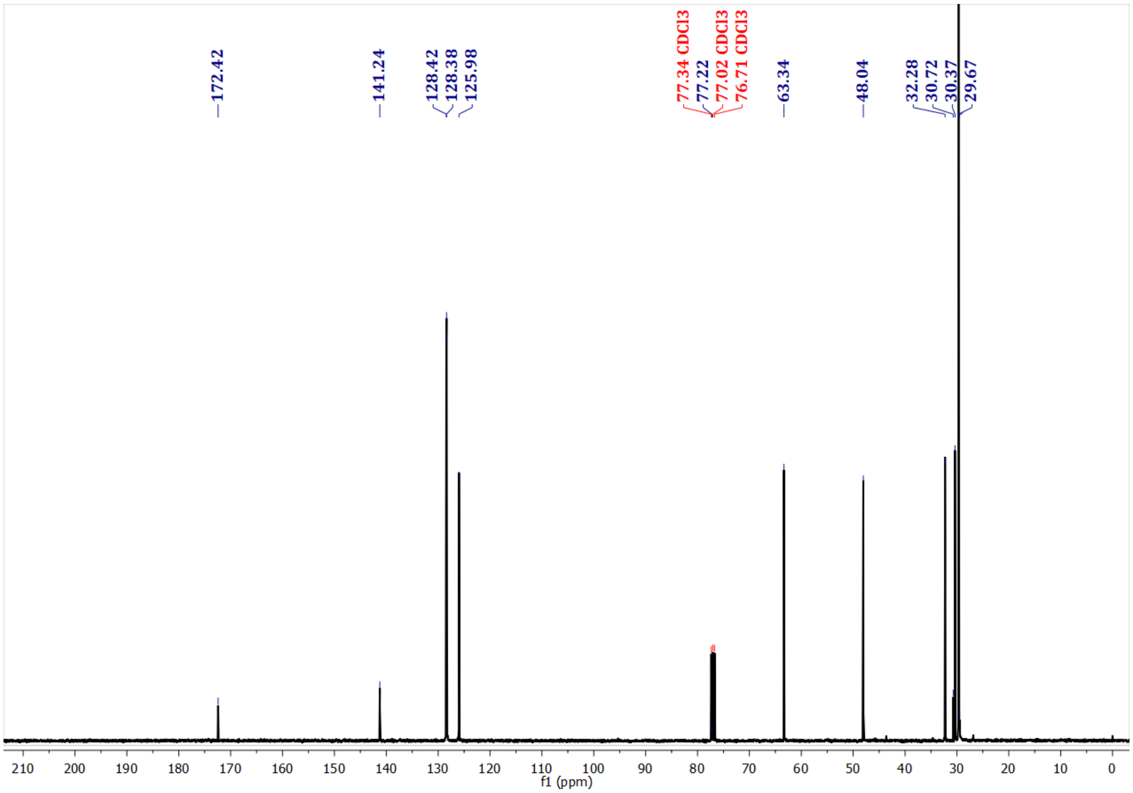

Figure S81. ^{13}C NMR spectrum of 3-phenylpropyl 3,3-dimethylbutanoate (**4c**) recorded in $CDCl_3$

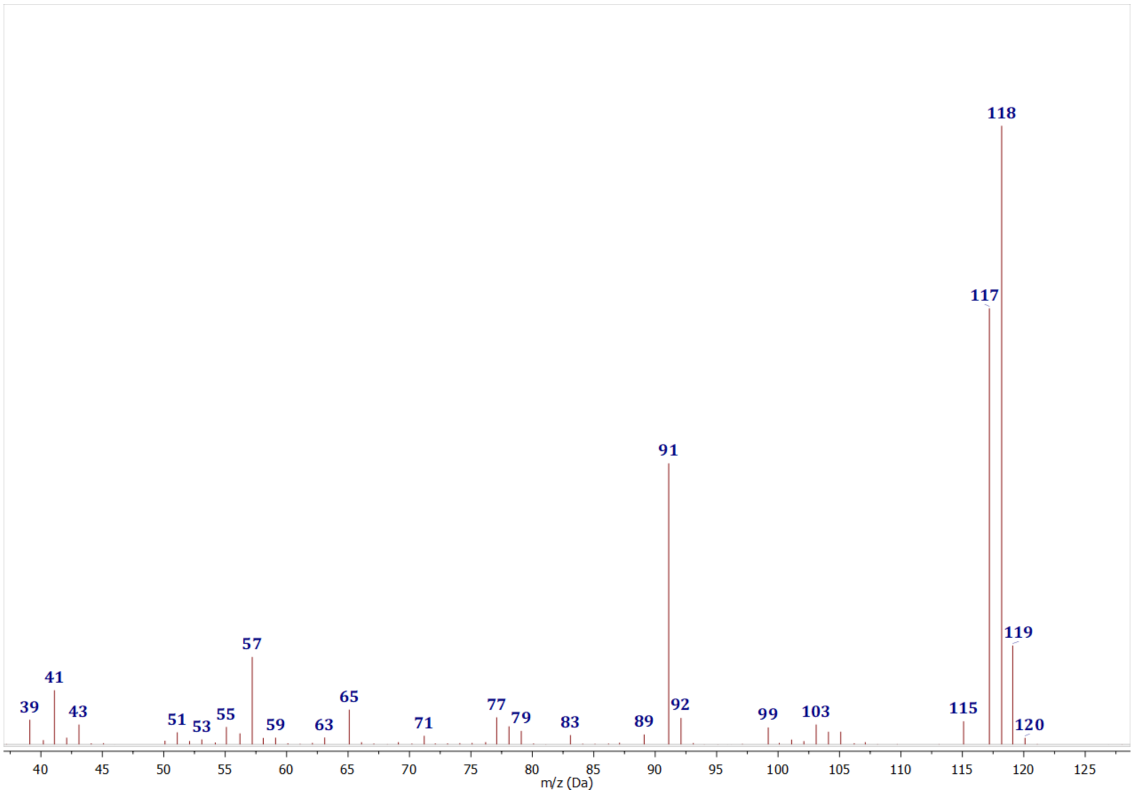

Figure S82. Mass spectrum of 3-phenylpropyl 2,3-dimethylbutanoate (**4d**)

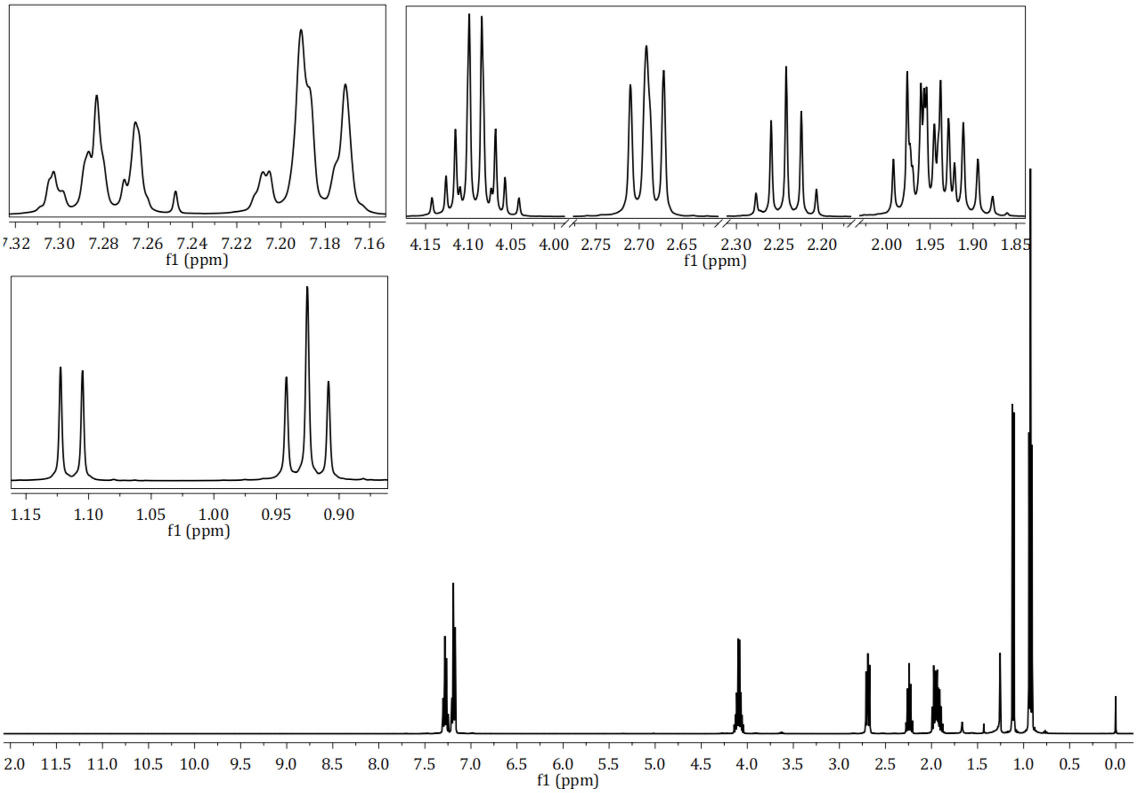

Figure S83. ^{1}H NMR spectrum of 3-phenylpropyl 2,3-dimethylbutanoate (**4d**) recorded in $CDCl_3$

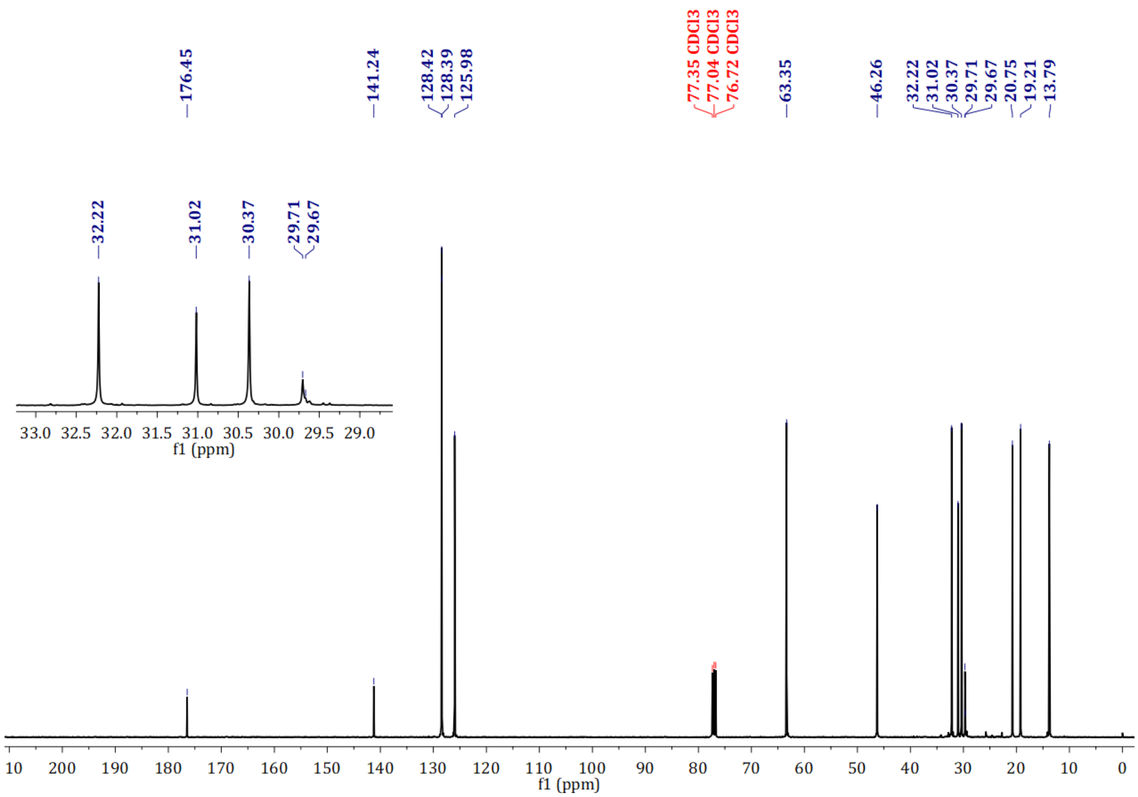

Figure S84. ^{13}C NMR spectrum of 3-phenylpropyl 2,3-dimethylbutanoate (**4d**) recorded in $CDCl_3$

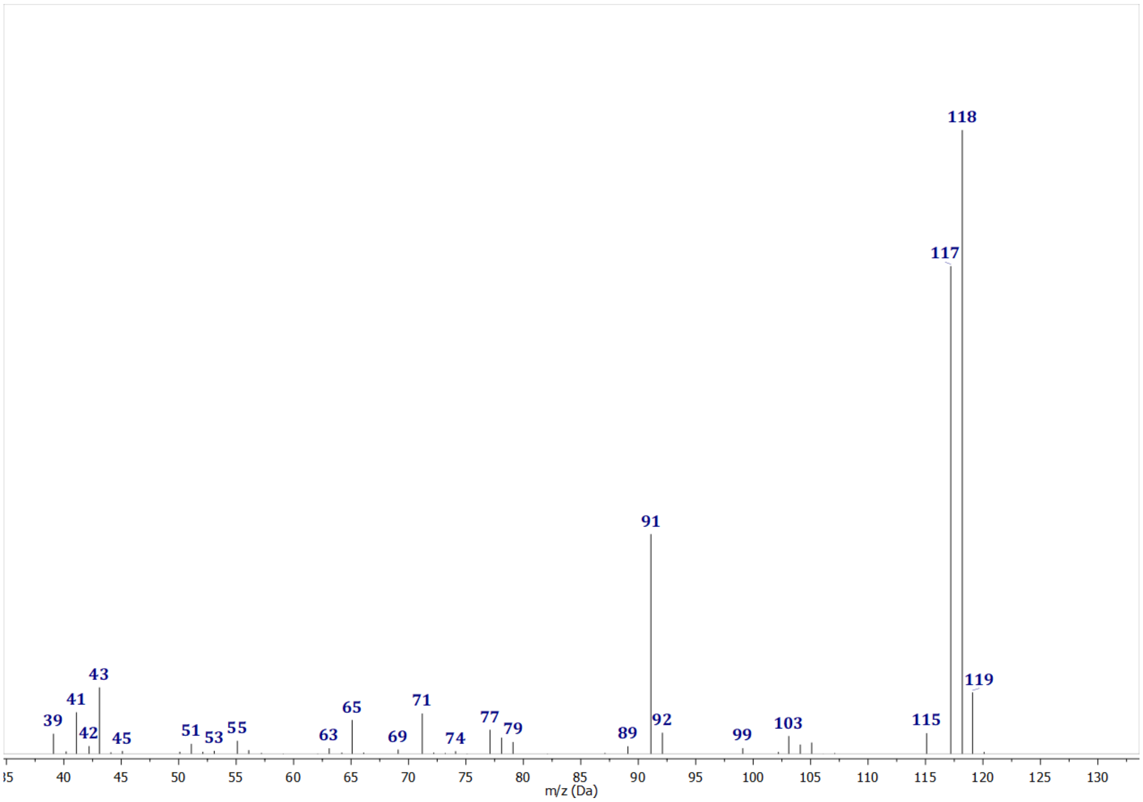

Figure S85. Mass spectrum of 3-phenylpropyl 2-methylpentanoate (**4e**)

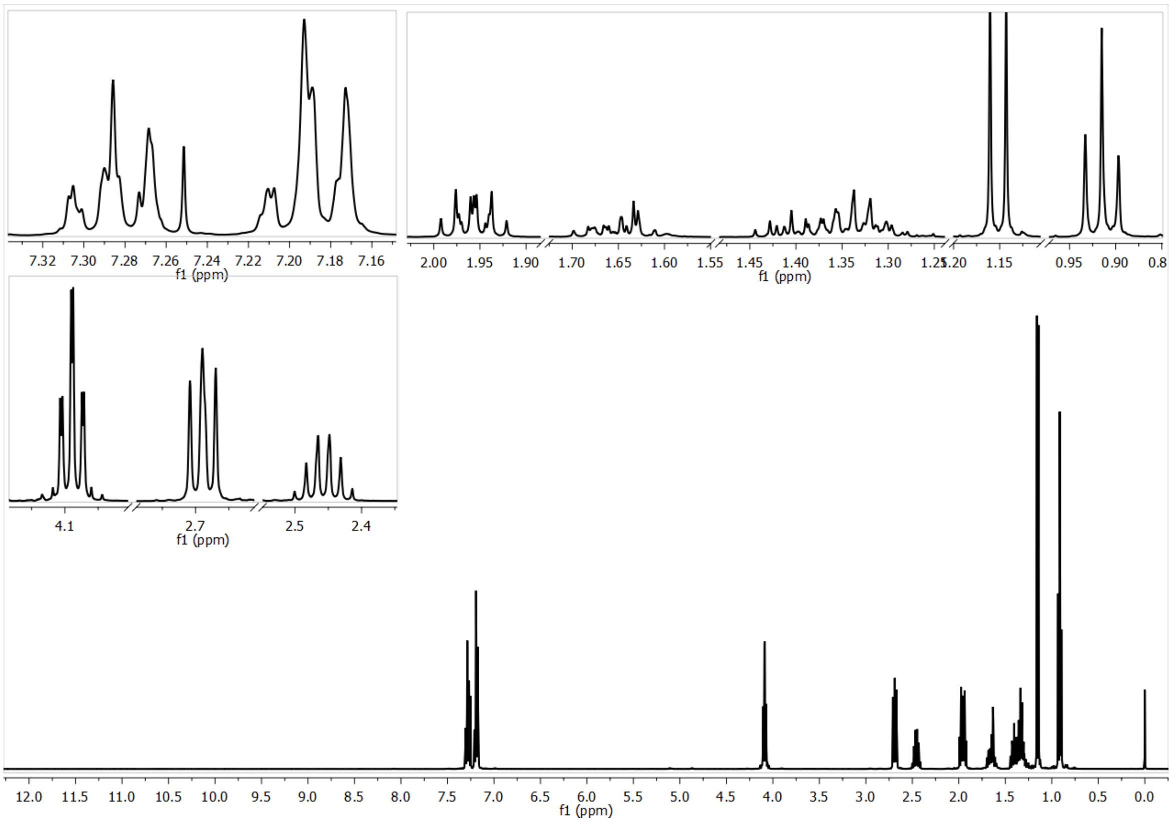

Figure S86a. ¹H NMR spectrum of 3-phenylpropyl 2-methylpentanoate (**4e**) recorded in $CDCl_3$

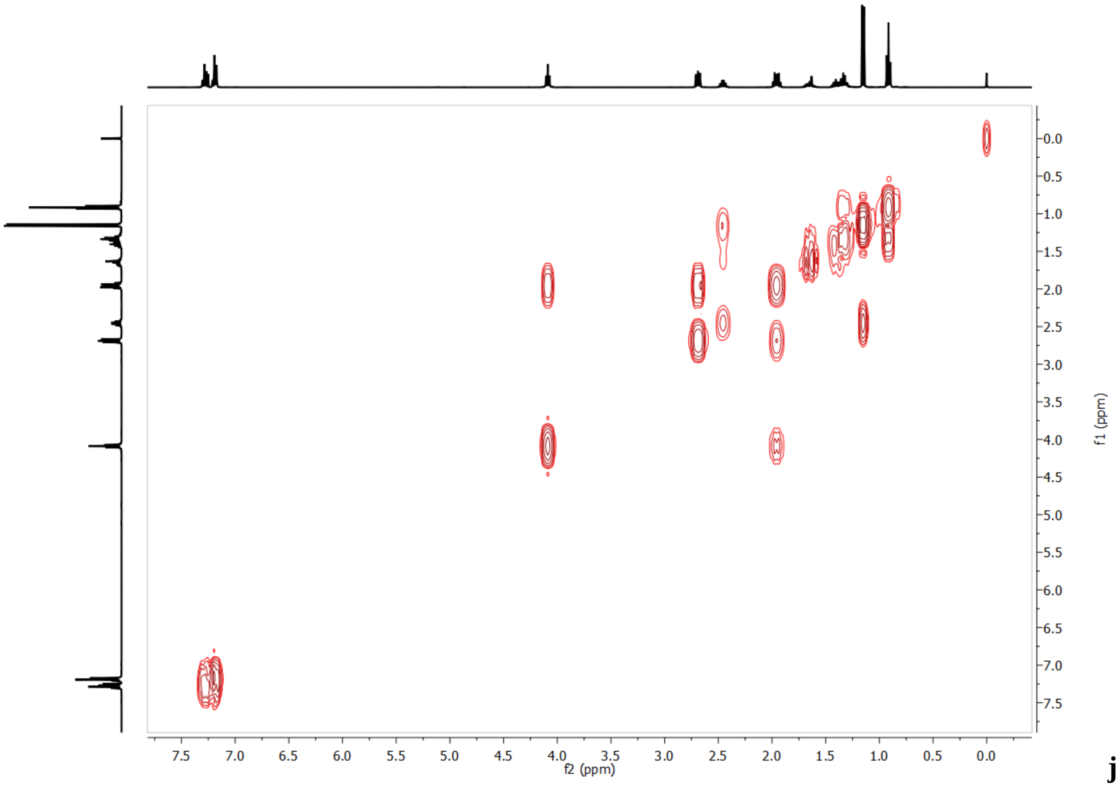

j

Figure S86b. ¹H – ¹H COSY spectrum of 3-phenylpropyl 2-methylpentanoate (**4e**)

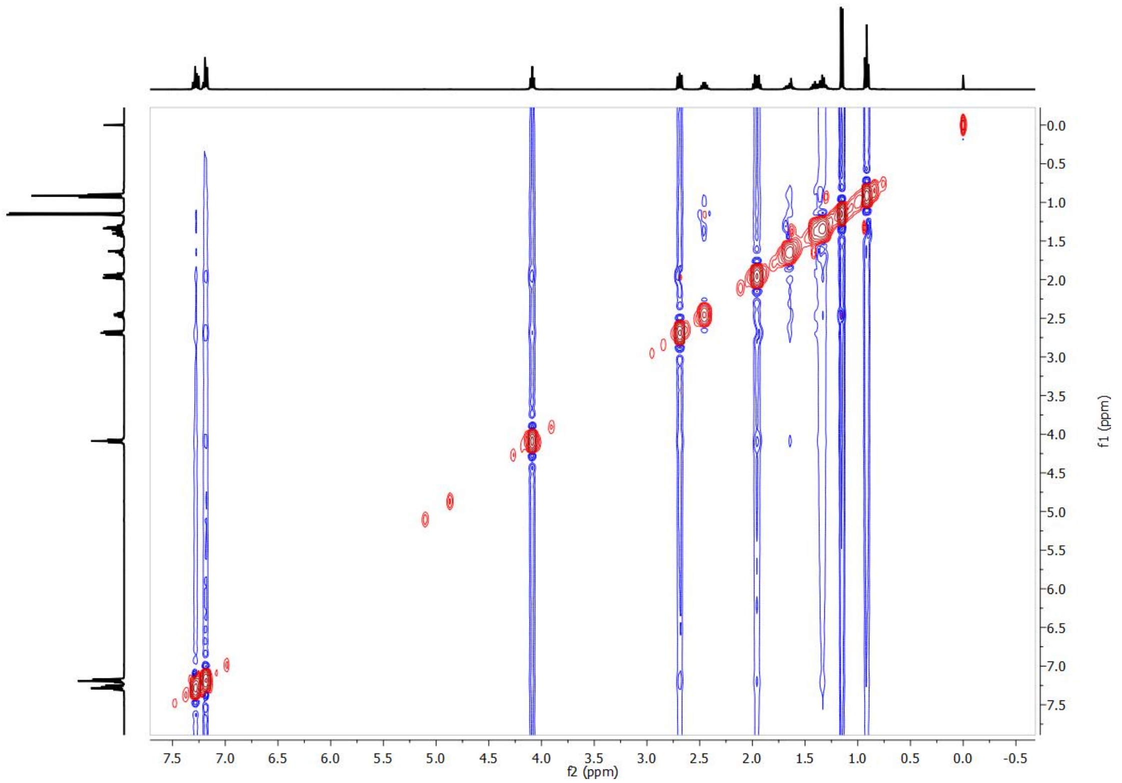

Figure S86c. NOESY spectrum of 3-phenylpropyl 2-methylpentanoate (**4e**)

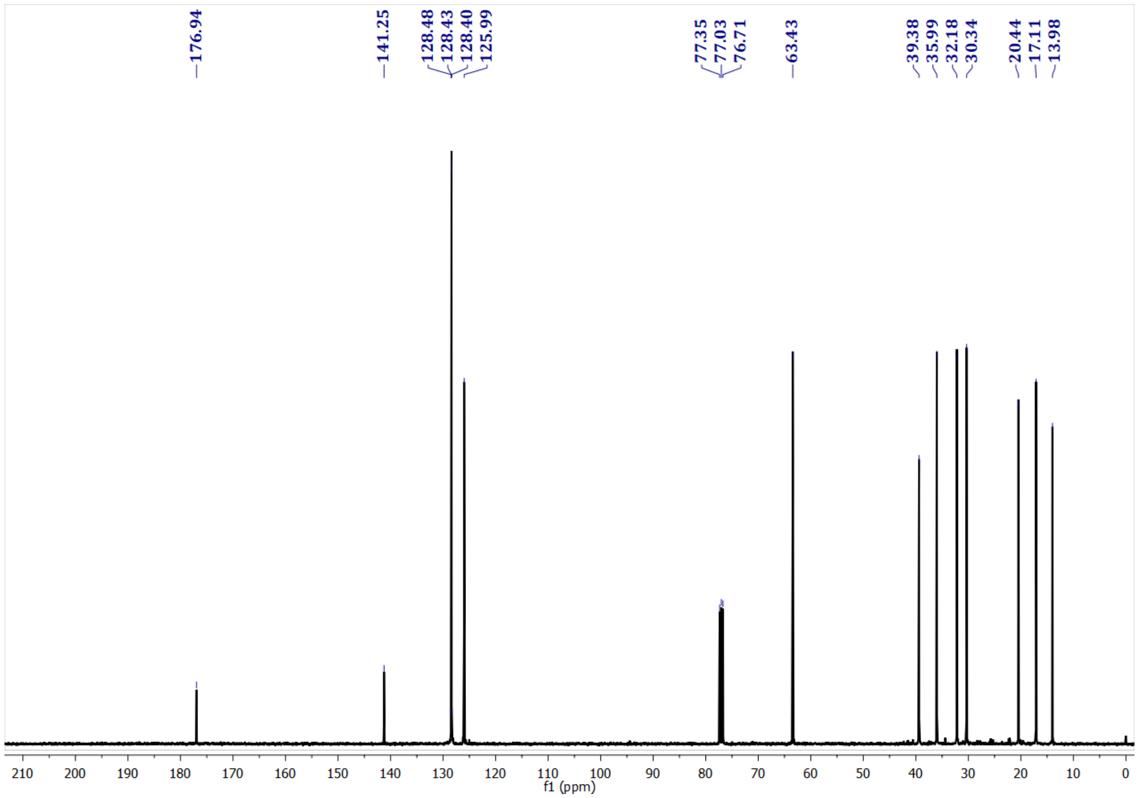

Figure S87a. ^{13}C NMR spectrum of 3-phenylpropyl 2-methylpentanoate (**4e**) recorded in $CDCl_3$

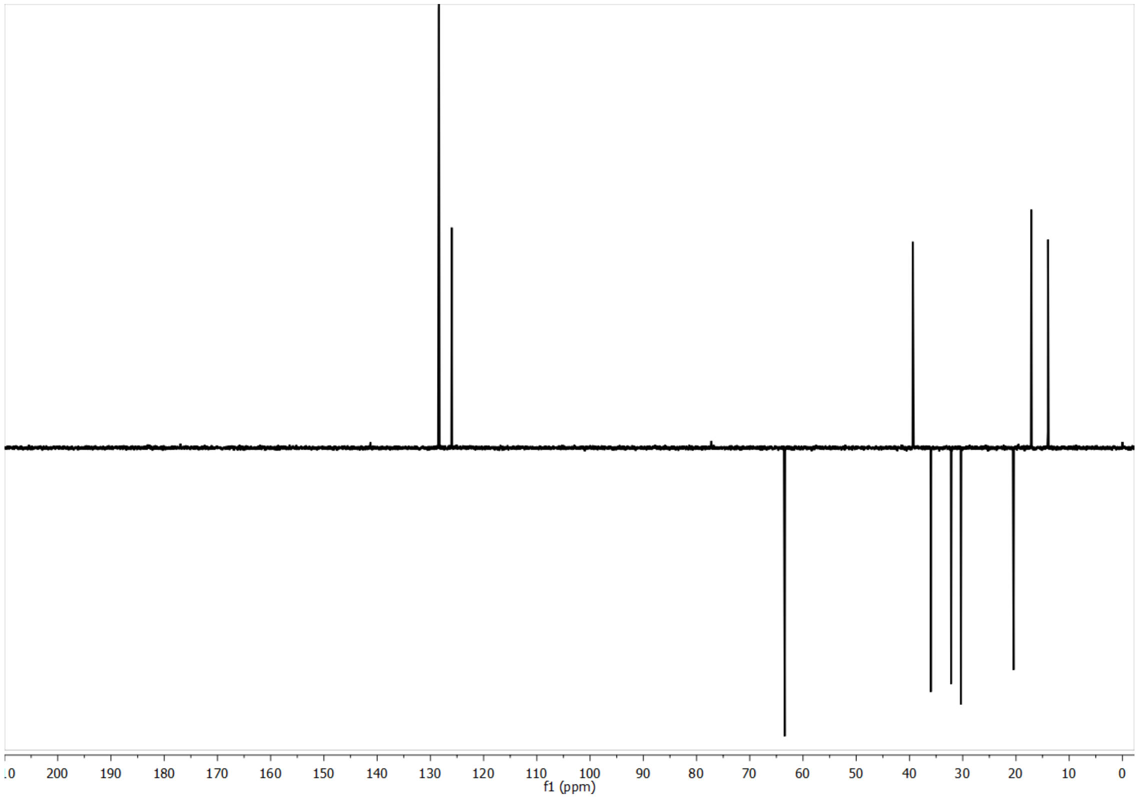

Figure S87b. DEPT 135 spectrum of 3-phenylpropyl 2-methylpentanoate (**4e**)

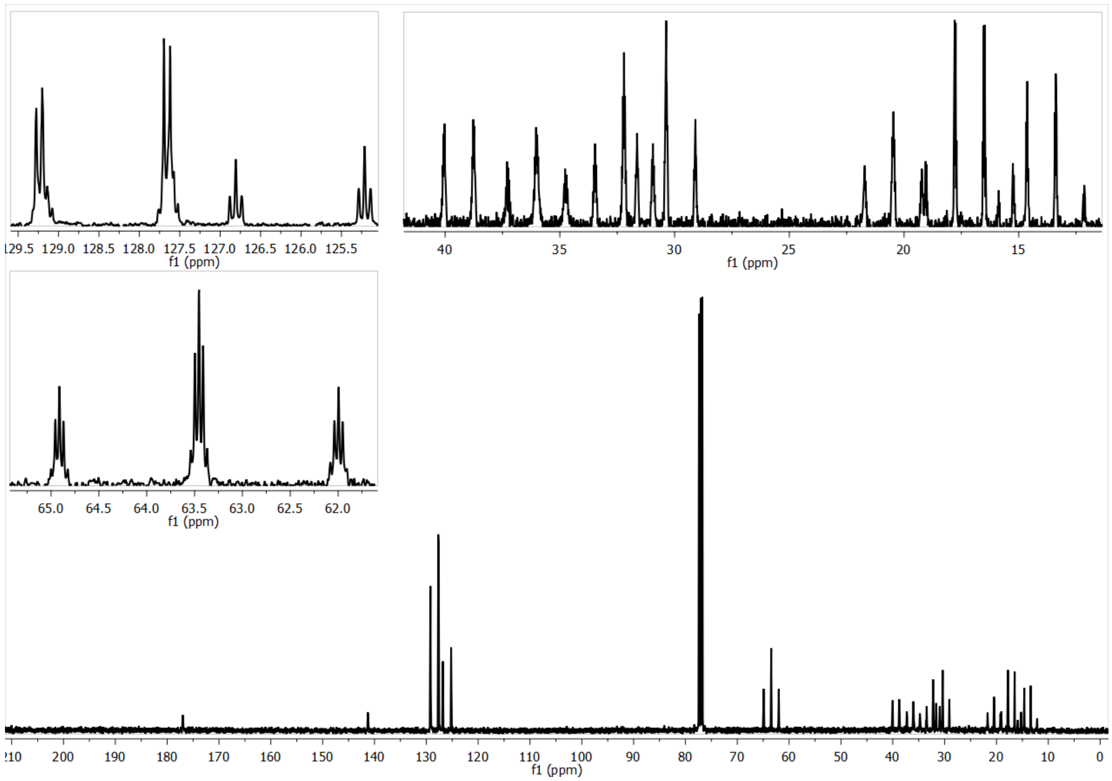

Figure S87c. Proton-coupled ^{13}C NMR spectrum of 3-phenylpropyl 2-methylpentanoate (**4e**)

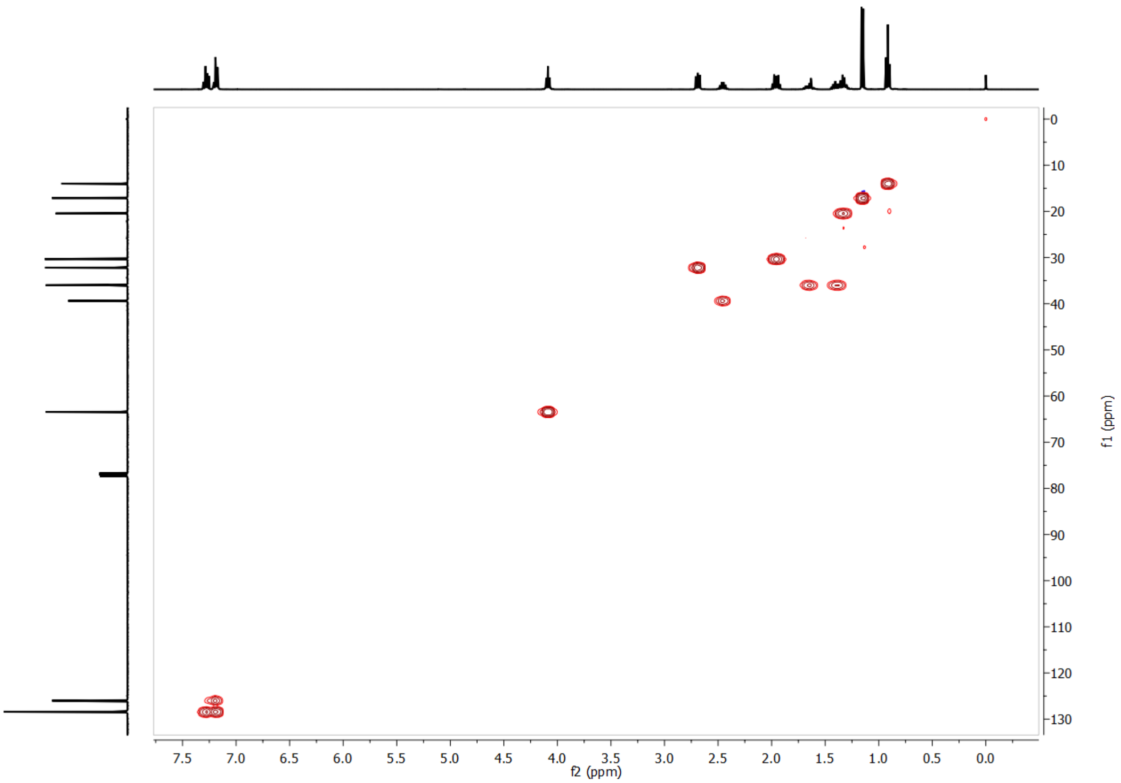

Figure S87d. grHSQC spectrum of 3-phenylpropyl 2-methylpentanoate (**4e**)

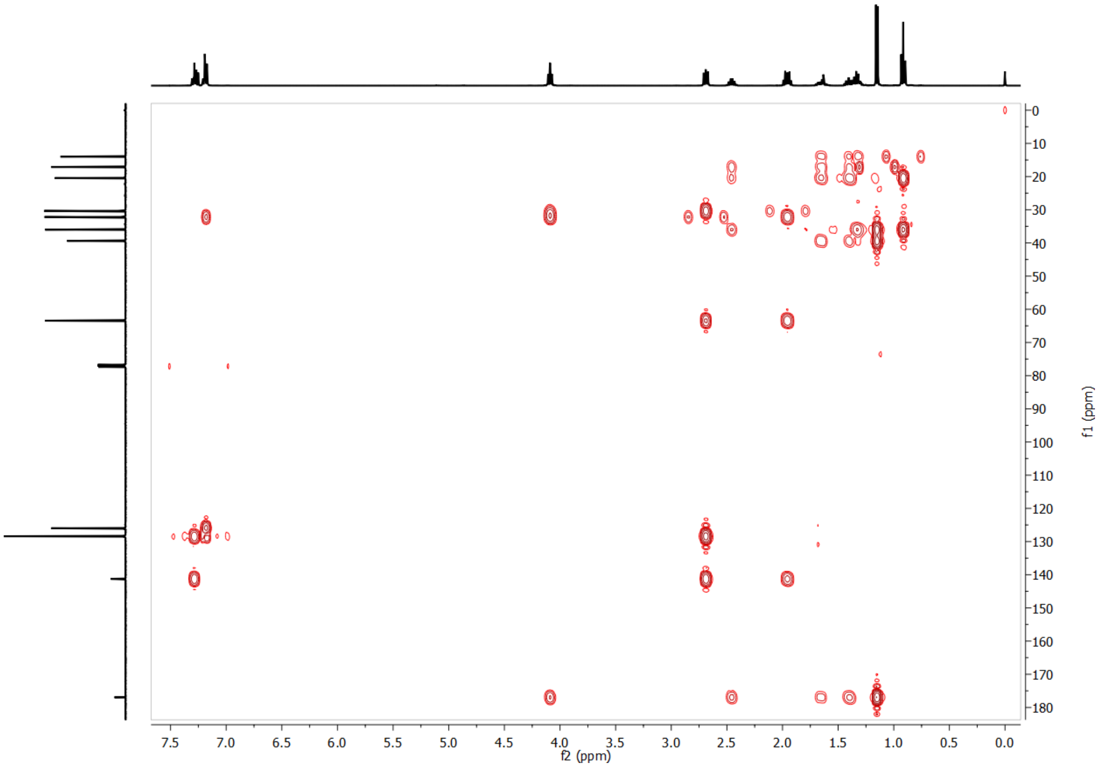

Figure S87e. grHMBC spectrum of 3-phenylpropyl 2-methylpentanoate (**4e**)

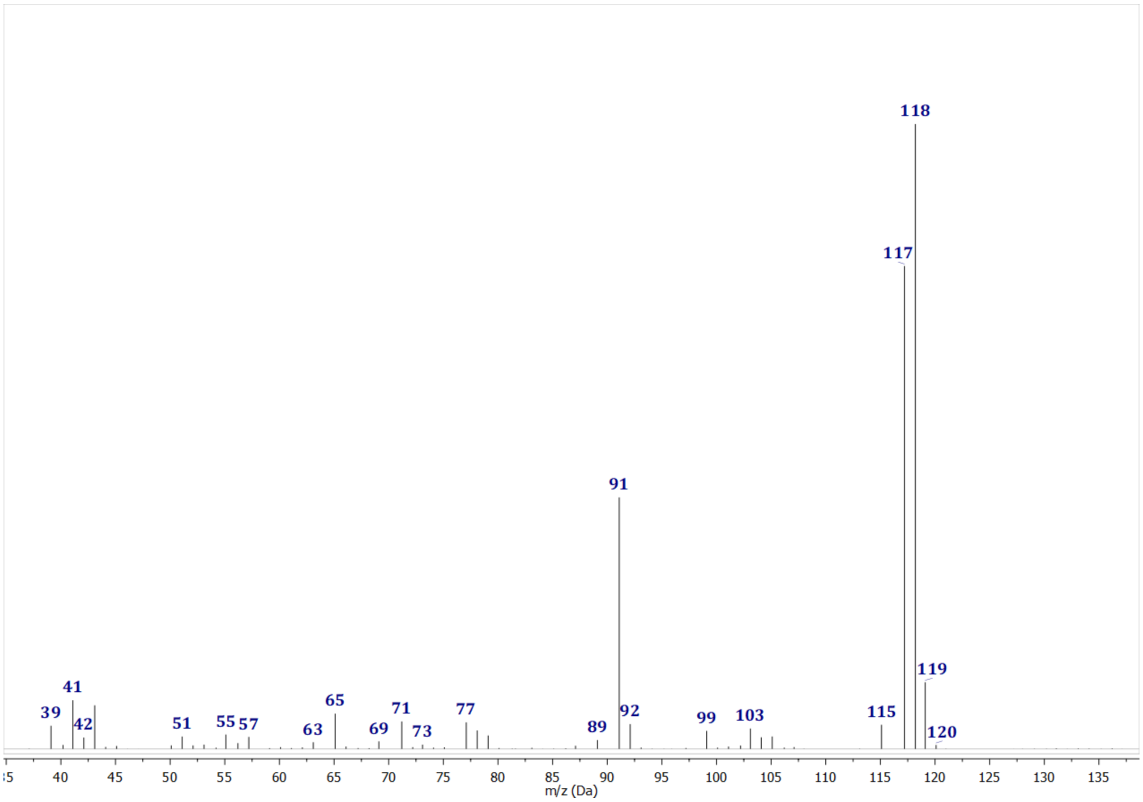

Figure S88. Mass spectrum of 3-phenylpropyl 3-methylpentanoate (**4f**)

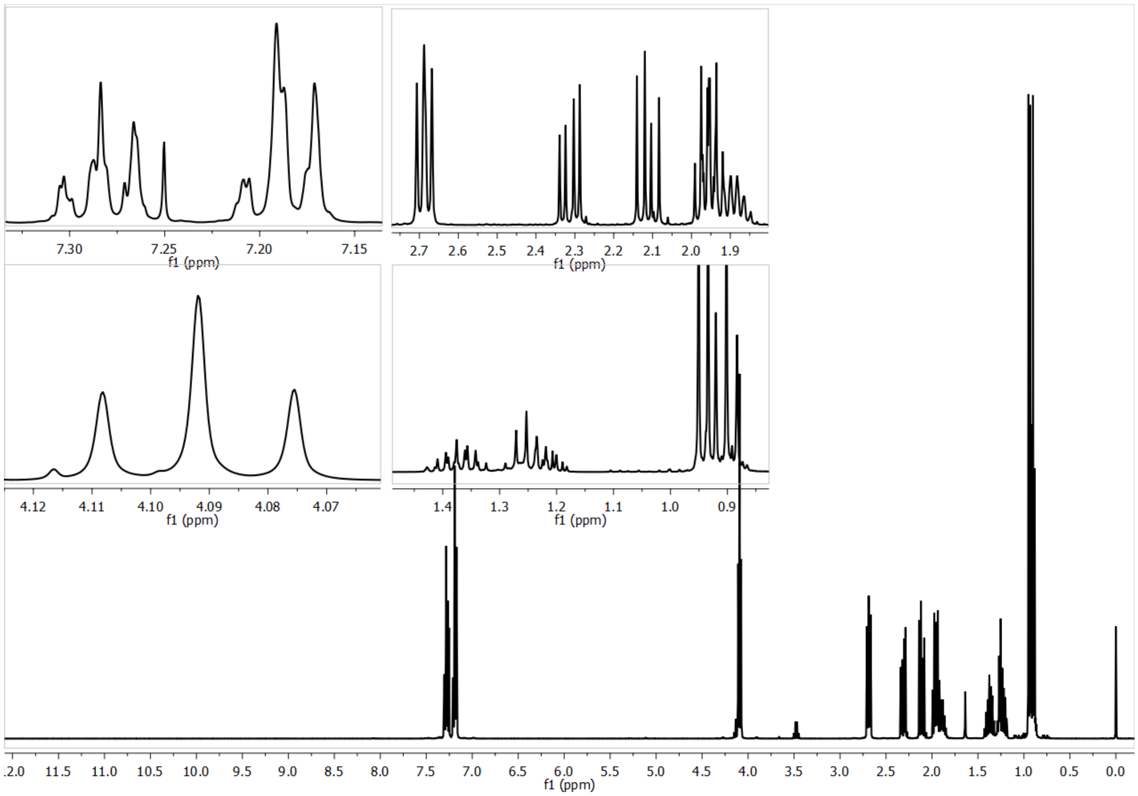

Figure S89. ^{1}H NMR spectrum of 3-phenylpropyl 3-methylpentanoate (**4f**) recorded in $CDCl_3$

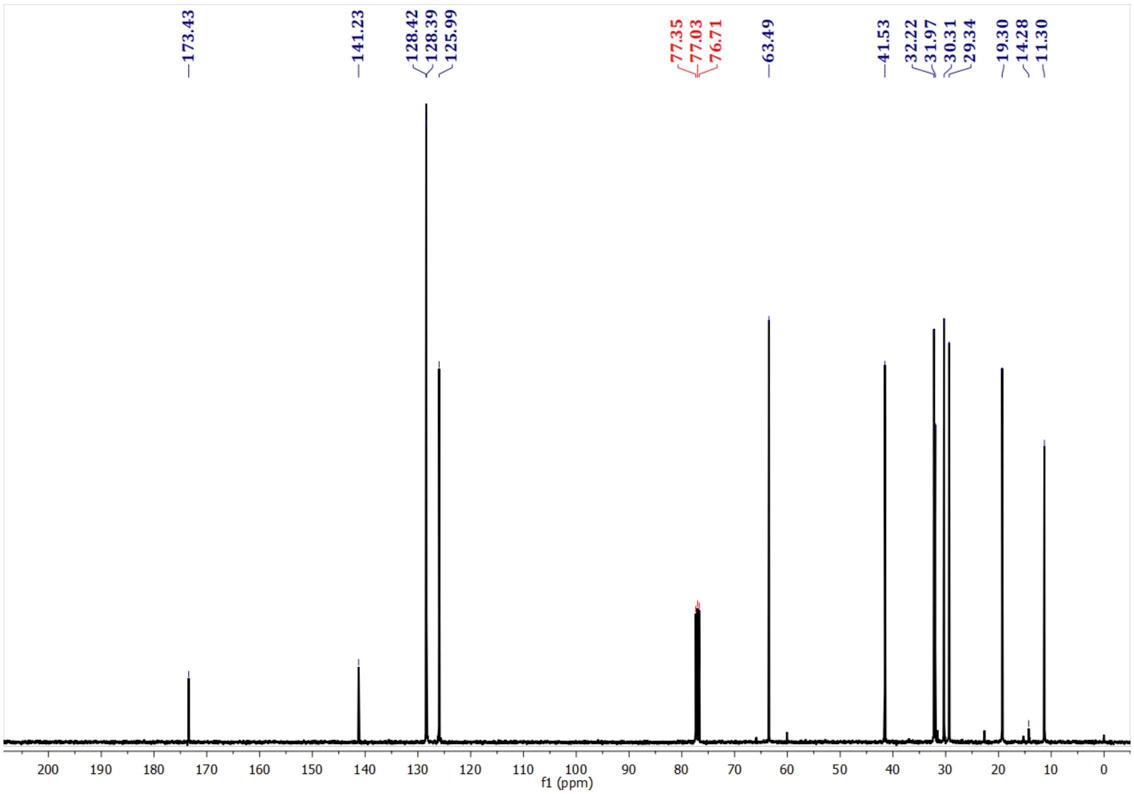

Figure S90. ^{13}C NMR spectrum of 3-phenylpropyl 3-methylpentanoate (**4f**) recorded in $CDCl_3$

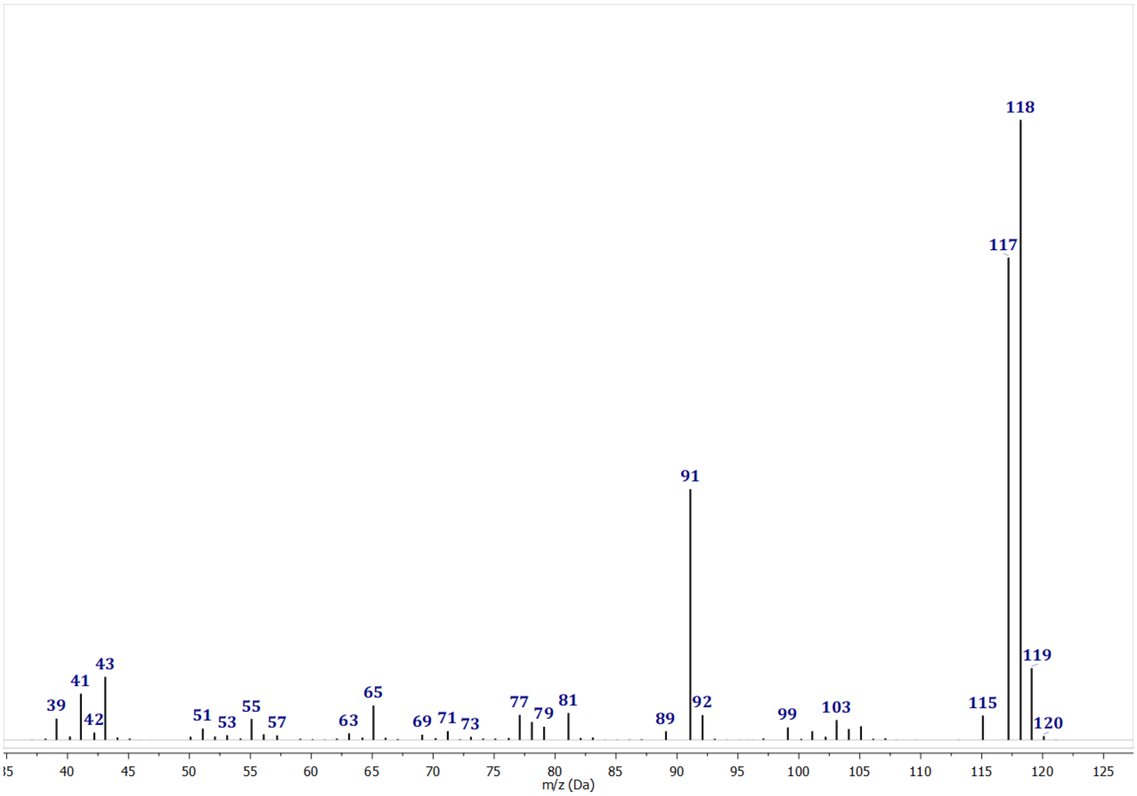

Figure S91. Mass spectrum of 3-phenylpropyl 4-methylpentanoate (**4g**)

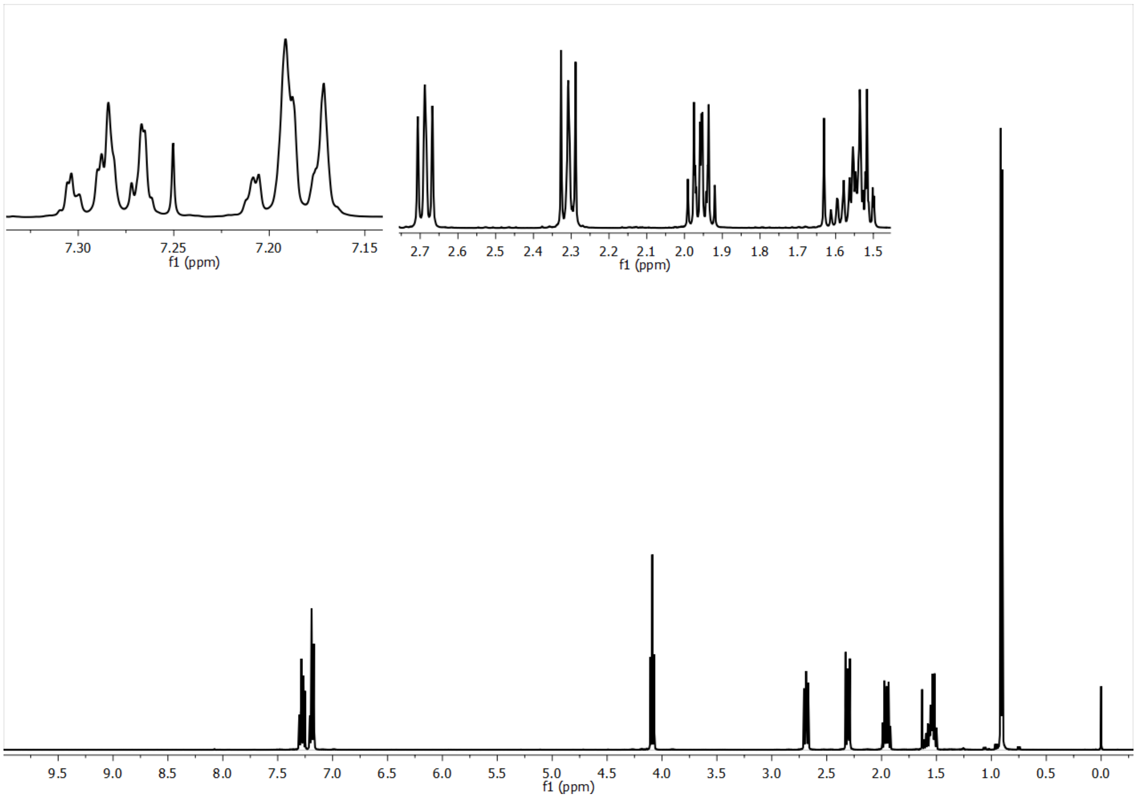

Figure S92. ^{1}H NMR spectrum of 3-phenylpropyl 4-methylpentanoate (**4g**) recorded in $CDCl_3$

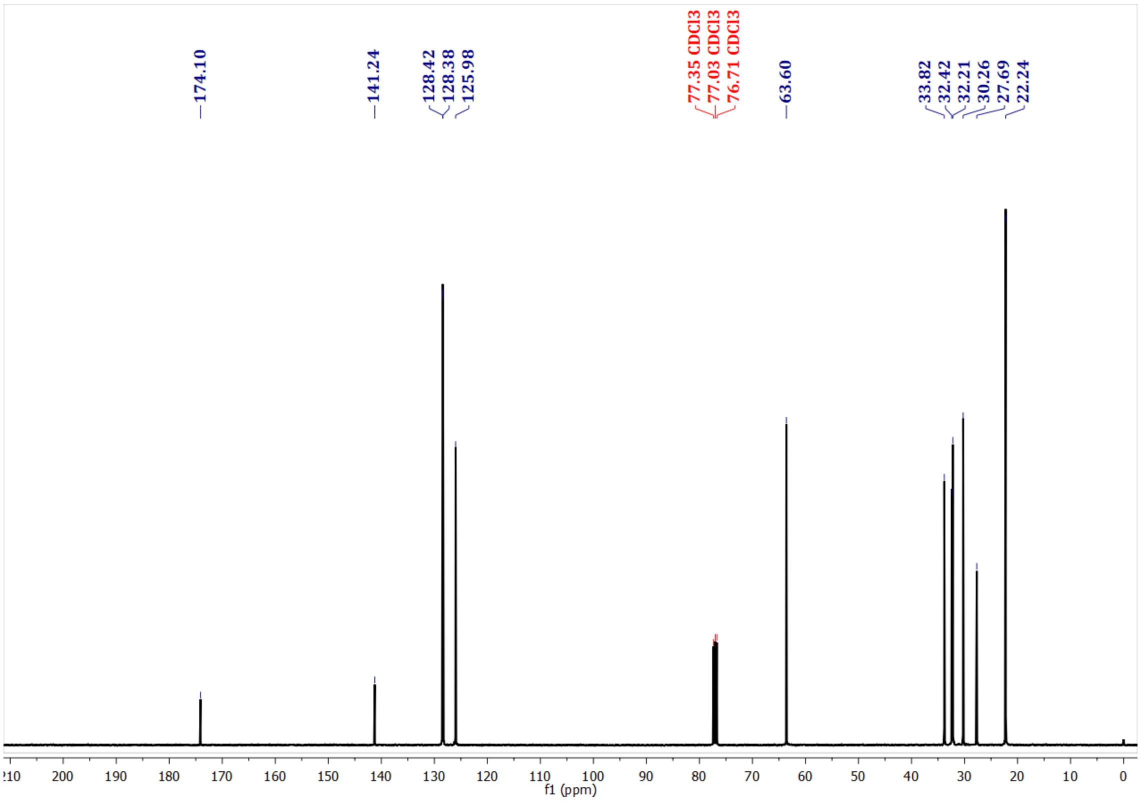

Figure S93. ^{13}C NMR spectrum of 3-phenylpropyl 4-methylpentanoate (**4g**) recorded in $CDCl_3$

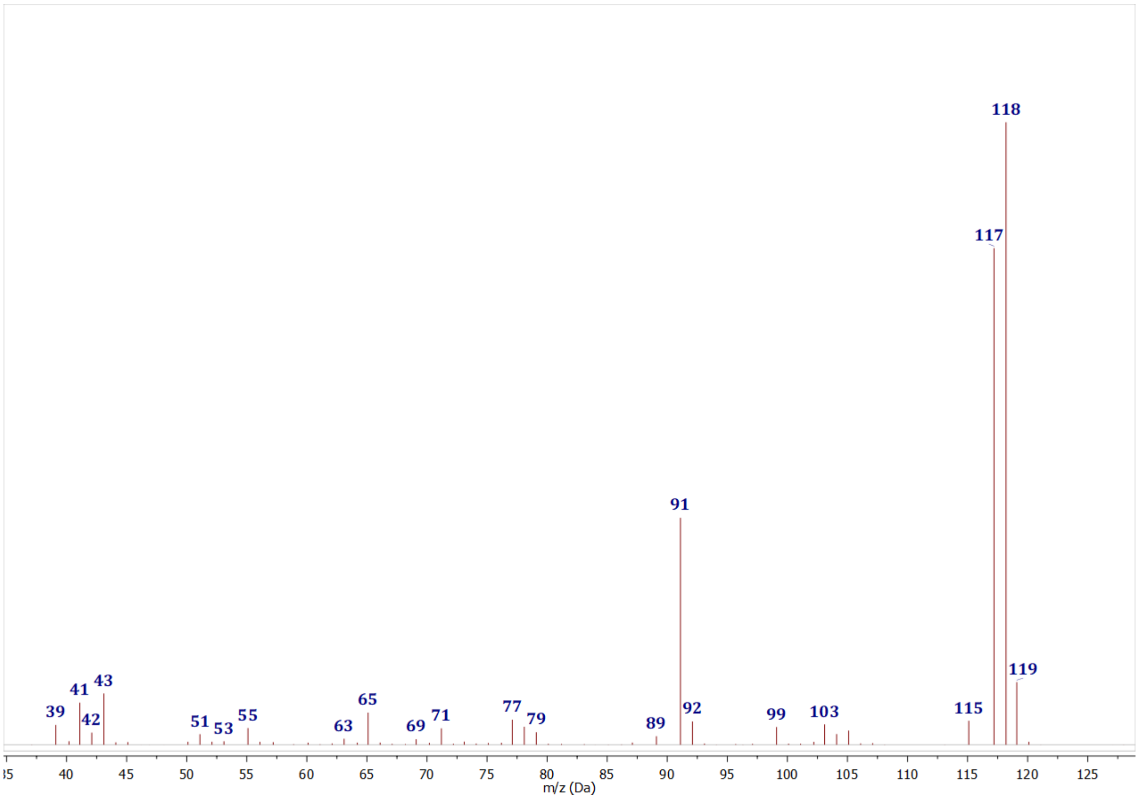

Figure S94. Mass spectrum of 3-phenylpropyl hexanoate (**4h**)

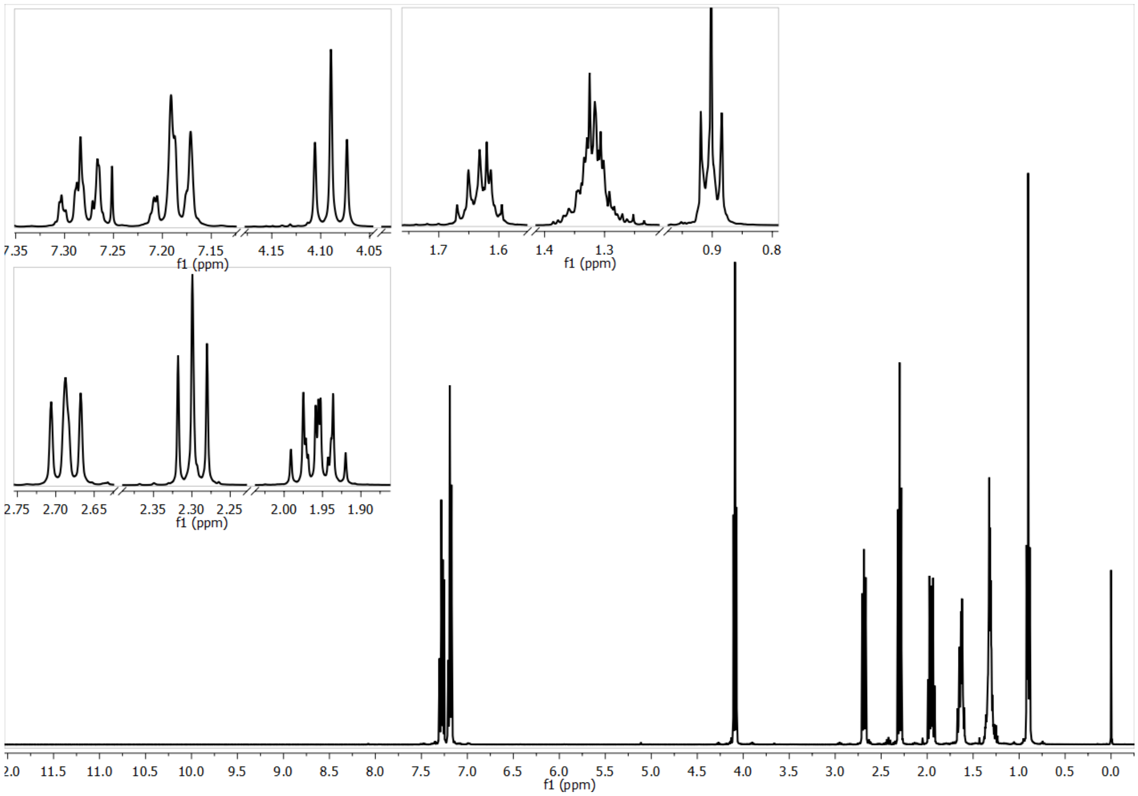

Figure S95. ^{1}H NMR spectrum of 3-phenylpropyl hexanoate (**4h**) recorded in $CDCl_3$

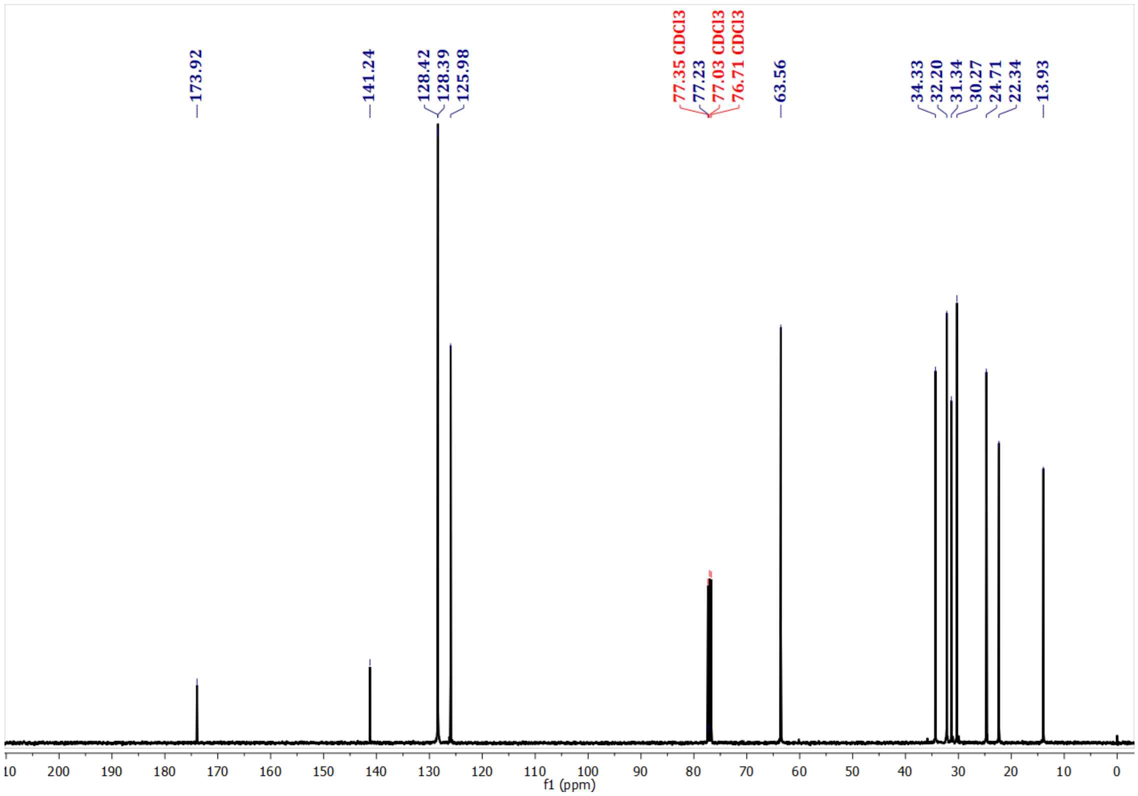

Figure S96. ^{13}C NMR spectrum of 3-phenylpropyl hexanoate (**4h**) recorded in $CDCl_3$

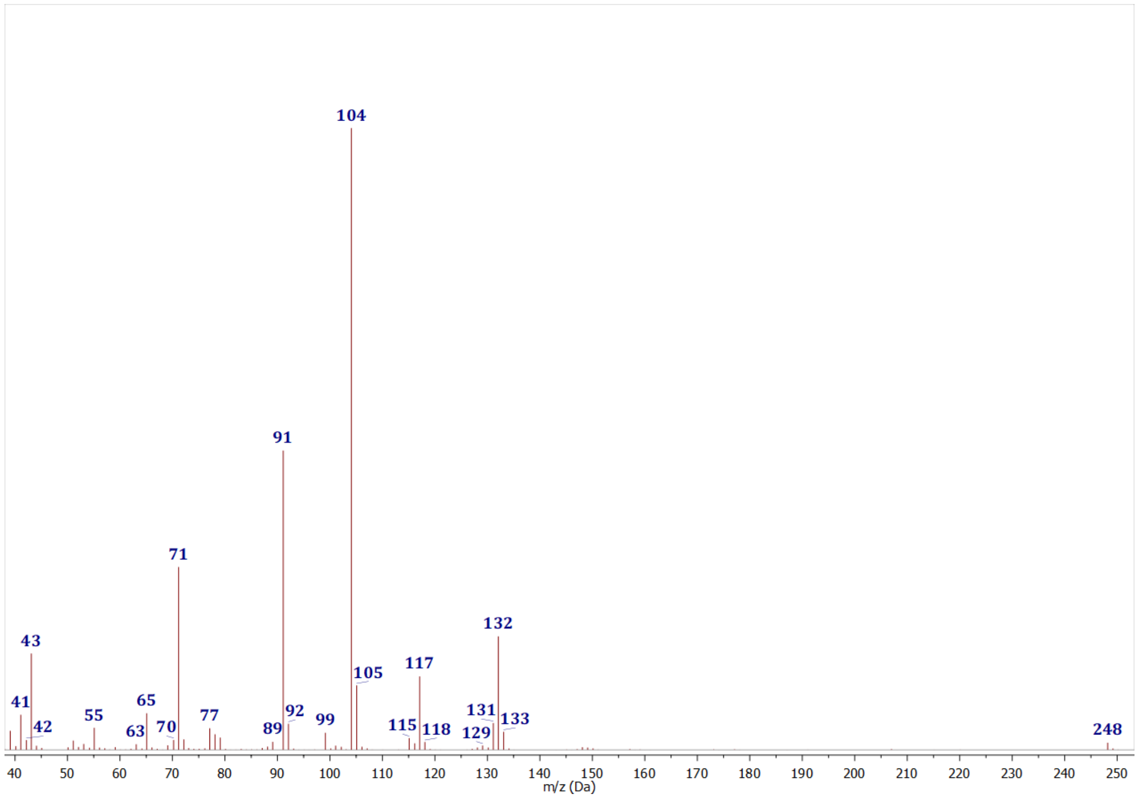

Figure S97. Mass spectrum of 4-phenylbutyl 2-ethylbutanoate (**5a**)

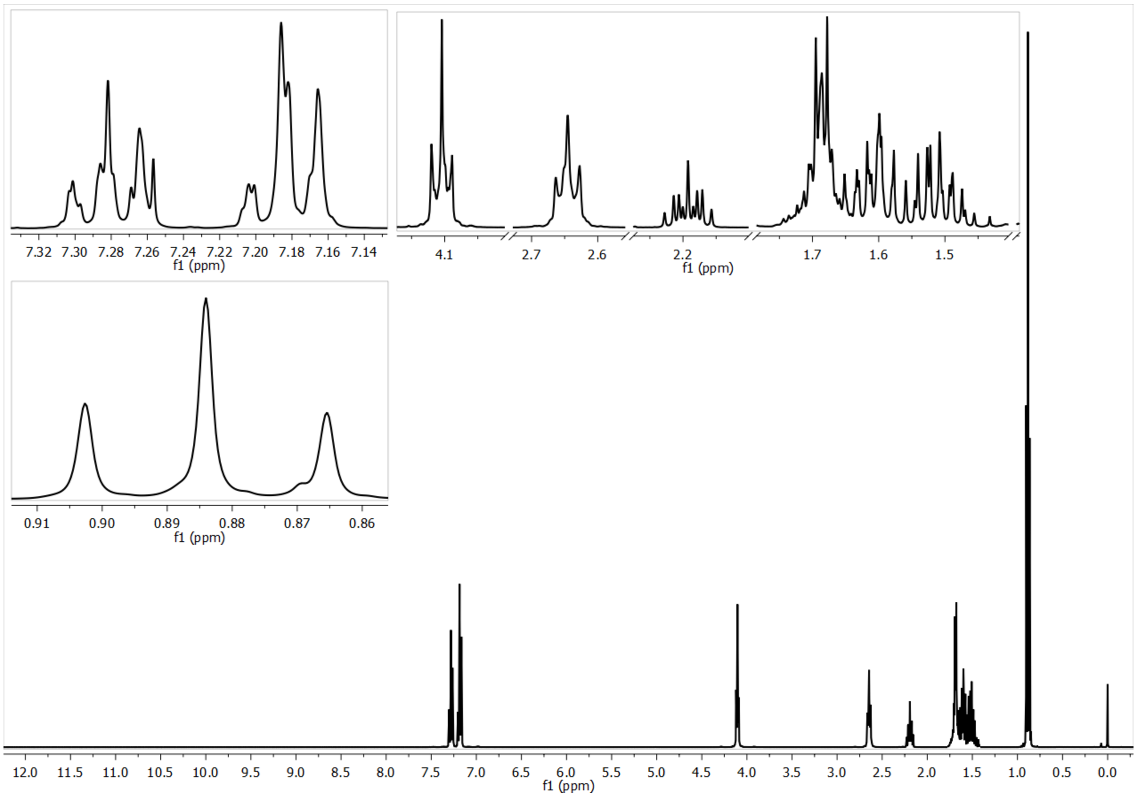

Figure S98. ^{1}H NMR spectrum of 4-phenylbutyl 2-ethylbutanoate (**5a**) recorded in $CDCl_3$

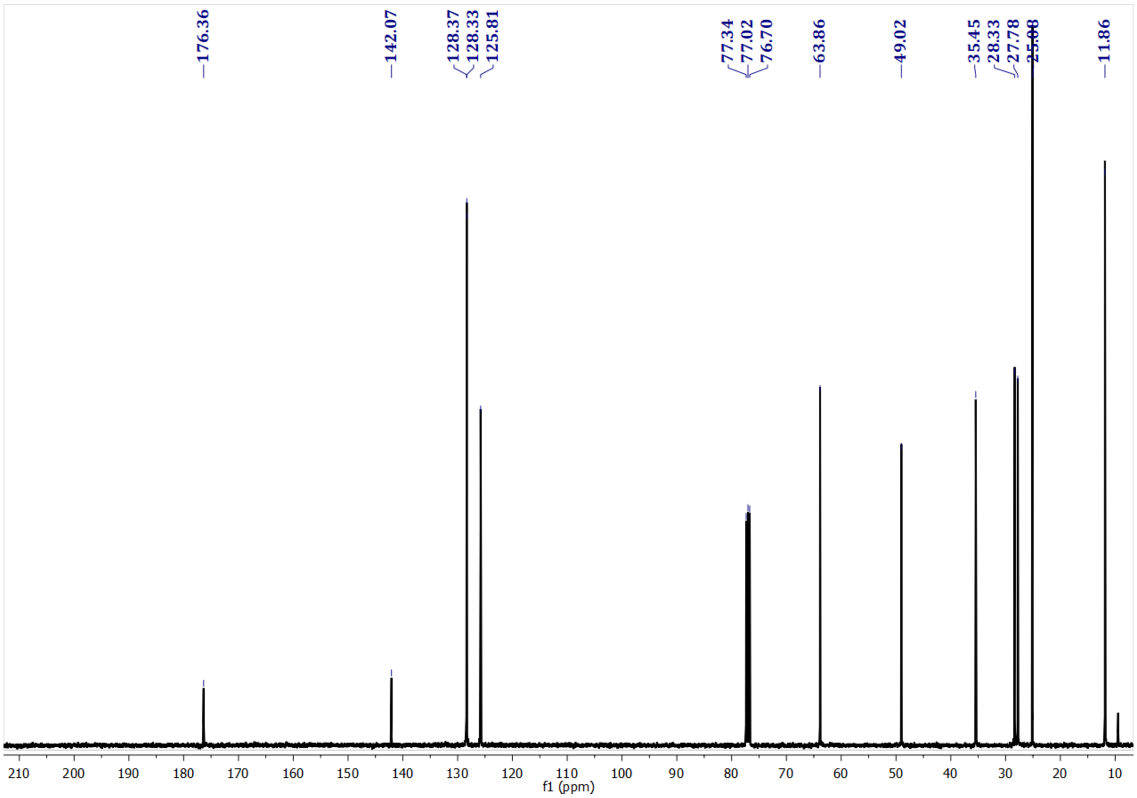

Figure S99. ^{13}C NMR spectrum of 4-phenylbutyl 2-ethylbutanoate (**5a**) recorded in $CDCl_3$

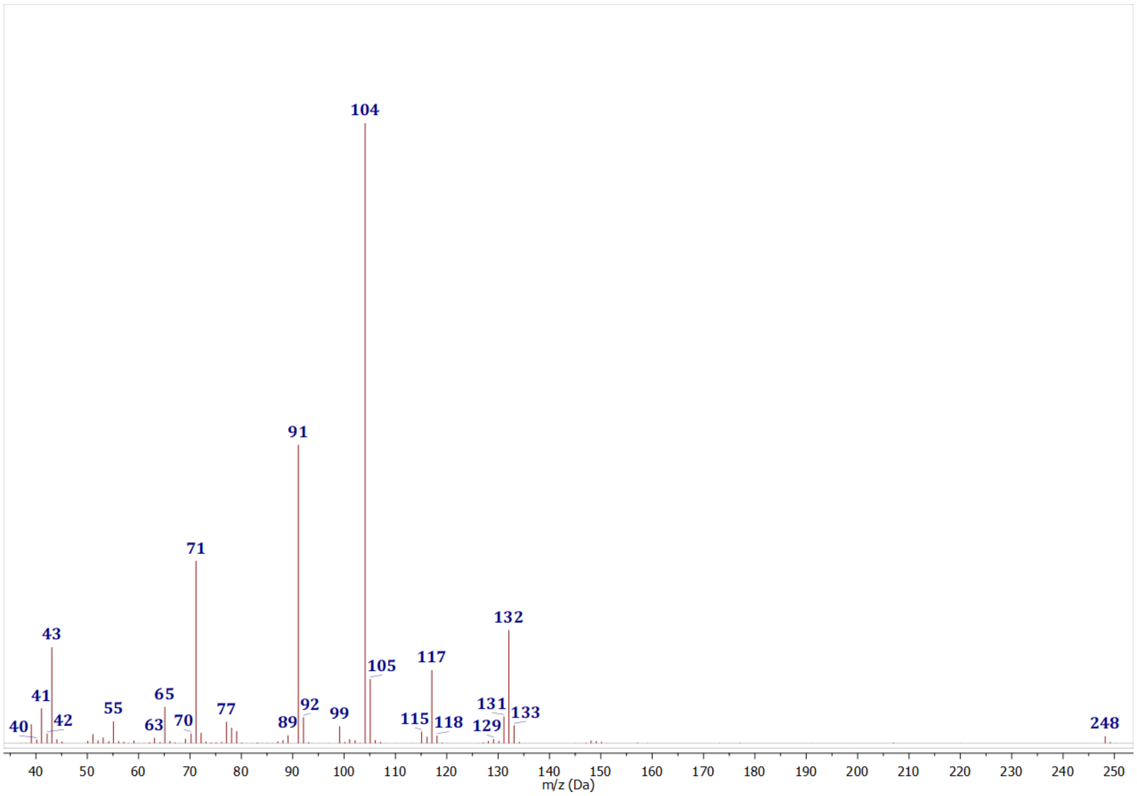

Figure S100. Mass spectrum of 4-phenylbutyl 2,2-dimethylbutanoate (**5b**)

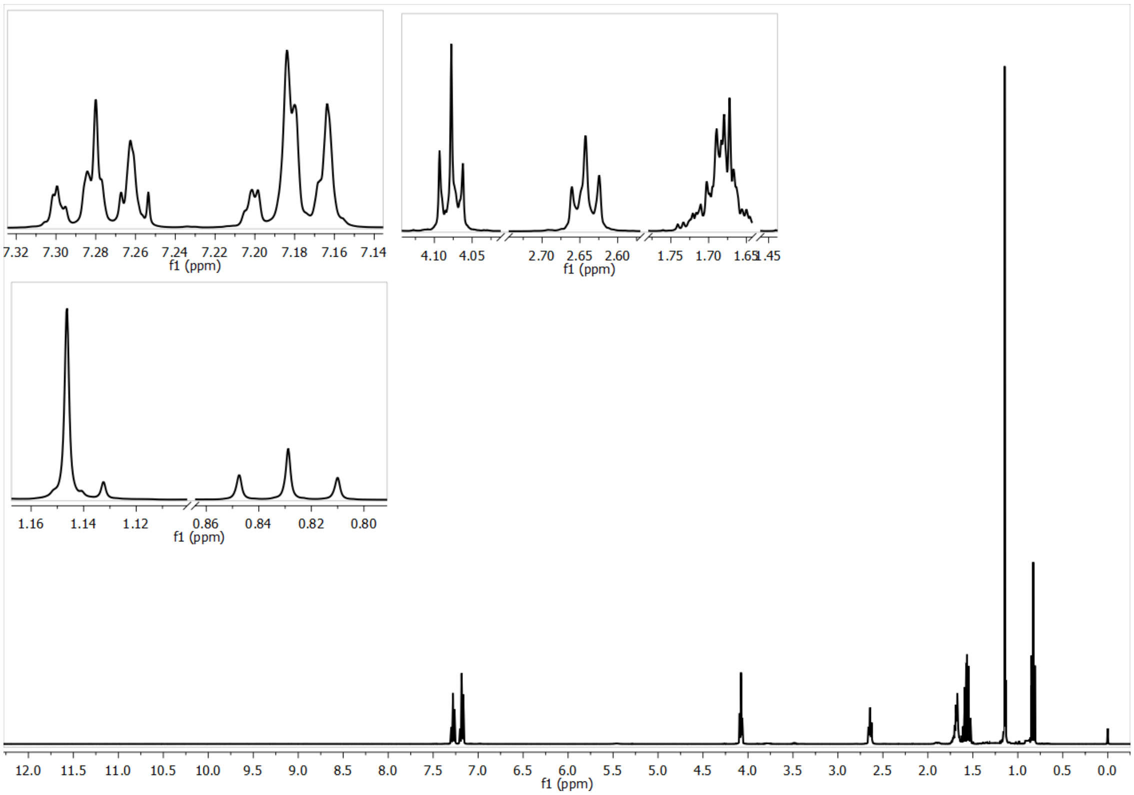

Figure S101. ^{1}H NMR spectrum of 4-phenylbutyl 2,2-dimethylbutanoate (**5b**) recorded in $CDCl_3$

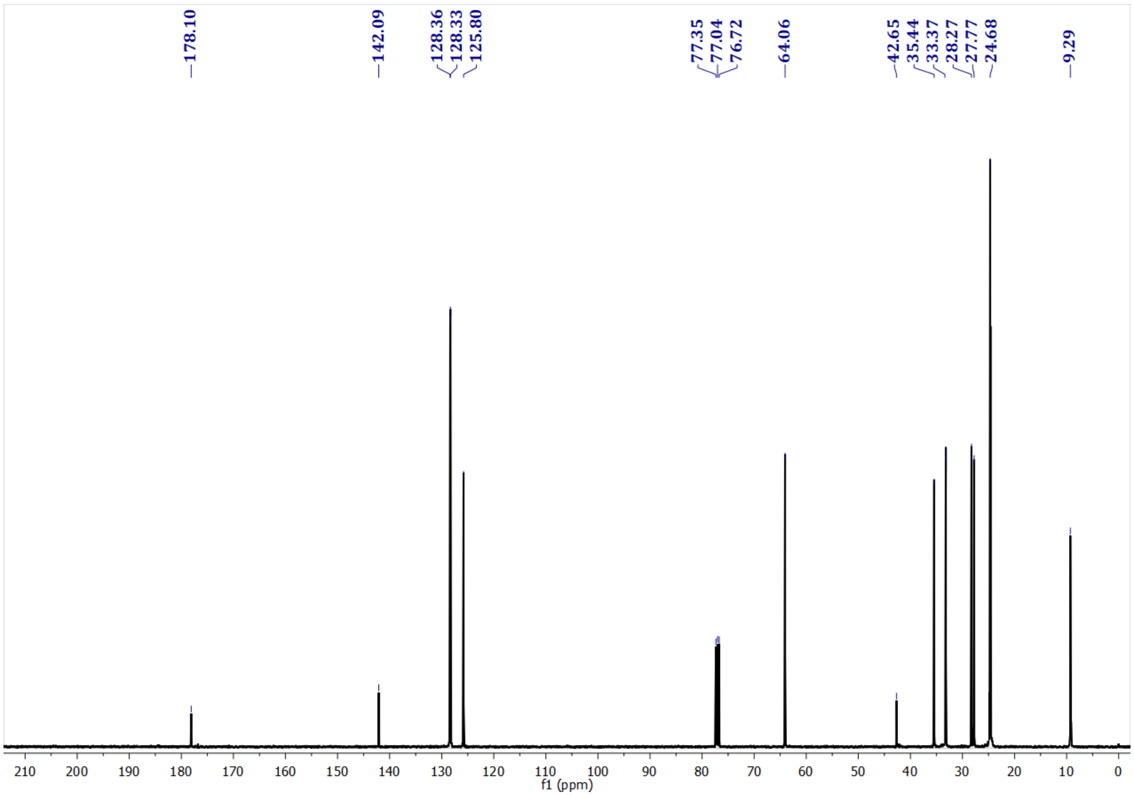

Figure S102. ^{13}C NMR spectrum of 4-phenylbutyl 2,2-dimethylbutanoate (**5b**) recorded in $CDCl_3$

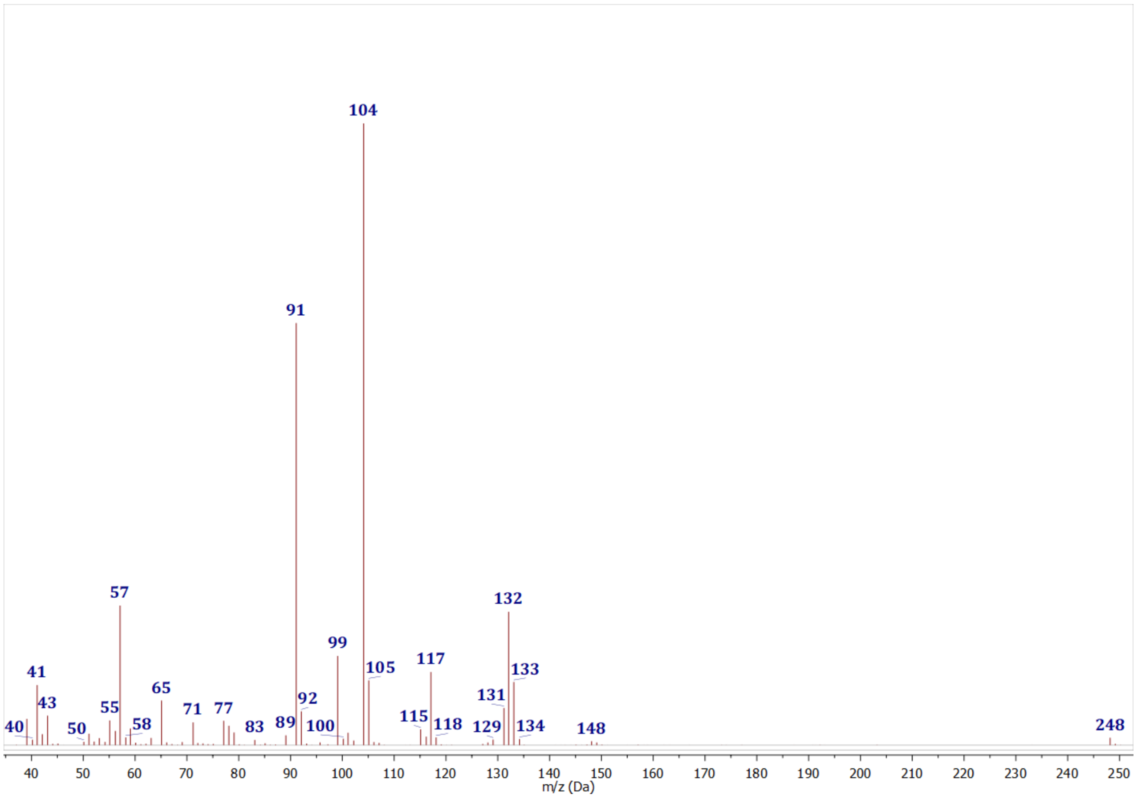

Figure S103. Mass spectrum of 4-phenylbutyl 3,3-dimethylbutanoate (**5c**)

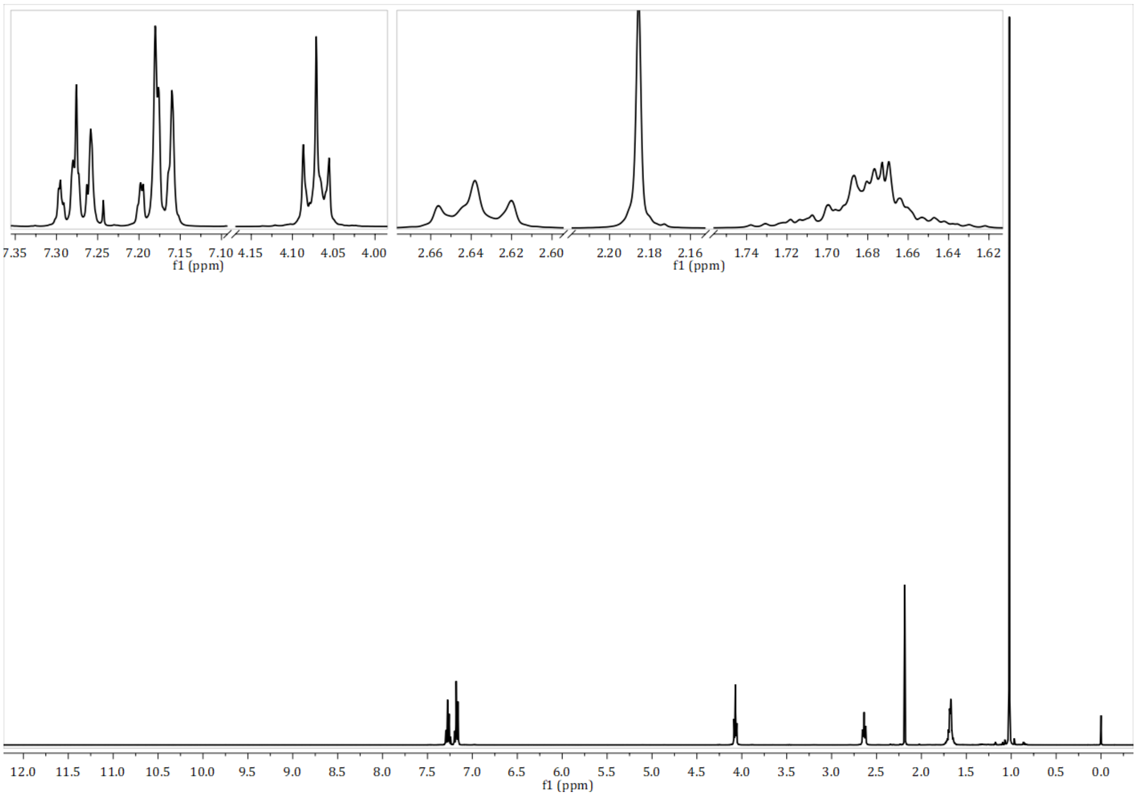

Figure S104. ^{1}H NMR spectrum of 4-phenylbutyl 3,3-dimethylbutanoate (**5c**) recorded in $CDCl_3$

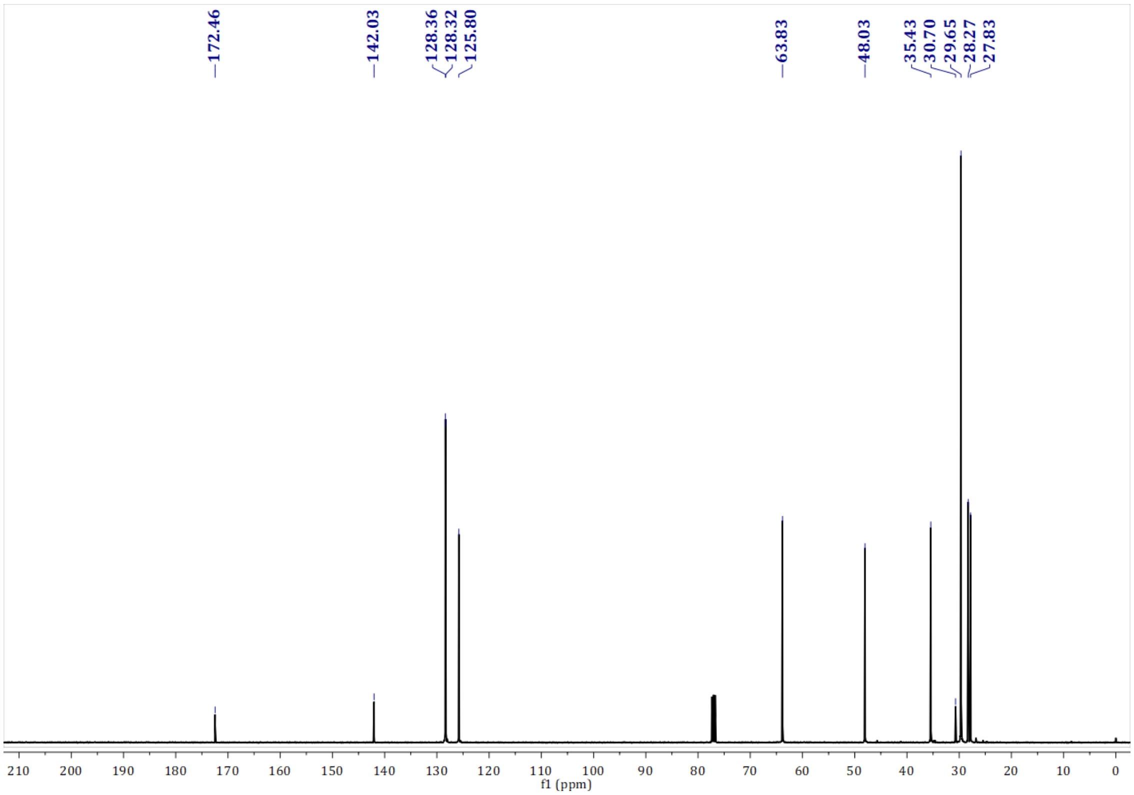

Figure S105. ^{13}C NMR spectrum of 4-phenylbutyl 3,3-dimethylbutanoate (**5c**) recorded in $CDCl_3$

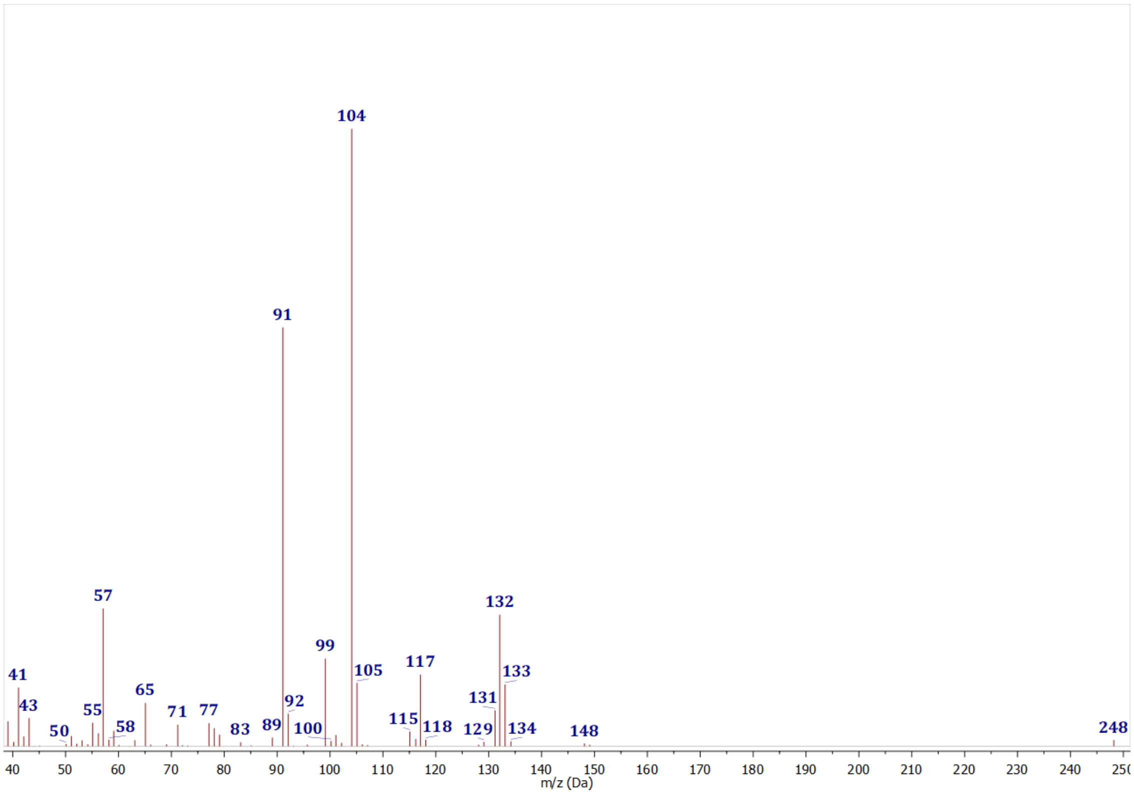

Figure S106. Mass spectrum of 4-phenylbutyl 2,3-dimethylbutanoate (**5d**)

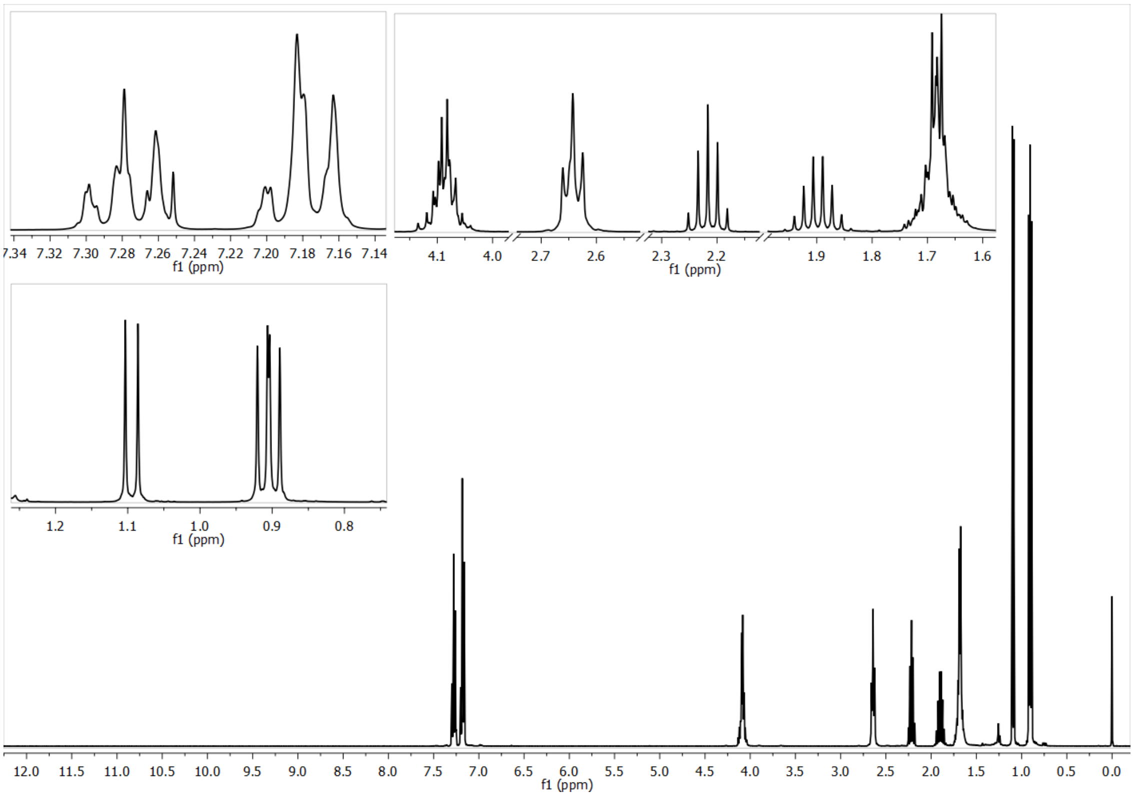

Figure S107. ^{1}H NMR spectrum of 4-phenylbutyl 2,3-dimethylbutanoate (**5d**) recorded in $CDCl_3$

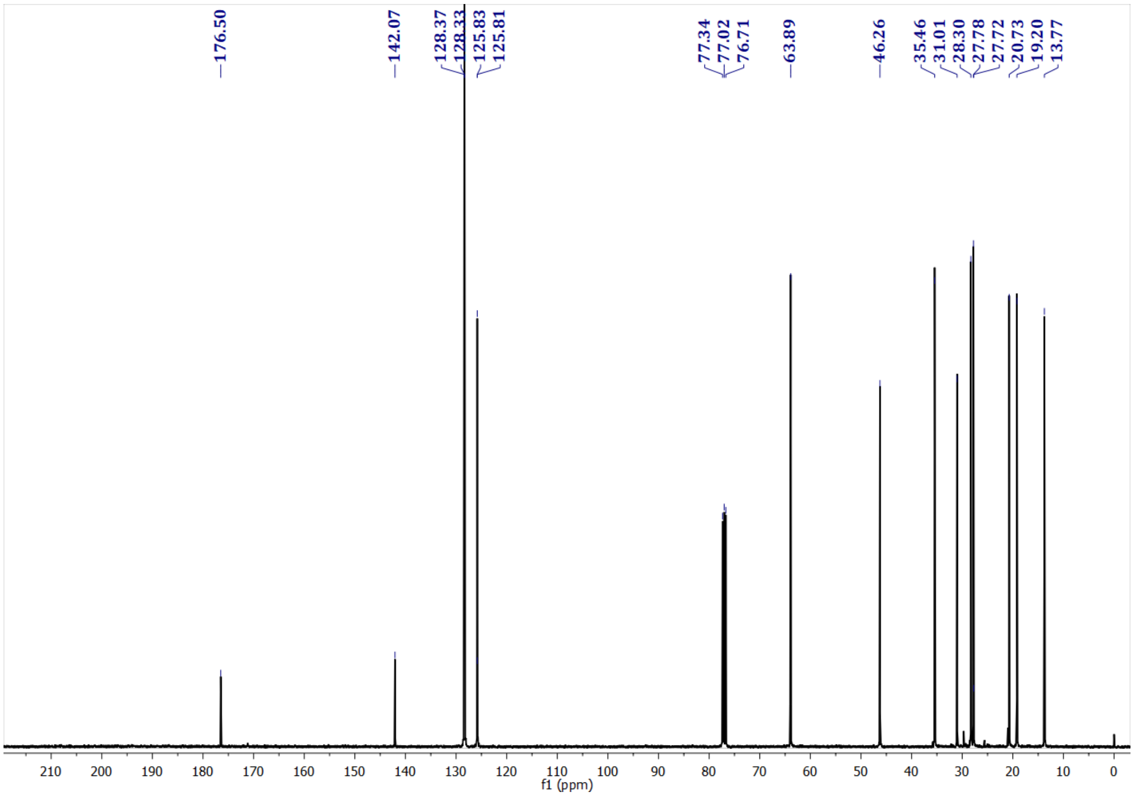

Figure S108. ^{13}C NMR spectrum of 4-phenylbutyl 2,3-dimethylbutanoate (**5d**) recorded in $CDCl_3$

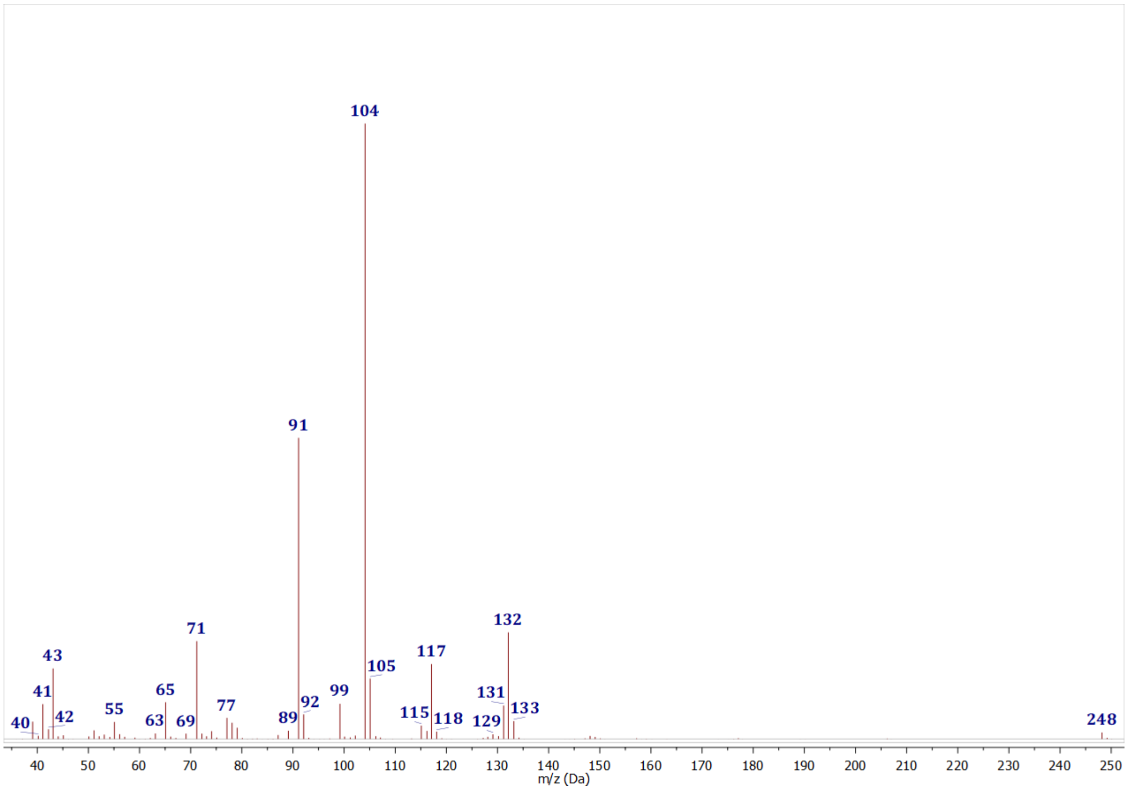

Figure S109. Mass spectrum of 4-phenylbutyl 2-methylpentanoate (**5e**)

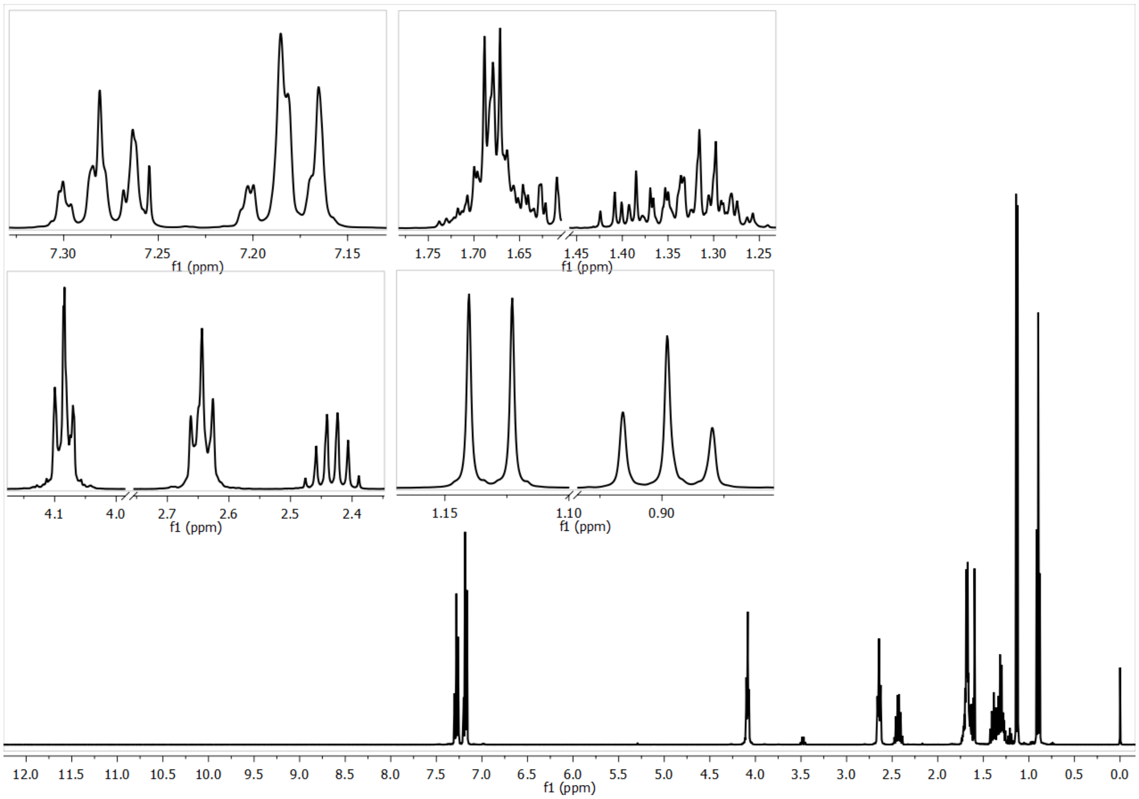

Figure S110. ^{1}H NMR spectrum of 4-phenylbutyl 2-methylpentanoate (**5e**) recorded in $CDCl_3$

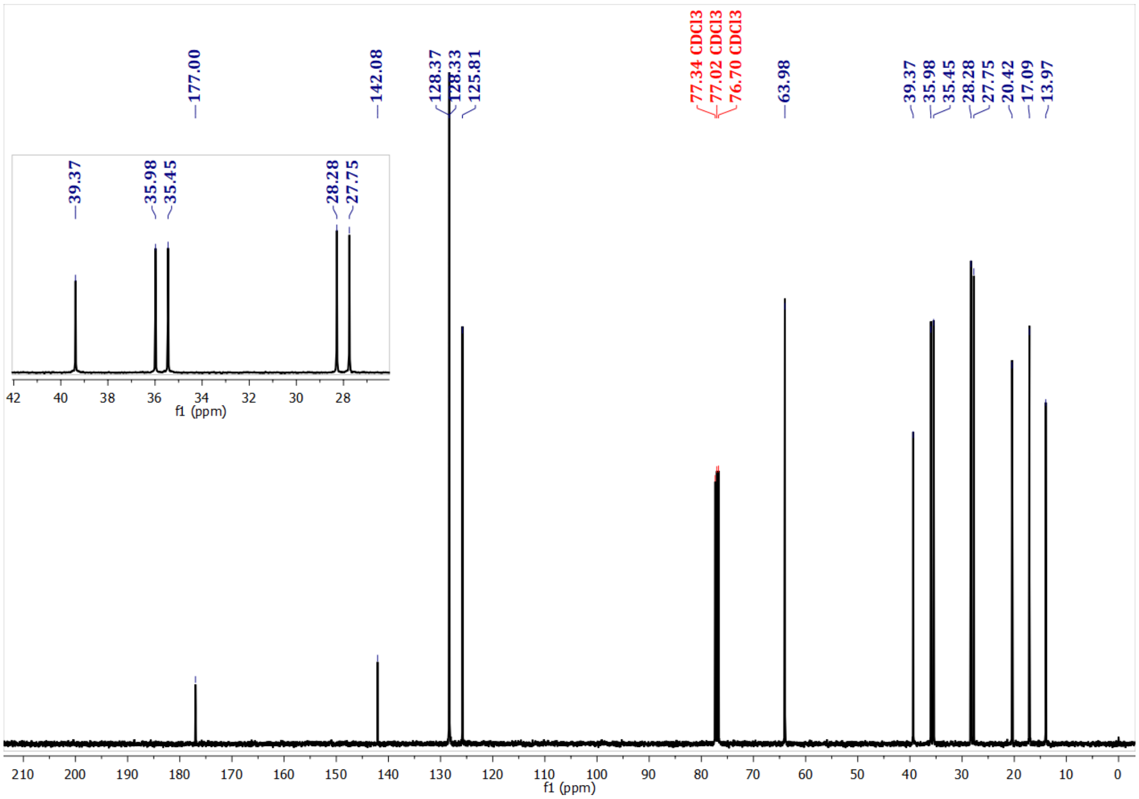

Figure S111. ^{13}C NMR spectrum of 4-phenylbutyl 2-methylpentanoate (**5e**) recorded in $CDCl_3$

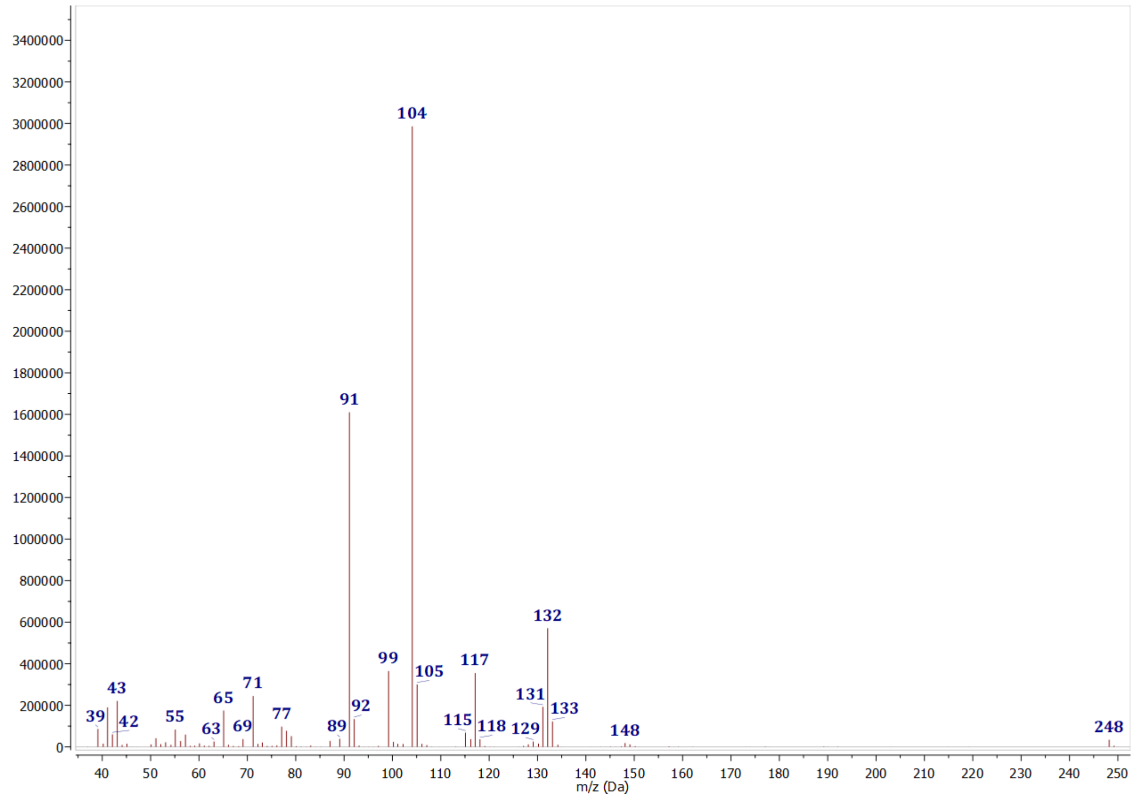

Figure S112. Mass spectrum of 4-phenylbutyl 3-methylpentanoate (**5f**)

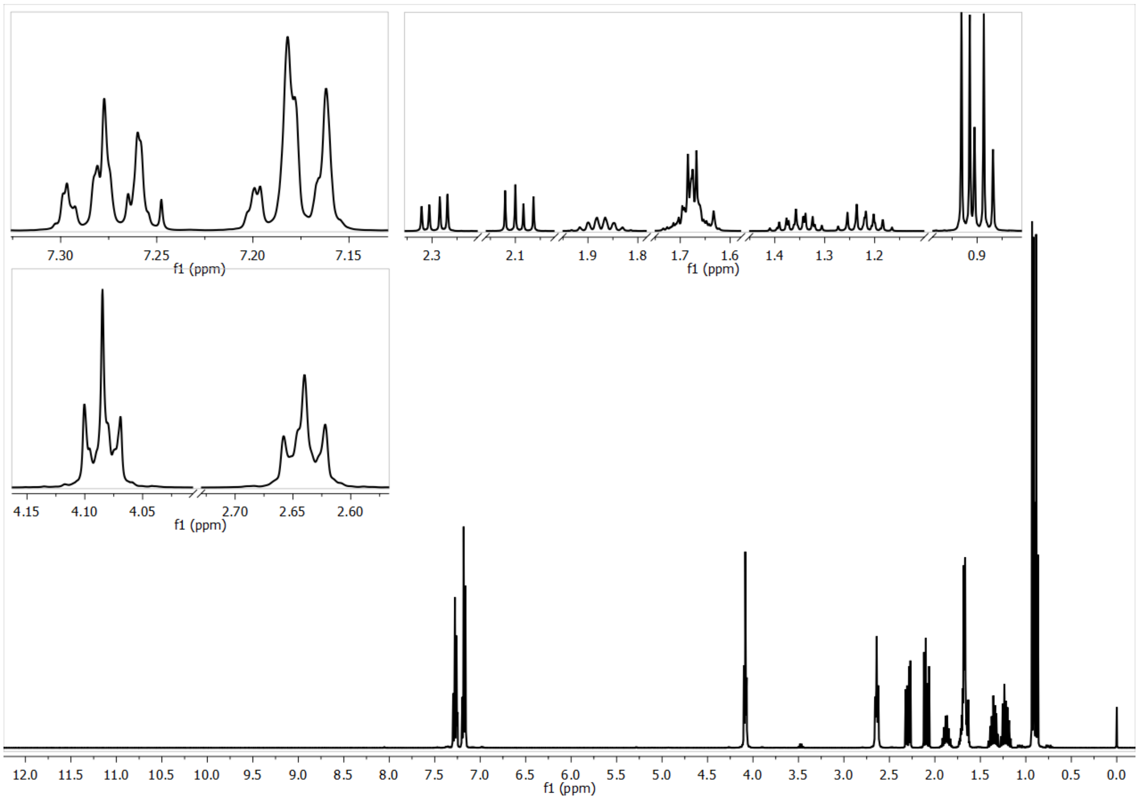

Figure S113. ^{1}H NMR spectrum of 4-phenylbutyl 3-methylpentanoate (**5f**) recorded in $CDCl_3$

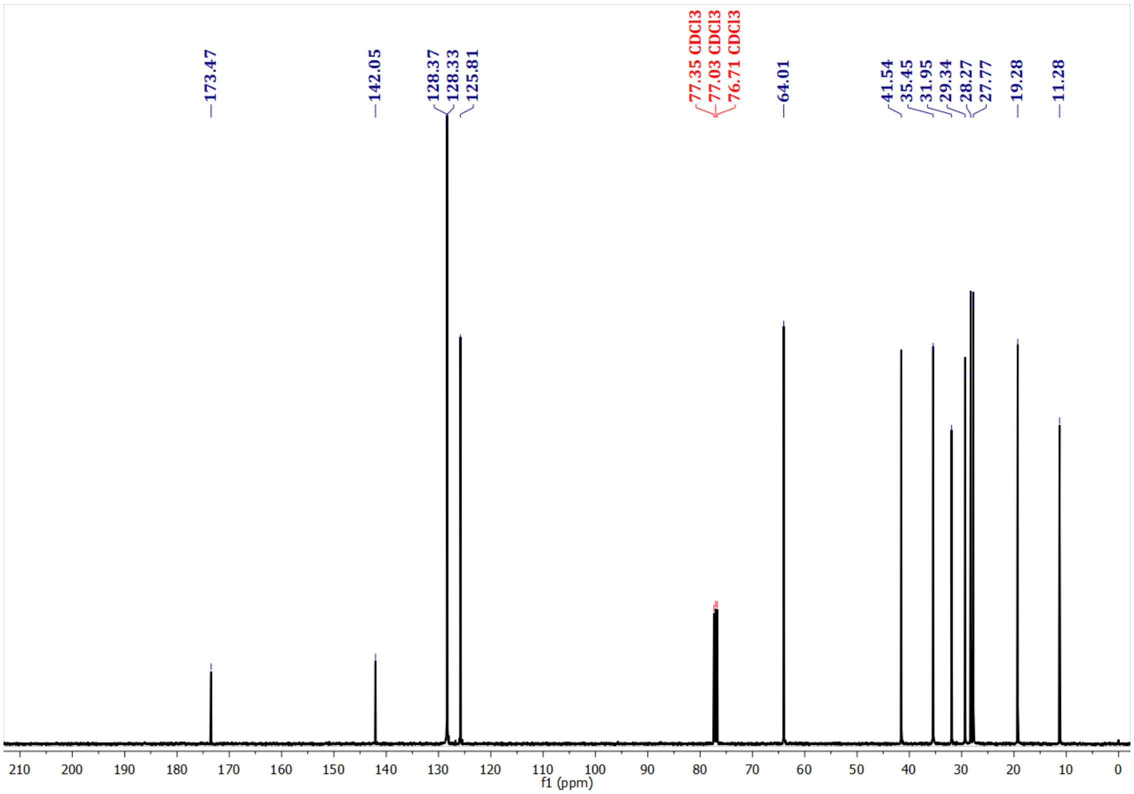

Figure S114. ^{13}C NMR spectrum of 4-phenylbutyl 3-methylpentanoate (**5f**) recorded in $CDCl_3$

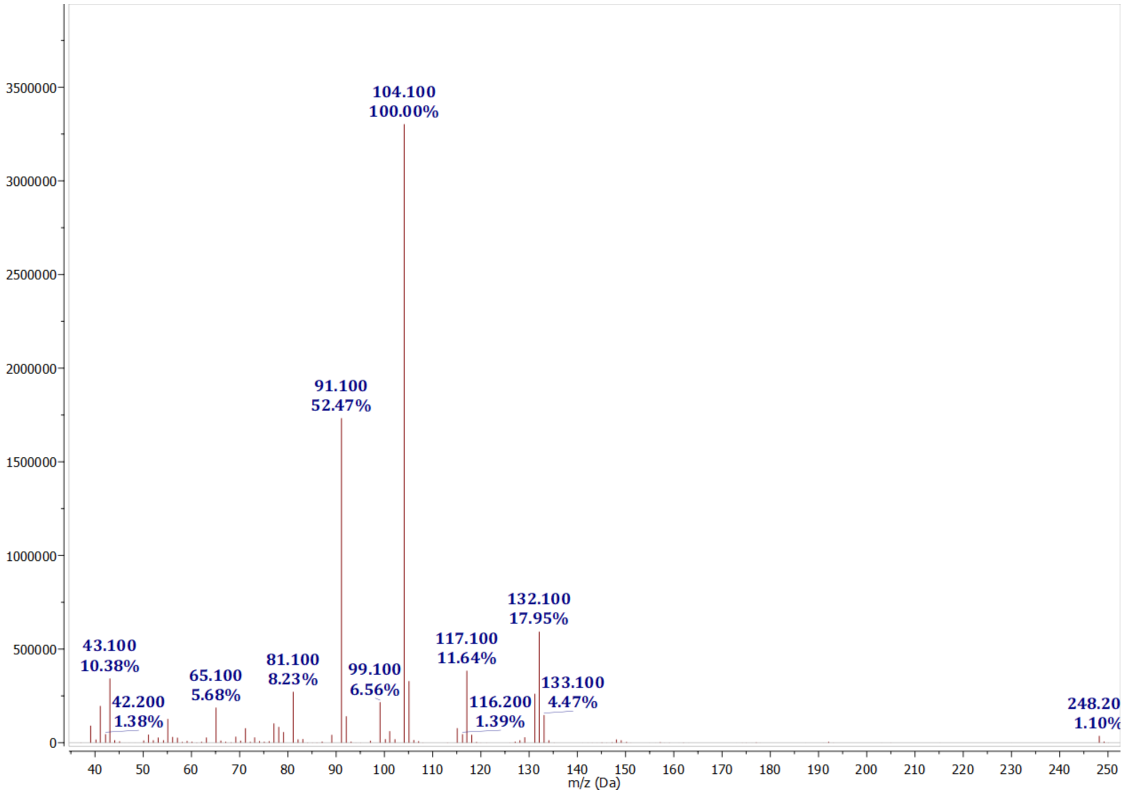

Figure S115. Mass spectrum of 4-phenylbutyl 4-methylpentanoate (**5g**)

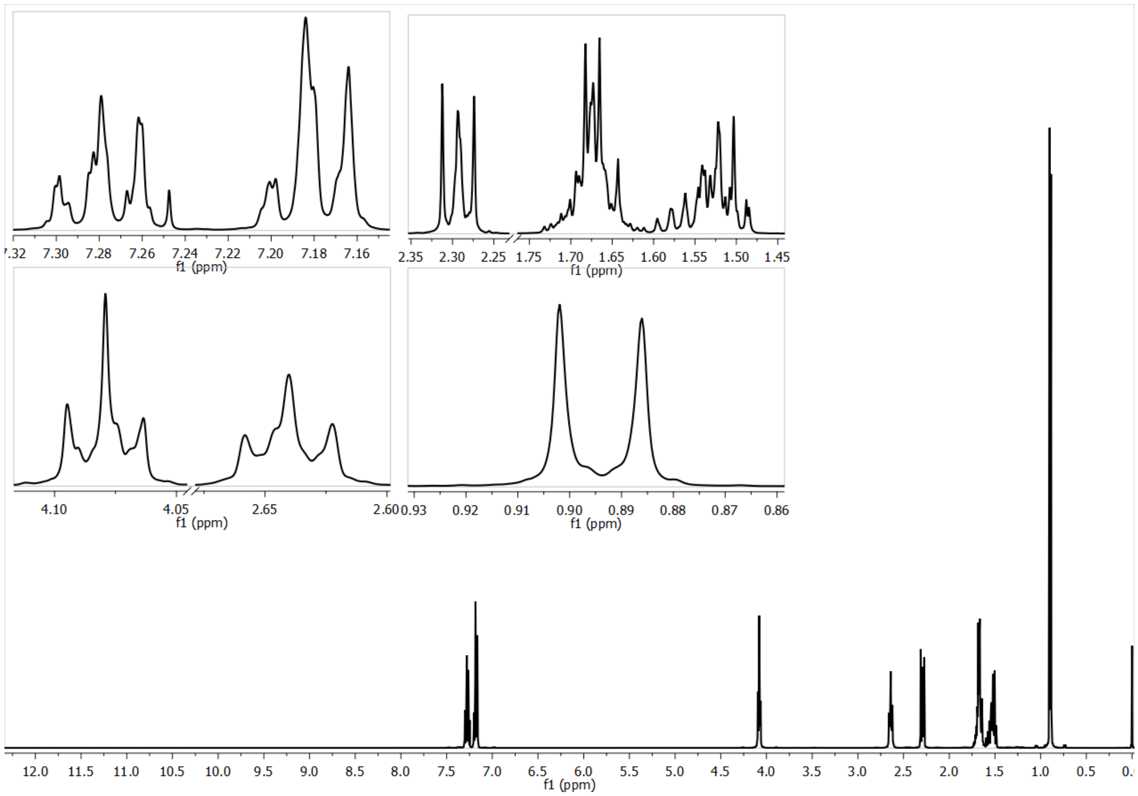

Figure S116. ^{1}H NMR spectrum of 4-phenylbutyl 4-methylpentanoate (**5g**) recorded in $CDCl_3$

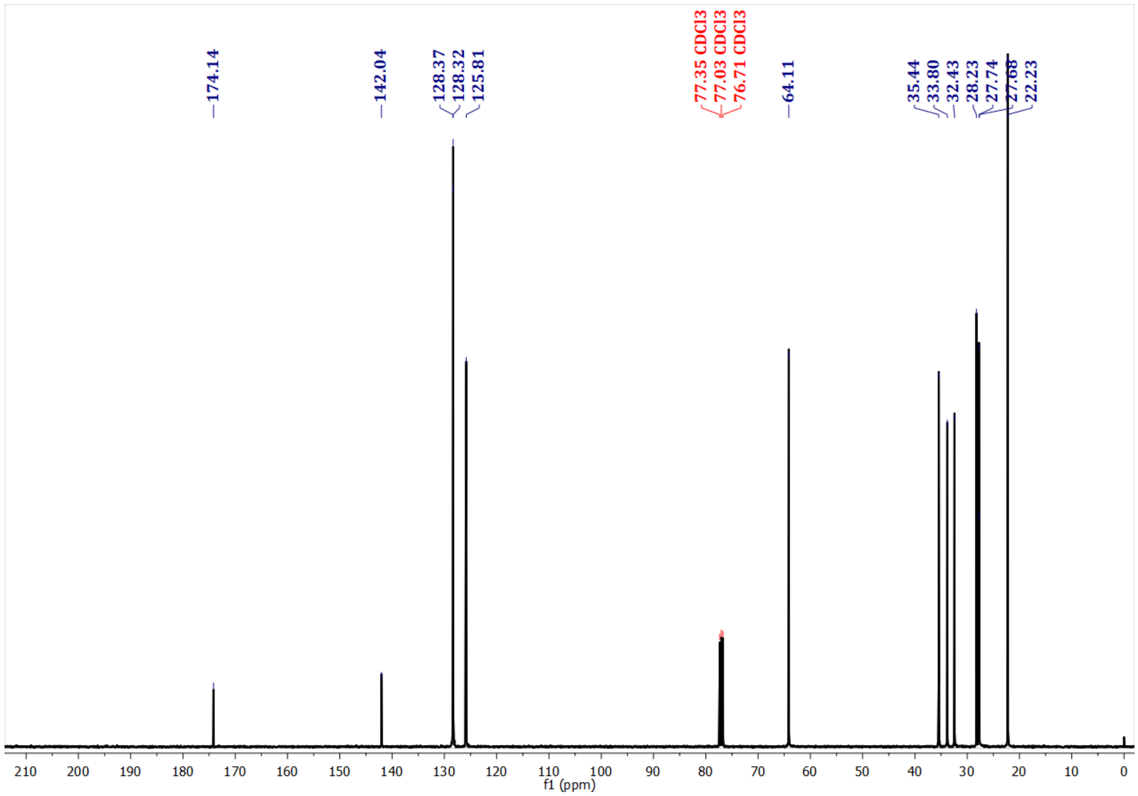

Figure S117. ^{13}C NMR spectrum of 4-phenylbutyl 4-methylpentanoate (**5g**) recorded in $CDCl_3$

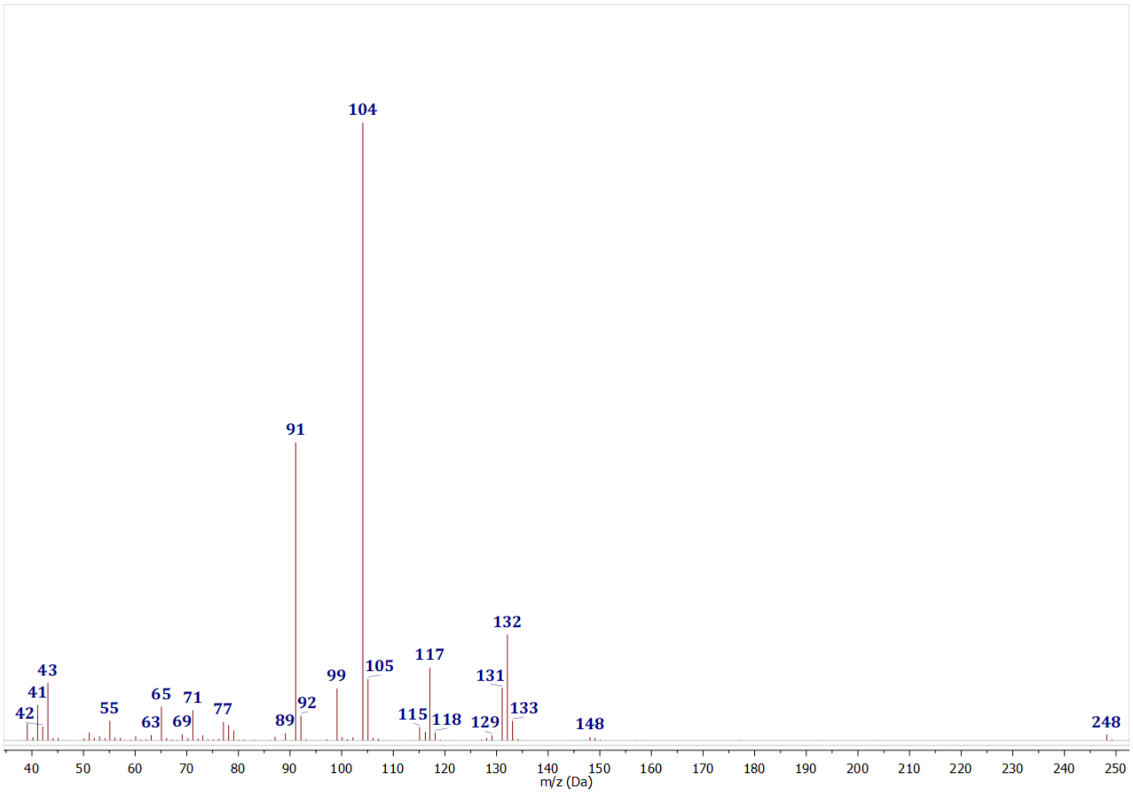

Figure S118. Mass spectrum of 4-phenylbutyl hexanoate (**5h**)

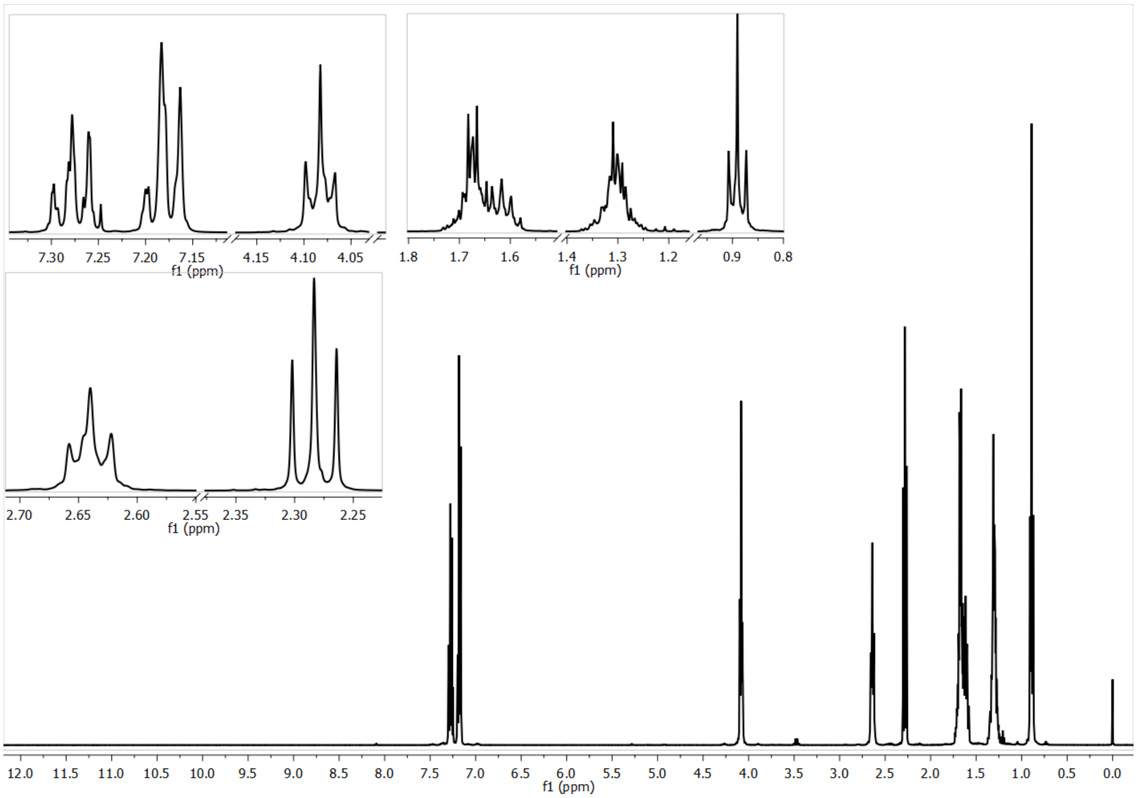

Figure S119. ^{1}H NMR spectrum of 4-phenylbutyl hexanoate (**5h**) recorded in $CDCl_3$

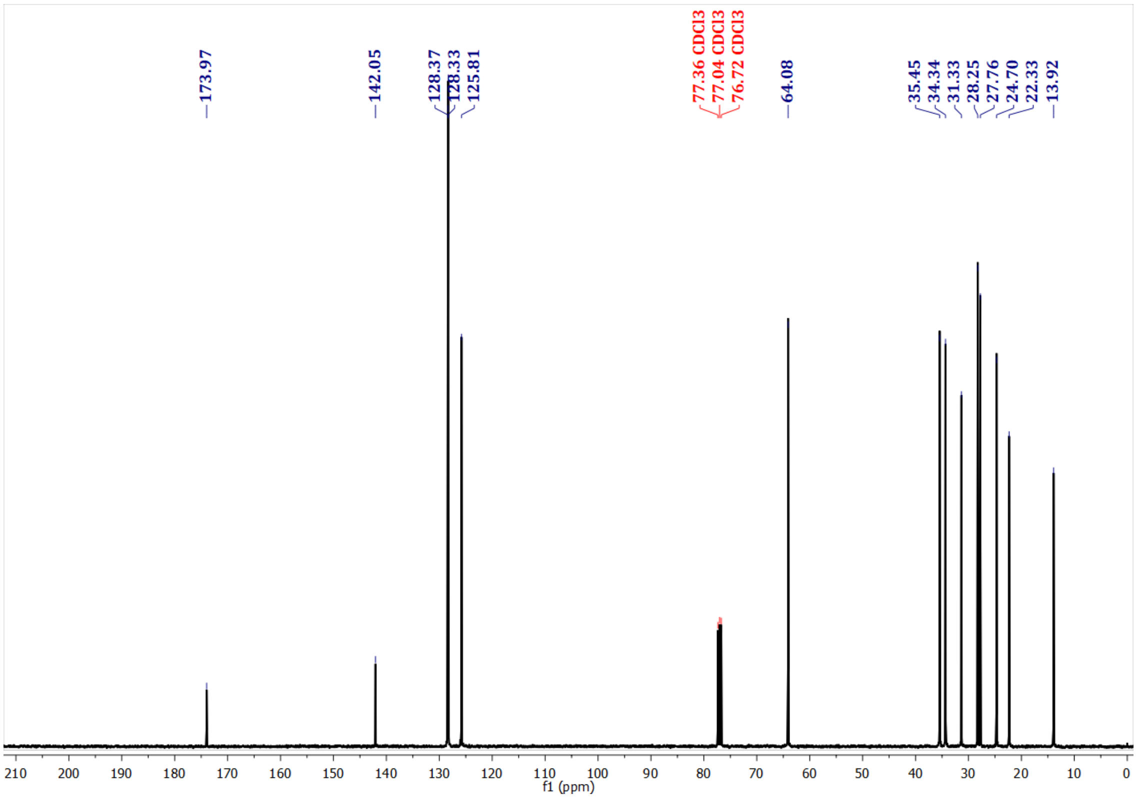

Figure S120. ^{13}C NMR spectrum of 4-phenylbutyl hexanoate (**5h**) recorded in $CDCl_3$

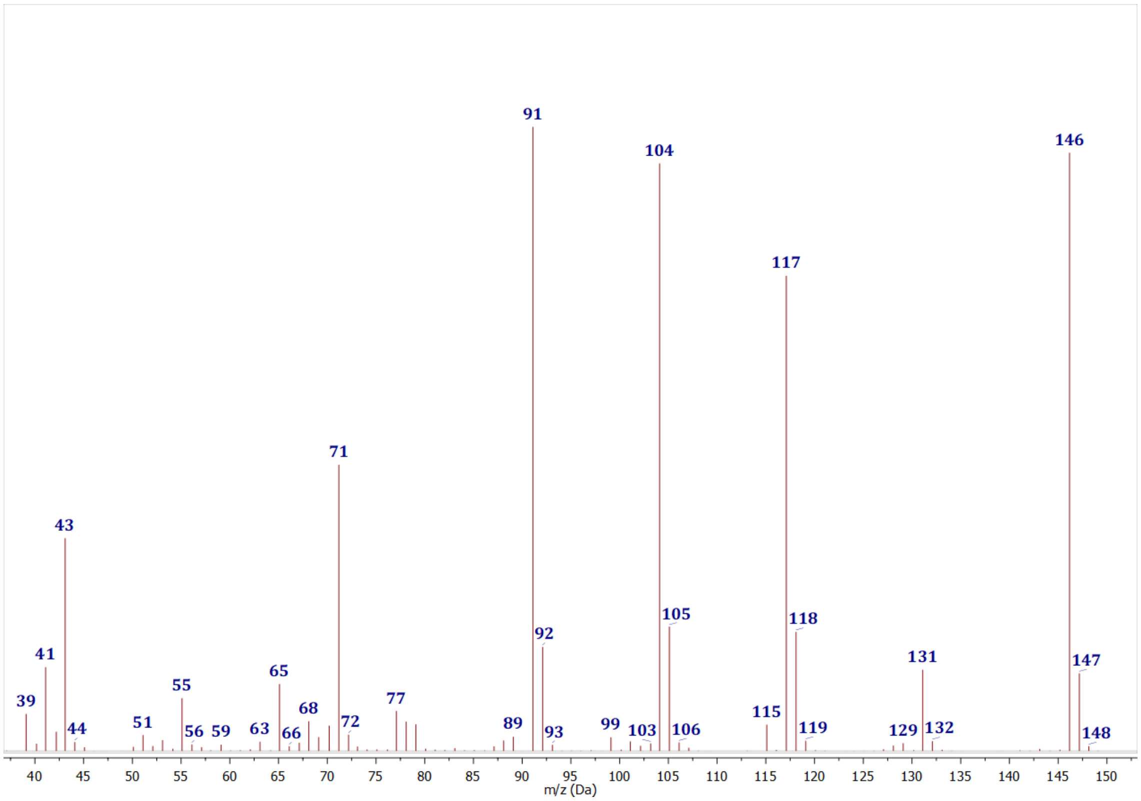

Figure S121. Mass spectrum of 5-phenylpentyl 2-ethylbutanoate (**6a**)

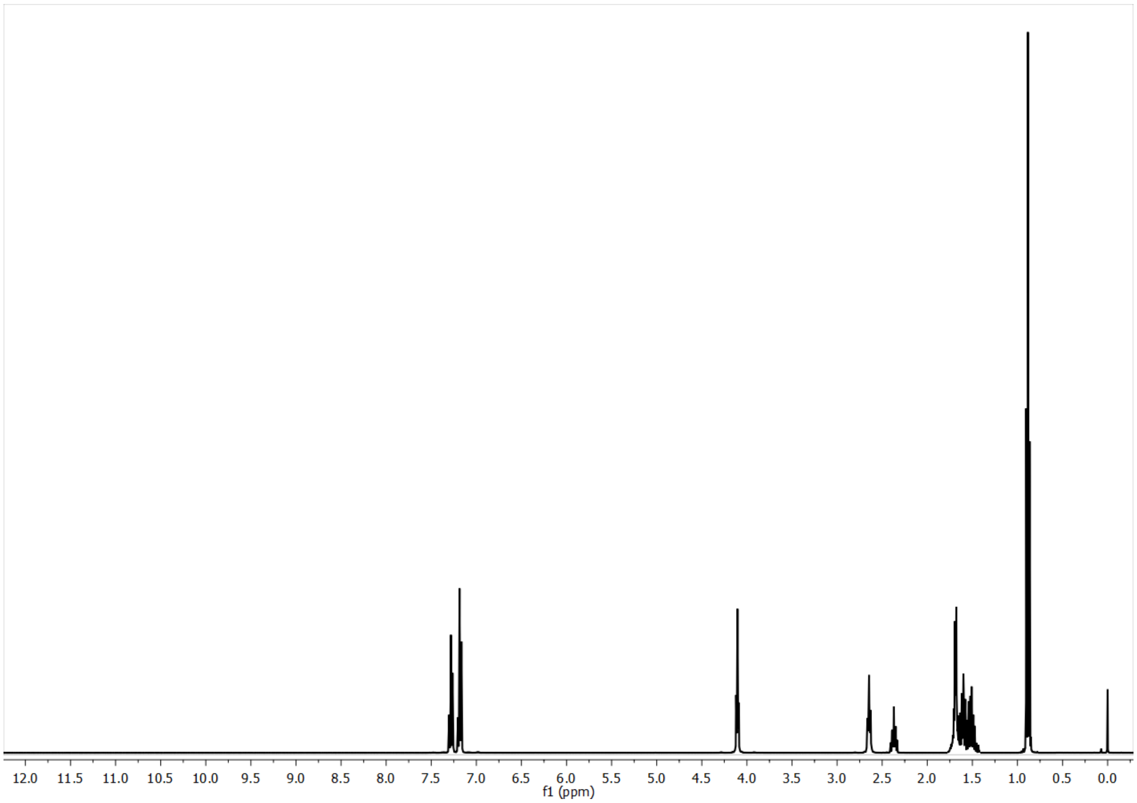

Figure S122. ^{1}H NMR spectrum of 5-phenylpentyl 2-ethylbutanoate (**6a**) recorded in $CDCl_3$

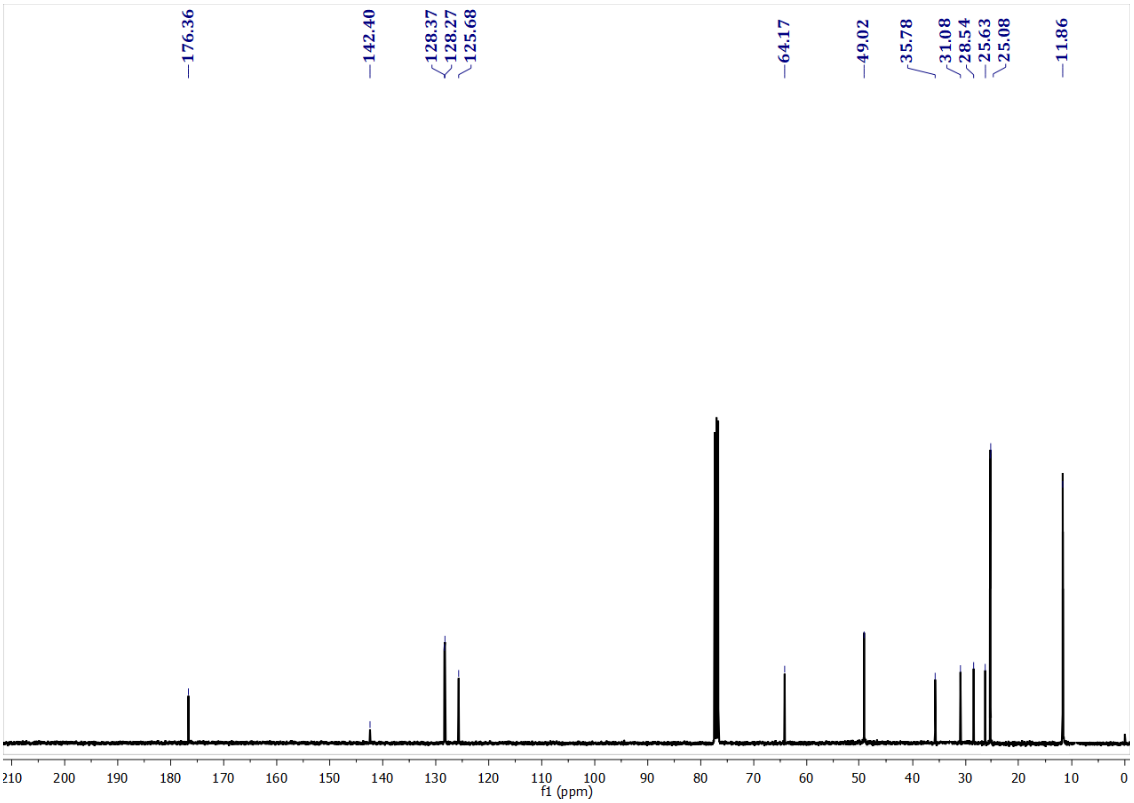

Figure S123. ^{13}C NMR spectrum of 5-phenylpentyl 2-ethylbutanoate (**6a**) recorded in $CDCl_3$

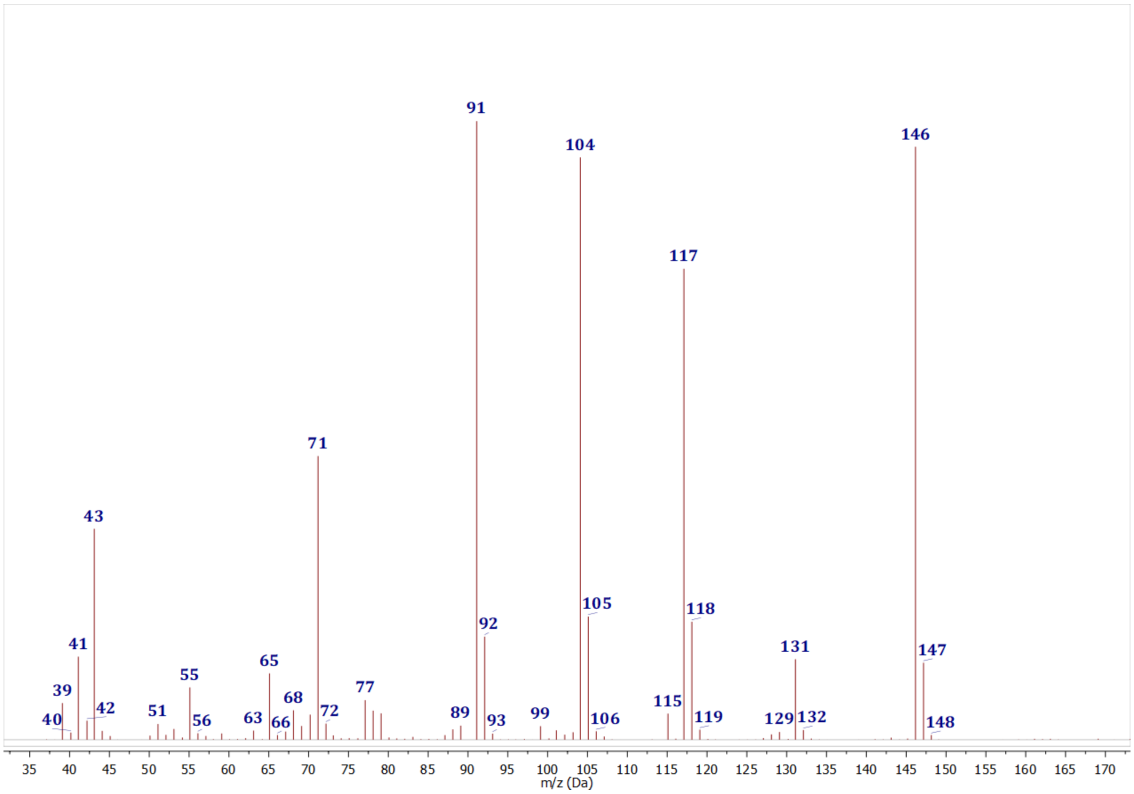

Figure S124. Mass spectrum of 5-phenylpentyl 2,2-dimethylbutanoate (**6b**)

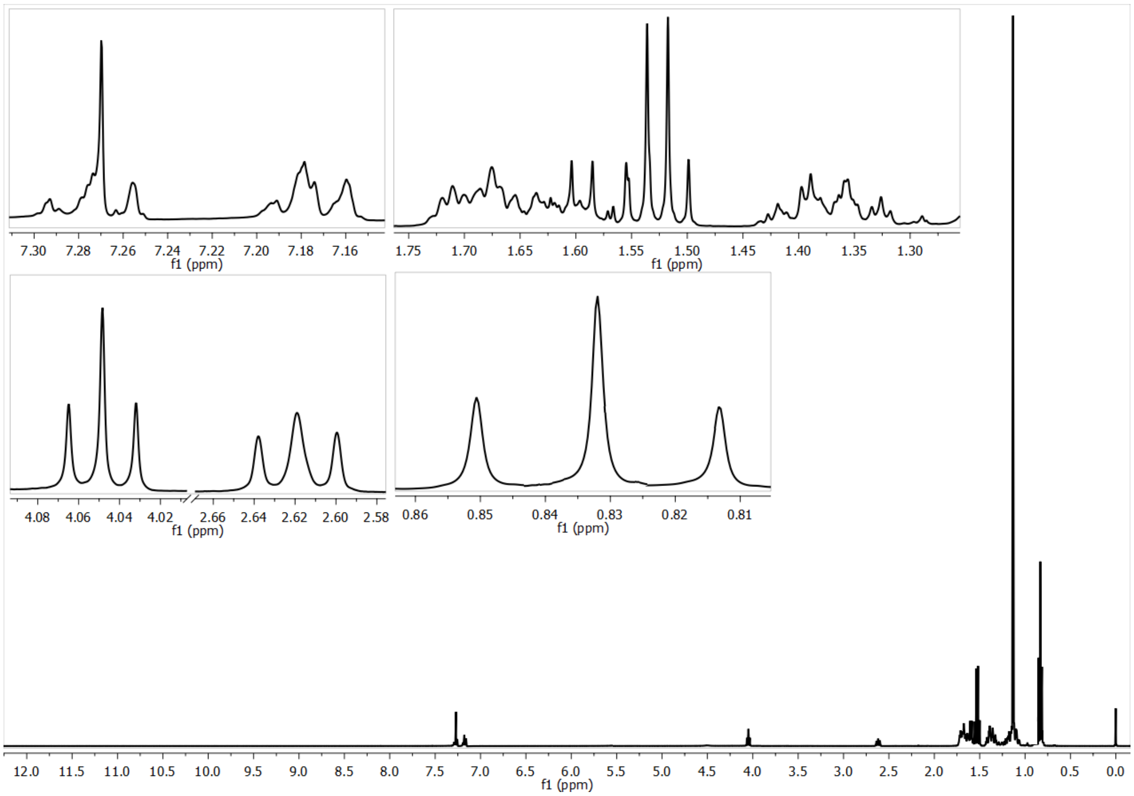

Figure S125. ^{1}H NMR spectrum of 5-phenylpentyl 2,2-dimethylbutanoate (**6b**) recorded in $CDCl_3$

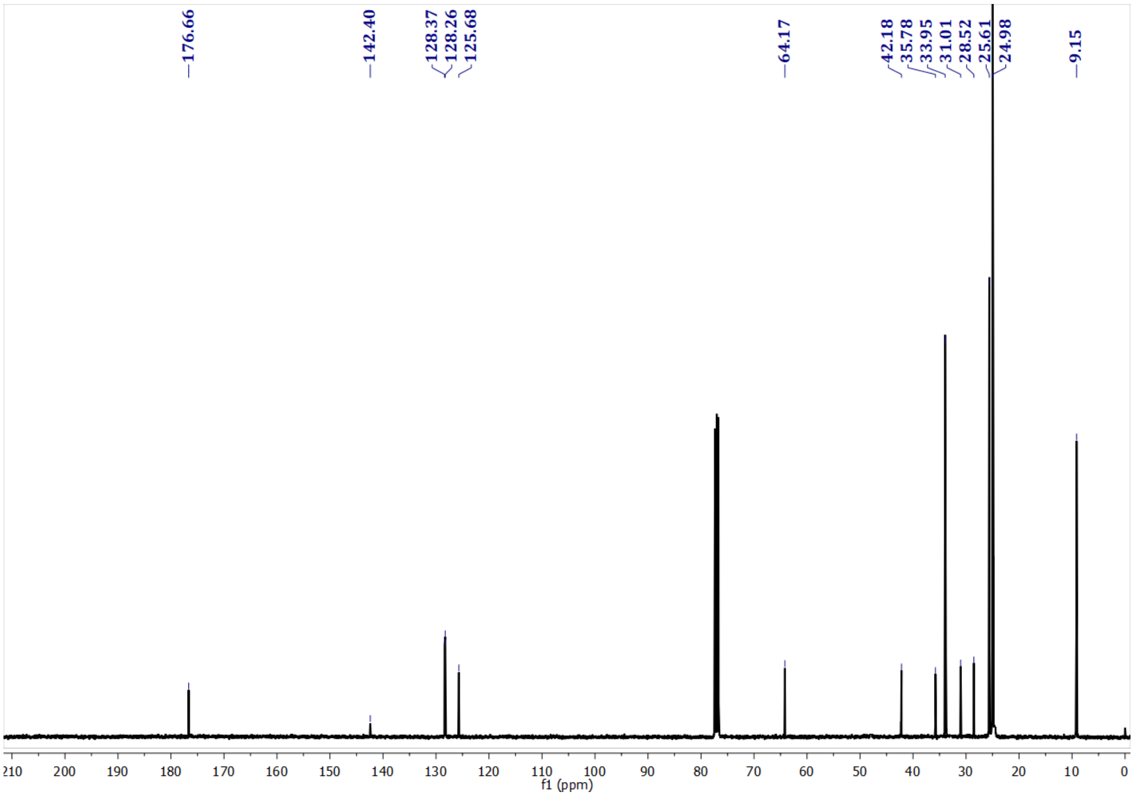

Figure S126. ^{13}C NMR spectrum of 5-phenylpentyl 2,2-dimethylbutanoate (**6b**) recorded in $CDCl_3$

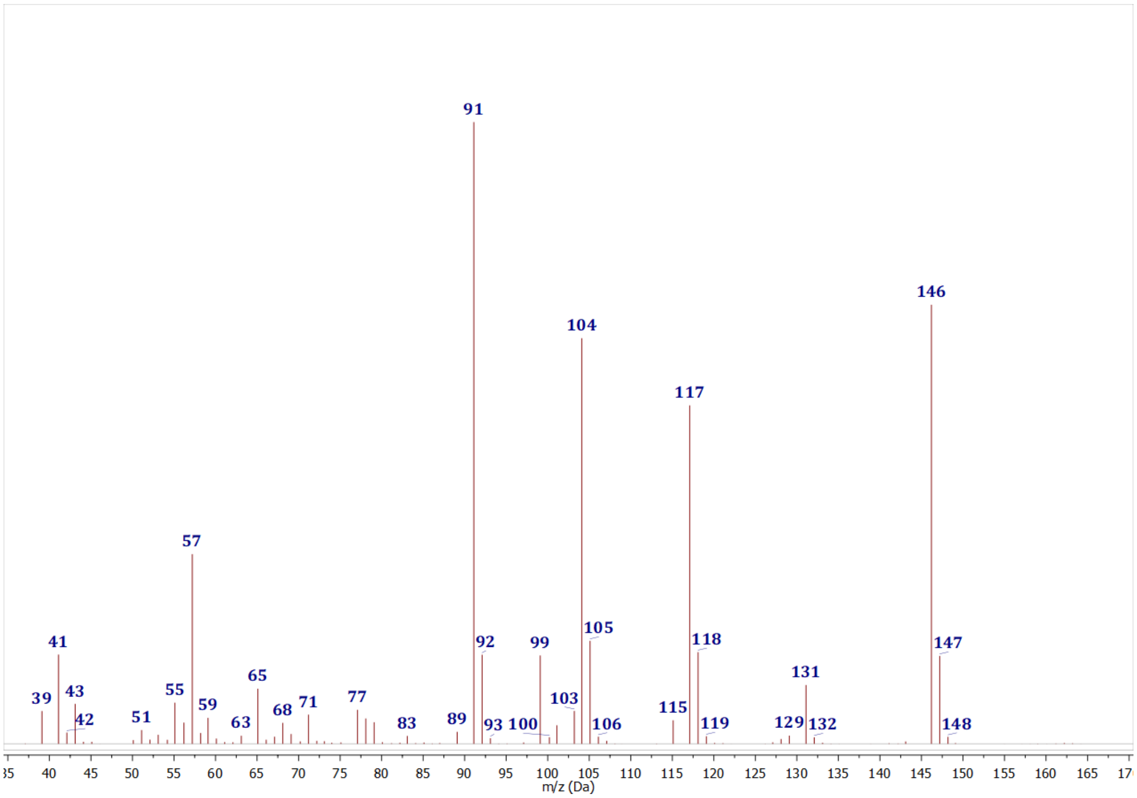

Figure S127. Mass spectrum of 5-phenylpentyl 3,3-dimethylbutanoate (**6c**)

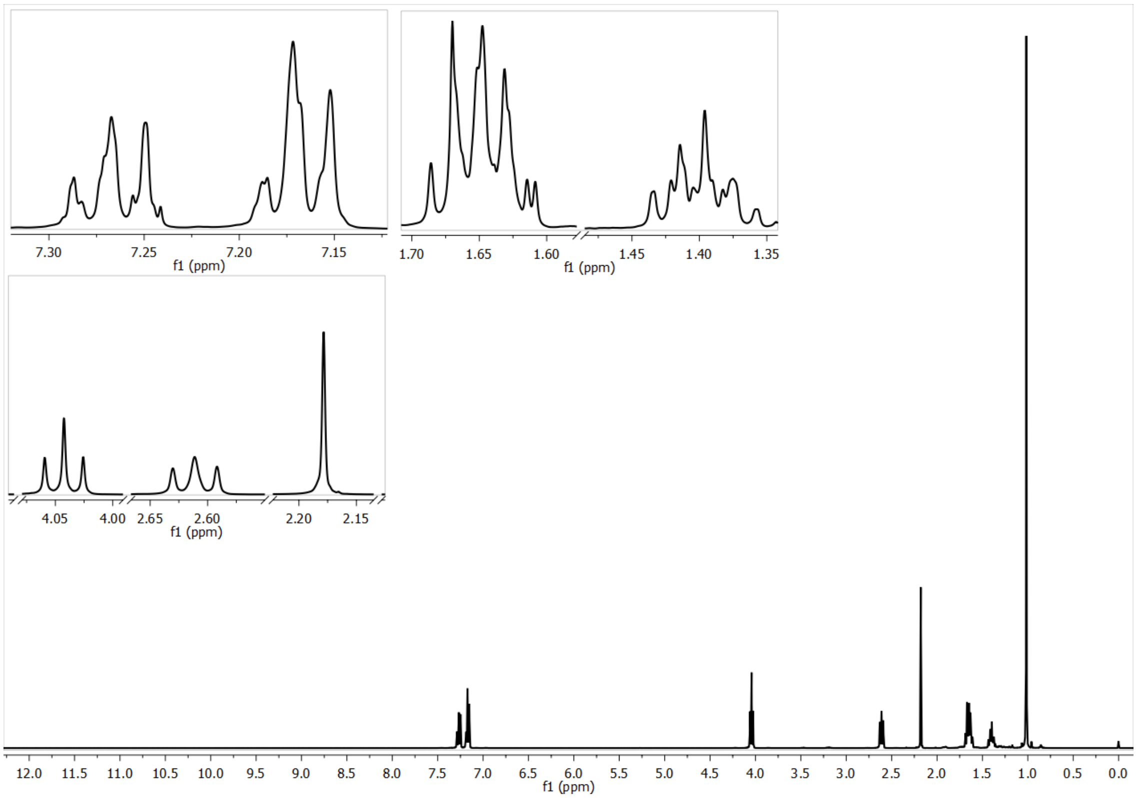

Figure S128. ^{1}H NMR spectrum of 5-phenylpentyl 3,3-dimethylbutanoate (**6c**) recorded in $CDCl_3$

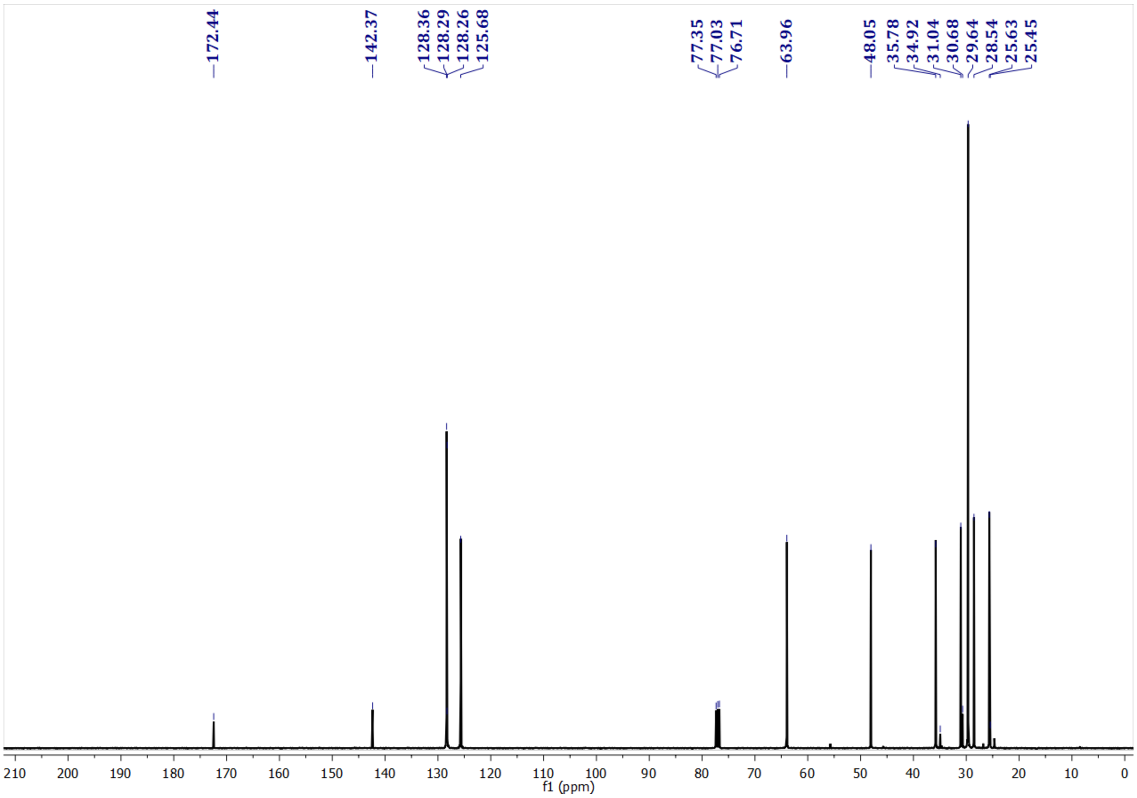

Figure S129. ^{13}C NMR spectrum of 5-phenylpentyl 3,3-dimethylbutanoate (**6c**) recorded in $CDCl_3$

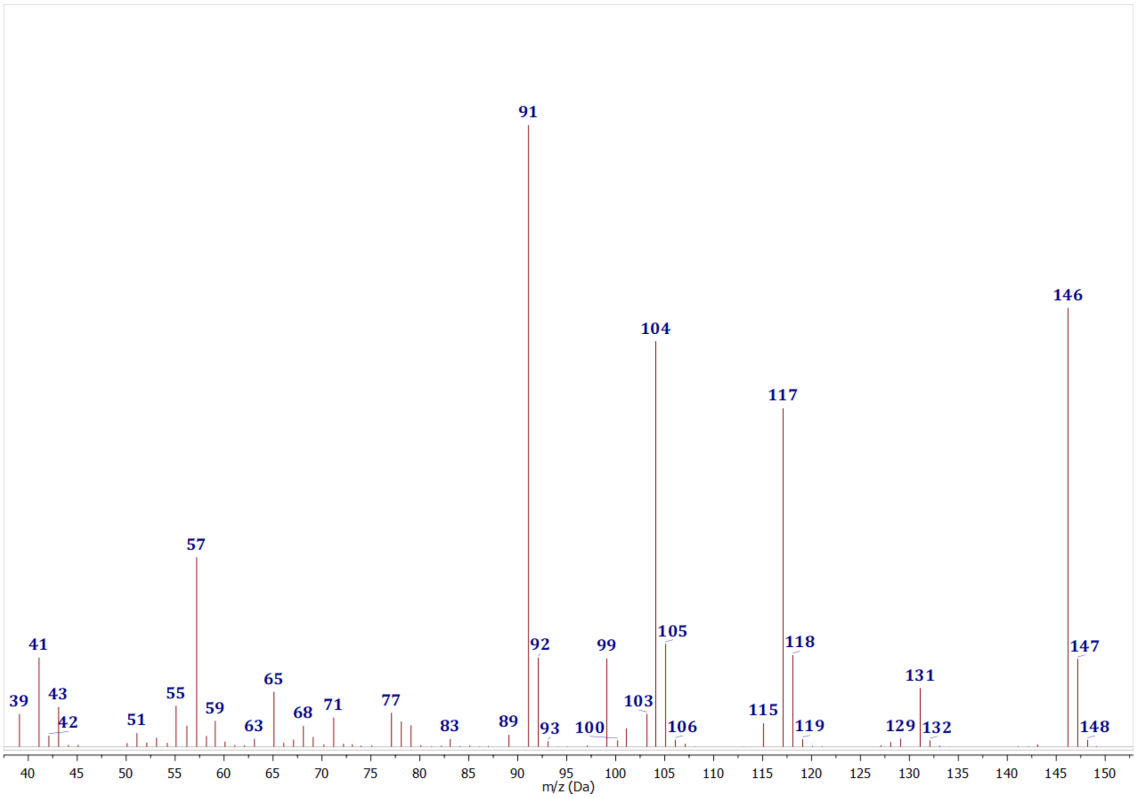

Figure S130. Mass spectrum of 5-phenylpentyl 2,3-dimethylbutanoate (**6d**)

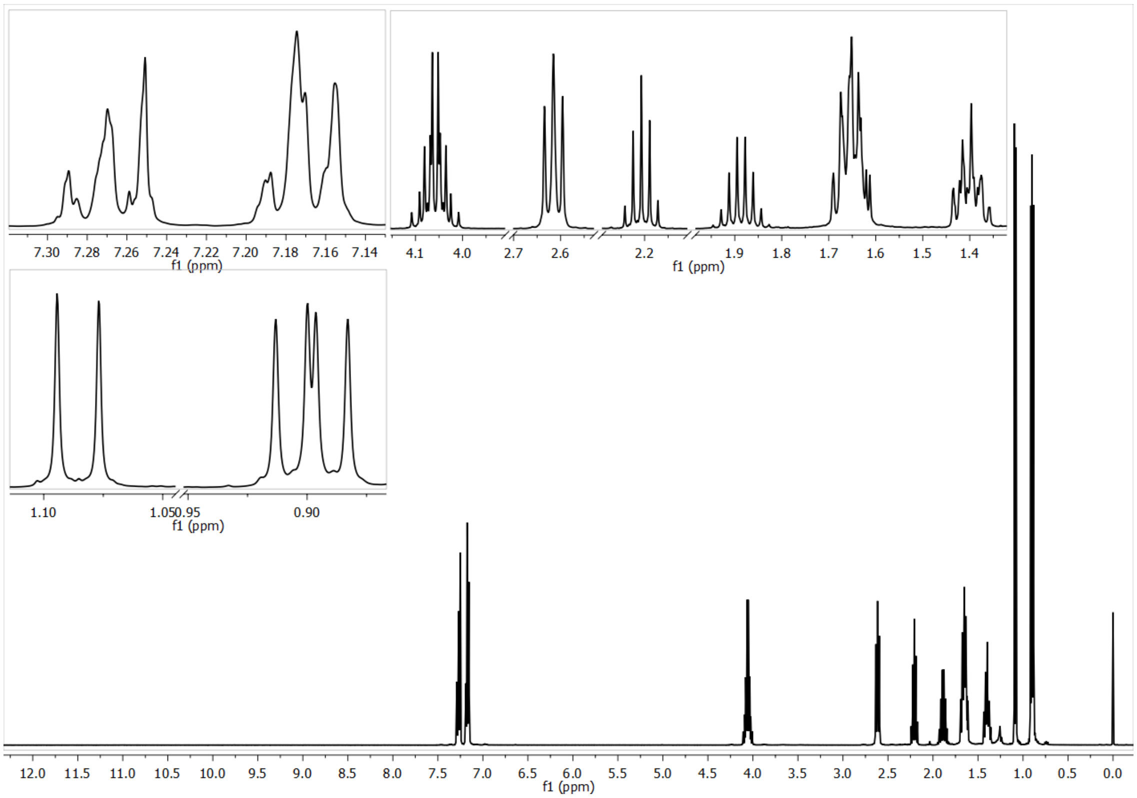

Figure S131. ^1H NMR spectrum of 5-phenylpentyl 2,3-dimethylbutanoate (**6d**) recorded in $CDCl_3$

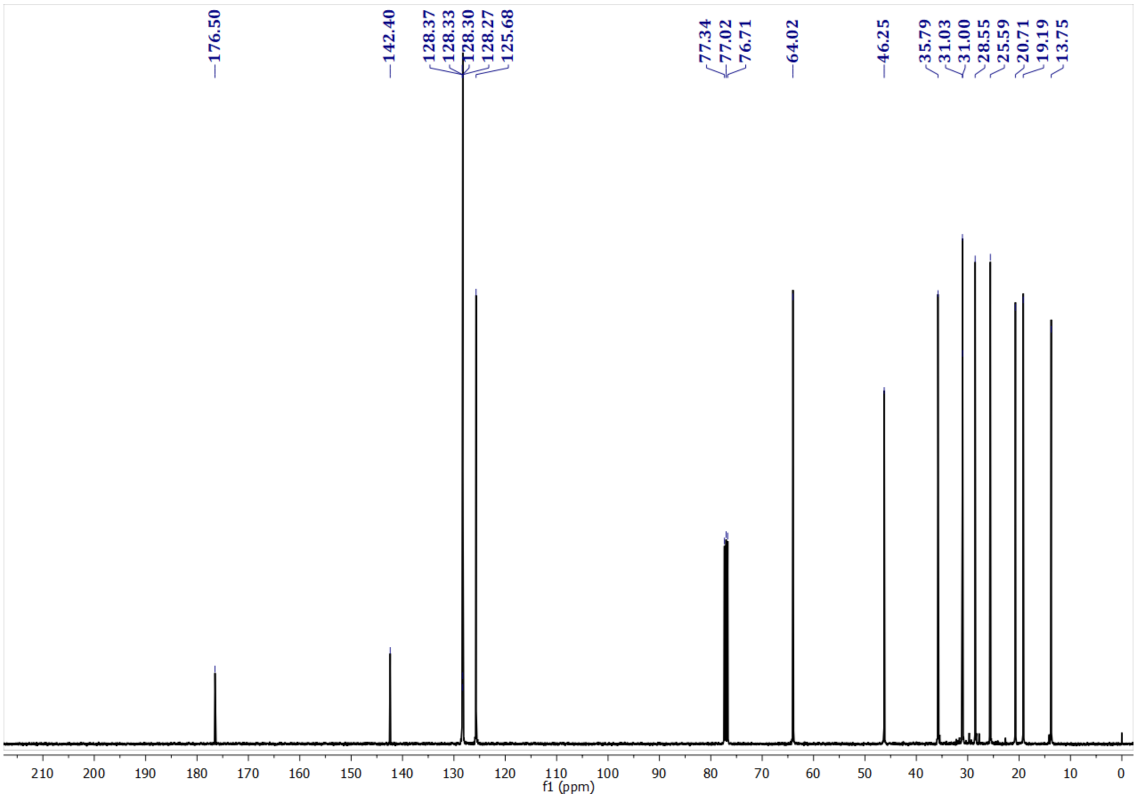

Figure S132. ^{13}C NMR spectrum of 5-phenylpentyl 2,3-dimethylbutanoate (**6d**) recorded in $CDCl_3$

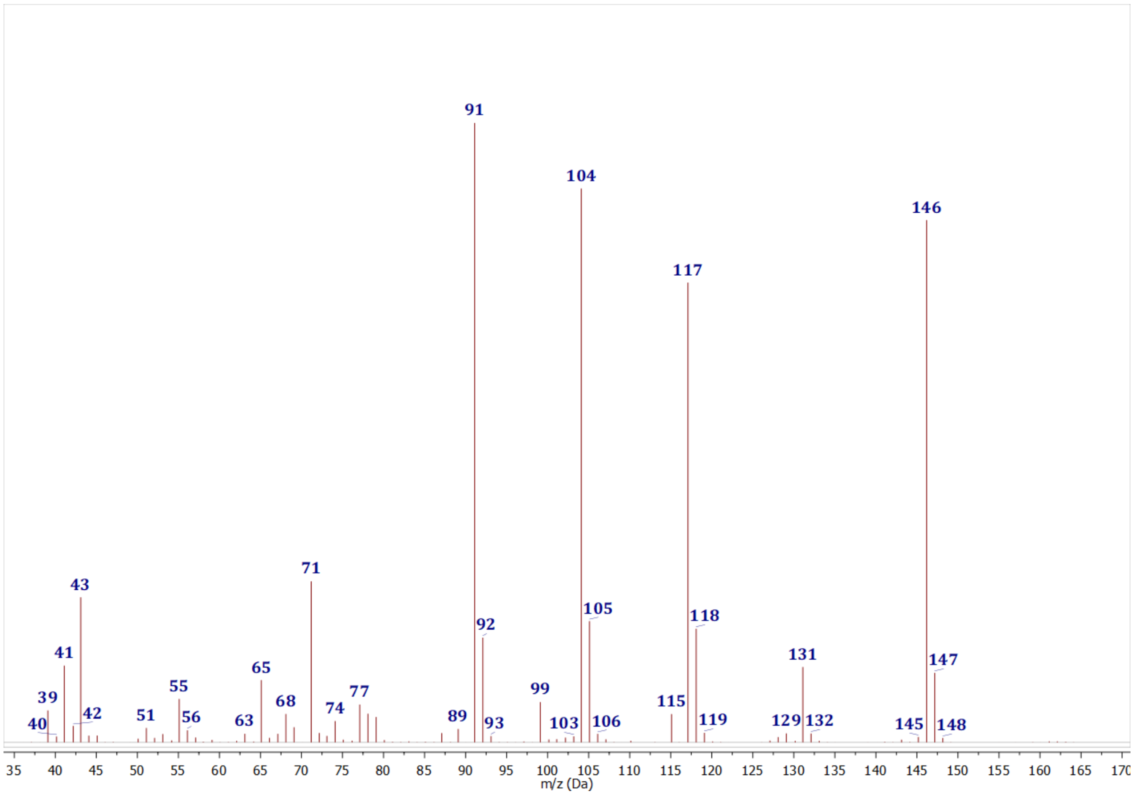

Figure S133. Mass spectrum of 5-phenylpentyl 2-methylpentanoate (**6e**)

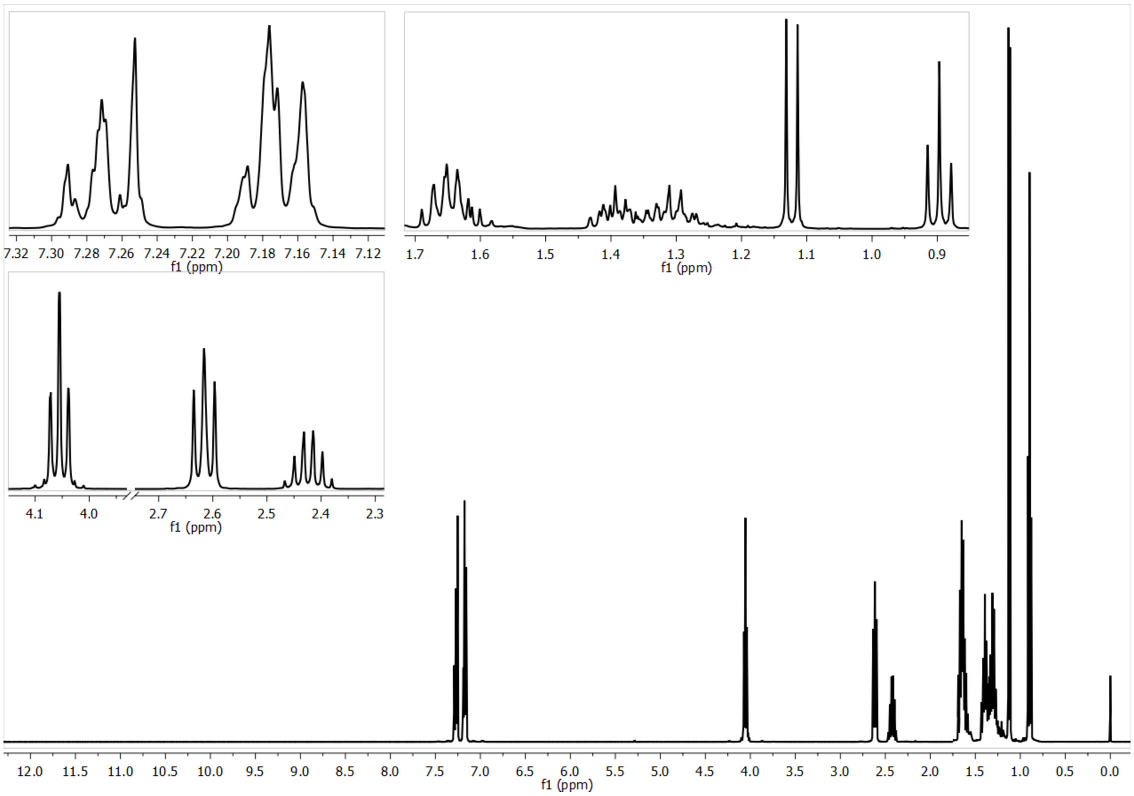

Figure S134. ^{1}H NMR spectrum of 5-phenylpentyl 2-methylpentanoate (**6e**) recorded in $CDCl_3$

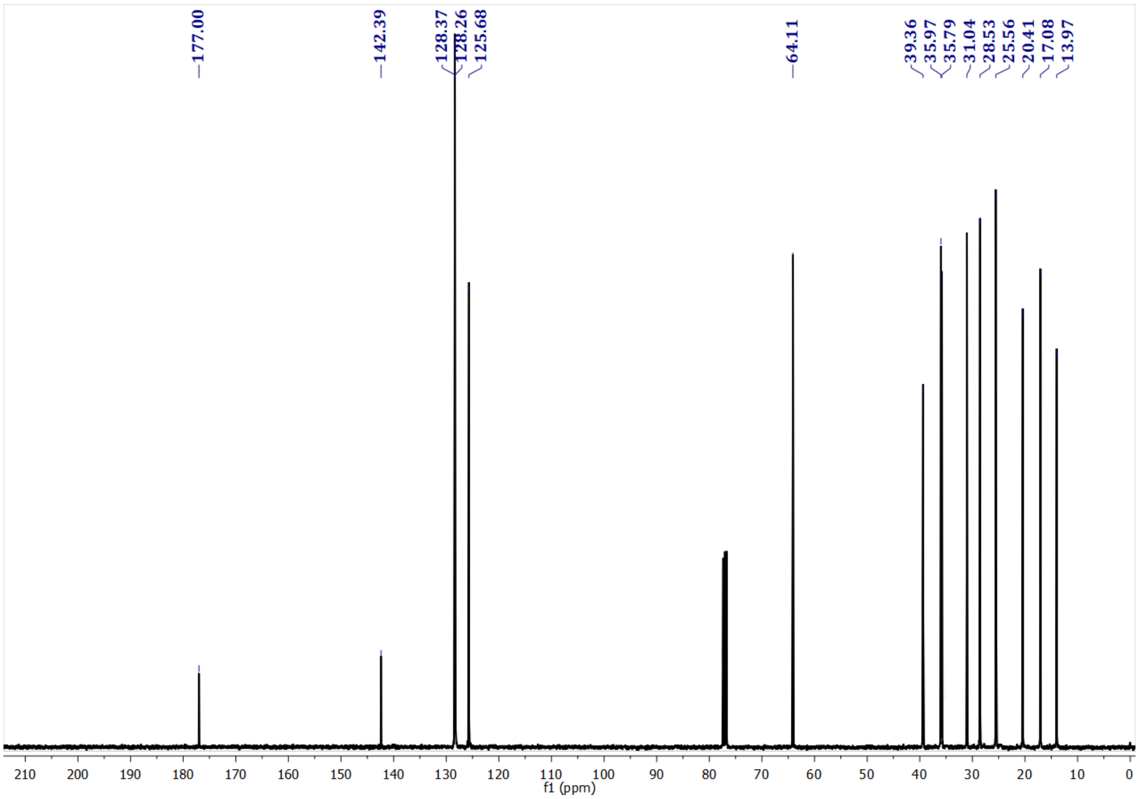

Figure S135. ^{13}C NMR spectrum of 5-phenylpentyl 2-methylpentanoate (**6e**) recorded in $CDCl_3$

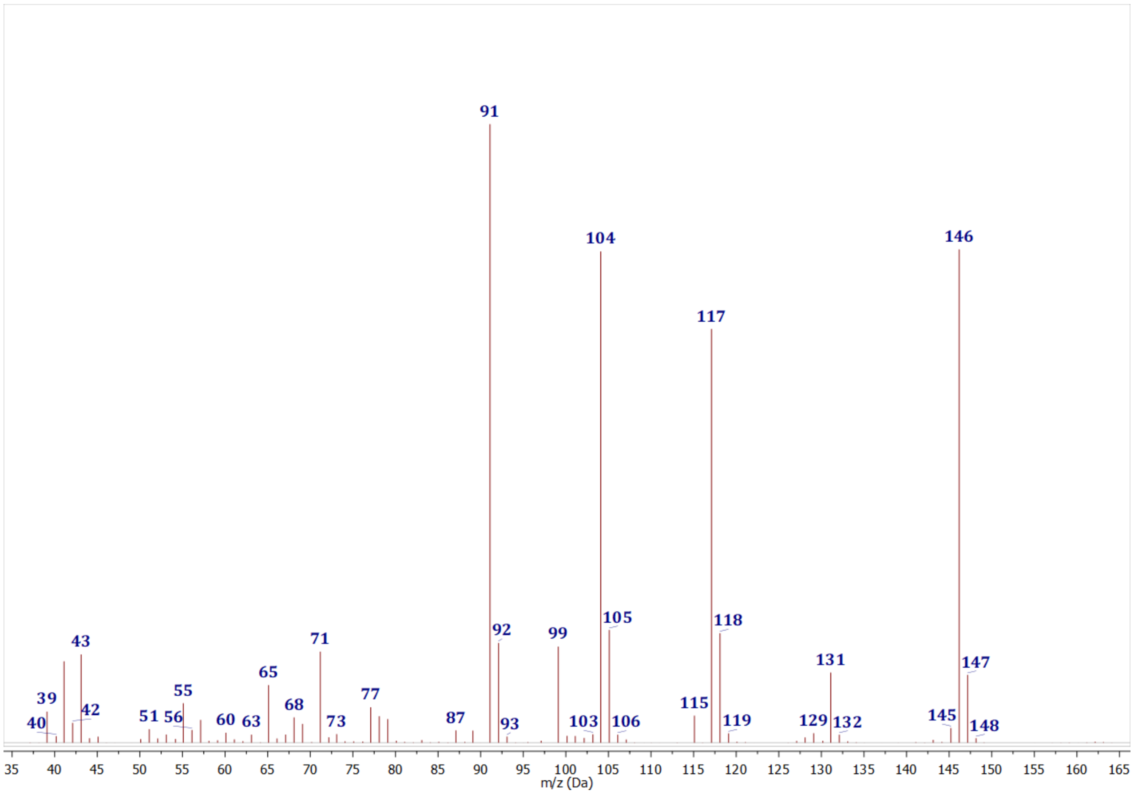

Figure S136. Mass spectrum of 5-phenylpentyl 3-methylpentanoate (**6f**)

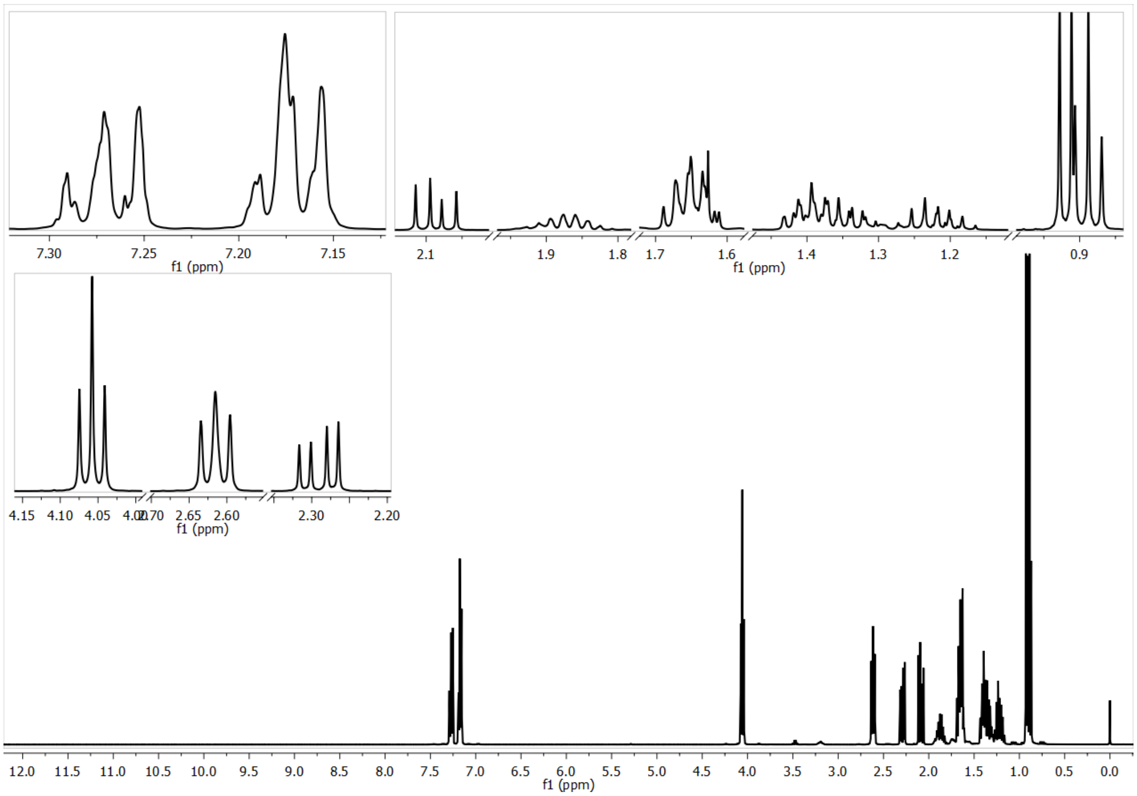

Figure S137. ^{1}H NMR spectrum of 5-phenylpentyl 3-methylpentanoate (**6f**) recorded in $CDCl_3$

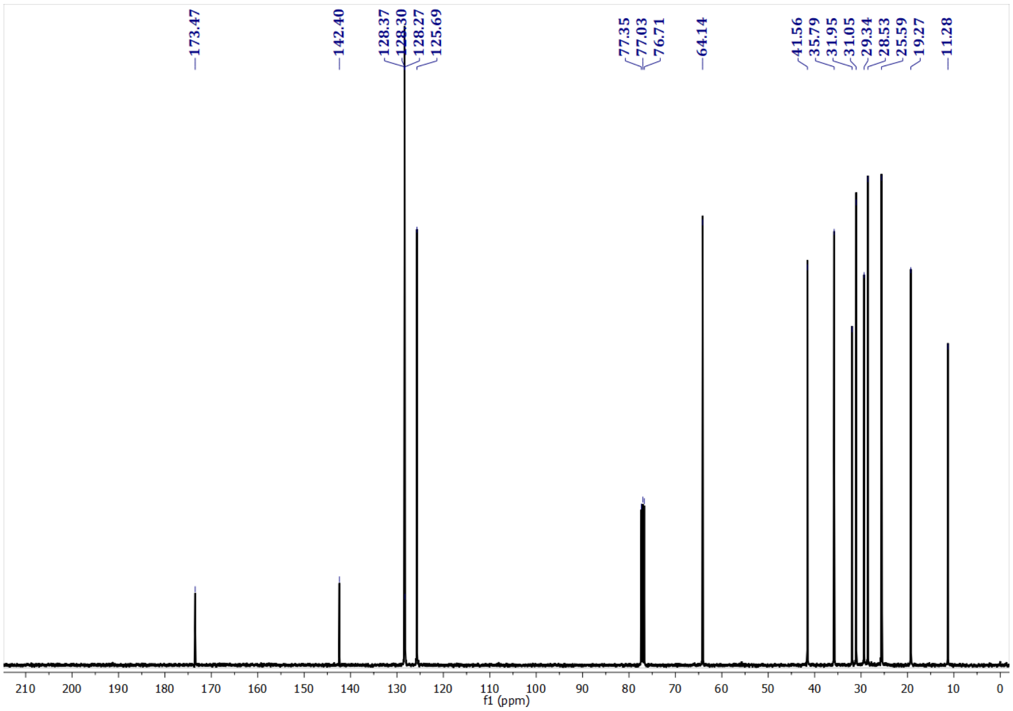

Figure S138. ^{13}C NMR spectrum of 5-phenylpentyl 3-methylpentanoate (**6f**) recorded in $CDCl_3$

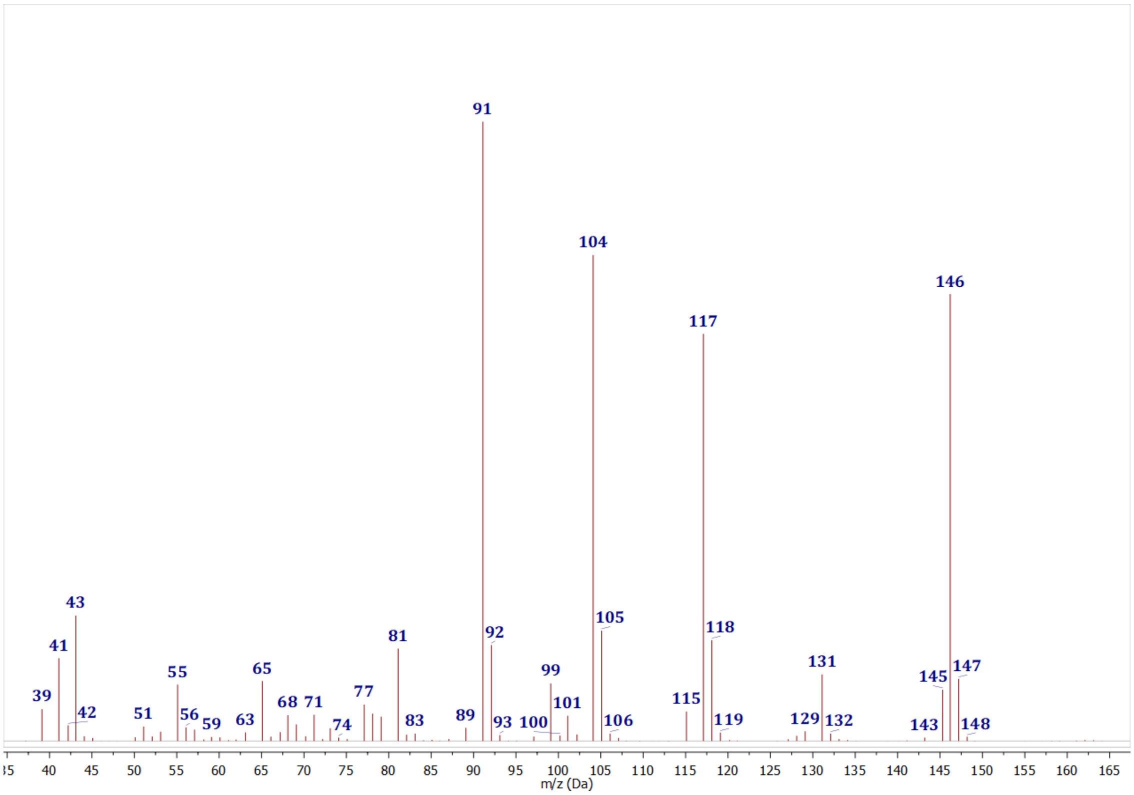

Figure S139. Mass spectrum of 5-phenylpentyl 4-methylpentanoate (**6g**)

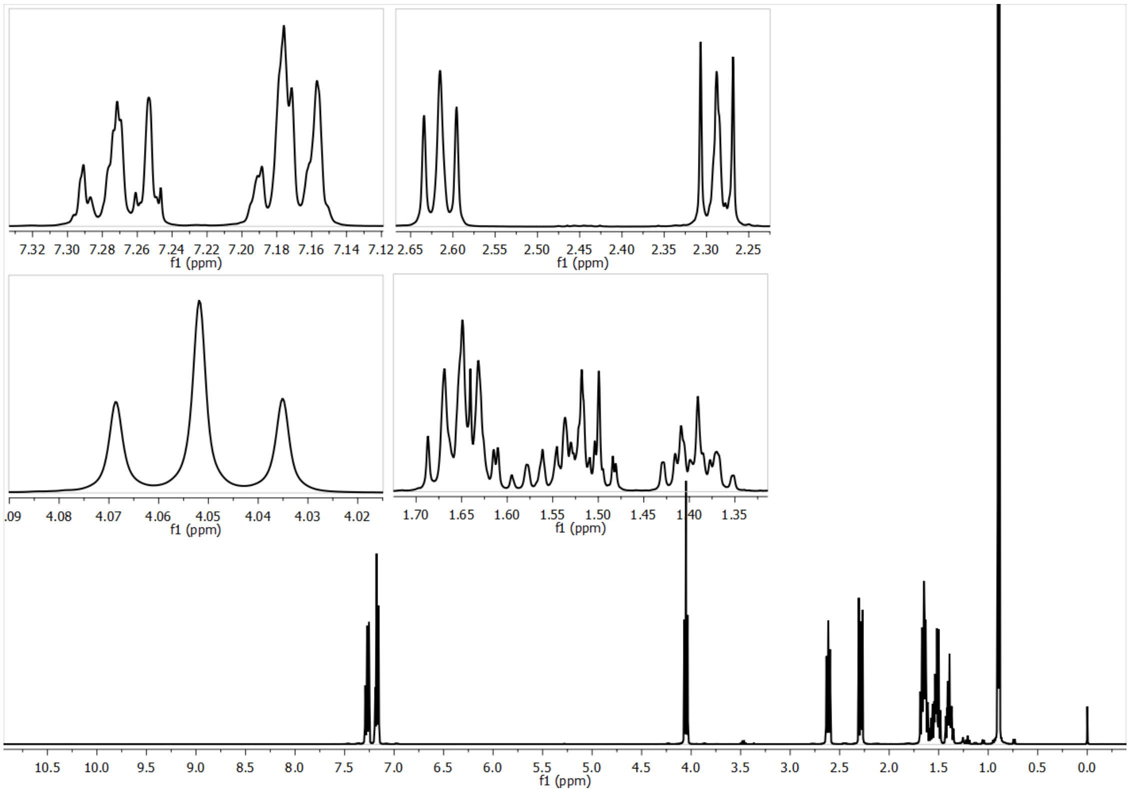

Figure S140. ^{1}H NMR spectrum of 5-phenylpentyl 4-methylpentanoate (**6g**) recorded in $CDCl_3$

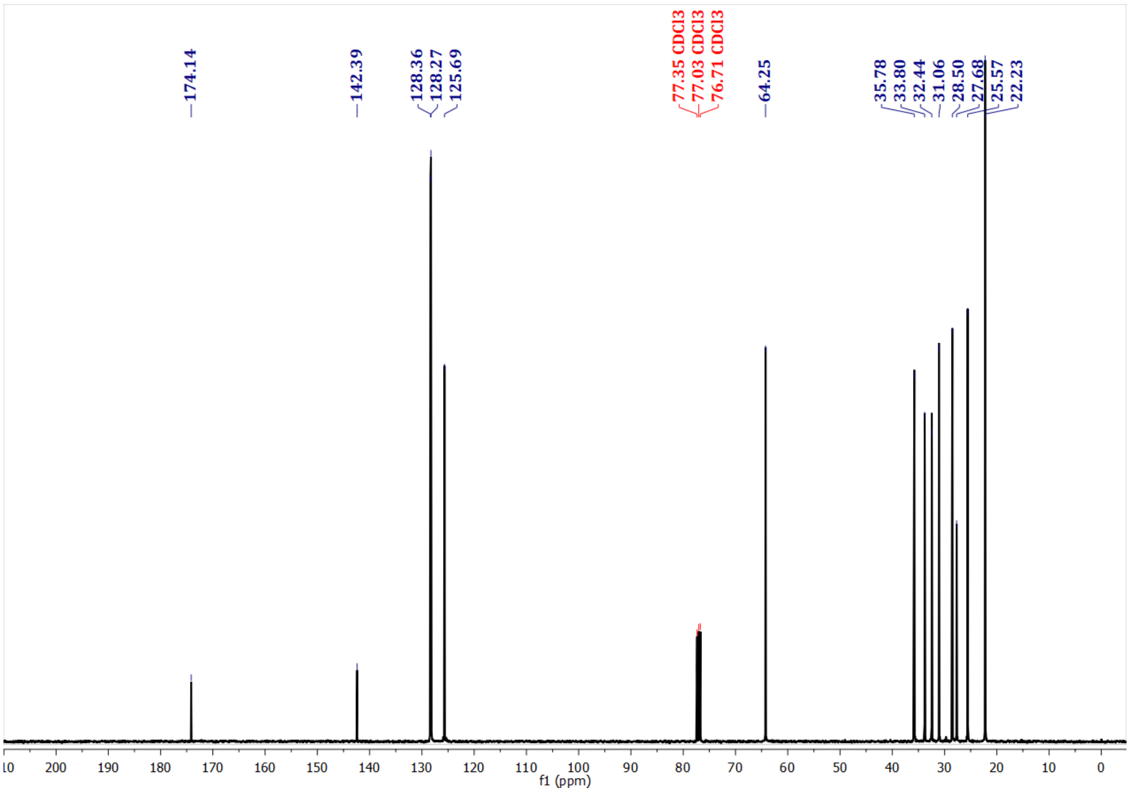

Figure S141. ^{13}C NMR spectrum of 5-phenylpentyl 4-methylpentanoate (**6g**) recorded in $CDCl_3$

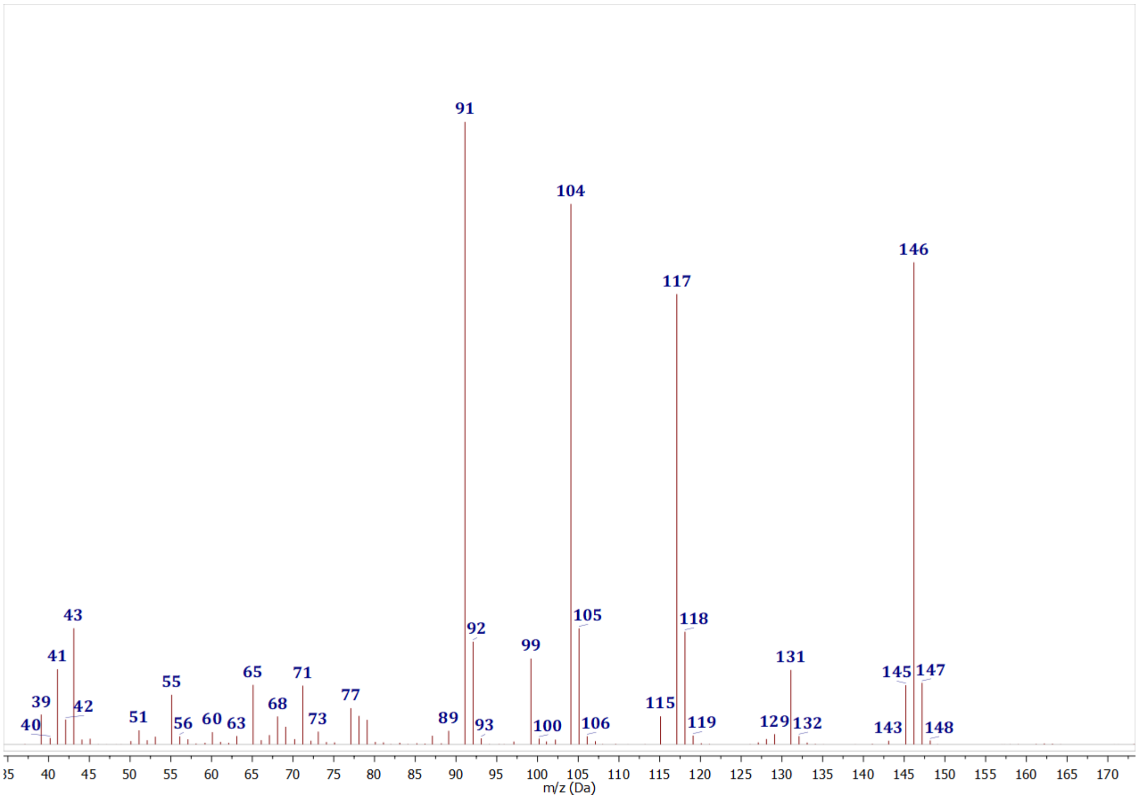

Figure S142. Mass spectrum of 5-phenylpentyl hexanoate (**6h**)

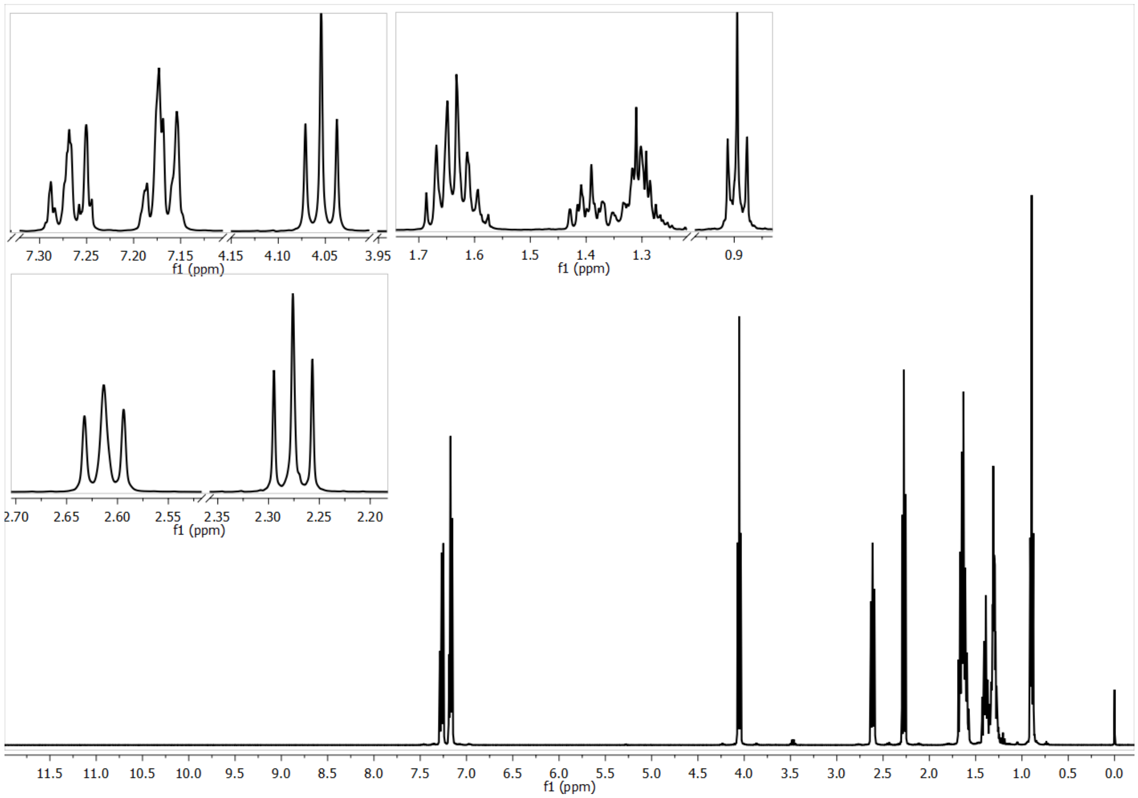

Figure S143. ^{1}H NMR spectrum of 5-phenylpentyl hexanoate (**6h**) recorded in $CDCl_3$

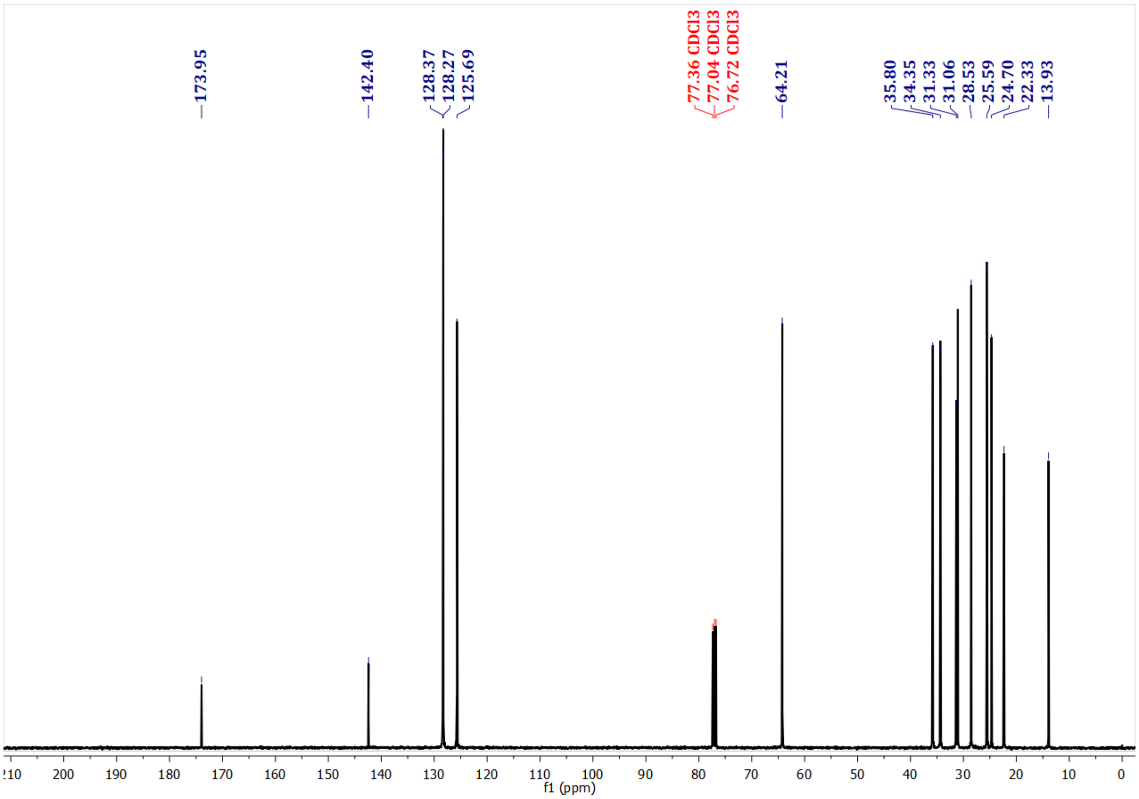

Figure S144. ^{13}C NMR spectrum of 5-phenylpentyl hexanoate (**6h**) recorded in $CDCl_3$

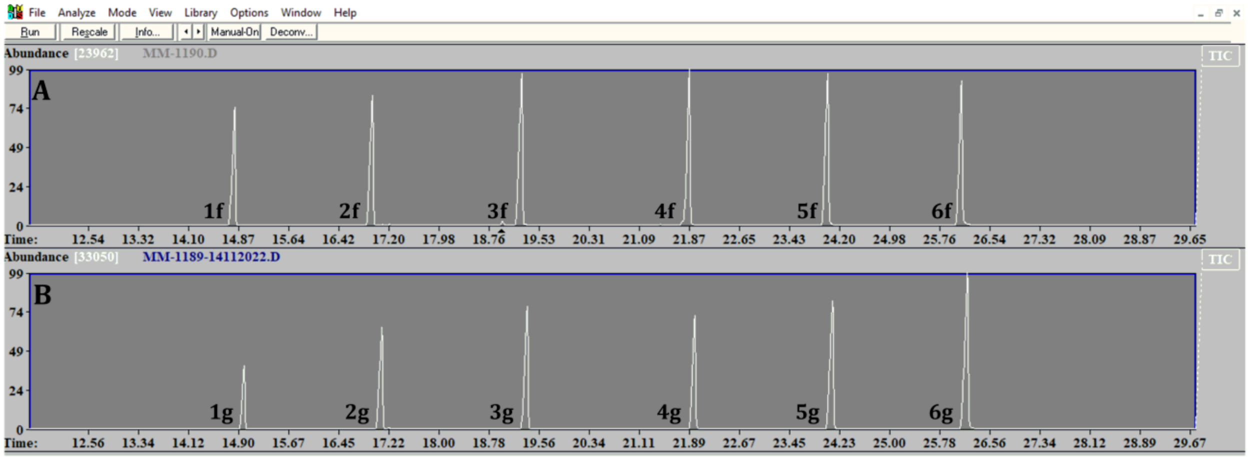

Figure S145. Typical total ion current (TIC) chromatogram of a mixture of synthesized (**A**) 3-methylpentanoates and (**B**) 4-methylpentanoates

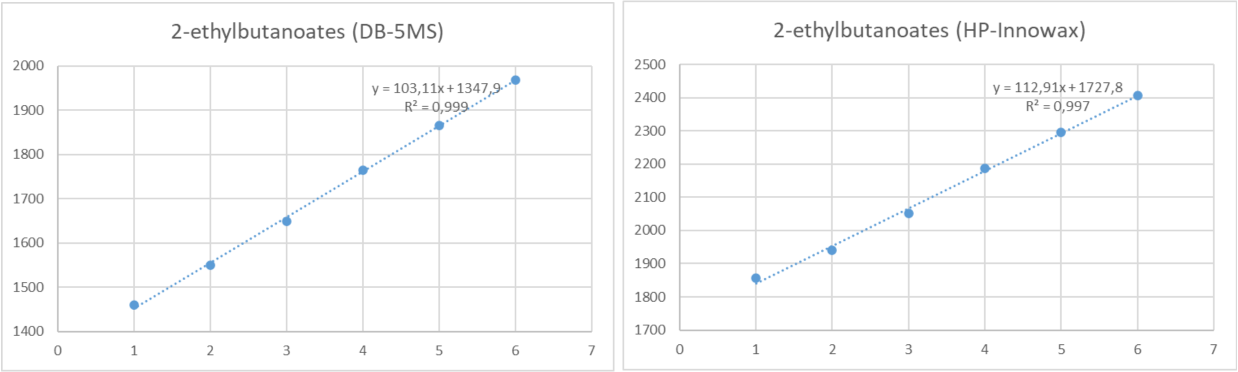

Figure S146. The dependence of RI values of the esters of the same acid (2-ethylbutanoic acid) and phenol (**1**), benzyl alcohol (**2**), phenethyl alcohol (**3**), 3-phenylpropanol (**4**), 4-phenylbutanol (**5**), and 5-phenylpentanol (**6**)

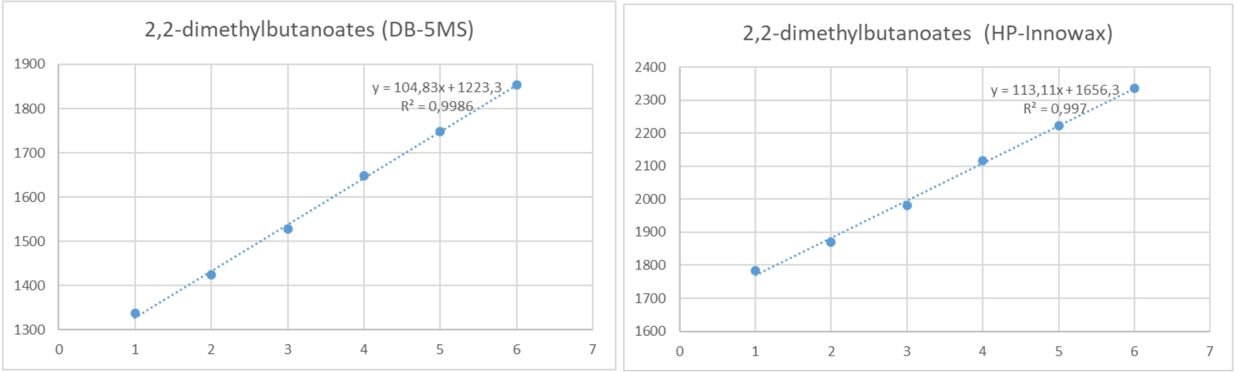

Figure S147. The dependence of RI values of the esters of the same acid (2,2-dimethylbutanoic acid) and phenol (**1**), benzyl alcohol (**2**), phenethyl alcohol (**3**), 3-phenylpropanol (**4**), 4-phenylbutanol (**5**), and 5-phenylpentanol (**6**)

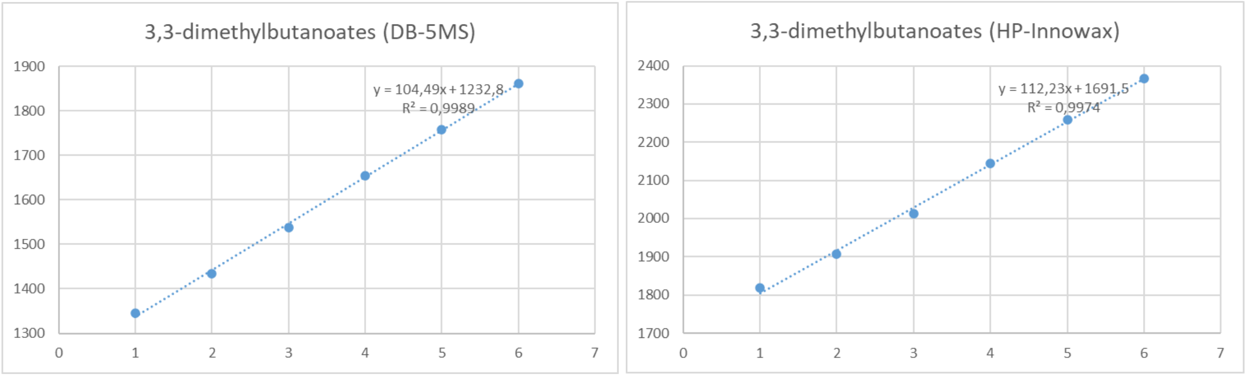

Figure S148. The dependence of RI values of the esters of the same acid (3,3-dimethylbutanoic acid) and phenol (**1**), benzyl alcohol (**2**), phenethyl alcohol (**3**), 3-phenylpropanol (**4**), 4-phenylbutanol (**5**), and 5-phenylpentanol (**6**)

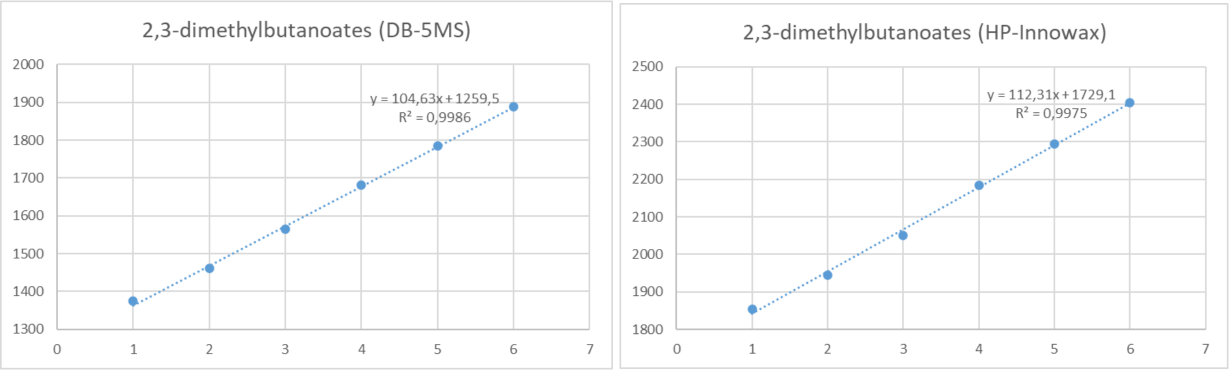

Figure S149. The dependence of RI values of the esters of the same acid (2,3-dimethylbutanoic acid) and phenol (**1**), benzyl alcohol (**2**), phenethyl alcohol (**3**), 3-phenylpropanol (**4**), 4-phenylbutanol (**5**), and 5-phenylpentanol (**6**)

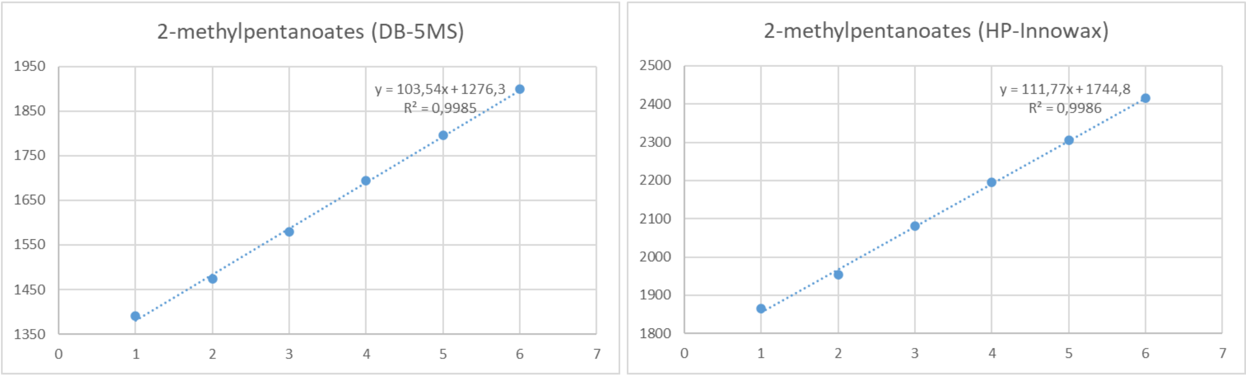

Figure S150. The dependence of RI values of the esters of the same acid (2-methylpentanoic acid) and phenol (**1**), benzyl alcohol (**2**), phenethyl alcohol (**3**), 3-phenylpropanol (**4**), 4-phenylbutanol (**5**), and 5-phenylpentanol (**6**)

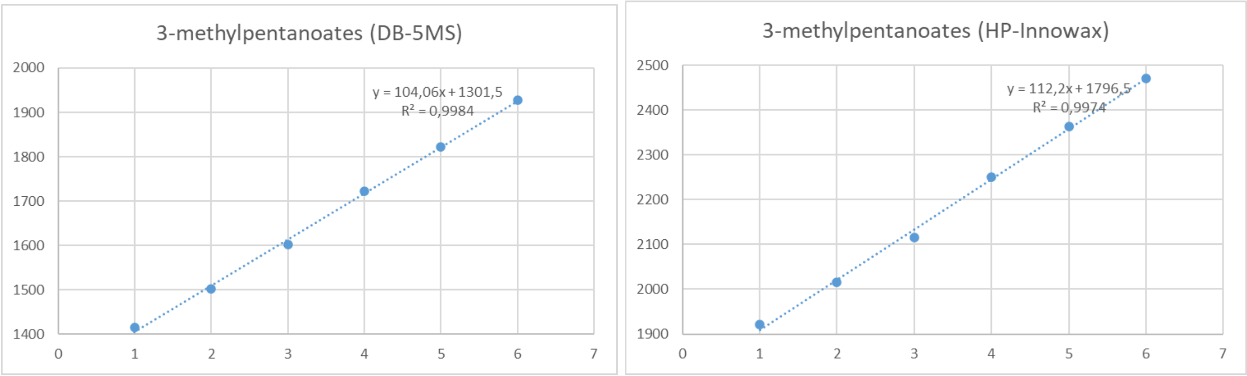

Figure S151. The dependence of RI values of the esters of the same acid (3-methylpentanoic acid) and phenol (**1**), benzyl alcohol (**2**), phenethyl alcohol (**3**), 3-phenylpropanol (**4**), 4-phenylbutanol (**5**), and 5-phenylpentanol (**6**)

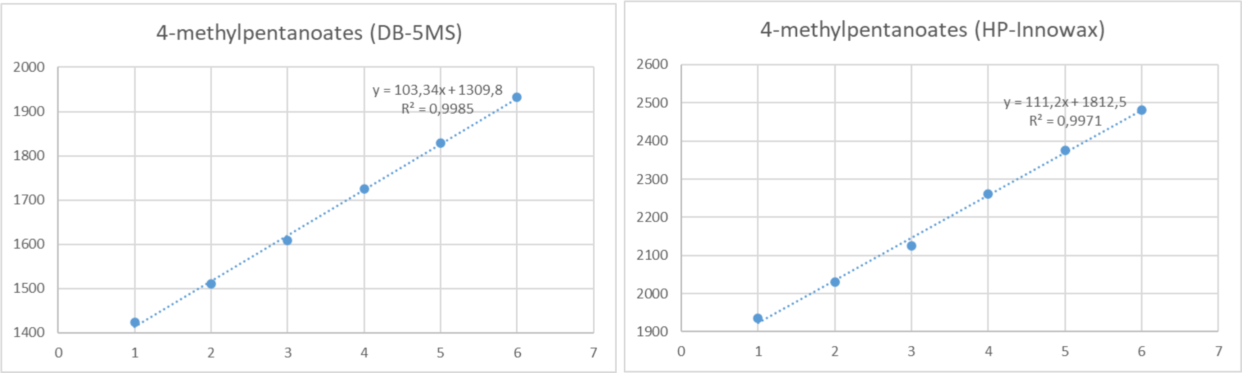

Figure S152. The dependence of RI values of the esters of the same acid (4-methylpentanoic acid) and phenol (**1**), benzyl alcohol (**2**), phenethyl alcohol (**3**), 3-phenylpropanol (**4**), 4-phenylbutanol (**5**), and 5-phenylpentanol (**6**)

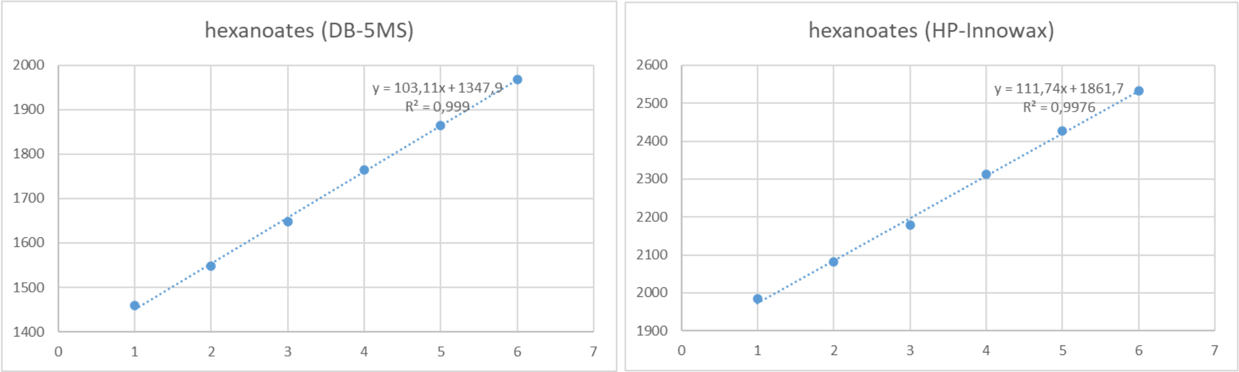

Figure S153. The dependence of RI values of the esters of the same acid (hexanoic acid) and phenol (**1**), benzyl alcohol (**2**), phenethyl alcohol (**3**), 3-phenylpropanol (**4**), 4-phenylbutanol (**5**), and 5-phenylpentanol (**6**)
